# Neuroprotective metabolites via fungal biotransformation of a novel sapogenin, cyclocephagenol

**DOI:** 10.1038/s41598-022-22799-5

**Published:** 2022-11-02

**Authors:** Melis Küçüksolak, Göklem Üner, Petek Ballar Kırmızıbayrak, Erdal Bedir

**Affiliations:** 1 Department of Bioengineering, Faculty of Engineering, İzmir Institute of Technology, Urla, 35430 İzmir, Türkiye; 2 Department of Biochemistry, Faculty of Pharmacy, Ege University, Bornova 35100 İzmir, Türkiye

**Keywords:** Drug screening, Medicinal chemistry

## Abstract

Cyclocephagenol (**1**), a novel cycloartane-type sapogenin with tetrahydropyran unit, is only encountered in *Astragalus* species. This rare sapogenin has never been a topic of biological activity or modification studies. The objectives of this study were; (i) to perform microbial transformation studies on cyclocephagenol (**1**) using *Astragalus* endophyte, *Alternaria eureka* 1E1BL1, followed by isolation and structural characterization of the metabolites; (ii) to investigate neuroprotective activities of the metabolites; (iii) to understand structure–activity relationships towards neuroprotection. The microbial transformation of cyclocephagenol (**1**) using *Alternaria eureka* resulted in the production of twenty-one (**2**–**22**) previously undescribed metabolites. Oxidation, monooxygenation, dehydration, methyl migration, epoxidation, and ring expansion reactions were observed on the triterpenoid skeleton. Structures of the compounds were established by 1D-, 2D-NMR, and HR-MS analyses. The neuroprotective activities of metabolites and parent compound (**1**) were evaluated against H_2_O_2_-induced cell injury. The structure–activity relationship (SAR) was established, and the results revealed that **1** and several other metabolites had potent neuroprotective activity. Further studies revealed that selected compounds reduced the amount of ROS and preserved the integrity of the mitochondrial membrane. This is the first report of microbial transformation of cyclocephagenol (**1**).

## Introduction

Biotransformation is the biochemical reactions performed by living systems or their components (enzymes) to alter molecules. Significant advantages of this methodology are; (i) stereo-, regio- and enantioselective catalysis; (ii) transformation at non-reactive sites of the substrates; (iii) mild condition requirements^1–6^. In the pharmaceutical industry, microbial biotransformation has been utilized in the enzymatic transformation to synthesize chiral intermediates and end products. Production of cortisone (*Rhizopus nigricans*), hydrocortisone (*Curvularia* sp.) and compactine (*Mucor hiemalis*) are examples of industrial applications of biotransformation where mainly P450 monooxygenases are involved. Additionally, biotransformation of natural products can provide a wide range of structural diversity and improved biological activity^7–12^.

Research groups have engaged in the discovery of new microorganisms and enzymes to be developed as novel biocatalysts. In this regard, endophytes are powerful organisms because of their capability to produce enzymes necessary for their colonization, and they have been proven to be potent biotransformation systems^3,13–15^.

Neurodegeneration refers to the loss of structure/function of neurons leading to neurological diseases including Alzheimer’s and Parkinson’s. The discovery of novel therapeutics against neurodegenerative diseases has been an area of intense research as neurodegenerative diseases are a huge burden on society and the economy^16^. Numerous studies reported that natural products have the potential for the prevention and treatment of neurodegeneration. Astragaloside IV (AST-IV), a cycloartane-type saponin from *Astragalus* species, efficiently attenuated hydrogen peroxide (H_2_O_2_)-induced neuronal cell death^17^. Moreover, aglycone of AST-IV, viz. cycloastragenol, diminished amyloid-beta mediated neurogenic disfunction^18^.

Herein, based on the potential of cycloartane-type saponins, we also focused on the neuroprotective activity of cyclocephagenol (**1**), a novel cycloartane-type sapogenin from *Astragalus microcephalus*. As **1** demonstrated significant protection, we further performed a modification study on **1** utilizing microbial transformation and examined the neuroprotective potential of metabolites in H_2_O_2_-induced injury in SH-SY5Y cells. As a result, the endophytic fungus *Alternaria eureka* 1E1BL1, an endophyte isolated from *Astragalus* plant, provided twenty-one new biotransformation products (**2**–**22**) with potent neuroprotective activities.

## Results

**Figure 1:**
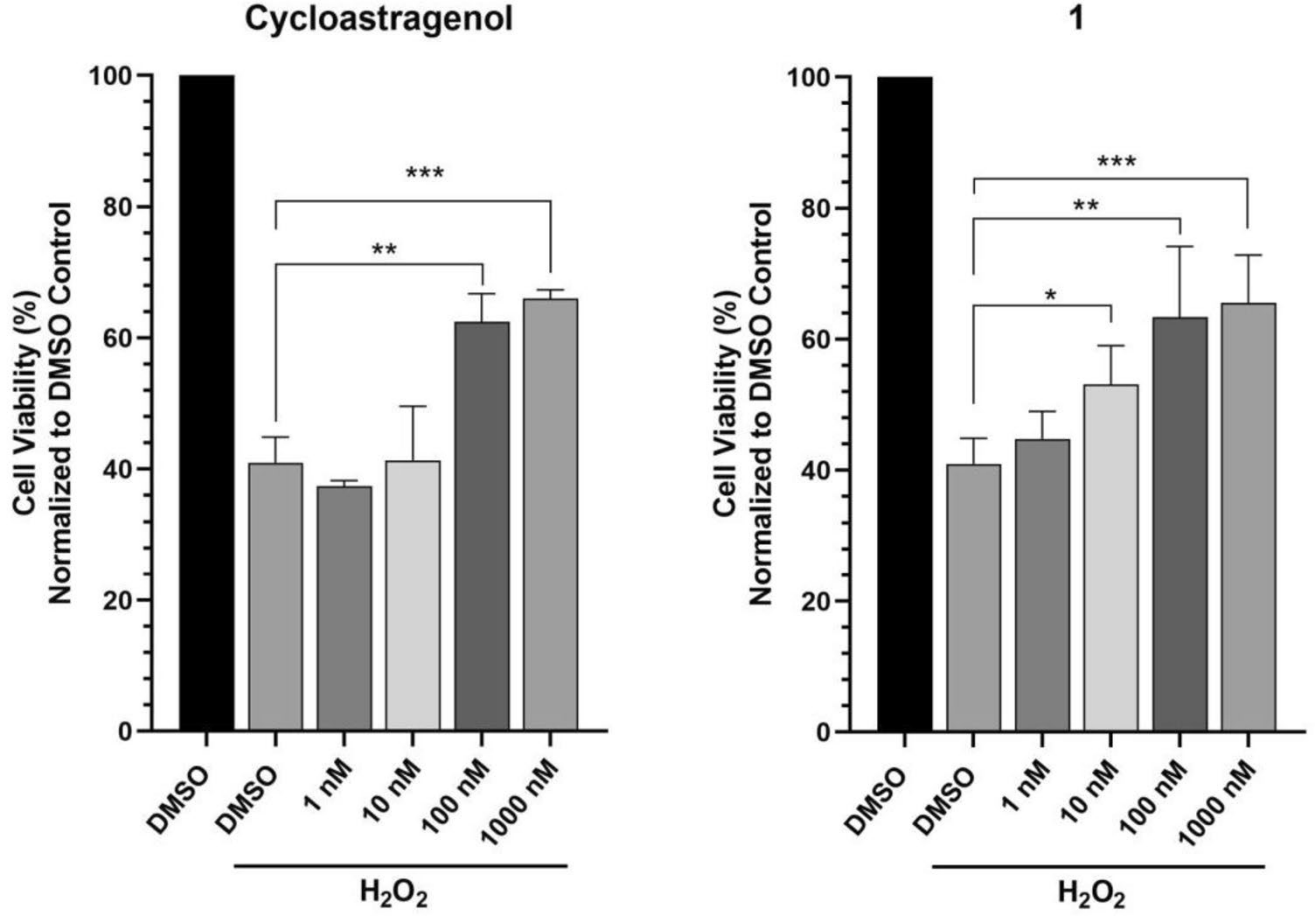
Neuroprotective activity of cycloastragenol and **1** against H_2_O_2_ toxicity. Error bars are the standard deviations (n = 3). p-Values were calculated with respect to H_2_O_2_-treated cells (*p < 0.05, **p < 0.01, ***p < 0.001).

**Figure 2:**
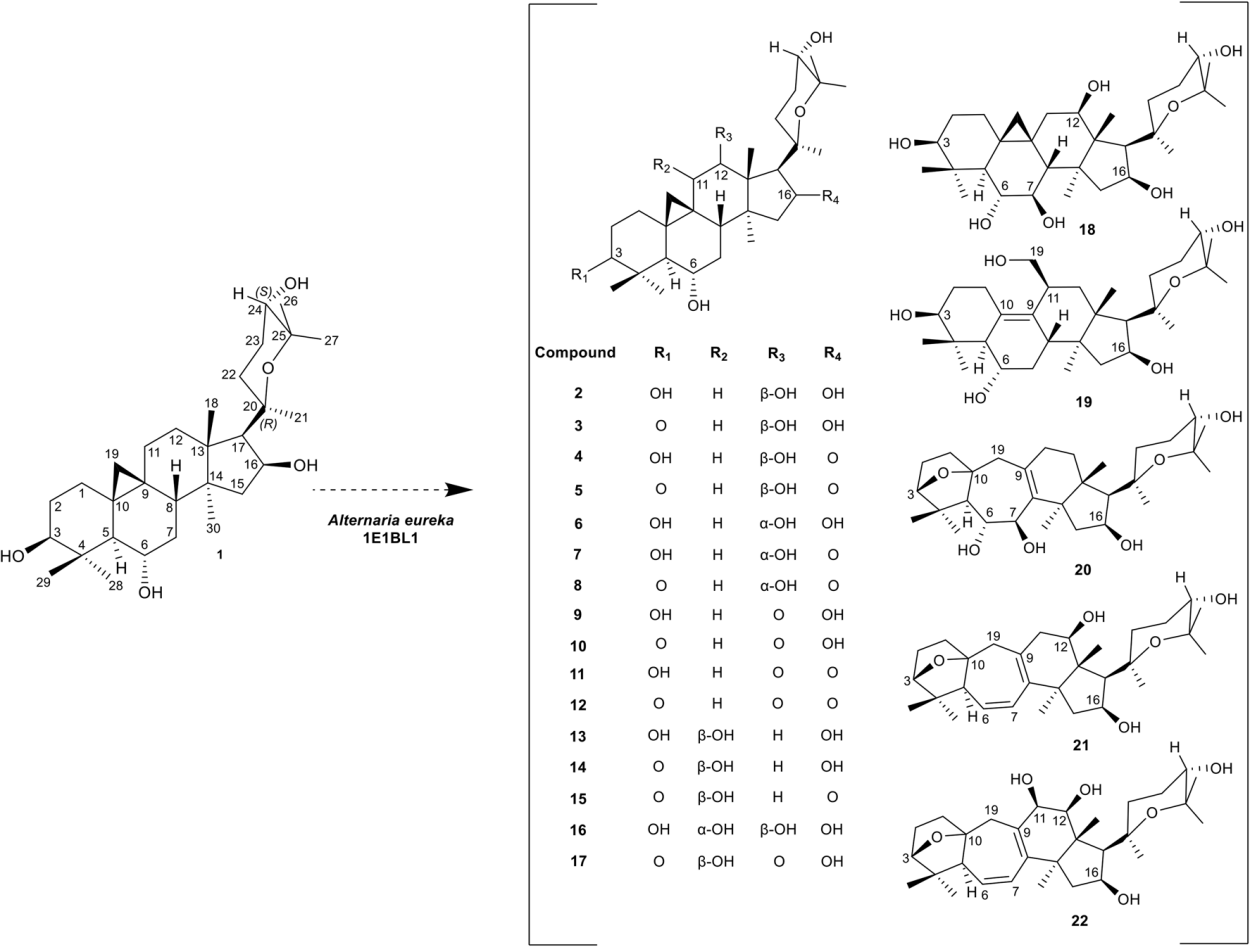
Biotransformation products of cyclocephagenol (**1**) by *Alternaria eureka* 1E1BL1.

The neuroprotective activities of cyclocephagenol (**1**) and cycloastragenol were determined against H_2_O_2_-induced SH-SY5Y cell death. Results showed that both cycloastragenol and **1** provided dose-dependent protection against H_2_O_2_-induced cell death (Fig. 1). However, the protective activity of **1** started at lower concentrations compared to cycloastragenol (Fig. 1). Based on the potent activity of **1**, a biotransformation study on **1** was carried out to develop a molecule library and to investigate structure–activity relationships by *A. eureka* affording notable chemical diversity^19–23^. Biotransformation of **1** using the endophytic fungus *A. eureka* for 13 days afforded twenty-one metabolites (**2–22**). The structures of the metabolites are shown in Fig. 2.

Compound **1** is a deglycosylated product of a known cycloartane diglycoside, namely cyclocephaloside I^24^. Since it has not been previously reported as a new sapogenin, herein, its structural elucidation is discussed. The molecular formula of **1** was determined as C_30_H_50_O_5_ due to the sodium adduct ion peak at *m/z* 513.35607 [M + Na]^+^ by HR-ESI–MS. The ^1^H-NMR spectrum showed characteristic signals of cyclopropane–methylene protons as an AX system at δ_H_ 0.30 and 0.58 (each d, *J*_AX_ = 4.1 Hz, H-19a and *J*_AX_ = 3.1 Hz H-19b) and seven tertiary methyl groups. Hence, compound **1** was a cycloartane-type triterpenoid, and this inspection was supported by the ^13^C-NMR spectral data. The resonances for low-field carbon atoms indicated the presence of four oxymethine carbons (δ_C_ 78.1, C-3; δ_C_ 68.3, C-6; δ_C_ 73.8, C-16; δ_C_ 68.5, C-24) and two oxygenated singlet carbons (δ_C_ 78.8, C-20; δ_C_ 75.1, C-25) substantiated by HSQC. The ^13^C and ^1^H-NMR data substantiated with 1D- and 2D-NMR spectra showed that resonances arising from the triterpenoid skeleton were almost superimposable with those of cycloastragenol^25^, which is one of the major aglycone constituents of *Astragalus* sp., except for the side chain signals. The HMBC experiment suggested that the 24-hydroxy-20,25-epoxy structure was intact in the side chain of **1**, as in cyclocephaloside I. The carbon resonances ascribed to the side chain consisting of a doublet (δ_C_ 68.5, C-24), two triplets (δ_C_ 26.4, C-22; 23.8, C-23), two singlets (δ_C_ 78.8, C-20; 75.1, C-25) and three quartets (δ_C_ 28.5, C-21; 28.4, C-26; 27.8; C-27). The HMBC spectrum displayed cross-peaks from H_3_-18 and H_3_-21 to C-17, H-17 and H_3_-21 to C-20, H-17 and H_3_-21 to C-22, H_3_-26 and H_3_-27 to C-24, and H_3_-26 and H_3_-27 to C-25 confirm this proposal. Thus, the structure of **1** was elucidated as 20,25-epoxy-3β,6α,16β,24α-tetrahydroxycycloartane, which is the aglycone of cyclocephaloside I^24^. Hence, **1** was named as cyclocephagenol.

### Oxygenation at C-7, C-11 and C-12

The HR-ESI–MS data of compounds **2**, **6** and **13** supported a molecular formula of C_30_H_50_O_6_ implying a monooxygenation of **1** due to a + 16 amu difference, while compounds **16** and **18** displayed a 32 amu increase over **1**, suggesting dihydroxy analogs.

The ^1^H-NMR spectra of **2** and **6** displayed a new oxymethine signal (δ_H_ 4.24 and 4.27, respectively). The ^13^C-NMR spectra of both compounds exhibited a significant down-field shift for neighbor carbon signals when compared to that of **1**, proposing a monooxygenation at the C-12 position: C-11 (δ_C_ 36.4) and C-13 (δ_C_ 52.3) signals underwent a significant down-field shift (ca. 10.2 and 5.7 ppm, respectively) for **2** and a down-field shift for C-17 and C-18 signals (ca. 9.2 and 7 ppm, respectively) for **6**. In the COSY spectra of both metabolites, the correlations between H-12 and H_2_-11 were readily noted. In addition, H-12 of **6** coupled with the exchangeable hydroxy proton [δ_H_ 5.38, C-12(OH)] to give a doublet of doublets of doublets type resonance (ddd, *J* = 9.1, 5.9, 2.6 Hz). This statement was verified with the HMBC experiment. The hydroxy group at C-12 was determined to be β-oriented for **2** and α-oriented for **6** based on NOESY correlations^26^. Based on this data, the structure of **2** and **6** were identified as 20,25-epoxy-3β,6α,12β,16β,24α-pentahydroxycycloartane and 20,25-epoxy-3β,6α,12α,16β,24α-pentahydroxycycloartane, respectively.

For metabolite **13**, as in the case of **2** and **6**, an additional oxymethine signal at δ_H_ 3.87 (dd, *J* = 8.7, 2.5 Hz) was observed, which correlated with a resonance at δ_C_ 65.4 in the HSQC spectrum. Furthermore, the ^1^H-NMR spectrum of **13** revealed that one of the 9,19-cyclopropane ring signals (δ_H_ 1.10, H-19a) underwent a significant downfield shift compared to **1**, indicating hydroxylation at the C-11 position as described previously^23,26,27^. The orientation of C-11(OH) was found to be β based on NOESY cross-peak between H-11 (δ_H_ 3.87) and α-oriented H_3_-30 (δ_H_ 0.84). Hence, the structure of **13** was established as 20,25-epoxy-3β,6α,11β,16β,24α-pentahydroxycycloartane.

When ^1^H- and ^13^C-NMR spectra of **16** were inspected in detail, two additional low-field protons (δ_H_ 3.35 and 3.93) were observed in the low-field region and two extra down-field carbon signals at δ_C_ 81.0 and 83.4 were also noted. The 2D-NMR spectra were examined in detail to deduce hydroxylation locations. Key long-range correlations from H_3_-18 (δ_H_ 1.35) and H-17 (δ_H_ 2.32) to 83.4 ppm substantiated that one of the hydroxy groups was located at C-12 based on the HMBC spectrum. The second hydroxy group was located at C-11 (δ_C_ 81.0) based on the COSY correlation of H-11 (δ_H_ 3.35) with H-12 (δ_H_ 3.93) together with the ^3^*J*_H-C_ correlations from H_2_-19 (δ_H_ 0.57 and 0.83) and H-12 (δ_H_ 3.93) to C-11. The relative configurations at C-11 and C-12 were determined based on the 2D-NOESY data. The orientation of C-12(OH) was found to be β based on NOESY cross-peak between H-12 (δ_H_ 3.93) and α-oriented H_3_-30. The strong correlation of H-11 with one of the β-oriented C-19 protons (δ_H-19a_ 0.83) revealed that the hydroxy group at C-11 was α-oriented. Based on this evidence, the structure of compound **16** was elucidated as 20,25-epoxy-3β,6α,11α,12β,16β,24α-hexahydroxycycloartane.

Compound **18** was the dihydroxylated derivative of **1**, and the first hydroxylation was located at C-12 based on the ^3^*J*_H-C_ correlations from the characteristic H-17 (δ_H_ 2.53) and H_3_-18 (δ_H_ 1.88) resonances to the new signal at δ_C_ 71.4 (C-12) in the HMBC spectrum. The ^13^C-NMR spectrum of **18** displayed that C-6 (δ_C_ 73.2) and C-8 (δ_C_ 52.4) signals underwent a significant down-field shift (ca. 4.9 and 5.3 ppm, respectively) when compared to **1**. Together with the spin system starting from H-5 [H-5 (δ_H_ 1.80 d, *J* = 9.3 Hz) → H-6 (δ_H_ 3.74) → H-7 (δ_H_ 3.71 s) → H-8 (δ_H_ 2.40 d, *J* = 6.9 Hz)] in the COSY spectrum, the location of the second hydroxylation was deduced to be C-7. Long-range correlations from H-6 and H-8 to C-7 (δ_C_ 75.7) in the HMBC spectrum verified the location of the transformation^22,23^. From a detailed inspection of the 2D-NOESY spectrum, the correlations of H-12 with α-oriented H_3_-30 (δ_H_ 1.03)/H-17 (δ_H_ 2.53) and cross-peaks between H-7 and α-oriented H_3_-30/H-5 (δ_H_ 1.80) disclosed the configurations of hydroxy groups. Consequently, metabolite **18** was elucidated as 20,25-epoxy-3β,6α,7β,12β,16β,24α-hexahydroxycycloartane.

### Additional oxidation at C-3, C-12 and C-16

All keto products isolated within the scope of this study also had oxygenations as in the abovementioned compounds. ^1^H-, ^13^C-NMR and HMBC correlations were inspected to assign locations of oxidation and oxygenation. In the ^1^H- and ^13^C-NMR spectra of oxidation products, the absence of low-field oxymethine signals and the observation of keto carbonyl carbons around 210–220 ppm suggested the oxidation of secondary alcohols. In the HMBC spectrum, H_3_-28, H_3_-29, H_2_-1 and H_2_-2 displayed cross-peaks with the carbonyl signal at 210–220 ppm region substantiating the existence of the carbonyl group at C-3 while the long-range correlations from H-17 and H_2_-15 to a carbon resonance at ca. 210–220 ppm confirmed the location of the carbonyl group to be at C-16^20,23,26^.

Thus, the assignment of oxidation locations for compounds **3** (3-oxo), **4** (16-oxo) and **5** (3,16-dioxo) was readily inferred based on the abovementioned evidence. Additionally, a new proton signal (δ_H_ 4.18 for **3**; δ_H_ 4.16 for **4**; δ_H_ 4.14 for **5**) in the ^1^H-NMR spectra of **3–5** and a new carbon resonance (δ_C_ 71.6 for **3**; δ_C_ 71.5 for **4**; δ_C_ 71.6 for **5**) in the ^13^C-NMR spectrum suggested a new hydroxy group in the structure. Examination of 2D-NMR spectra of **3**–**5** verified monooxygenation at C-12 and the relative configuration was established as β-oriented. Thus, metabolites **3**, **4** and **5** were determined as 20,25-epoxy-6α,12β,16β,24α-tetrahydroxycycloartan-3-one, 20,25-epoxy-3β,6α,12β,24α-tetrahydroxycycloartan-16-one and 20,25-epoxy-6α,12β,24α-trihydroxycycloartan-3,16-dione, respectively.

Like metabolites **3**–**5**, after positions of oxidation were determined unambiguously (C-16 for **7**; C-3 and C-16 for **8**), ^1^H- and ^13^C-NMR spectra of **7** and **8** were further examined. A new proton signal (δ_H_ 4.19 for **7**; δ_H_ 4.15 for **8**) and a new carbon signal (δ_C_ 72.7 for **7**; δ_C_ 73.7 for **8**) suggested that a monooxygenation reaction took place. The 2D-NMR spectra of **7** and **8** implied the location of monooxygenation at C-12 as in metabolites **3**–**5**; however, 2D-NOESY data revealed that the hydroxy group at C-12 was α-oriented in **7** and **8**. Therefore, metabolites **7** and **8** were deduced to be 20,25-epoxy-3β,6α,12α,24α-tetrahydroxycycloartan-16-one and 20,25-epoxy-6α,12α,24α-trihydroxycycloartan-3,16-dione, respectively.

Compound **9** gave a major ion peak at *m/z* 527.33662 ([M + Na]^+^, calcd for C_30_H_48_NaO_6_, 527.33486). When the ^1^H-NMR spectrum of **9** was inspected, the characteristic signals belonging to H-3, H-6, H-16 and H-24 oxymethine protons were observed readily, suggesting that oxidation occurred in a new oxymethine carbon. In the HMBC spectrum, H_2_-11 (δ_H_ 1.97 and 2.61), H_3_-18 (δ_H_ 1.72) and H-17 (δ_H_ 2.36) showed cross-peaks with a carbon resonating at δ_C_ 212.0, confirming the location of the carbonyl group to be at C-12. Based on these results, the structure of **9** was elucidated as 20,25-epoxy-3β,6α,16β,24α-tetrahydroxycycloartan-12-one.

When the ^13^C-NMR and HMBC spectra of **10** and **11** were examined, signals at δ_C_ 210–220 range suggested two keto carbonyl groups in the structure of both compounds. The HMBC correlations from H_2_-11 (δ_H_ 1.96 and 2.64) and H_3_-18 (δ_H_ 1.73) to δ_C_ 211.3 for **10** and H_2_-11 (δ_H_ 2.09 and 2.83) and H_3_-18 (δ_H_ 2.06) to δ_C_ 210.2 for **11** confirmed the location of the first ketone group to be at C-12. Moreover, the low-field signal of H-3 in **10** was absent in the ^1^H-NMR spectrum, whereas the H-16 signal disappeared in that of **11**. The carbon signal δ_C_ 216.1 had long-range correlations with H_3_-28 and H_3_-29, readily assigned to C-3 in the HMBC spectrum of **10**, while the HMBC correlations of H-17 (δ_H_ 3.08) and H_2_-15 (δ_H_ 2.18 and 2.52) with the carbonyl carbon at δ_C_ 214.4 confirmed the oxidation at C-16 to establish the structure of **11**. Hence, the structure of **10** was deduced as 20,25-epoxy-6α,16β,24α-trihydroxycycloartan-3,12-dione, and the structure of **11** was established as 20,25-epoxy-3β,6α,24α-trihydroxycycloartan-12,16-dione.

The metabolite **12** gave a molecular formula of C_30_H_44_O_6_ based on the HR-ESI–MS data (*m/z* 523.30530 ([M + Na]^+^, calcd for C_30_H_44_NaO_6_, 523.30356). The absence of low-field oxymethine signals due to H-3 and H-16 in the ^1^H-NMR spectrum and observation of two keto carbonyl carbons in the ^13^C-NMR and HMBC spectra suggested that C-3 (δ_C_ 215.7) and C-16 (δ_C_ 214.1) secondary alcohols had been oxidized, as in **5**. Also, the signal at δ_C_ 209.6 suggested an additional keto carbonyl group in structure, which showed cross-peaks with H_2_-11 (δ_H_ 2.05 and 2.83), H-17 (δ_H_ 3.07) and H-18 (δ_H_ 2.06) in the HMBC spectrum, revealing the oxidation at C-12. Based on these results, the structure of **12** was elucidated as 20,25-epoxy-6α,24α-dihydroxycycloartan-3,12,16-trione.

Compounds **14** and **15** possessing similar oxidation patterns with metabolites **3** and **5**, respectively, were determined as 11β-hydroxycyclocephagenol derivatives by comparing 1D- and 2D-NMR spectra of **14** and **15** with **13**. Consequently, the structures of **14** and **15** were determined as 20,25-epoxy-6α,11β,16β,24α-tetrahydroxycycloartan-3-one and 20,25-epoxy-6α,11β,24α-trihydroxycycloartan-3,16-dione, respectively.

The HR-ESI–MS spectrum of **17** showed a major ion peak at *m/z* 541.31528 [M + Na]^+^ (C_30_H_46_NaO_7_). The oxymethine proton at C-3 was lost in the ^1^H-NMR spectrum. A detailed inspection of ^13^C-NMR and HMBC spectra suggested that the C-3 (δ_C_ 216.3) secondary alcohol had been oxidized and the signal at δ_C_ 211.4 suggested an additional keto carbonyl group in **17**, as in **10**. Unlike metabolite **10**, a broad singlet observed at δ_H_ 3.71 displaying a long-distance correlation with the C-12 (δ_C_ 211.4) suggested an additional oxymethine group. With these findings, a monooxygenation at C-11 was suggested. The configuration of C-11(OH) was deduced based on the 2D-NOESY data. The correlation of H-11 with α-oriented H_3_-30 (δ_H_ 0.66) revealed that the configuration of the OH group at C-11 was β-oriented. Thus, the structure of **17** was determined to be 20,25-epoxy-6α,11β,16β,24α-tetrahydroxycycloartan-3,12-dione.

### Ring cleavage and ring expansion products

In the ^1^H-NMR spectra of metabolites **19**, **20**, **21** and **22**, the characteristic 9,19-cyclopropane ring signals were absent, suggesting a ring cleavage reaction in the triterpenoid skeleton.

The metabolite **19** gave a molecular formula of C_30_H_50_O_6_ based on the HR-ESI–MS data (*m/z* 529.35144 [M + Na]^+^, calcd for C_30_H_50_NaO_6_, 529.35051) indicating a 16 amu increase over **1**, suggesting being a monohydroxy derivative. An additional oxymethylene group at δ_C_ 68.5 and two olefinic carbon signals (δ_C_ 134.5, 132.7) were observed in the low field of the ^13^C-NMR spectrum. In the HSQC spectrum, double bond carbons showed no correlation with any proton, denoted a tetrasubstituted olefinic system. Based on these findings, the structure of **19** was proposed to have a C-9(10) double bond with a primary alcohol substitution at C-11 based on our previous biotransformation studies, and this assumption was confirmed by the HMBC experiment^20,23,27,28^. In conclusion, metabolite **19** was determined to be 20,25-epoxy-3β,6α,16β,19,24α-pentahydroxy-ranunculan-9(10)-ene.

The HMBC spectra of **20**, **21** and **22** displayed a cross-peak between H-3 (δ_H_ 3.82) and C-10 (δ_C_ 88.7), indicative of an oxygen bridge between C-3, which correlated with H_3_-28 and H_3_-29, and C-10. Based on this evidence and previous biotransformation studies^20,29^, ring B was inferred as a seven-membered ring system. Also, olefinic carbon resonances were detected in the ^13^C-NMR spectra. The nature of double bonds was determined by examining the correlation of olefinic carbons with protons in the HSQC spectra.

The molecular formula of **20** was established as C_30_H_48_O_6_ by HR-ESI–MS analysis (*m/z* 527.33874 [M + Na]^+^, calcd for C_30_H_48_NaO_6_, 527.33486). In the ^13^C-NMR spectrum, two additional oxymethine carbons at δ_C_ 80.2 and 88.7 and two olefinic carbon resonances at δ_C_ 126.7 and 139.5 were detected. From the HSQC spectrum, the nature of the double bond was determined as a tetra-substituted olefinic system. The δ_C_ 126.7 and 139.5 resonances were assigned to C-9 and C-8, respectively, based on the long-distance correlations in the HMBC spectrum (C-9 to H_2_-12; C-8 to H_2_-12 and H_3_-30). The resonance observed at δ_H_ 4.39 suggested an additional oxymethine group corresponding to carbon at δ_C_ 80.2 in the HSQC spectrum. A detailed inspection of the ^1^H- and ^13^C-NMR spectra showed down-field shifts for H-6 and C-6 signals (ca. 0.64 and 8.6 ppm, respectively) when compared to that of **1**; therefore, a hydroxylation at C-7 was suggested. The COSY and HSQC spectra revealed a spin system of H-5 (δ_H_ 1.47) → H-6 (δ_H_ 4.42) → H-7 (δ_H_ 4.39), justifying this assignment. In the 2D-NOESY spectrum, the orientation of C-7(OH) was found to be β based on NOESY cross-peaks between H-7 (δ_H_ 4.39) and the α-oriented H_3_-30 and H-5. Consequently, the structure of **20** was established as 3β,10β;20,25-diepoxy-6α,7β,16β,24α-tetrahydroxy-9,10-seco-cycloartan-8-ene.

The HR-ESI–MS data of metabolite **21** displayed a sodium adduct ion at *m/z* 509.32640 [M + Na]^+^ (calcd. 509.32429 for C_30_H_46_NaO_5_). The oxymethine proton at C-6 was absent in the ^1^H-NMR spectrum. Also, an additional hydroxymethine signal at δ_H_ 4.45 was observed corresponding to carbon at δ_C_ 70.1 in the HSQC spectrum. The ^13^C-NMR spectrum of **21** exhibited four olefinic carbon resonances (δ_C_ 128.7, 131.8, 132.1 and 138.5). Locations of the olefinic double bonds were assigned from the correlations in the HMBC spectrum and with the combined use of COSY, HSQC and HSQC-TOCSY spectra. The long-distance correlation from H-5 (δ_H_ 1.96) to the olefinic methine carbon at δ_C_ 128.7 confirmed the location of the double bond between C-6 and C-7. The other tetrasubstituted double bond was positioned between C-8 and C-9 based on the HMBC correlations from H_2_-11a to the olefinic carbon at δ_C_ 131.8 (C-9), and H_2_-11a and H_3_-30 to the second double bond carbon at 138.5 ppm (C-8). The ^3^*J*-HMBC correlations of the oxymethine proton at δ_H_ 4.45 with the C-18 signal revealed oxygenation at C-12. The hydroxy group at C-12 was deduced to be β-oriented based on the NOESY correlation of H-12 (δ_H_ 4.45) with the α-oriented H_3_-30 (δ_H_ 0.94). As a result, metabolite **21** was established as 3β,10β;20,25-diepoxy-12β,16β,24α-trihydroxy-9,10-seco-cycloartan-6,8-diene.

Compound **22** gave a major ion peak at *m/z* 525.3166 ([M + Na]^+^, calcd for C_30_H_46_NaO_6_, 525.31921). Its NMR spectra were very similar to those of **21**, except for the resonances corresponding to ring C. A new low-field proton (δ_H_ 4.73) was observed in the ^1^H-NMR spectrum. Besides, in the ^13^C-NMR spectrum, an extra down-field carbon signal at δ_C_ 70.8 was noted. In the COSY and HSQC-TOCSY spectra, the δ_H_ 4.73 proton coupled with an oxymethine proton at C-12 (δ_H_ 4.36) had a long-range correlation with C-18 (δ_H_ 16.1) in the HMBC spectrum. Based on these data, a new hydroxy group was undeniably located at C-11. The NOE correlation between H-11 and α-oriented H-12 indicated the β-configuration of 11-OH. Consequently, the structure of **22** was determined to be 3β,10β;20,25-diepoxy-11β,12β,16β,24α-tetrahydroxy-3(10)β-epoxy-9,10-seco-cycloartan-6,8-diene.

### Neuroprotective activity of biotransformation products

The neuroprotective activities of isolated metabolites (except for **22** due to its scarce amount) were determined against H_2_O_2_-induced SH-SY5Y cell death. Compared to the control group, the compounds except **2**, **3**, **4**, **6**, **7**, **9**, **10**, **13**, **14**, **17**, and **20** did not exhibit promising neuroprotective activity (Table 1).

**Table 1:** Neuroprotective activity of metabolites against H_2_O_2_ toxicity. Data are presented as means ± S.D (n = 3). *p < 0.05, **p < 0.05, ***p < 0.001, ****p < 0.0001 significant difference from H_2_O_2_-treated cells.

Metabolite	Concentration	Cell viability (%)	Metabolite	Concentration	Cell viability (%)
**2**	1 nM	56.2 ± 1.8****	**12**	1 nM	36.2 ± 1.9
	10 nM	61.4 ± 3.7****		10 nM	46.2 ± 1.8*
	100 nM	61.1 ± 0.7****		100 nM	49.0 ± 6.2**
	1000 nM	58.1 ± 5.9****		1000 nM	42.4 ± 2.0
**3**	1 nM	46.8 ± 6.0	**13**	1 nM	51.5 ± 7.9
	10 nM	46.7 ± 2.1		10 nM	56.4 ± 3.0*
	100 nM	50.9 ± 0.2**		100 nM	58.1 ± 12.1*
	1000 nM	65.9 ± 2.3****		1000 nM	66.4 ± 9.3**
**4**	1 nM	34.1 ± 0.4	**14**	1 nM	60.8 ± 4.4****
	10 nM	59.7 ± 3.5****		10 nM	56.5 ± 4.2****
	100 nM	56.3 ± 3.3****		100 nM	39.9 ± 2.7
	1000 nM	53.6 ± 5.9****		1000 nM	34.1 ± 1.3
**5**	1 nM	26.9 ± 2.5	**15**	1 nM	37.0 ± 3.0
	10 nM	25.1 ± 3.6		10 nM	41.2 ± 2.6
	100 nM	43.8 ± 1.3		100 nM	43.5 ± 5.3*
	1000 nM	42.5 ± 5.7		1000 nM	37.0 ± 0.5
**6**	1 nM	45.7 ± 2.4	**16**	1 nM	42.0 ± 2.3
	10 nM	46.0 ± 5.9		10 nM	46.5 ± 7.2
	100 nM	54.8 ± 5.8***		100 nM	42.7 ± 3.1
	1000 nM	52.1 ± 1.3*		1000 nM	41.4 ± 6.6
**7**	1 nM	42.6 ± 3.9	**17**	1 nM	41.9 ± 1.4
	10 nM	45.9 ± 5.6		10 nM	42.7 ± 2.3
	100 nM	55.6 ± 13.3*		100 nM	62.8 ± 7.9***
	1000 nM	49.0 ± 3.9		1000 nM	72.5 ± 13.3****
**8**	1 nM	36.6 ± 11.0	**18**	1 nM	44.0 ± 0.7
	10 nM	40.3 ± 1.5		10 nM	42.0 ± 0.8
	100 nM	36.6 ± 2.7		100 nM	44.4 ± 0.9
	1000 nM	40.2 ± 12.9		1000 nM	38.1 ± 4.8
**9**	1 nM	44.2 ± 2.3	**19**	1 nM	38.0 ± 1.9
	10 nM	46.2 ± 3.2		10 nM	40.5 ± 7.1
	100 nM	51.4 ± 4.8*		100 nM	39.9 ± 4.7
	1000 nM	70.5 ± 0.8****		1000 nM	49.1 ± 3.6
**10**	1 nM	51.1 ± 8.5*	**20**	1 nM	56.8 ± 1.9**
	10 nM	58.5 ± 3.4***		10 nM	58.3 ± 6.3**
	100 nM	74.7 ± 3.3****		100 nM	70.6 ± 6.9****
	1000 nM	68.1 ± 4.8****		1000 nM	64.8 ± 6.0****
**11**	1 nM	38.2 ± 0.7	**21**	1 nM	47.0 ± 1.6**
	10 nM	43.9 ± 6.2		10 nM	44.7 ± 2.4*
	100 nM	46.9 ± 9.5		100 nM	38.7 ± 3.1
	1000 nM	54.3 ± 3.7**		1000 nM	35.7 ± 1.4
**H**_**2**_**O**_**2**_	70 µM	37.9 ± 2.0			

In addition to their protective effect against oxidative injury in SH-SY5Y, the selected compounds were also screened for their effect against 6-OHDA induced neurotoxicity in in vitro Parkinson’s disease model. Despite the lower potency of compounds in this model compared to the H_2_O_2_-induced neurotoxicity model, the tested metabolites still showed a statistically significant protective effect, suggesting that they may act as general protective agents against neurotoxicity mediated by a different mechanism of action (Fig. S213).

### Selected compounds decreased H_2_O_2_-mediated oxidative stress

**Figure 3:**
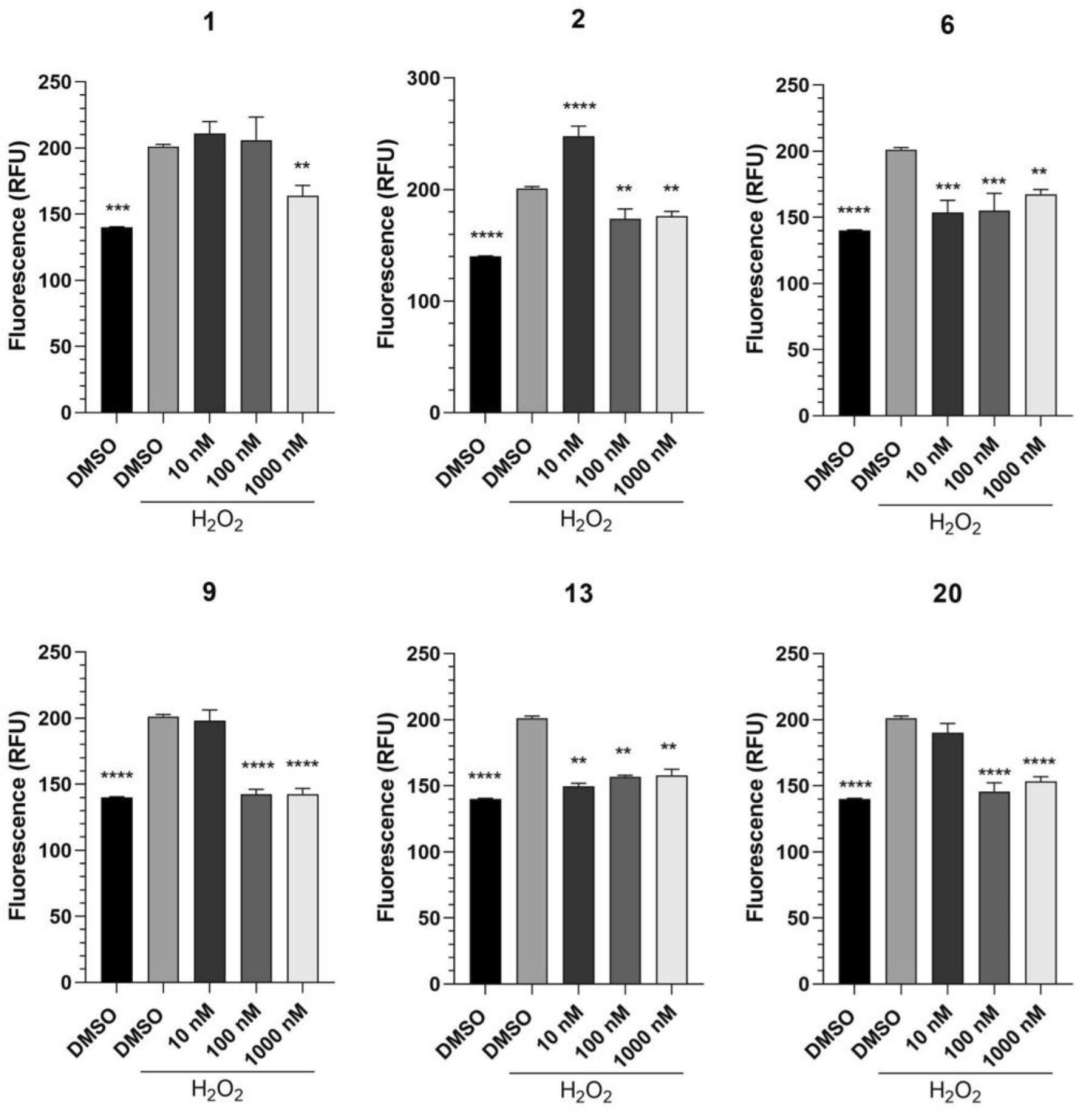
Selected metabolites abbreviated H_2_O_2_-induced oxidative stress in SH-SY5Y cells. Data are presented as means ± S.D. (n = 3).

Excessive ROS was reported to cause severe cell damage and induce cell death^30^. Since H_2_O_2_ is well known to increase the level of ROS, we aimed to determine the effect of compounds on H_2_O_2_-mediated increased ROS levels. Six molecules, including parent compound (**1**), were selected to detect their potential in rescuing H_2_O_2_-induced oxidative stress, considering their cell viability results, structure, and available quantity. Results showed that treatment with H_2_O_2_ significantly increased ROS levels compared to control. In line with the cell viability assay, all selected compounds reduced H_2_O_2_-induced ROS levels in cells (Fig. 3). Parent **1** was the least potent, while **6** and **13** were the most effective compounds reducing ROS at all concentrations (Fig. 3). Interestingly, **2** enhanced ROS production via H_2_O_2_ at 10 nM, but higher concentrations significantly decreased ROS level (Fig. 3).

### Selected metabolites prevent H_2_O_2_-induced mitochondria damage

**Figure 4:**
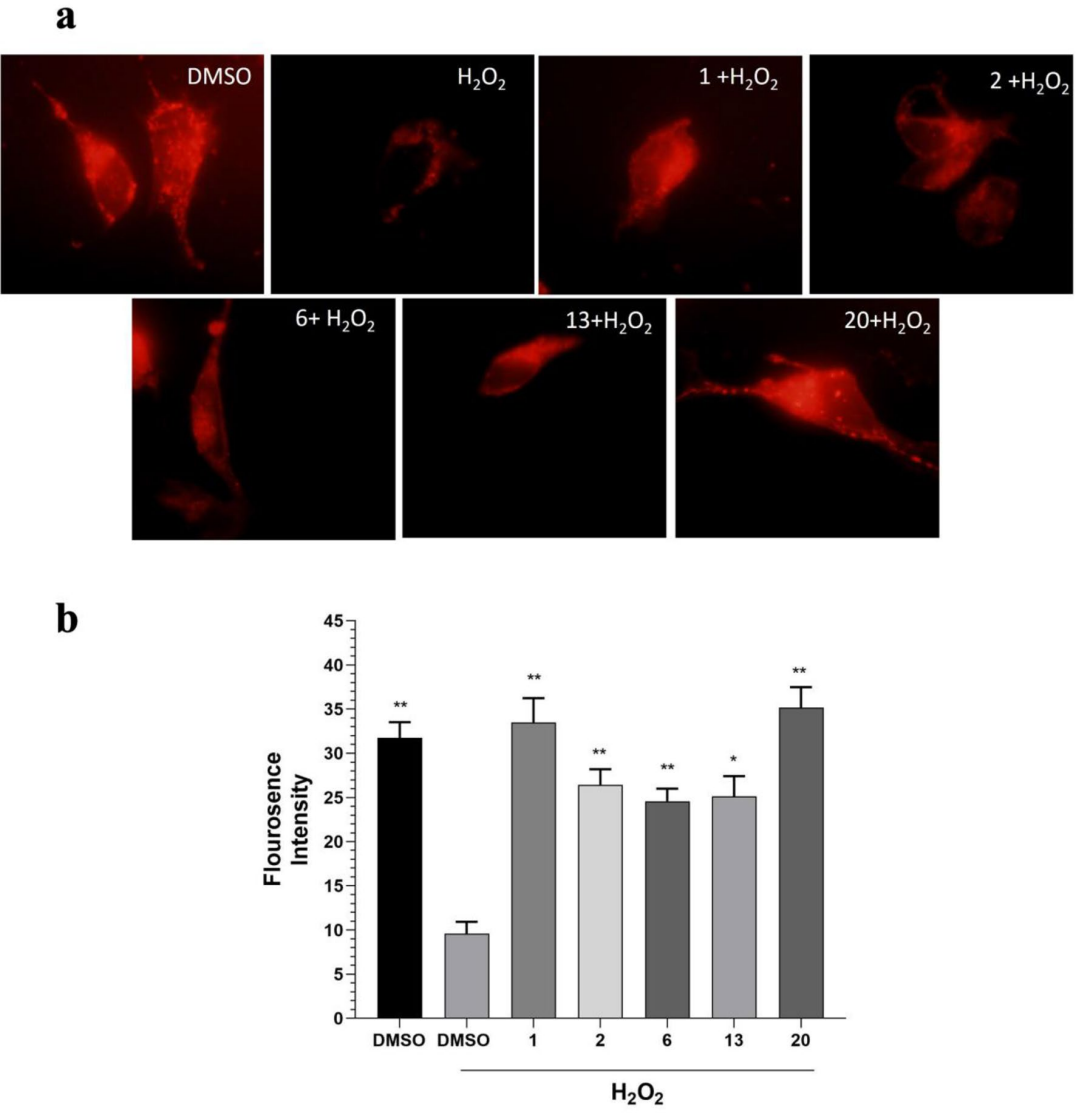
Selected metabolites protect SH-SY5Y cells from H_2_O_2_-induced mitochondrial damage. (**a**) Representative microscopy images of cells stained with MitoTracker Red. (**b**) Quantification of MitoTracker Red fluorescence intensities per cell volume (n = 30 cells). Data are presented as means ± S.E. (n = 30).

Mitochondrial dysfunction is one of the most emerging pathological processes in neurodegenerative diseases^31^. Since mitochondrial membrane potential was reported as an indicator to detect mitochondrial dysfunction, we next evaluated the effect of **1** and four selected compounds on mitochondrial membrane potential by using Mitotracker Red. As expected, the fluorescence intensity significantly decreased after treatment with H_2_O_2_ treatment, which suggested that H_2_O_2_ could induce mitochondrial dysfunction. All selected compounds efficiently protected cells from H_2_O_2_-mediated mitochondrial damage (Fig. 4a,b).

## Discussion

In recent years, endophytic fungi have received great attention as a whole-cell catalyst because of their capability to produce enzymes necessary for their colonization. It is anticipated that the endophytic biocatalysts will match chemical reactions even as powerful as conventional chemical methods in near future^3,13–15^. Additionally, studies on fungal biotransformation of plant secondary metabolites with the plant’s own endophytes are very limited. Our previous studies demonstrated that *Alternaria eureka* expresses P450 monooxygenase enzymes and effectively catalyzes various modifications on triterpenoid and steroid structures^19–23^. In this study, the biocatalysis of cyclocephagenol (**1**) by *A. eureka* yielded twenty-one metabolites. *A. eureka* was found to be capable of modifications including monooxygenation, dehydration, methyl migration, epoxidation, and ring expansion resulted in the formation of metabolites that would be difficult or impossible to prepare by conventional synthetic methods. Although fungal biotransformation has been used in the modification of natural products for a long time, demonstration of endophytic fungi's utilization in biotransformation is essential for the field not only for utilizing endophytes as potent catalysis systems but also for proving the potential of the plant's own microbiota for transformation studies.

The regioselective hydroxylation at C-12 position was the most prevalent reaction in the cycloartane skeleton. We suggest that *A. eureka* first catalyzes α- or β-hydroxylation at C-12, then performs further modifications. The other monooxygenation locations were identified as C-7 and C-11. Although hydroxylation at C-7 has been encountered with steroidal sapogenins^19,21–23^, this modification is reported for the first time on the triterpenoid framework by *A. eureka*.

One of the most interesting biotransformation was the dihydroxylation at C-11 and C-12. Previously, 11α-hydroxy,12β-acetoxy steroids were obtained from the gorgonian *Isis hippuris*^32^. This is the first report of C-11 and C-12 dihydroxylated products obtained via microbial biotransformation. Additionally, 3(10)-epoxy formation and ring expansion modifications have been reported in the previous studies^20,29,33^, whereas the 6,8-diene system is being reported for the first time in triterpenoid chemistry.

Natural products have the potential for the prevention and treatment of neurodegeneration. In recent years, cycloartane-type saponins, especially astragaloside IV and cycloastragenol, have been reported as a new class of neuroprotective agents^17,18^. Based on the promising bioactivity of cycloartane-types saponins, we first evaluated the neuroprotective activity of cyclocephagenol (**1**) against H_2_O_2_-induced SH-SY5Y cell death. Compared to cycloastragenol, the neuroprotective activity of **1** started at lower concentrations. Since the neuroprotective activity of cyclocephagenol was noteworthy, a biotransformation study on **1** was carried out to develop a molecule library and to investigate structure–activity relationships by *A. eureka*.

Among the oxygenated metabolites, **2** and **6** possessing hydroxy group at position 12 (12β and 12α, respectively) showed potent neuroprotective activity. While the most active concentration of **2** was 10 nM, the neuroprotection by **6** was observed at a higher concentration (100 nM). Also, the metabolite **13** possessing hydroxy group at position 11 demonstrated potent neuroprotective activity at higher concentrations (100 and 1000 nM). On the other hand, a dihydroxy group in ring C (**16**; 11α,12β-dihydroxy) led to a loss of neuroprotection suggesting that the hydroxylation pattern was critical for the activity.

The presence of ketone functionality at C-3 or C-16 (**3**: 3-oxo-12β-hydroxy, **4**: 16-oxo-12β-hydroxy, **7**: 16-oxo-12α-hydroxy) had no detrimental effect on neuroprotection compared to metabolite **2** and **6**. However, the co-presence of ketone group at C-3 and C-16 (**5**: 3,16-dioxo-12β-hydroxy, **8**: 3,16-dioxo-12α-hydroxy) diminished neuroprotective activity. Moreover, the oxidation at C-12 (**9**: 12-oxo, 1000 nM) improved neuroprotective activity whereas additional oxidation at C-16 (**11**: 12,16-dioxo) lessened neuroprotective activity. This finding suggested that the co-presence of ketone group at C-12 and C-16 affects the biological activity negatively. In contrast to metabolite **3** (3-oxo-12β-hydroxy), oxidation at position 3 improved neuroprotective activity in **14** (3-oxo,11β-hydroxy) and **10** (3,12-dioxo). Although metabolite **16** (11α,12β-dihydroxy) was not active, activity in **17** (3,12-dioxo, 11β-hydroxy) indicated that oxidation in C-12 was important for biological activity.

Compound **20** was one of the most potent compounds, while neuroprotection was considerably decreased in **21**, a dehydration product of **20**, revealing that conformational flexibility in the B ring was also crucial.

As a result of SAR studies, we conclude that i) monooxygenation at positions 11 (**13**) and 12 (**2** and **6**) is significant bioactivity; ii) oxidation at C-12 (**9**, **10**, **17**) improves neuroprotective activity; iii) further increase of hydrophobicity (**5**, **8**, **11**, **12**) and hydrophilicity (**16**, **18**) diminishes bioactivity; iv) 3(10)β-epoxy-9,10-seco-cycloartane products formed by ring expansion and epoxidation reactions could be potential neuroprotective agents as long as they have conformational flexibility. Further studies revealed that selected compounds reduced the amount of ROS and preserved the integrity of the mitochondrial membrane.

## Conclusion

Collectively, the biocatalyst potential of *A. eureka*, which plays an important role in expanding our cycloartane molecule library, has been demonstrated once again by producing 21 new metabolites. In addition to chemical diversity, biotransformation provided several novel compounds having potent protective activity against H_2_O_2_- and 6-OHDA-induced neurotoxicity. Further studies are warranted to establish a mechanism of action of the bioactive metabolites.

## Methods

### General experimental procedures

The spectroscopic (NMR and HR-ESI–MS) and chromatographic procedures were described previously^26^.

### Microorganism and starting compound

Cyclocephagenol (**1**) and cycloastragenol were provided by Bionorm Natural Products, Ltd. (İzmir, Turkey). The fungal endophyte used in this study was isolated from leaves of *Astragalus angustifolius* and the original strain (Deposit number: 20131E1BL1) was banked at the Bedir Laboratory^20^. Before biotransformation process, the stock culture of *A. eureka* was pre-cultivated on PDA in Petri dishes for 10 days at 25 °C.

### Microbial biotransformation procedures

A one-stage microbial biotransformation process was carried out at a preparative scale using a biotransformation medium^26^. A preparative scale biotransformation study was performed utilizing 1850 mg of **1** with *A. eureka* for 13 days (25 °C and 180 rpm).

### Extraction and isolation

For termination biotransformation, the mycelia were filtered, and the broth was extracted with ethyl acetate (EtOAc) (3×). The EtOAc phase was evaporated by using a rotary evaporator. Compounds **2**–**22** were isolated from the EtOAc extract (3.05 g). The EtOAc extract was first applied on a reversed-phase column (RP-C18, 80 g) and eluted by MeOH:H_2_O (25:75, 35:65, 45:55, 50:50, 55:45, 70:30, 80:20, 90:10, 100:0) to obtain 12 main fractions (A–L). Fraction B (19.3 mg) was submitted to silica gel column chromatography (10 g), eluting with CHCl_3_:MeOH (90:10) to afford 2 mg of **18** (yield: 0.11%). Fraction D (175.3 mg) was applied to a silica gel column (52 g) using a CHCl_3_:MeOH gradient (95:5, 93:7), to yield **17** (3.5 mg, yield: 0.19%) and **2** (120.5 mg, yield: 6.51%). Fraction E (301.2 mg) was subjected to a silica gel column (52 g) using a CHCl_3_:MeOH gradient (93:7, 92:8, 90:10) to give **3** (18.2 mg, yield: 0.98%) and four fractions (E1–4). Fraction E1 (7.6 mg) was further purified on a silica gel column (10 g) and eluted with *n*-hexane:EtOAc:MeOH (10:10:1) to give 2.8 mg of **14** (yield: 0.15%). To isolate metabolite **9** (8 mg, yield: 0.43%), fraction E2 (27.8 mg) was further purified on a silica gel column (15 g) using *n*-hexane:EtOAc:MeOH (10:10:1). Fraction E3 (50.1 mg) was subjected to a silica gel column (15 g) using CHCl_3_:MeOH (95:5) to afford 15 mg of **13** (yield: 0.81%). Fraction E4 (21.7 mg) was subjected to a silica gel column (15 g), using CHCl_3_:MeOH (95:5) for elution, to give **16** (4 mg, yield: 0.22%). Fraction F (192.9 mg) was further purified on a silica gel column (50 g) using CHCl_3_:MeOH (97:3, 95:5, 93:7) for elution, to give metabolites **10** (6.3 mg, yield: 0.34%), **19** (31.1 mg, yield: 1.68%) and one impure fraction (F1). Fraction F1 was further purified by a silica gel column (10 g) with the solvent system *n*-hexane:EtOAc:MeOH (10:10:1), to provide 3.6 mg of **15** (yield: 0.19%). Fraction G (98.7 mg) was submitted to a silica gel column (53 g) using mixtures of CHCl_3_:MeOH (95:5, 94:6, 93:7) to give **12** (3.5 mg, yield: 0.19%) and two fractions (G1-2). To isolate metabolite **11** (1.4 mg, yield: 0.075%), fraction G1 (23.3 mg) was subjected to a silica gel column (10 g), using *n*-hexane:EtOAc:MeOH (10:10:1). Fraction G2 (9 mg) was further fractionated over a silica gel column (10 g) with the solvent system *n*-hexane:EtOAc:MeOH (10:10:1, 10:10:2), to provide 4.6 mg of **4** (yield: 0.25%). Fraction H (275.1 mg) was submitted to a silica gel column (50 g) and eluted with a CHCl_3_:MeOH gradient (95:5, 93:7, 92:8, 90:10) to afford two fractions (H1–2). Fraction H1 (18.3 mg) was purified by a silica gel column (10 g) and eluted with *n*-hexane:EtOAc:MeOH (10:10:1), to give 5.4 mg of **5** (yield: 0.29%). Fraction H2 (138.2 mg) was applied to VLC packed with reversed-phase silica gel (RP-C18, 30 g), using ACN:H_2_O gradient (25:75, 30:70, 40:60, 50:50), to afford **6** (95.4 mg, yield: 5.16%). Fraction I (86.9 mg) was subjected to a silica gel column (50 g) using CHCl_3_:MeOH solvent system (95:5) to give **7** (16.9 mg, yield: 0.91%) and one impure fraction (I1). Fraction I1 (12.9 mg) was further purified on a silica gel column (10 g) and was eluted with a CHCl_3_:MeOH gradient (99:1, 98:2) to give 1.1 mg of **22** (yield: 0.06%) and fraction I1a. To purify metabolite **8** (2.4 mg, yield: 0.13%), fraction I1a (7.4 mg) was subjected to silica gel column chromatography (10 g) with the solvent system *n*-hexane:EtOAc:MeOH (10:10:1). Fraction J (126.5 mg) was submitted to a silica gel column (50 g), eluted with CHCl_3_:MeOH (95:5) to give fraction J1 (5 mg). Fraction K (56.7 mg) was subjected to silica gel column chromatography (10 g) to yield **20** (16.3 mg, yield: 0.88%) one impure fraction (K1) after elution with DCM:MeOH gradient (96:4, 95:5, 94:6). Fraction K1 (9.6 mg) was chromatographed on a silica gel column (10 g) using *n*-hexane:EtOAc:MeOH (10:10:0.5) to afford fraction K1a (2.1 mg). To isolate metabolite **21** (2.2 mg, yield: 0.12%), fractions K1a and J1 (7.1 mg) were combined and subjected to a preparative thin layer chromatography employed with EtOAc:IPA:H_2_O (100:10:2.5).

### Structural characterization

*Metabolite 1:* ^1^H-NMR (C_5_D_5_N, 400 MHz): see Table 2; ^13^C-NMR (C_5_D_5_N, 100 MHz): see Table 5; HR-ESI–MS (positive ion mode): *m/z* 513.35607 (C_30_H_50_NaO_5_, calcd. 513.35559).

**Table 2:** ^1^H NMR spectroscopic data of compounds **1**–**7** (400 MHz).

Position	1^a^	2^a^	3^a^	4^a^	5^a^	6^a^	7^a^
	δ_H_ (*J* in Hz)	δ_H_ (*J* in Hz)	δ_H_ (*J* in Hz)	δ_H_ (*J* in Hz)	δ_H_ (*J* in Hz)	δ_H_ (*J* in Hz)	δ_H_ (*J* in Hz)
**1**	1.20 m, 1.61 m	1.37 m, 1.65 m	1.40 m, 1.94 m	1.27 m, 1.58 m	1.29 m, 1.95 dt (14.7, 8.5)	1.32 m, 1.64 td (12.5, 4.5)	1.27 m, 1.63 m
**2**	1.92 m, 2.00 m	1.94 m, 2.03 m	2.48 m, 2.60 m	1.88 m, 2.00 m	2.45 m, 2.62 m	1.94 m (2H)	1.98 m (2H)
**3**	3.64 dd (8.6, 2.7)	3.66 dd (11.5, 4.5)		3.60 td (7.9, 1.7)		3.63 dt (10.7, 5.1)	3.64 m
**4**							
**5**	1.70 m	1.74 d (9.0)	2.11 d (9.2)	1.69 d (9.2)	2.13 m	1.72 m	1.70 m
**6**	3.78 t (8.7)	3.89 dp (11.7, 3.9, 3.3)	3.72 m	3.80 q (7.7)	3.68 m	3.76 tdd (9.5, 5.8, 3.0)	3.75 ddd (12.3, 8.9, 3.4)
**7**	1.62 m, 1.79 m	1.67 m, 1.93 m	1.55 m, 1.77 m	1.54 m, 1.74 m	1.54 d (13.3), 1.66 dt (12.0, 3.6)	1.79 td (8.0, 3.5) (2H)	1.66 m (2H)
**8**	1.94 m	2.25 m	2.05 dd (11.5, 4.8)	1.96 d (5.7)	1.85 m	1.86 m	1.76 m
**9**							
**10**							
**11**	1.14 m, 1.97 m	1.72 m, 2.43 dd (15.2, 8.1)	1.46 dd (15.5, 4.3), 2.49 m	1.58 m, 2.49 dd (15.5, 8.6)	1.38 m, 2.56 dd (15.6, 9.0)	1.89 m, 2.37 dd (14.9, 5.8)	1.85 td (8.3, 6.9, 3.5), 2.33 m
**12**	1.72 m, 1.87 m	4.24 t (7.2)	4.18 dd (6.7, 2.5)	4.16 dd (8.8, 4.3)	4.14 dd (9.1, 2.7)	4.27 ddd (9.1, 5.9, 2.6)	4.19 dt (9.5, 4.8)
**13**							
**14**							
**15**	1.79 m, 2.11 m	1.98 m, 2.20 m	1.93 m, 2.11 m	2.10 d (17.6), 2.36 d (17.7)	2.11 d (17.6), 2.38 d (17.9)	1.86 m, 2.15 dd (12.6, 7.9)	2.09 d (17.4), 2.28 d (17.8)
**16**	4.90 dtd (8.0, 5.7, 3.1)	4.87 tt (8.1, 4.4)	4.82 tt (8.3, 4.6)			4.88 tt (8.0, 5.0)	
**17**	2.10 d (5.9)	2.60 d (7.6)	2.49 m	3.06 s	3.02 s	2.93 d (8.0)	3.56 s
**18**	1.70 s	1.84 s	1.80 s	1.47 s	1.49 s	1.71 s	1.37 s
**19**	0.30 d (3.1), 0.58 d (4.1)	0.36 d (4.3), 0.71 d (4.4)	0.45 d (4.3), 0.76 d (4.4)	0.44 d (4.3), 0.67 d (4.4)	0.54 d (3.7), 0.76 d (4.1)	0.48 d (4.2), 0.59 d (4.2)	0.53 d (4.5), 0.58 d (4.2)
**20**							
**21**	1.57 s	2.04 s	2.00 s	1.44 s	1.44 s	1.94 s	1.52 s
**22**	1.24 m, 3.09 td (11.2, 4.3)	2.30 m, 2.66 m	2.22 d (16.4), 2.62 m	2.64 dd (12.8, 5.1), 2.27 dd (14.2, 3.2)	2.27 d (13.3), 2.62 m	2.08 dd (13.3, 3.5), 2.75 td (13.4, 4.1)	2.33 m (2H)
**23**	1.88 m, 2.18 m	1.95 m, 2.32 m	1.89 m, 2.30 d (13.9)	1.82 m, 2.20 d (14.1)	1.84 m, 2.20 dd (13.9, 3.0)	1.89 m, 2.28 dt (13.8, 3.4)	1.81 m, 2.15 m
**24**	3.67 brs	3.74 brs	3.69 m	3.64 brs	3.66 brs	3.69 brs	3.64 brs
**25**							
**26**	1.45 s	1.41 s	1.36 s	1.57 s	1.58 s	1.52 s	1.47 s
**27**	1.29 s	1.62 s	1.56 s	1.30 s	1.31 s	1.35 s	1.30 s
**28**	1.88 s	1.86 s	1.72 s	1.84 s	1.74 s	1.91 s	1.92 s
**29**	1.34 s	1.34 s	1.38 s	1.31 s	1.41 s	1.37 s	1.37 s
**30**	0.97 s	1.04 s	0.87 s	1.00 s	0.92 s	1.26 s	1.32 s

^a^In C_5_D_5_N.

*Metabolite 2:* ^1^H-NMR (C_5_D_5_N, 400 MHz): see Table 2; ^13^C-NMR (C_5_D_5_N, 100 MHz): see Table 5; HR-ESI–MS (positive ion mode): *m/z* 529.35165 (C_30_H_50_NaO_6_, calcd. 529.35031).

*Metabolite 3:* ^1^H-NMR (C_5_D_5_N, 400 MHz): see Table 2; ^13^C-NMR (C_5_D_5_N, 100 MHz): see Table 5; HR-ESI–MS (positive ion mode): *m/z* 527.33685 (C_30_H_48_NaO_6_, calcd. 527.33486).

*Metabolite 4:* ^1^H-NMR (C_5_D_5_N, 400 MHz): see Table 2; ^13^C-NMR (C_5_D_5_N, 100 MHz): see Table 5; HR-ESI–MS (positive ion mode): *m/z* 527.33596 (C_30_H_48_NaO_6_, calcd. 527.33486).

*Metabolite 5:* ^1^H-NMR (C_5_D_5_N, 400 MHz): see Table 2; ^13^C-NMR (C_5_D_5_N, 100 MHz): see Table 5; HR-ESI–MS (positive ion mode): *m/z* 525.31989 (C_30_H_46_NaO_6_, calcd. 525.31921).

*Metabolite 6:* ^1^H-NMR (C_5_D_5_N, 400 MHz): see Table 2; ^13^C-NMR (C_5_D_5_N, 100 MHz): see Table 5; HR-ESI–MS (positive ion mode): *m/z* 529.35093 (C_30_H_50_NaO_6_, calcd. 529.35051).

*Metabolite 7:* ^1^H-NMR (C_5_D_5_N, 400 MHz) see Table 2; ^13^C-NMR (C_5_D_5_N, 100 MHz): see Table 5; HR-ESI–MS (positive ion mode): *m/z* 527.33594 (C_30_H_48_NaO_6_, calcd. 527.33486).

*Metabolite 8:* ^1^H-NMR (C_5_D_5_N, 400 MHz): see Table 3; ^13^C-NMR (C_5_D_5_N, 100 MHz): see Table 5; HR-ESI–MS (negative ion mode): *m/z* 547.3276 (C_31_H_47_O_8_, calcd. 547.32709).

**Table 3:** ^1^H NMR spectroscopic data of compounds **8**–**14** (400 MHz).

Position	8^a^	9^b^	10^b^	11^a^	12^a^	13^c^	14^a^
	δ_H_ (*J* in Hz)	δ_H_ (*J* in Hz)	δ_H_ (*J* in Hz)	δ_H_ (*J* in Hz)	δ_H_ (*J* in Hz)	δ_H_ (*J* in Hz)	δ_H_ (*J* in Hz)
**1**	1.32 m, 2.04 m	1.00 dd (9.0, 3.1), 1.60 m	1.24 m, 2.08 m	0.99 d (13.5), 1.58 m	1.16 m, 1.97 m	1.32 m, 1.82 dt (12.7, 3.5)	2.02 dd (13.2, 5.1), 2.56 m
**2**	2.38 m, 2.75 ddd (13.9, 9.2, 6.4)	1.60 m, 1.81 d (9.4)	2.49 m, 2.60 m	1.87–2.02 (2H)	2.49 m, 2.66 ddd (13.5, 7.9, 5.1)	1.58 dd (12.3, 3.8), 1.68 m	2.55 m, 2.75 dd (13.0, 4.7)
**3**		3.29 m		3.60 td (12.6, 1.8)		3.22 dd (11.6, 4.6)	
**4**							
**5**	2.26 m	1.31 d (9.6)	1.87 d (9.9)	1.64 d (10.0)	2.14 d (9.8)	1.36 d (9.4)	2.30 d (9.9)
**6**	3.65 m	3.56 t (10.8)	3.58 t (10.3)	3.83 t (11.0)	3.75 t (10.2)	3.54 td (9.5, 3.9)	3.81 td (10.3, 2.9)
**7**	1.62 m, 1.73 m	1.47 m (2H)	1.32 m, 1.50 m	1.56 m, 1.70 d (11.5)	1.53 m, 1.67 m	1.36 m, 1.48 m	1.69 m, 1.81 m
**8**	1.73 m	2.00 m	2.04 m	2.12 m	2.09 m	2.05 m	2.16 m
**9**							
**10**							
**11**	1.66 m, 2.31 m	1.97 d (19.6), 2.61 d (19.8)	1.96 d (19.8), 2.64 d (19.9)	2.09 m, 2.83 d (20.1)	2.05 d (20.0), 2.83 d (20.0)	3.87 dd (8.7, 2.5)	4.34 dd (9.4, 3.4)
**12**	4.15 ddd (9.7, 7.3, 2.6)					1.86 m, 2.31 dd (14.3, 8.6)	2.26 dd (10.8, 3.4), 2.67 dd (13.9, 9.4)
**13**							
**14**							
**15**	2.09 d (17.5), 2.27 d (16.2)	1.68 m, 2.12 m	1.68 m, 2.12 m	2.18 d (18.1), 2.52 d (16.2)	2.19 d (16.6), 2.53 d (17.5)	1.49 m, 1.93 m	1.85 m, 2.10 m
**16**		4.54 m	4.56 q (7.6)			4.59 td (7.7, 5.3)	4.91 m
**17**	3.55 s	2.36 d (8.7)	2.37 d (8.7)	3.08 s	3.07 s	1.94 d (7.7)	2.10 d (7.6)
**18**	1.33 s	1.72 s	1.73 s	2.06 s	2.06 s	1.44 s	1.80 s
**19**	0.47 d (4.2), 0.65 d (4.2)	0.49 d (4.6), 0.73 d (4.7)	0.55 d (4.7), 0.89 d (4.8)	0.40 d (4.9), 0.76 d (4.5)	0.45 d (4.5), 0.87 d (4.7)	0.29 d (4.7), 1.10 d (4.7)	0.52 d (4.1), 1.70 d (4.1)
**20**							
**21**	1.49 s	1.62 s	1.62 s	1.88 s	1.88 s	1.47 s	1.57 s
**22**	1.84 m, 2.34 m	1.39 m, 2.07 m	1.41 m, 2.03 m	1.58 m, 2.47 m	1.59 m, 2.47 m	1.15 m, 2.63 dt (13.5, 6.9)	1.22 m, 3.09 td (13.6, 4.5)
**23**	1.85 m, 2.21 m	1.70 m, 2.17 m	1.70 m, 2.17 m	1.83 m, 2.22 m	1.85 m, 2.24 m	1.67 m, 2.14 t (13.5)	1.82 m, 2.15 m
**24**	3.64 brs	3.49 brs	3.50 brs	3.63 brs	3.61 brs	3.45 brs	3.64 brs
**25**							
**26**	1.45 s	1.27 s	1.27 s	1.43 s	1.42 s	1.17 s	1.42 s
**27**	1.28 s	1.18 s	1.18 s	1.26 s	1.26 s	1.23 s	1.26 s
**28**	1.73 s	1.28 s	1.36 s	1.92 s	1.76 s	1.26 s	1.79 s
**29**	1.45 s	0.97 s	1.22 s	1.37 s	1.44 s	0.96 s	1.50 s
**30**	1.27 s	0.62 s	0.65 s	0.76 s	0.79 s	0.84 s	0.93 s

^a^In C_5_D_5_N. ^b^In CDCl_3_. ^c^In CD_3_OD and a drop of C_5_D_5_N.

*Metabolite 9:* ^1^H-NMR (CDCl_3_, 400 MHz): see Table 3; ^13^C-NMR (CDCl_3_, 100 MHz): see Table 5; HR-ESI–MS (positive ion mode): *m/z* 527.33662 (C_30_H_48_NaO_6_, calcd. 527.33486).

*Metabolite 10:* ^1^H-NMR (CDCl_3_, 400 MHz): see Table 3; and ^13^C-NMR (CDCl_3_, 100 MHz): see Table 5; HR-ESI–MS (positive ion mode): *m/z* 525.31890 (C_30_H_46_NaO_6_, calcd. 525.31921).

*Metabolite 11:* ^1^H-NMR (C_5_D_5_N, 400 MHz): see Table 3; ^13^C-NMR (C_5_D_5_N, 100 MHz): see Table 5; HR-ESI–MS (positive ion mode): *m/z* 525.32125 (C_30_H_46_NaO_6_, calcd. 525.31921).

*Metabolite 12:* ^1^H-NMR (C_5_D_5_N, 400 MHz): see Table 3; ^13^C-NMR (C_5_D_5_N, 100 MHz): see Table 5; HR-ESI–MS (positive ion mode): *m/z* 523.30530 (C_30_H_44_NaO_6_, calcd. 523.30356).

*Metabolite 13:* ^1^H-NMR (CD_3_OD and a drop of C_5_D_5_N, 400 MHz): see Table 3; ^13^C-NMR (CD_3_OD and a drop of C_5_D_5_N, 100 MHz): see Table 5; HR-ESI–MS (positive ion mode): *m/z* 529.35090 (C_30_H_50_NaO_6_, calcd. 529.35051).

*Metabolite 14:* ^1^H-NMR (C_5_D_5_N, 400 MHz): see Table 3; ^13^C-NMR (C_5_D_5_N, 100 MHz): see Table 5; HR-ESI–MS (positive ion mode): *m/z* 527.33574 (C_30_H_48_NaO_6_, calcd. 527.33486).

*Metabolite 15:* ^1^H-NMR (C_5_D_5_N, 500 MHz): see Table 4; ^13^C-NMR (C_5_D_5_N, 125 MHz): see Table 5; HR-ESI–MS (positive ion mode): *m/z* 525.3170 (C_30_H_46_NaO_6_, calcd. 525.31921).

**Table 4:** ^1^H NMR spectroscopic data of compounds **15**–**22** (400 MHz and *500 MHz).

Position	15^a^*	16^c^	17^b^	18^a^	19^a^	20^a^	21^a^*	22^a^*
	δ_H_ (*J* in Hz)	δ_H_ (*J* in Hz)	δ_H_ (*J* in Hz)	δ_H_ (*J* in Hz)	δ_H_ (*J* in Hz)	δ_H_ (*J* in Hz)	δ_H_ (*J* in Hz)	δ_H_ (*J* in Hz)
**1**	2.00 m, 2.56 m	1.28 m, 1.96 m	1.24 m, 1.80 m	1.34 m, 1.61 m	1.86 m, 2.91 d (9.1)	1.33 m, 1.58 m	1.39 m, 1.64 m	1.53 m, 1.72 m
**2**	2.59 m, 2.79 m	1.70 m (2H)	2.54 t (6.6) (2H)	1.88 m, 1.97 m	1.85 m, 2.02 m	1.68 m, 1.93 m	1.65 m, 1.83 m	1.68 m, 1.84 m
**3**		3.26 m		3.61 m	3.73 m	3.82 dd (5.6, 1.7)	3.78 m	3.79 m
**4**								
**5**	2.32 m	1.54 d (9.8)	1.86 d (9.9)	1.80 d (9.3)	2.72 d (7.2)	1.47 d (12.0)	1.96 m	1.94 m
**6**	3.86 m	3.42 m	3.65 m	3.74 m	4.14 t (8.4)	4.42 m	5.88 m	6.09 m
**7**	1.70 m (2H)	1.42 m (2H)	1.34 m, 1.52 m	3.71 m	1.70 d (11.8), 2.02 m	4.39 m	5.98 m	6.05 d (10.4)
**8**	2.13 m	1.53 m	2.23 m	2.40 d (6.9)	2.65 d (11.7)			
**9**								
**10**								
**11**	4.34 m	3.35 brs	3.71 brs	1.67 m, 2.43 d (5.6)	3.32 m	1.74 m, 2.14 m	2.65 m, 2.91 m	4.73 m
**12**	2.48 dd (14.6, 3.1), 2.84 m	3.93 brs		4.21 m	1.85 m, 2.78 d (13.9)	1.77 m, 2.16 m	4.45 m	4.36 d (6.7)
**13**								
**14**								
**15**	2.13 m, 2.30 m	1.57 m, 2.02 m	1.68 m, 2.12 m	2.45 m, 2.69 d (8.3)	1.90 d (14.3), 2.09 d (12.7)	2.14 m, 2.94 m	2.20 m (2H)	2.16 dd (12.3, 7.7), 2.25 m
**16**		4.51 q (7.4, 7.0)	4.56 d (7.8)	4.84 m	4.94 t (6.9)	4.97 m	4.89 m	4.96 m
**17**	2.86 m	2.32 d (7.8)	2.33 d (8.7)	2.53 d (7.5)	2.20 d (7.7)	2.10 d (7.6)	2.57 m	2.57 d (7.4)
**18**	1.60 s	1.35 s		1.88 s	1.61 s	1.60 s	1.80 s	1.78 s
**19**	0.58 d (4.3), 1.80 d (4.2)	0.57 d (4.2), 0.83 m	0.51 d (4.8), 1.47 d (5.1)	0.40 d (4.5), 0.92 d (4.4)	3.91 m, 3.97 m	1.91 m, 3.17 m	2.42 m, 2.54 m	2.95 d (11.8), 3.13 m
**20**								
**21**	1.49 s	1.65 s	1.62 s	2.02 s	1.55 s	1.51 s	2.01 s	1.99 s
**22**	1.33 m, 2.31 m	1.88 d (12.9), 2.04 d (7.6)	1.42 m, 2.16 m	2.29 m, 2.63 m	1.22 d (14.0), 3.26 d (13.9)	1.26 m, 3.23 td (13.7, 4.4)	2.38 m, 2.67 m	2.33 m, 2.68 m
**23**	1.86 m, 2.17 m	1.64 m, 2.17 t (13.5)	1.72 m, 2.18 m	1.90 m, 2.29 m	1.83 m, 2.11 m	1.86 m, 2.16 m	1.99 m, 2.32 m	1.98 m, 2.32 m
**24**	3.64 brs	3.45 brs	3.51 brs	3.70 brs	3.65 brs	3.65 brs	3.75 brs	3.78 brs
**25**								
**26**	1.42 s	1.26 s	1.28 s	1.37 s	1.44 s	1.42 s	1.42 s	1.48 s
**27**	1.28 s	1.21 s	1.19 s	1.58 s	1.29 s	1.31 s	1.65 s	1.70 s
**28**	1.83 s	1.31 s	1.38 s	1.86 s	1.77 s	1.66 s	0.96 s	1.16 s
**29**	1.55 s	0.97 s	1.24 s	1.32 s	1.08 s	1.31 s	1.08 s	0.97 s
**30**	1.00 s	1.11 s	0.66 s	1.03 s	0.81 s	0.92 s	0.94 s	0.94 s

^a^In C_5_D_5_N. ^b^In CDCl_3_. ^c^In CD_3_OD and a drop of C_5_D_5_N.

**Table 5:** ^13^C NMR spectroscopic data of compounds **1**–**22** (100 MHz and *125 MHz).

Position	1^a^	2^a^	3^a^	4^a^	5^a^	6^a^	7^a^	8^a^	9^b^	10^b^	11^a^	12^a^	13^c^	14^a^	15^a^*	16^c^	17^b^	18^a^	19^a^	20^a^	21^a^*	22^a^*
	δ_C_	δ_C_	δ_C_	δ_C_	δ_C_	δ_C_	δ_C_	δ_C_	δ_C_	δ_C_	δ_C_	δ_C_	δ_C_	δ_C_	δ_C_	δ_C_	δ_C_	δ_C_	δ_C_	δ_C_	δ_C_	δ_C_
**1**	32.6	32.3	31.8	32.3	31.5	32.9	32.6	32.5	32.8	32.3	33.0	31.9	29.7	29.3	29.9	35.1	29.5†	32.1	29.0	37.1	39.1	38.1
**2**	31.2	31.0	36.0	31.0	35.8	31.3	31.2	36.3	30.0	35.6	30.9	35.7	30.2	36.1	36.8	30.2	36.1	30.9	32.8	24.8	26.1	26.0
**3**	78.1	78.0	216.3	77.9	216.1	78.1	78.0	217.8	77.9	216.1	77.6	215.7	78.5	217.2	218.7	78.0	216.3	77.8	77.2	86.3	85.3	85.6
**4**	42.2	42.1	50.3	42.1	50.2	42.2	42.0	51.3	41.3	50.0	42.1	50.2	41.9	50.5	51.2	41.5	50.2	42.0	42.0	46.9	46.4	45.9
**5**	53.8	53.1	53.2	53.3	53.2	54.2	54.1	54.2	52.9	53.0	53.1	52.7	53.8	54.4	55.0	53.0	53.6	50.8	57.5	55.5	58.1	56.8
**6**	68.3	66.9	68.1	67.2	68.3	69.0	68.8	70.2	69.0	69.2	68.1	68.1	67.7	68.6	69.0	69.5	68.8	73.2	67.5	76.9	132.1	134.1
**7**	38.7	37.2	37.6	37.8	37.8	39.5	39.4	39.4	38.1	37.7	39.0	38.2	37.6	38.6	39.3	38.8	37.9	75.7	37.1	80.2	128.7	128.8
**8**	47.1	44.4	46.2	44.1	45.5	48.3	46.9	48.0	47.3	47.5	46.2	46.2	45.7	47.8	47.7	48.9	47.9†	52.4	40.7	139.5	138.5	141.9
**9**	21.1	21.8	22.1	20.8	21.0	20.9	20.1	21.3	21.0	21.5	20.5	20.9	27.6	27.9	28.5	28.9	26.5	20.9	132.7	126.7	131.8	117.2
**10**	29.6	29.2	28.6	29.5	28.6	28.9	29.8	28.9	30.9	29.9	31.3	29.9	29.9	28.3	29.6	31.9	29.7†	29.1	134.5	88.7	100.1	-d
**11**	26.2	36.4	36.6	36.5	36.5	37.9	36.1	37.3	45.7	45.4	45.4	44.9	65.4	63.8	64.0	81.0	71.3	36.7	39.4	30.8	41.0	70.8
**12**	34.0	71.6	71.6	71.5	71.6	73.0	72.7	73.7	212.0	211.3	210.2	209.6	47.4	49.3	48.3	83.4	211.4	71.4	33.8	32.4	70.1	72.3
**13**	46.6	52.3	52.3	52.4	52.4	51.2	51.7	52.8	61.0	61.1	60.3	60.3	46.8†	46.6	51.2	51.4	59.8	52.5	45.7	49.1	51.4	52.5
**14**	45.7	47.5	47.3	42.8	42.6	46.2	42.0	43.0	47.8†	47.8	43.7	43.5	46.1	46.3	47.1	46.5	46.5	47.9	45.3	44.3	46.5	49.1
**15**	47.8	48.5	48.9	50.9	51.2	50.6	52.7	53.8	48.0†	47.9	51.0	50.9	46.8†	47.9	51.8	49.2	47.9†	50.4	45.3	41.6	45.2	44.6
**16**	73.8	71.3	71.3	215.8	215.6	71.9	216.6	217.5	72.9	72.9	214.4	214.1	74.2	73.7	219.2	71.1	72.8	71.5	73.7	74.1	72.0	72.5
**17**	60.7	62.0	62.1	71.2	71.2	55.0	65.6	66.7	51.5	51.6	60.1	60.1	60.3	60.7	70.9	61.5	52.0	61.8	60.7	59.6	61.0	60.6
**18**	20.7	13.5	13.8	14.0	14.4	20.5	20.1	21.2	13.1	13.1	12.7	12.7	20.1	21.3	21.2†	13.2	13.9	13.4	19.8	19.5	13.9	16.1
**19**	31.0	28.4	30.1	29.9	30.9	31.6	31.0	31.2	32.8	32.0	32.2	31.2	21.6	21.6	22.3	35.6	22.0	29.8	68.5	36.3	39.1	35.9
**20**	78.8	78.5	78.3	76.5	76.4	79.3	76.4	77.5	79.3	79.3	75.8	75.9	79.5	78.8	76.3	78.3	79.2	78.5	78.4	78.1	79.3	79.6
**21**	28.5	27.0	27.0	23.3	23.2	27.2	23.5†	24.5	27.0†	27.0	27.6	27.6	27.8	28.3	25.5	25.8	27.0	26.9	29.2	29.3	27.2	27.3
**22**	26.4	29.5	29.3	31.9	32.0	28.6	32.4	33.5	26.9†	26.9	27.2	27.2	26.3	26.4	30.5	28.8	26.9	29.5	26.4	26.5	30.1	29.3
**23**	23.8	23.7	23.6	23.3	23.3	23.6	23.4†	24.5	23.1	23.1	23.5	23.5	23.1	23.8	24.3	22.3	23.1	23.7	23.7	23.9	24.6	24.4
**24**	68.5	69.1	68.9	68.5	68.4	68.6	68.0	69.0	69.4	69.4	68.5	68.5	68.9	68.4	69.5	68.8	69.3	69.0	68.6	68.6	70.1	70.0
**25**	75.1	75.7	75.6	76.6	76.6	75.6	75.5	76.7	75.3	75.3	74.7	74.7	75.6	75.2	75.3	75.3	75.4	75.7	74.7	74.6	76.8	77.0
**26**	28.4	28.4	28.3	28.0	28.0	28.4	27.8	28.9	27.9	27.9	28.1	28.1	27.6	28.5	29.1	27.5	27.9	28.4	28.6	28.7	29.3	29.0
**27**	27.8	28.2	28.2	28.0	28.0	28.0	27.5	28.5	27.5	27.5	28.0	28.0	27.5	27.8	28.8	27.0	27.5	28.1	27.7	27.6	28.6	28.8
**28**	29.2	28.8	27.7	28.9	28.0	29.5	29.5	29.9	28.2	27.6	29.3	28.1	27.9	28.5	29.1	27.9	27.2	28.8	27.3	24.1	26.6	26.1
**29**	15.9	15.7	20.5	15.7	20.3	16.0	16.1	21.0	15.0	20.4	15.7	20.4	15.1	20.3	21.1†	14.7	20.7	15.4	15.0	25.7	26.0	25.3
**30**	20.2	19.7	20.0	19.0	19.3	21.8	20.5	21.7	20.6	20.7	19.5	19.6	20.5	21.5	21.4	18.4	21.7	19.5	19.3	25.9	25.4	26.7

^a^In C_5_D_5_N. ^b^In CDCl_3_. ^c^In CD_3_OD and a drop of C_5_D_5_N. ^†^Overlapped signals. ^d^Not detected.

*Metabolite 16:* ^1^H-NMR (CD_3_OD and a drop of C_5_D_5_N, 400 MHz): see Table 4; ^13^C-NMR (CD_3_OD and a drop of C_5_D_5_N, 100 MHz): see Table 5; HR-ESI–MS (positive ion mode): *m/z* 545.34597 (C_30_H_50_NaO_7_, calcd. 545.34542).

*Metabolite 17:* ^1^H-NMR (CDCl_3_, 400 MHz): see Table 4; ^13^C-NMR (CDCl_3_, 100 MHz): see Table 5; HR-ESI–MS (positive ion mode): *m/z* 541.31528 (C_30_H_46_NaO_7_, calcd. 541.31412).

*Metabolite 18:* ^1^H-NMR (C_5_D_5_N, 400 MHz): see Table 4; ^13^C-NMR (C_5_D_5_N, 100 MHz): see Table 5; HR-ESI–MS (positive ion mode): *m/z* 545.34775 (C_30_H_50_NaO_7_, calcd. 545.34542).

*Metabolite 19:* ^1^H-NMR (C_5_D_5_N, 400 MHz): see Table 4; ^13^C-NMR (C_5_D_5_N, 100 MHz): see Table 5; HR-ESI–MS (positive ion mode): *m/z* 529.35144 (C_30_H_50_NaO_6_, calcd. 529.35051).

*Metabolite 20:* ^1^H-NMR (C_5_D_5_N, 400 MHz): see Table 4; ^13^C-NMR (C_5_D_5_N, 100 MHz): see Table 5; HR-ESI–MS (positive ion mode): *m/z* 527.33874 (C_30_H_48_NaO_6_, calcd. 527.33486).

*Metabolite 21:* ^1^H-NMR (C_5_D_5_N, 500 MHz): see Table 4; ^13^C-NMR (C_5_D_5_N, 125 MHz): see Table 5; HR-ESI–MS (positive ion mode): *m/z* 509.32640 (C_30_H_46_NaO_5_, calcd. 509.32429).

*Metabolite 22:* ^1^H-NMR (C_5_D_5_N, 500 MHz): see Table 4; ^13^C-NMR (C_5_D_5_N, 125 MHz): see Table 5; HR-ESI–MS (positive ion mode): *m/z* 525.3166 (C_30_H_46_NaO_6_, calcd. 525.31921).

### Biological activities

#### Determination of cell viability

SH-SY5Y cell line was maintained in high-glucose Dulbecco’s modified Eagle medium (DMEM) containing 10% FBS at 37 °C, and 5% CO_2_. The cells were homogenously seeded in 96 well plate (20,000 cells/well) and incubated for 24 h. Following 2 h incubation with compounds or vehicle (DMSO), the cells were treated with 70 µM H_2_O_2_. For the 6-OHDA mediated toxicity experiments, cells were treated with 50 µM 6-OHDA after 8 h of treatment with the compounds. For both experiments, cell viability was determined after 24 h via the MTT (3-[4,5-Dimethylthiazol-2-yl]-2,5-diphenyltetrazolium bromide) assay. Briefly, the cells were incubated with MTT (0.5 mg/ml final concentration) for 4 h. Then, all the media was pulled out, and DMSO was added to wells. Photometric absorbance was measured at a wavelength of 590/690 nm by using Varioscan flash spectrophotometer by Thermo Scientific. The statistical significance of differences between compounds and H_2_O_2_ or 6-OHDA treatments were assessed by one-way ANOVA using GraphPad Prism software.

### Determination of ROS levels

SH-SY5Y cells were seeded onto the 6 well plates and were treated with either compounds or the vesicle for 2 h. Then, cells were treated with 70 µM H_2_O_2_. Following procedures were performed as described previously^34^.

### Determination of mitochondrial membrane potential

SH-SY5Y cells were seeded onto the coverslip. After 2 h pretreatment with 100 nM of each compound, cells were exposed to 70 µM H_2_O_2._ Following 24 h incubation, cells were treated with 100 nM MitoTracker^®^ Red FM (Thermo Fisher Scientific, US) for 30 min at 37 °C. Then cells were washed with PBS. After mounting, cells were immediately observed using a fluorescence microscope (Olympus IX70).

All photographs were taken under the same conditions, and the fluorescence intensity of the mitochondria relative to the cell volume was calculated in 30 cells using the ImageJ software.

## Supplementary Information

Supplementary Information.

## Data Availability

The data that support the findings of this study are available from the corresponding authors (EB and PBK) upon reasonable request.
