# Supplementary material for: Neuroprotective metabolites via fungal biotransformation of a novel sapogenin, cyclocephagenol
Source: Sci Rep. 2022 Nov 2;12:18481. doi: 10.1038/s41598-022-22799-5 (PMC9630500; doi:10.1038/s41598-022-22799-5)
Supplement: Supplementary file 1 — Supplementary Information. [file 41598_2022_22799_MOESM1_ESM.pdf]

SUPPLEMENTARY MATERIAL

Neuroprotective Metabolites via Fungal Biotransformation of a Novel Sapogenin, Cyclocephagenol

Melis KÜÇÜKSOLAK[1], Göklem ÜNER[1], Petek BALLAR KIRMIZIBAYRAK[2*], Erdal BEDİR[1*]

[1]Department of Bioengineering, Faculty of Engineering, İzmir Institute of Technology, 35430, Urla, İzmir, Turkey

[2]Department of Biochemistry, Faculty of Pharmacy, Ege University, 35100, Bornova, İzmir, Turkey

***Corresponding Authors:**

Petek BALLAR KIRMIZIBAYRAK: Ege University, 35100 Bornova/İzmir, Turkey; phone: +90(232) 311 3141; email: petek.ballar@ege.edu.tr

Erdal BEDİR: İzmir Institute of Technology, 35430 Urla/İzmir, Turkey; phone: +90(232) 750 6952; email: erdalbedir@iyte.edu.tr

TABLE OF CONTENTS

Figure S 1 Structure of compound **1** 7
Figure S 2 HR-ESI-MS spectrum of compound **1** 7
Figure S 3 ^{1}H-NMR spectrum of compound **1** (400 MHz, C_5D_5N) 8
Figure S 4 ^{13}C-NMR spectrum of compound **1** (100 MHz, C_5D_5N) 9
Figure S 5 COSY spectrum of compound **1** 10
Figure S 6 HSQC spectrum of compound **1** 11
Figure S 7 HMBC spectrum of compound **1** 12
Figure S 8 Key HMBC correlations of compound **1** (arrows from H to C) 13
Figure S 9 Structure of compound **2** 14
Figure S 10 HR-ESI-MS spectrum of compound **2** 14
Figure S 11 ^{1}H-NMR spectrum of compound **2** (400 MHz, C_5D_5N) 15
Figure S 12 ^{13}C-NMR spectrum of compound **2** (100 MHz, C_5D_5N) 16
Figure S 13 COSY spectrum of compound **2** 17
Figure S 14 HSQC spectrum of compound **2** 18
Figure S 15 HMBC spectrum of compound **2** 19
Figure S 16 NOESY spectrum of compound **2** 20
Figure S 17 Key HMBC correlations of compound **2** (arrows from H to C) 21
Figure S 18 Key NOE correlations of compound **2** 22
Figure S 19 Structure of compound **3** 23
Figure S 20 HR-ESI-MS spectrum of compound **3** 23
Figure S 21 ^{1}H-NMR spectrum of compound **3** (400 MHz, C_5D_5N) 24
Figure S 22 ^{13}C-NMR spectrum of compound **3** (100 MHz, C_5D_5N) 25
Figure S 23 COSY spectrum of compound **3** 26
Figure S 24 HSQC spectrum of compound **3** 27
Figure S 25 HMBC spectrum of compound **3** 28
Figure S 26 NOESY spectrum of compound **3** 29
Figure S 27 Key HMBC correlations of compound **3** (arrows from H to C) 30
Figure S 28 Key NOE correlations of compound **3** 31
Figure S 29 Structure of compound **4** 32
Figure S 30 HR-ESI-MS spectrum of compound **4** 32
Figure S 31 ^{1}H-NMR spectrum of compound **4** (400 MHz, C_5D_5N) 33
Figure S 32 ^{13}C-NMR spectrum of compound **4** (100 MHz, C_5D_5N) 34
Figure S 33 COSY spectrum of compound **4** 35
Figure S 34 HSQC spectrum of compound **4** 36
Figure S 35 HMBC spectrum of compound **4** 37
Figure S 36 NOESY spectrum of compound **4** 38
Figure S 37 Key HMBC correlations of compound **4** (arrows from H to C) 39
Figure S 38 Key NOE correlations of compound **4** 40
Figure S 39 Structure of compound **5** 41
Figure S 40 HR-ESI-MS spectrum of compound **5** 41
Figure S 41 ^{1}H-NMR spectrum of compound **5** (400 MHz, C_5D_5N) 42
Figure S 42 ^{13}C-NMR spectrum of compound **5** (100 MHz, C_5D_5N) 43
Figure S 43 COSY spectrum of compound **5** 44

Figure S 44 HSQC spectrum of compound **5** 45
Figure S 45 HMBC spectrum of compound **5** 46
Figure S 46 NOESY spectrum of compound **5** 47
Figure S 47 Key HMBC correlations of compound **5** (arrows from H to C) 48
Figure S 48 Key NOE correlations of compound **5** 49
Figure S 49 Structure of compound **6** 50
Figure S 50 HR-ESI-MS spectrum of compound **6** 50
Figure S 51 ^{1}H-NMR spectrum of compound **6** (400 MHz, C_5D_5N) 51
Figure S 52 ^{13}C-NMR spectrum of compound **6** (100 MHz, C_5D_5N) 52
Figure S 53 COSY spectrum of compound **6** 53
Figure S 54 HSQC spectrum of compound **6** 54
Figure S 55 HMBC correlations of compound **6** 55
Figure S 56 NOESY spectrum of compound **6** 56
Figure S 57 Key HMBC correlations of compound **6** (arrows from H to C) 57
Figure S 58 Key NOE correlations of compound **6** 58
Figure S 59 Structure of compound **7** 59
Figure S 60 HR-ESI-MS spectrum of compound **7** 59
Figure S 61 ^{1}H-NMR spectrum of compound **7** (400 MHz, C_5D_5N) 60
Figure S 62 ^{13}C-NMR spectrum of compound **7** (100 MHz, C_5D_5N) 61
Figure S 63 COSY spectrum of compound **7** 62
Figure S 64 HSQC spectrum of compound **7** 63
Figure S 65 HMBC spectrum of compound **7** 64
Figure S 66 Key HMBC correlations of compound **7** (arrows from H to C) 65
Figure S 67 Structure of compound **8** 66
Figure S 68 HR-ESI-MS spectrum of compound **8** 66
Figure S 69 ^{1}H-NMR spectrum of compound **8** (400 MHz, C_5D_5N) 67
Figure S 70 ^{13}C-NMR spectrum of compound **8** (100 MHz, C_5D_5N) 68
Figure S 71 COSY spectrum of compound **8** 69
Figure S 72 HSQC spectrum of compound **8** 70
Figure S 73 HMBC spectrum of compound **8** 71
Figure S 74 NOESY spectrum of compound **8** 72
Figure S 75 Key HMBC correlations of compound **8** (arrows from H to C) 73
Figure S 76 Key NOE correlations of compound **8** 74
Figure S 77 Structure of compound **9** 75
Figure S 78 HR-ESI-MS spectrum of compound **9** 75
Figure S 79 ^{1}H-NMR spectrum of compound **9** (400 MHz, $CDCl_3$) 76
Figure S 80 ^{13}C-NMR spectrum of compound **9** (100 MHz, $CDCl_3$) 77
Figure S 81 COSY spectrum of compound **9** 78
Figure S 82 HSQC spectrum of compound **9** 79
Figure S 83 HMBC spectrum of compound **9** 80
Figure S 84 Key HMBC correlations of compound **9** (arrows from H to C) 81
Figure S 85 Structure of compound **10** 82
Figure S 86 HR-ESI-MS spectrum of compound **10** 82
Figure S 87 ^{1}H-NMR spectrum of compound **10** (400 MHz, $CDCl_3$) 83
Figure S 88 ^{13}C-NMR spectrum of compound **10** (100 MHz, $CDCl_3$) 84
Figure S 89 COSY spectrum of compound **10** 85

Figure S 90 HSQC spectrum of compound **10** 86
Figure S 91 HMBC spectrum of compound **10** 87
Figure S 92 Key HMBC correlations of compound **10** (arrows from H to C) 88
Figure S 93 Structure of compound **11** 89
Figure S 94 HR-ESI-MS spectrum of compound **11** 89
Figure S 95 ^{1}H-NMR spectrum of compound **11** (400 MHz, C_5D_5N) 90
Figure S 96 ^{13}C-NMR spectrum of compound **11** (100 MHz, C_5D_5N) 91
Figure S 97 COSY spectrum of compound **11** 92
Figure S 98 HSQC spectrum of compound **11** 93
Figure S 99 HMBC spectrum of compound **11** 94
Figure S 100 Key HMBC correlations of compound **11** (arrows from H to C) 95
Figure S 101 Structure of compound **12** 96
Figure S 102 HR-ESI-MS spectrum of compound **12** 96
Figure S 103 ^{1}H-NMR spectrum of compound **12** (400 MHz, C_5D_5N) 97
Figure S 104 ^{13}C-NMR spectrum of compound **12** (100 MHz, C_5D_5N) 98
Figure S 105 COSY spectrum of compound **12** 99
Figure S 106 HSQC spectrum of compound **12** 100
Figure S 107 HMBC spectrum of compound **12** 101
Figure S 108 Key HMBC correlations of compound **12** (arrows from H to C) 102
Figure S 109 Structure of compound **13** 103
Figure S 110 HR-ESI-MS spectrum of compound **13** 103
Figure S 111 ^{1}H-NMR spectrum of compound **13** (400 MHz, CD_3OD and a drop of C_5D_5N). 104
Figure S 112 ^{13}C-NMR spectrum of compound **13** (100 MHz, CD_3OD and a drop of C_5D_5N) 105
Figure S 113 COSY spectrum of compound **13** 106
Figure S 114 HSQC spectrum of compound **13** 107
Figure S 115 HMBC spectrum of compound **13** 108
Figure S 116 NOESY spectrum of compound **13** 109
Figure S 117 Key HMBC correlations of compound **13** (arrows from H to C) 110
Figure S 118 Key NOE correlation of compound **13** 111
Figure S 119 Structure of compound **14** 112
Figure S 120 HR-ESI-MS spectrum of compound **14** 112
Figure S 121 ^{1}H-NMR spectrum of compound **14** (400 MHz, C_5D_5N) 113
Figure S 122 ^{13}C-NMR spectrum of compound **14** (100 MHz, C_5D_5N) 114
Figure S 123 COSY spectrum of compound **14** 115
Figure S 124 HSQC spectrum of compound **14** 116
Figure S 125 HMBC spectrum of compound **14** 117
Figure S 126 NOESY spectrum of compound **14** 118
Figure S 127 Key HMBC correlations of compound **14** (arrows from H to C) 119
Figure S 128 Key NOE correlation of compound **14** 120
Figure S 129 Structure of compound **15** 121
Figure S 130 HR-ESI-MS spectrum of compound **15** 121
Figure S 131 ^{1}H-NMR spectrum of compound **15** (500 MHz, C_5D_5N) 122
Figure S 132 ^{13}C-NMR spectrum of compound **15** (125 MHz, C_5D_5N) 123
Figure S 133 COSY spectrum of compound **15** 124
Figure S 134 HSQC spectrum of compound **15** 125
Figure S 135 HMBC spectrum of compound **15** 126

Figure S 136 NOESY spectrum of compound **15** 127
Figure S 137 Key HMBC correlations of compound **15** (arrows from H to C) 128
Figure S 138 Key NOE correlation of compound **15** 129
Figure S 139 Structure of compound **16** 130
Figure S 140 HR-ESI-MS spectrum of compound **16** 130
Figure S 141 ^{1}H-NMR spectrum of compound **16** (400 MHz, CD_3OD and a drop of C_5D_5N). 131
Figure S 142 ^{13}C-NMR spectrum of compound **16** (100 MHz, CD_3OD and a drop of C_5D_5N) 132
Figure S 143 COSY spectrum of compound **16** 133
Figure S 144 HSQC spectrum of compound **16** 134
Figure S 145 HMBC spectrum of compound **16** 135
Figure S 146 NOESY spectrum of compound **16** 136
Figure S 147 Key HMBC correlations of compound **16** (arrows from H to C) 137
Figure S 148 Key NOE correlations of compound **16** 138
Figure S 149 Structure of compound **17** 139
Figure S 150 HR-ESI-MS spectrum of compound **17** 139
Figure S 151 ^{1}H-NMR spectrum of compound **17** (400 MHz, $CDCl_3$) 140
Figure S 152 ^{13}C-NMR spectrum of compound **17** (100 MHz, $CDCl_3$) 141
Figure S 153 COSY spectrum of compound **17** 142
Figure S 154 HSQC spectrum of compound **17** 143
Figure S 155 HMBC spectrum of compound **17** 144
Figure S 156 NOESY spectrum of compound **17** 145
Figure S 157 Key HMBC correlations of compound **17** (arrows from H to C) 146
Figure S 158 Key NOE correlation of compound **17** 147
Figure S 159 Structure of compound **18** 148
Figure S 160 HR-ESI-MS spectrum of compound **18** 148
Figure S 161 ^{1}H-NMR spectrum of compound **18** (400 MHz, C_5D_5N) 149
Figure S 162 ^{13}C-NMR spectrum of compound **18** (100 MHz, C_5D_5N) 150
Figure S 163 COSY spectrum of compound **18** 151
Figure S 164 HSQC spectrum of compound **18** 152
Figure S 165 HMBC spectrum of compound **18** 153
Figure S 166 NOESY spectrum of compound **18** 154
Figure S 167 Key HMBC correlations of compound **18** (arrows from H to C) 155
Figure S 168 Key NOE correlations of compound **18** 156
Figure S 169 Structure of compound **19** 157
Figure S 170 HR-ESI-MS spectrum of compound **19** 157
Figure S 171 ^{1}H-NMR spectrum of compound **19** (400 MHz, C_5D_5N) 158
Figure S 172 ^{13}C-NMR spectrum of compound **19** (100 MHz, C_5D_5N) 159
Figure S 173 COSY spectrum of compound **19** 160
Figure S 174 HSQC spectrum of compound **19** 161
Figure S 175 HMBC spectrum of compound **19** 162
Figure S 176 NOESY spectrum of compound **19** 163
Figure S 177 Key HMBC correlations of compound **19** (arrows from H to C) 164
Figure S 178 Key NOE correlations of compound **19** 165
Figure S 179 Structure of compound **20** 166
Figure S 180 HR-ESI-MS spectrum of compound **20** 166
Figure S 181 ^{1}H-NMR spectrum of compound **20** (400 MHz, C_5D_5N) 167

Figure S 182 ^{13}C-NMR spectrum of compound **20** (100 MHz, C_5D_5N) 168
Figure S 183 COSY spectrum of compound **20** 169
Figure S 184 HSQC spectrum of compound **20** 170
Figure S 185 HMBC spectrum of compound **20** 171
Figure S 186 NOESY spectrum of compound **20**.. 172
Figure S 187 Key HMBC correlations of compound **20** (arrows from H to C) 173
Figure S 188 Key NOE correlations of compound **20**.. 174
Figure S 189 Structure of compound **21** 175
Figure S 190 HR-ESI-MS spectrum of compound **21** 175
Figure S 191 ^{1}H-NMR spectrum of compound **21** (500 MHz, C_5D_5N) 176
Figure S 192 ^{13}C-NMR spectrum of compound **21** (125 MHz, C_5D_5N) 177
Figure S 193 COSY spectrum of compound **21** 178
Figure S 194 HSQC spectrum of compound **21** 179
Figure S 195 HSQC-TOCSY spectrum of compound **21**.. 180
Figure S 196 HMBC spectrum of compound **21** 181
Figure S 197 NOESY spectrum of compound **21**.. 182
Figure S 198 Key HMBC correlations of compound **21** (arrows from H to C) 183
Figure S 199 Key NOE correlation of compound **21**.. 184
Figure S 200 Structure of compound **22** 185
Figure S 201 HR-ESI-MS spectrum of compound **22** 185
Figure S 202 ^{1}H-NMR spectrum of compound **22** (500 MHz, C_5D_5N) 186
Figure S 203 ^{13}C-NMR spectrum of compound **22** (125 MHz, C_5D_5N) 187
Figure S 204 COSY spectrum of compound **22** 188
Figure S 205 HSQC spectrum of compound **22** 189
Figure S 206 HSQC-TOCSY spectrum of compound **22**.. 190
Figure S 207 HMBC spectrum of compound **22** 191
Figure S 208 NOESY spectrum of compound **22**.. 192
Figure S 209 Key HMBC correlations of compound **22** (arrows from H to C) 193
Figure S 210 Key NOE correlations of compound **22**.. 194
Figure S 211 Thin layer chromatogram of EtOAc extracts for different biotransformation time points (Day 4, 6, 8, 11 and 13) [(Silica gel, Mobile phase: 87.5:12.5 ($CHCl_3$:MeOH)] 195
Figure S 212 Thin layer chromatogram of the isolated metabolites and the EtOAc extract [Silica gel, Mobile phase: 87.5:12.5 ($CHCl_3$:MeOH)].. 196
Figure S 213 Neuroprotective activity of selected compounds against 6-OHDA toxicity Error bars are the standard deviations (n = 3). p-Values were calculated with respect to 6-OHDA-treated cells (*p < 0.05, **p < 0.01, ***p < 0.001, ****p < 0.0001).. 197

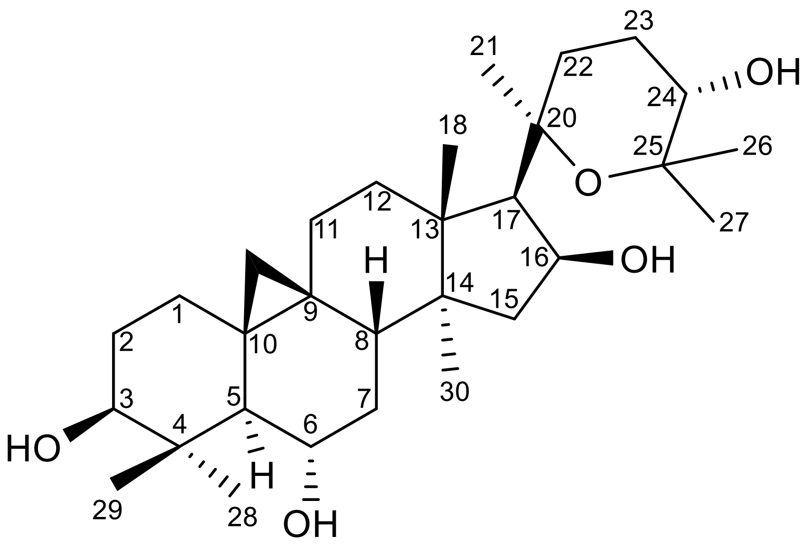

Figure S 1 Structure of compound **1**

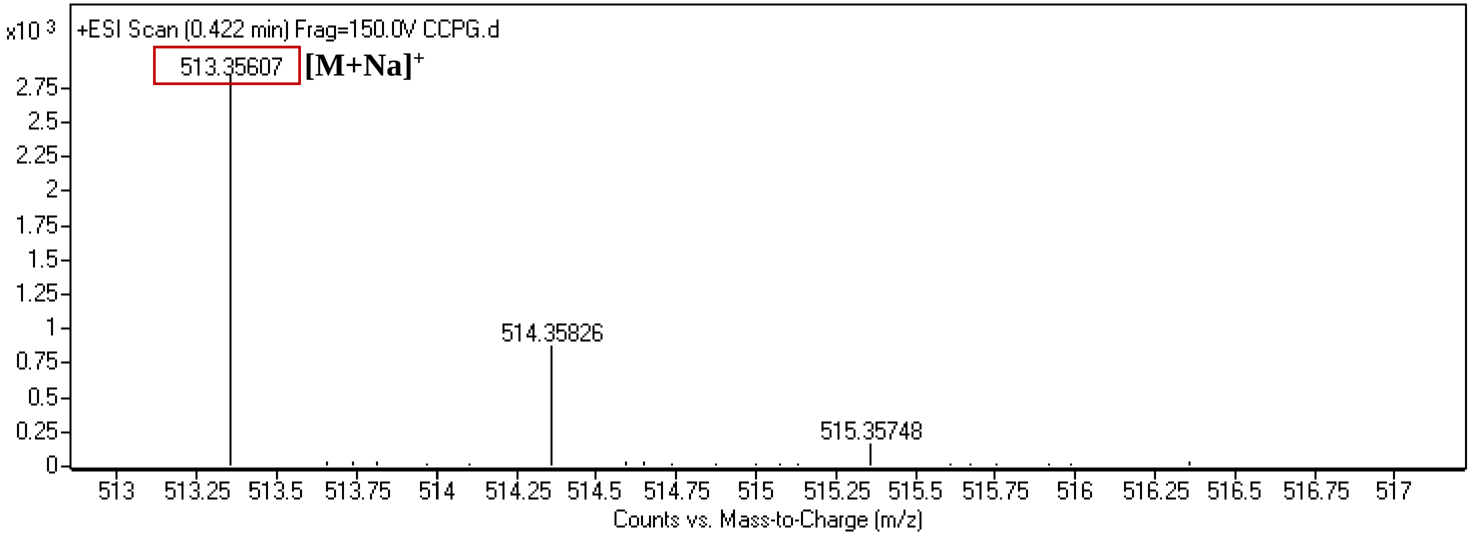

Figure S 2 HR-ESI-MS spectrum of compound **1**

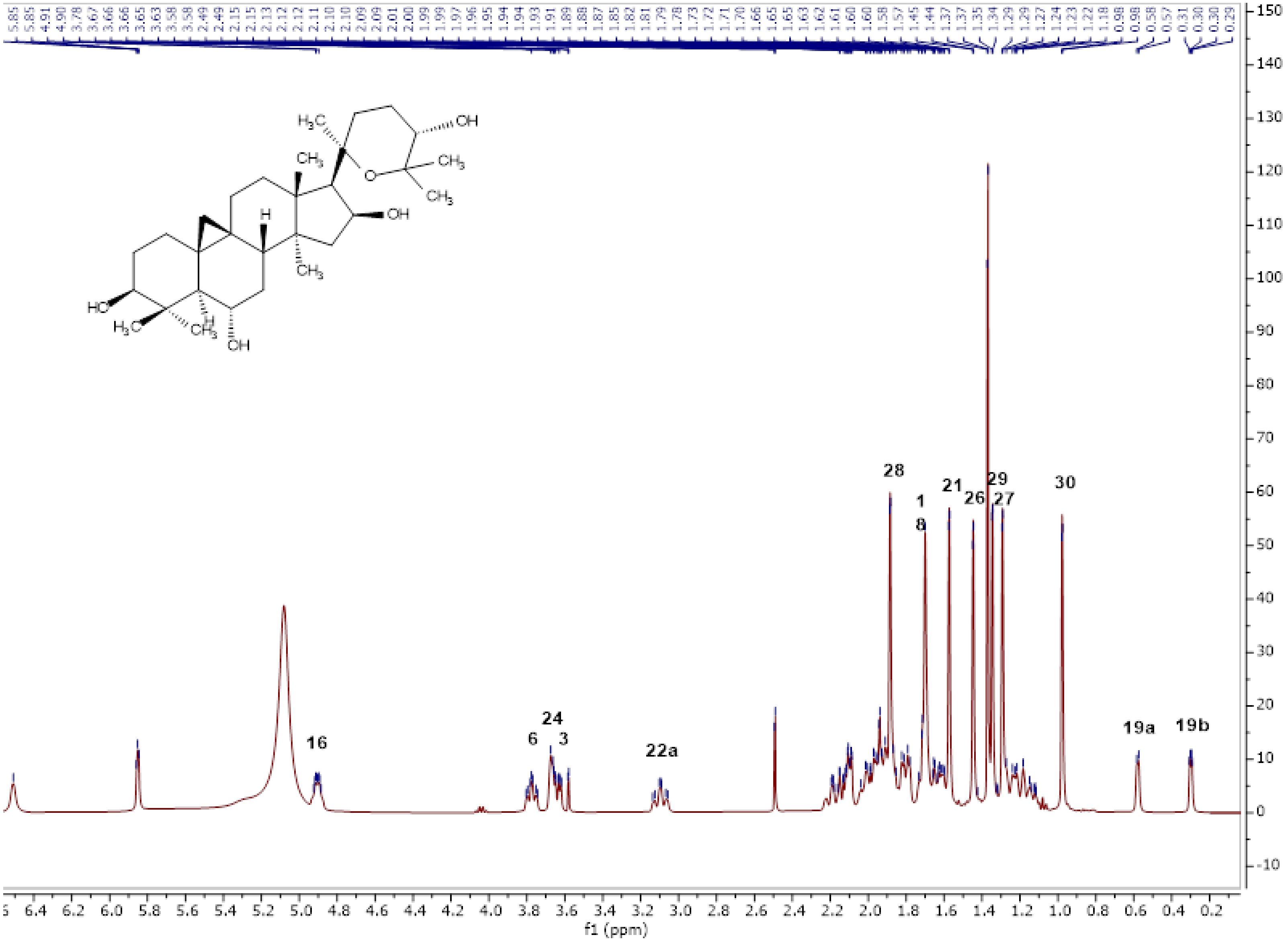

Figure S 3 ^{1}H-NMR spectrum of compound **1** (400 MHz, C_5D_5N)

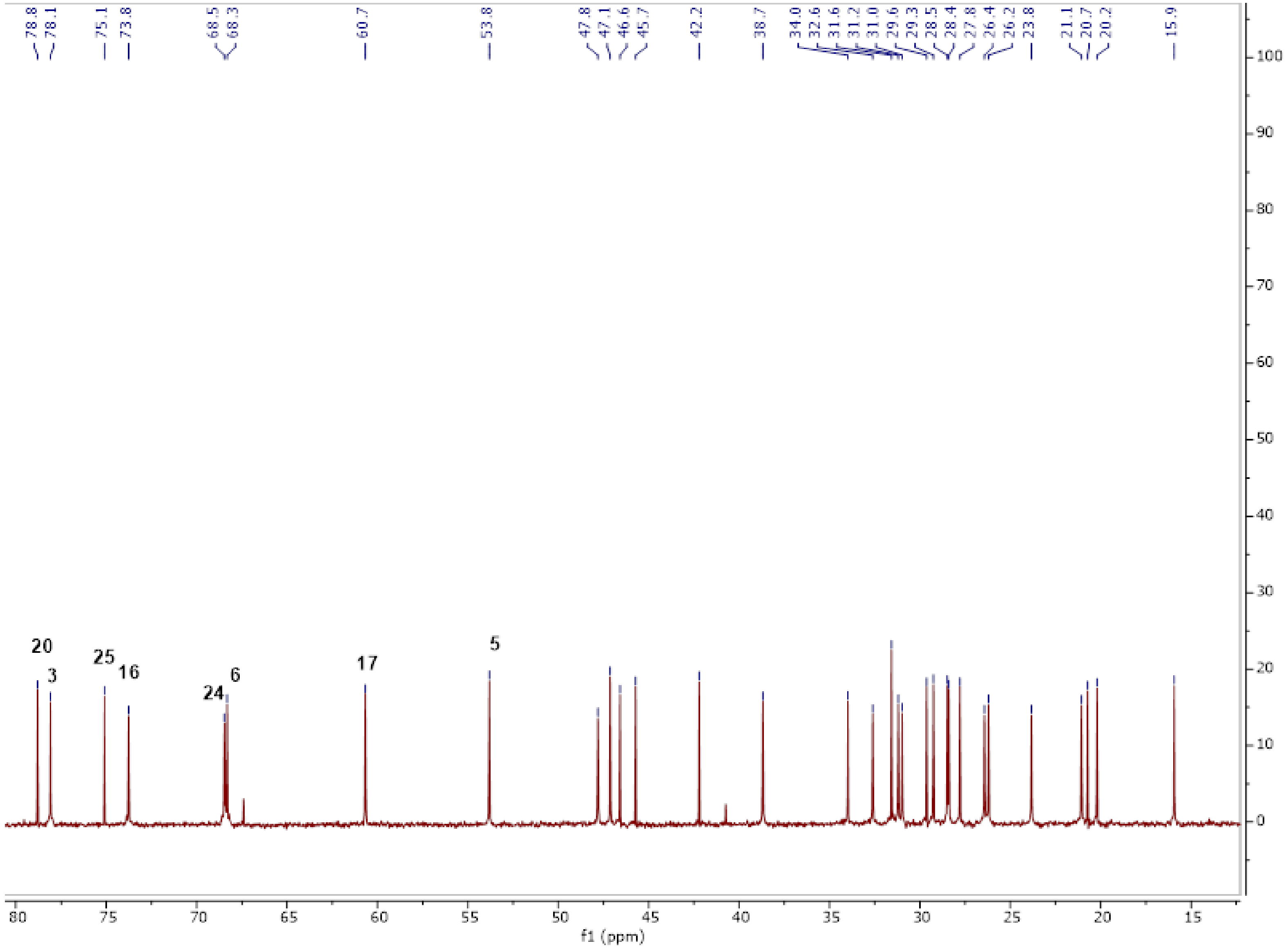

Figure S 4 ^{13}C-NMR spectrum of compound **1** (100 MHz, C_5D_5N)

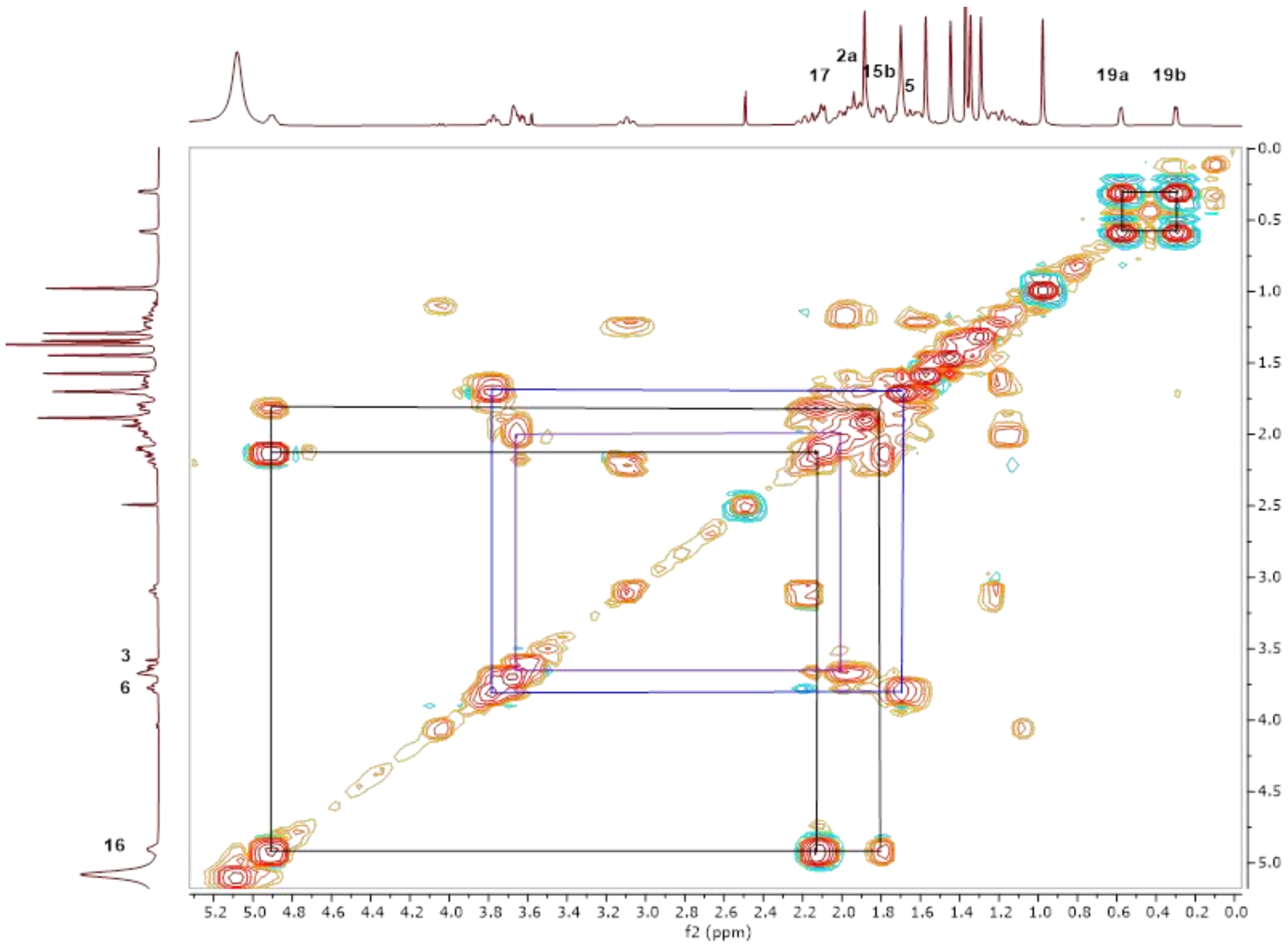

Figure S 5 COSY spectrum of compound **1**

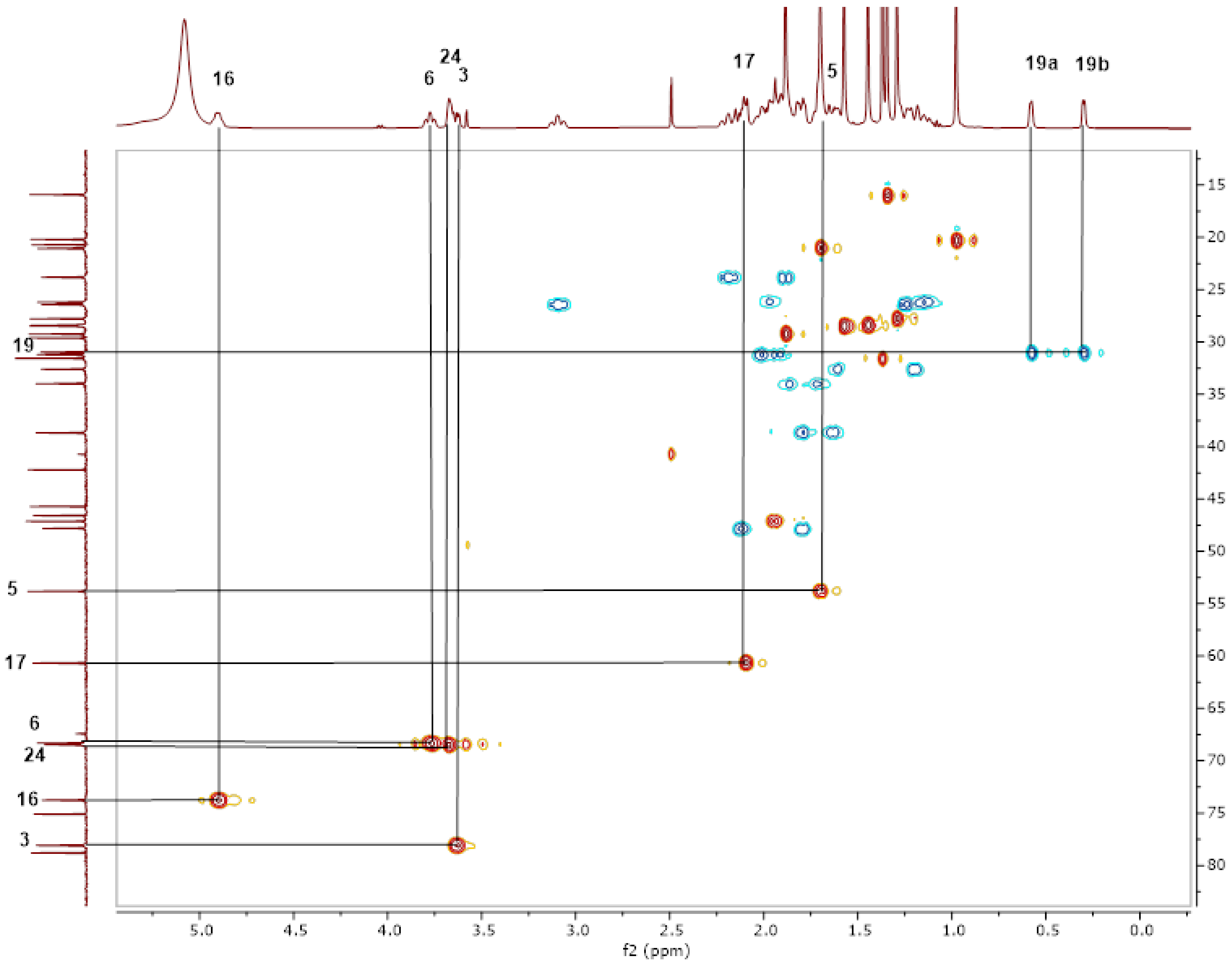

Figure S 6 HSQC spectrum of compound **1**

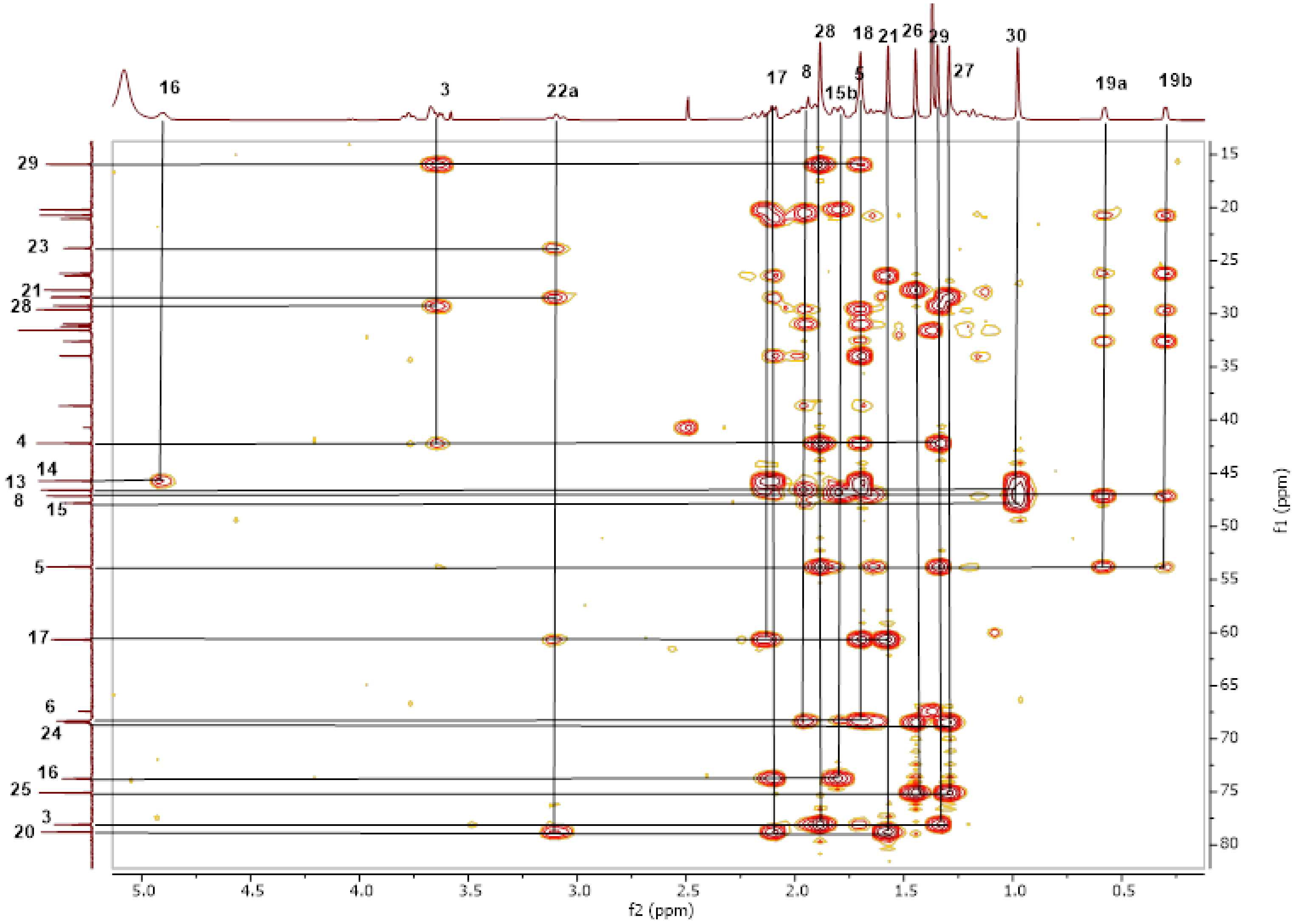

Figure S 7 HMBC spectrum of compound **1**

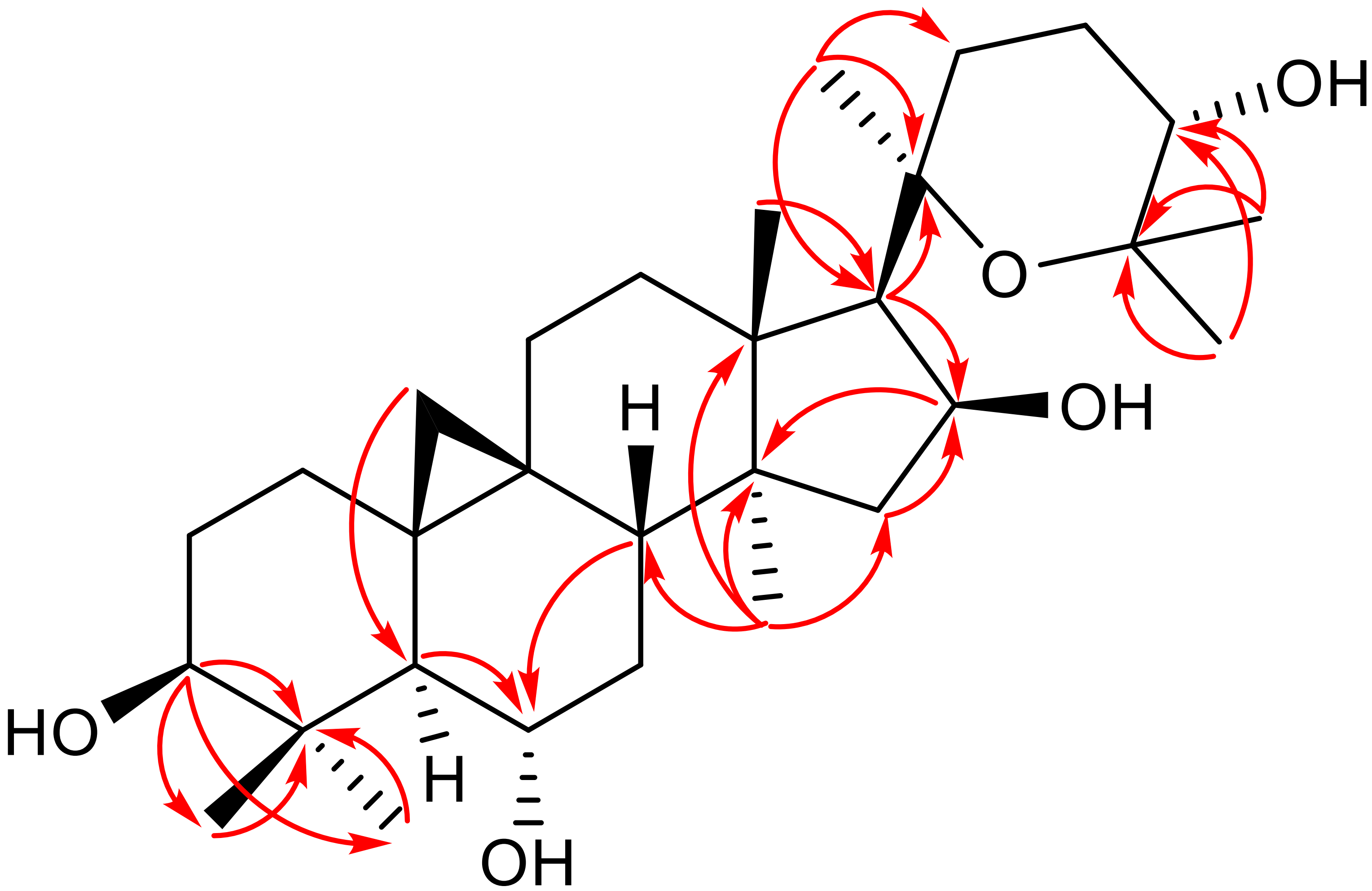

Figure S 8 Key HMBC correlations of compound **1** (arrows from H to C)

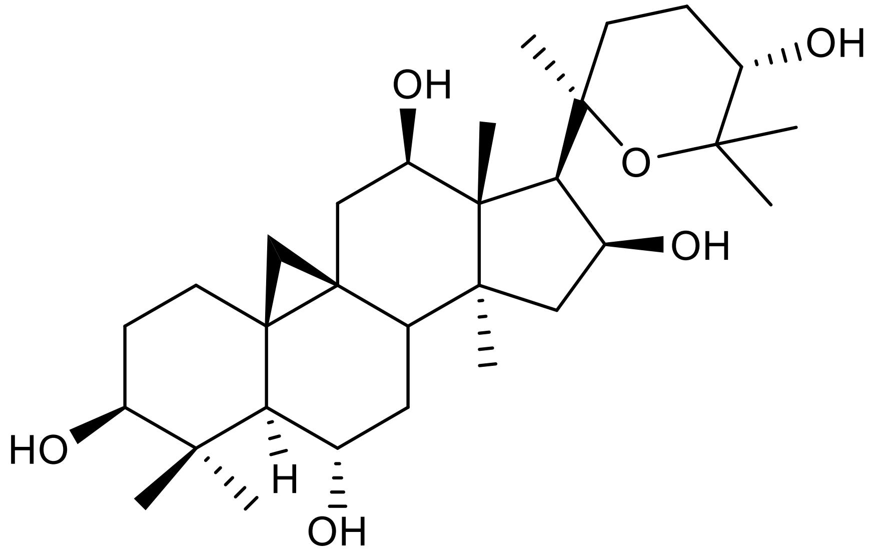

Figure S 9 Structure of compound **2**

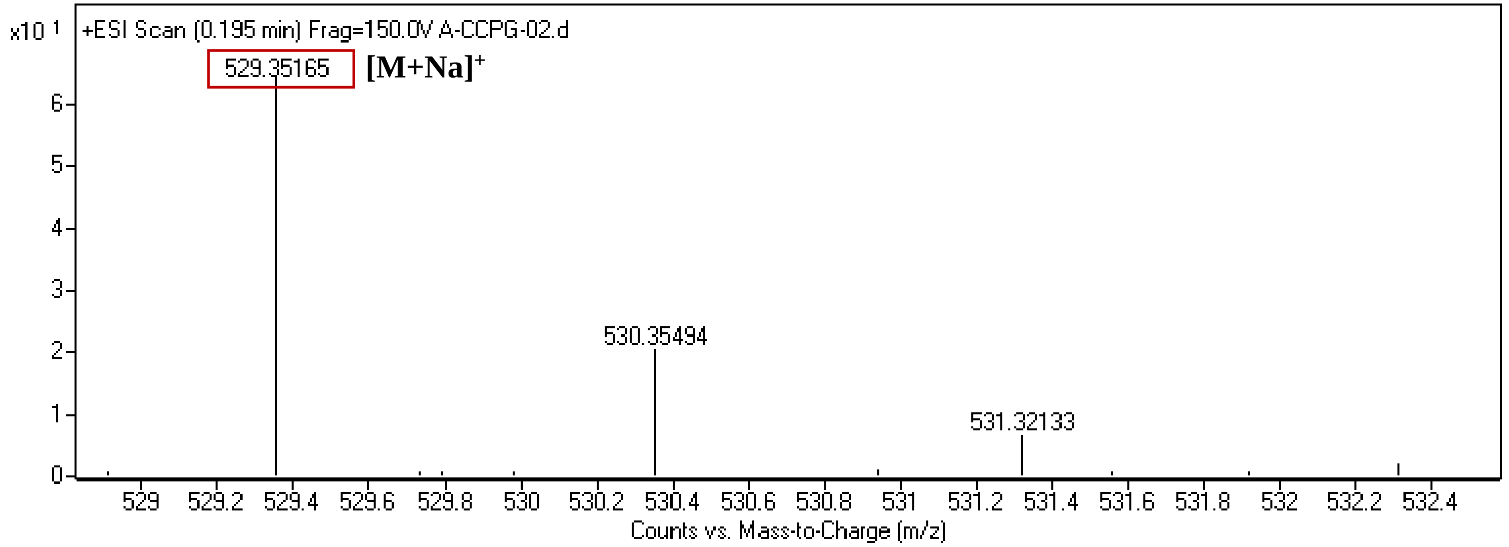

Figure S 10 HR-ESI-MS spectrum of compound **2**

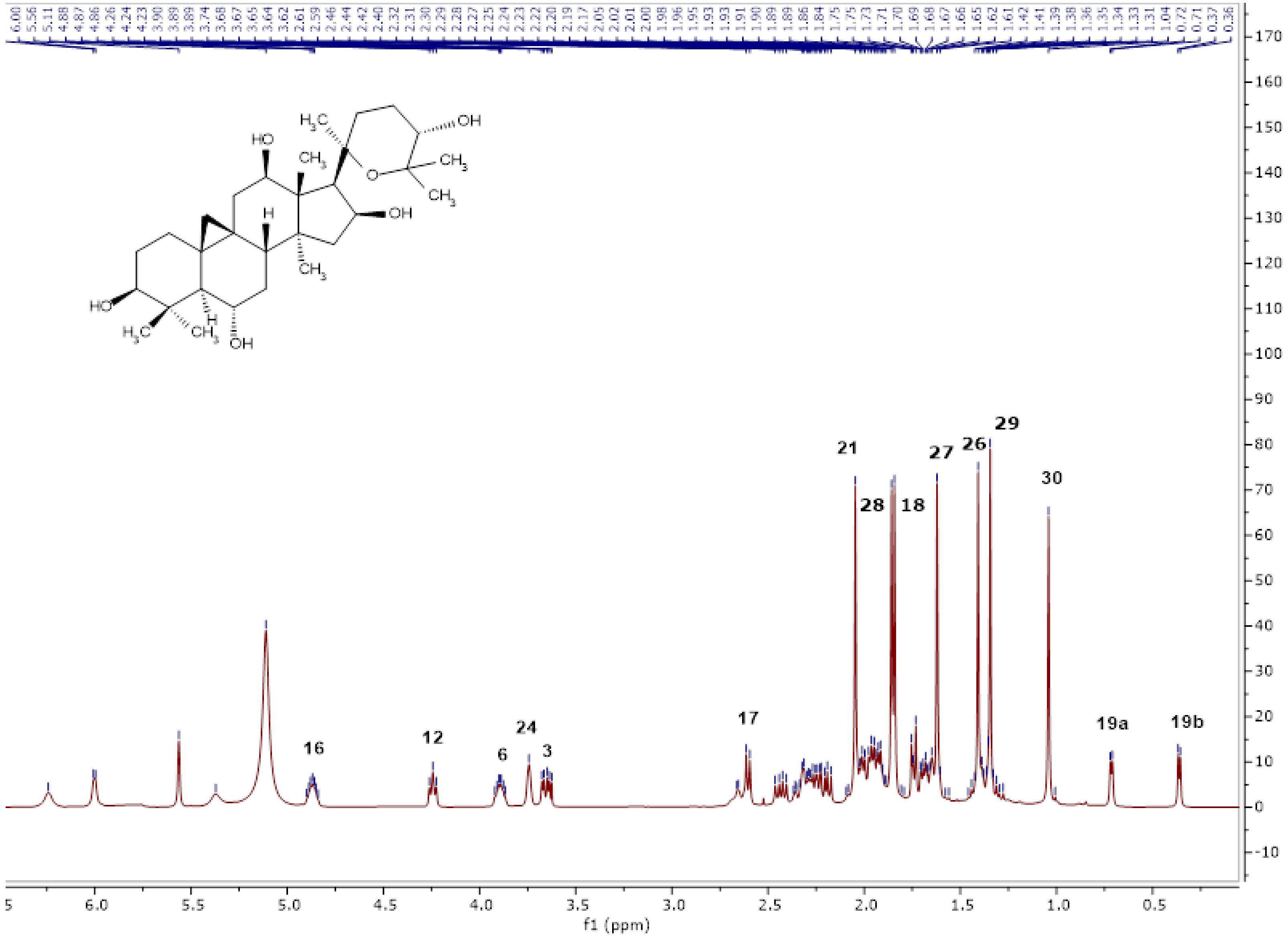

Figure S 11 ^{1}H-NMR spectrum of compound **2** (400 MHz, C_5D_5N)

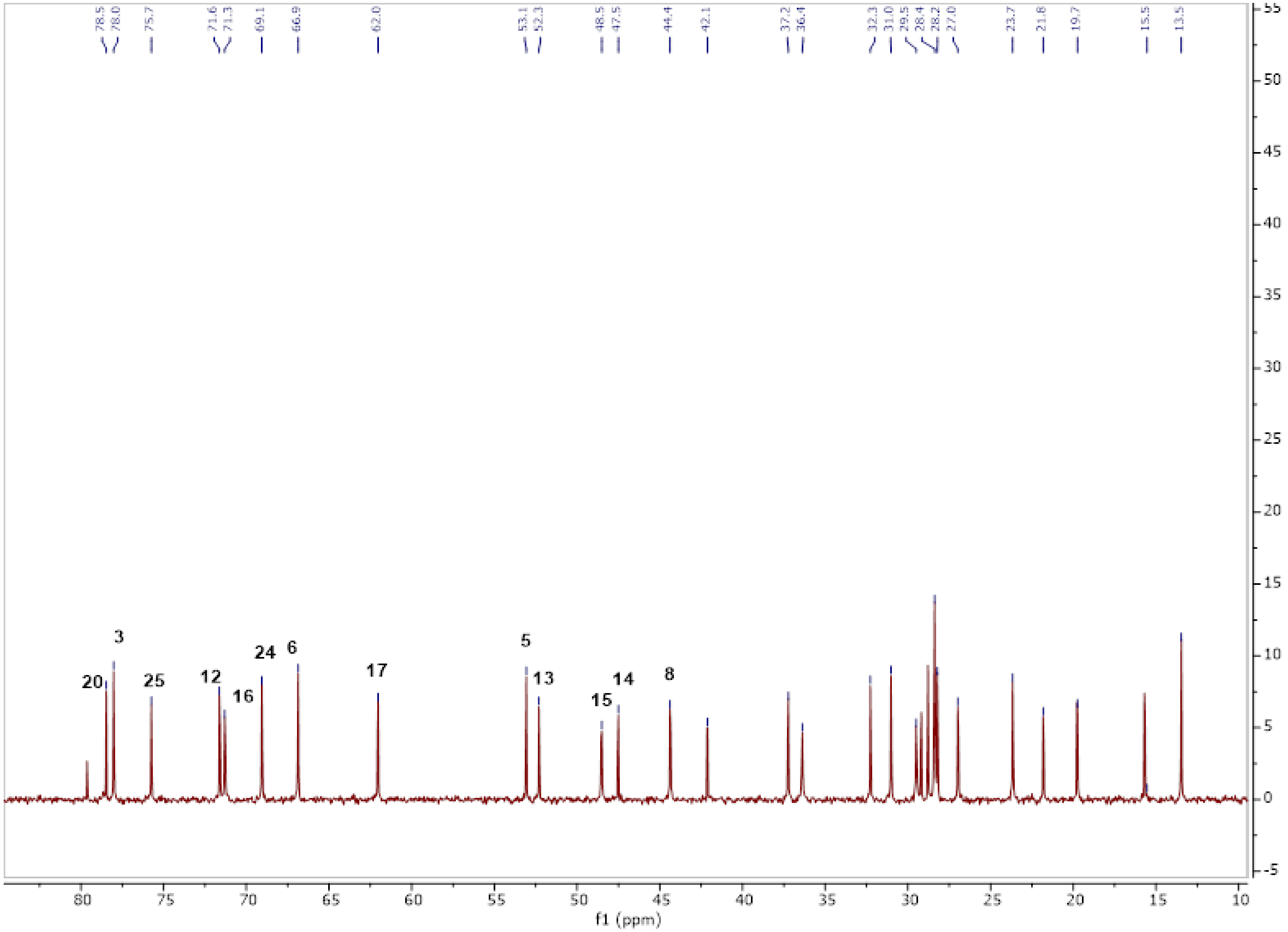

Figure S 12 ^{13}C-NMR spectrum of compound **2** (100 MHz, C_5D_5N)

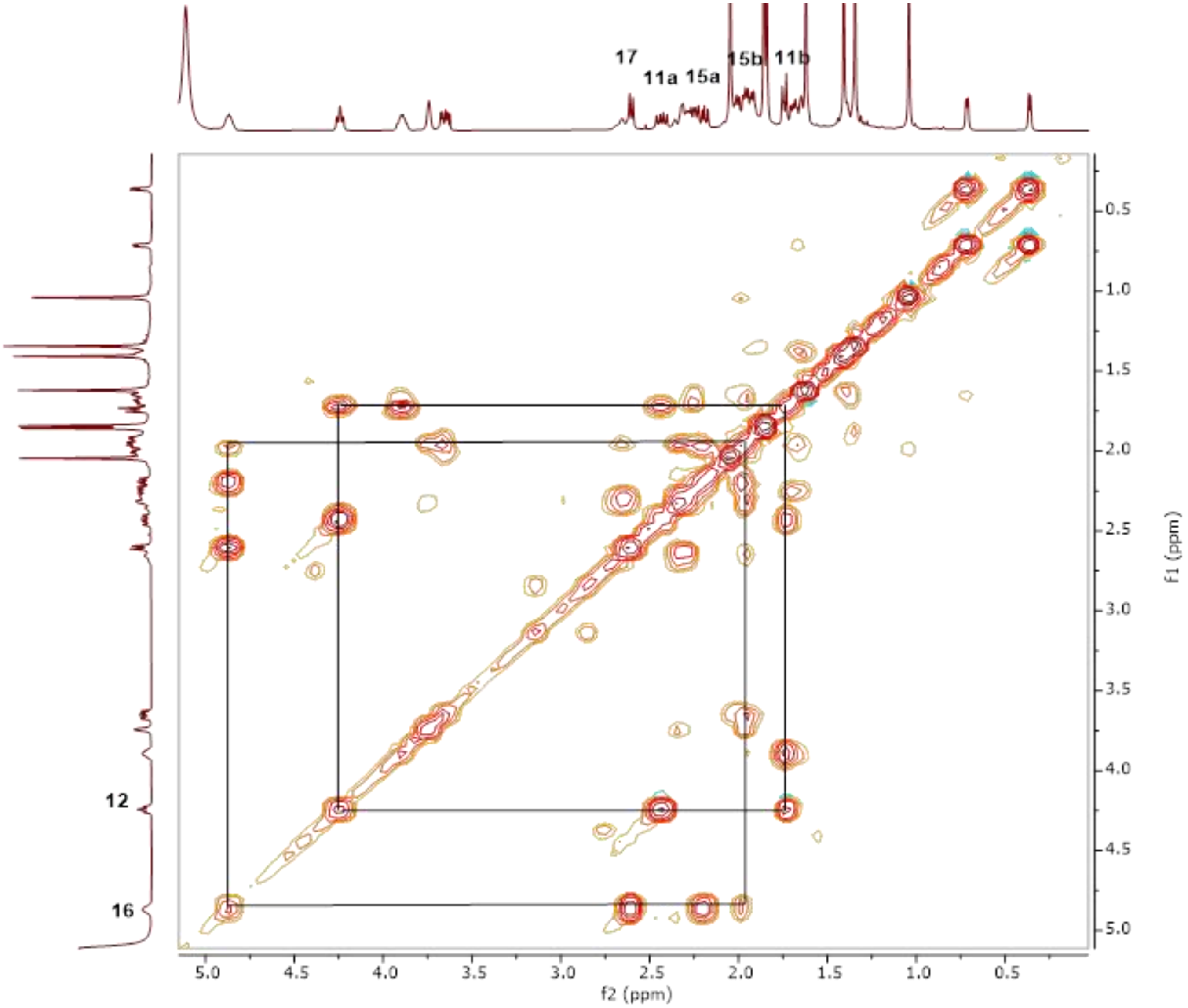

Figure S 13 COSY spectrum of compound **2**

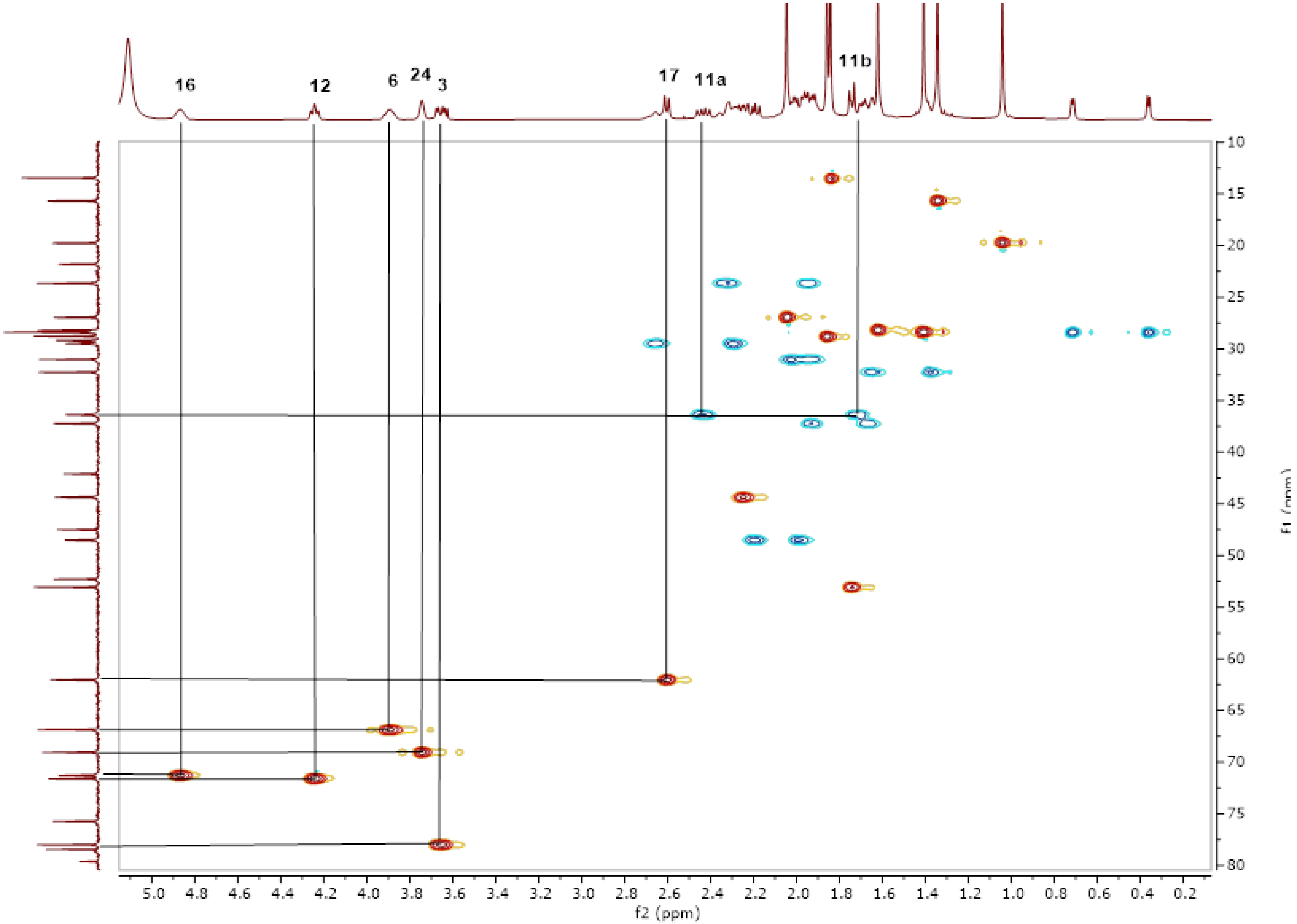

Figure S 14 HSQC spectrum of compound **2**

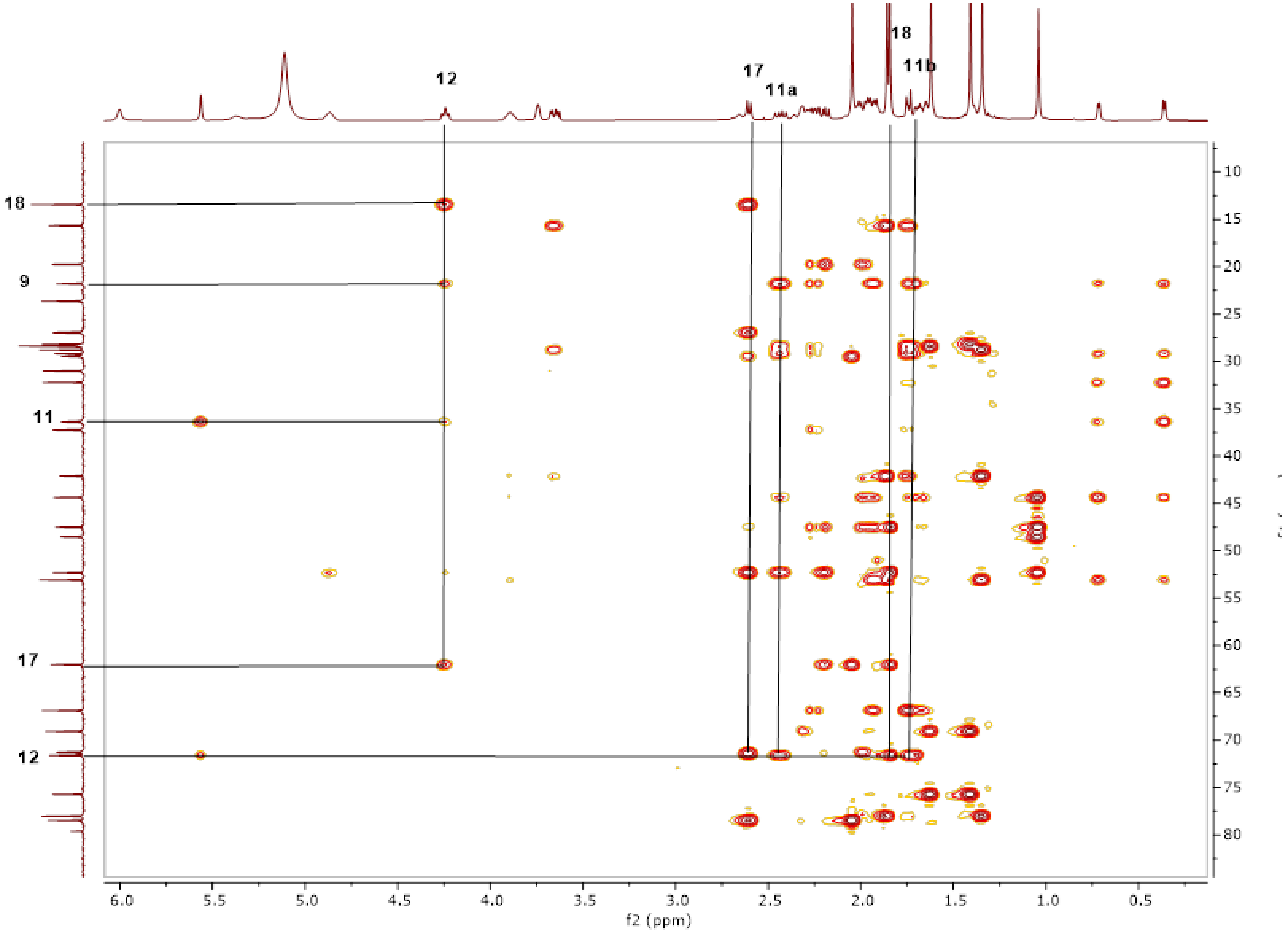

Figure S 15 HMBC spectrum of compound **2**

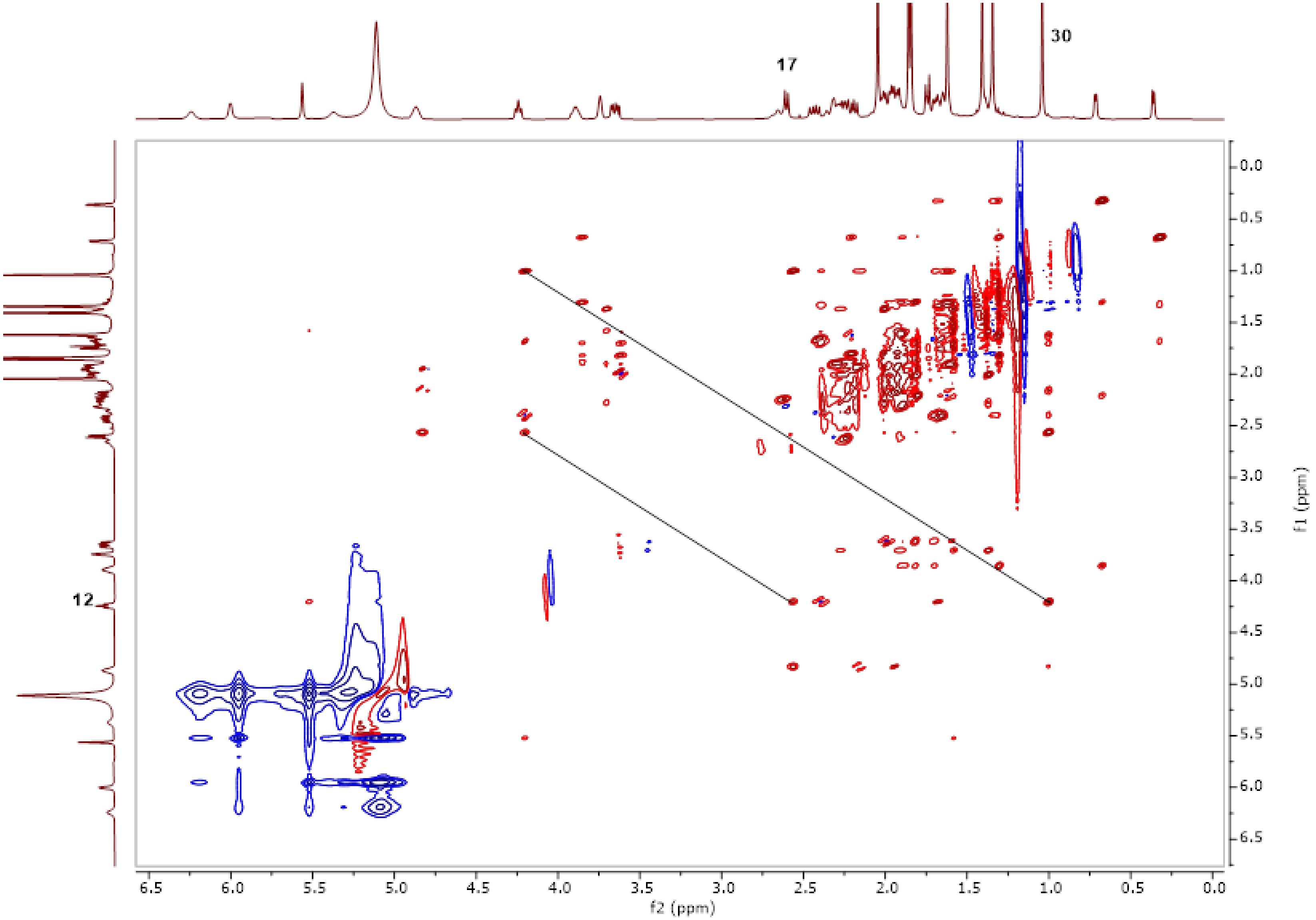

Figure S 16 NOESY spectrum of compound **2**

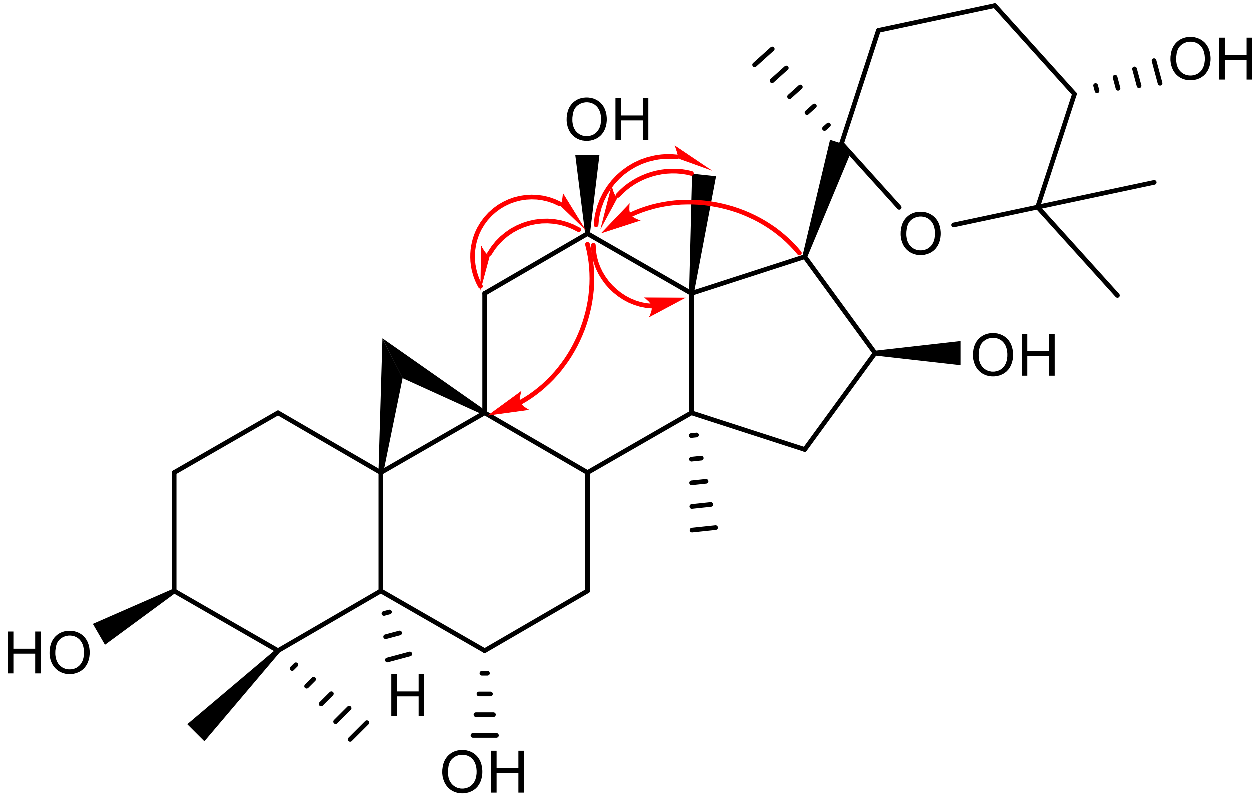

Figure S 17 Key HMBC correlations of compound **2** (arrows from H to C)

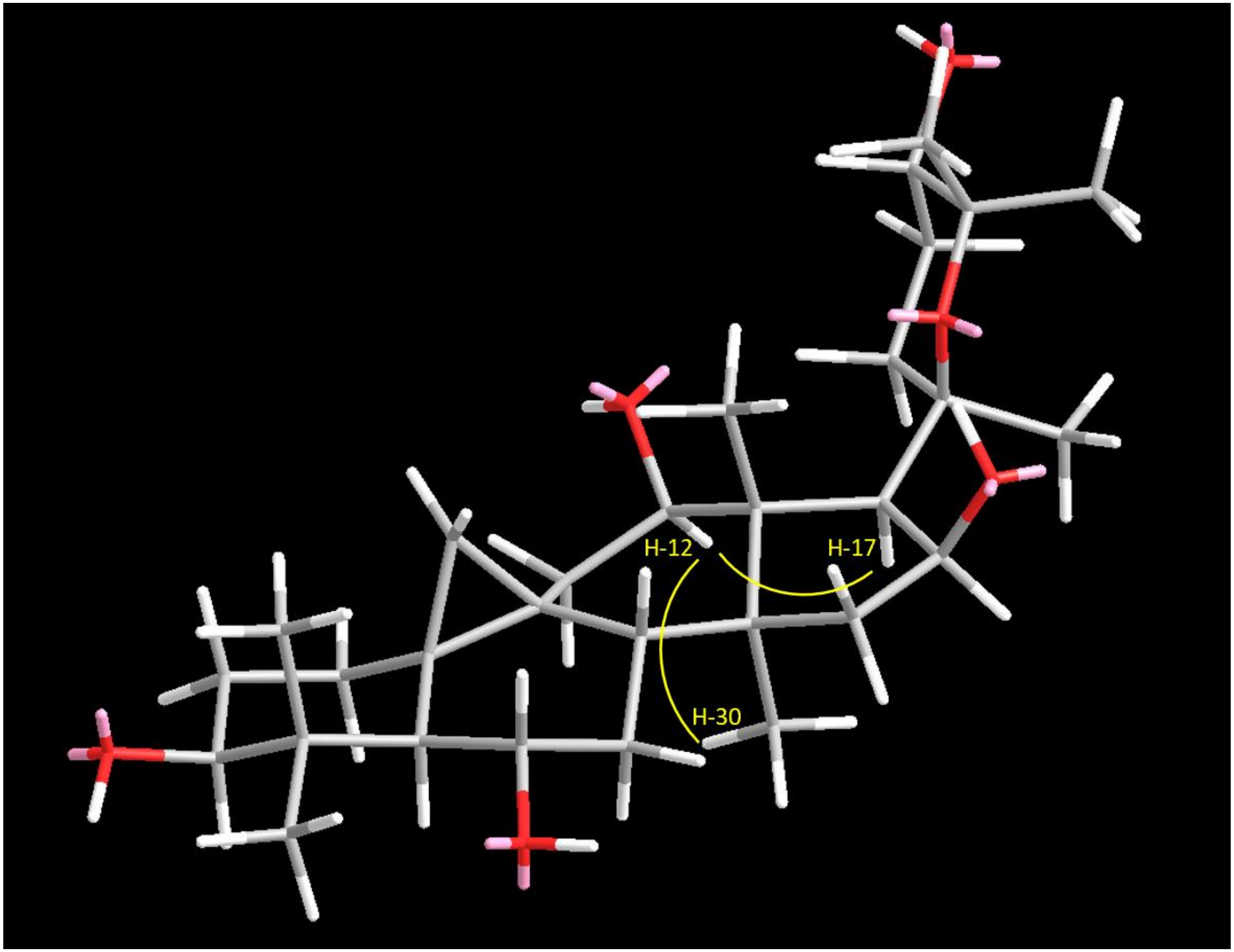

Figure S 18 Key NOE correlations of compound **2**

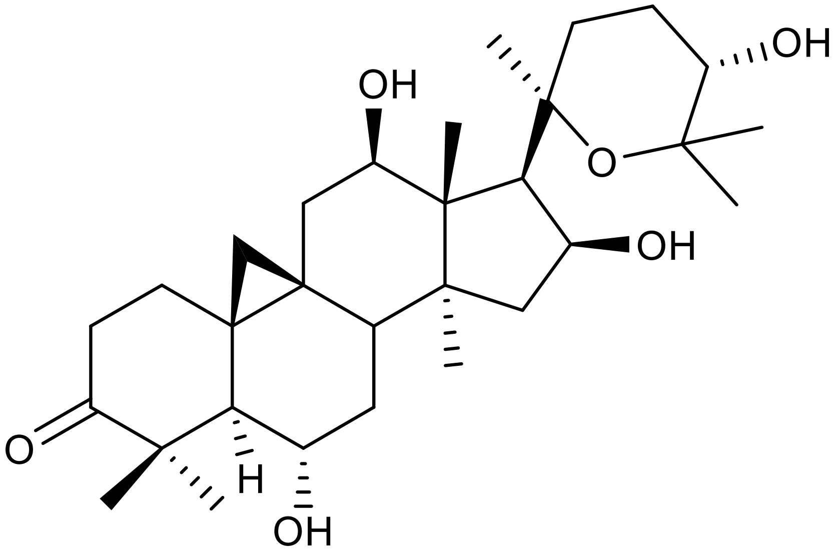

Figure S 19 Structure of compound **3**

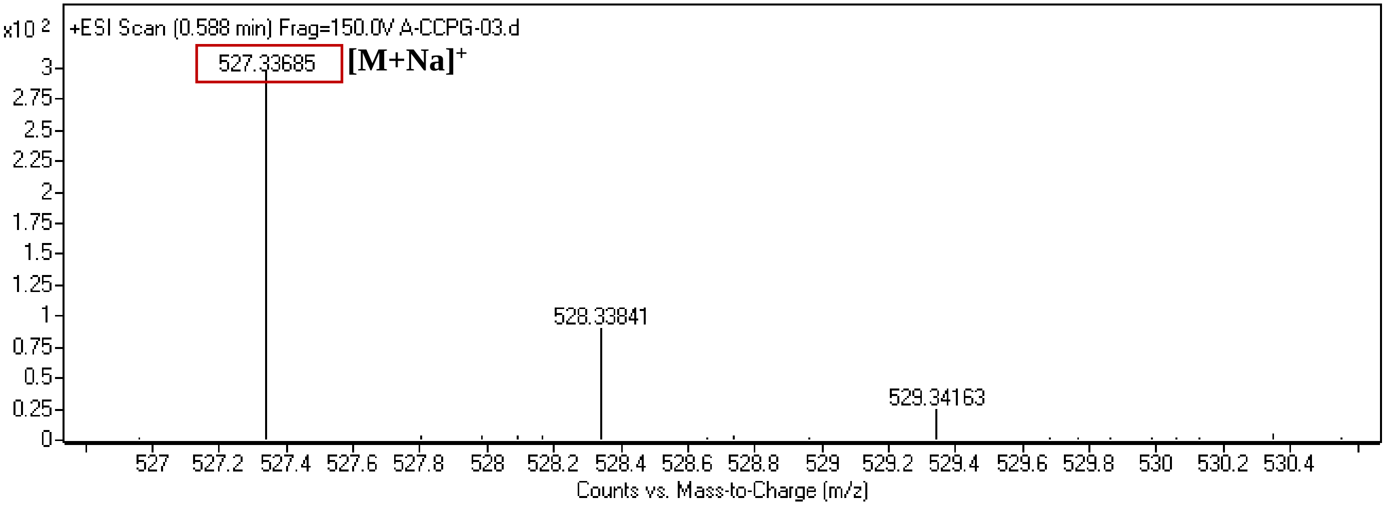

Figure S 20 HR-ESI-MS spectrum of compound **3**

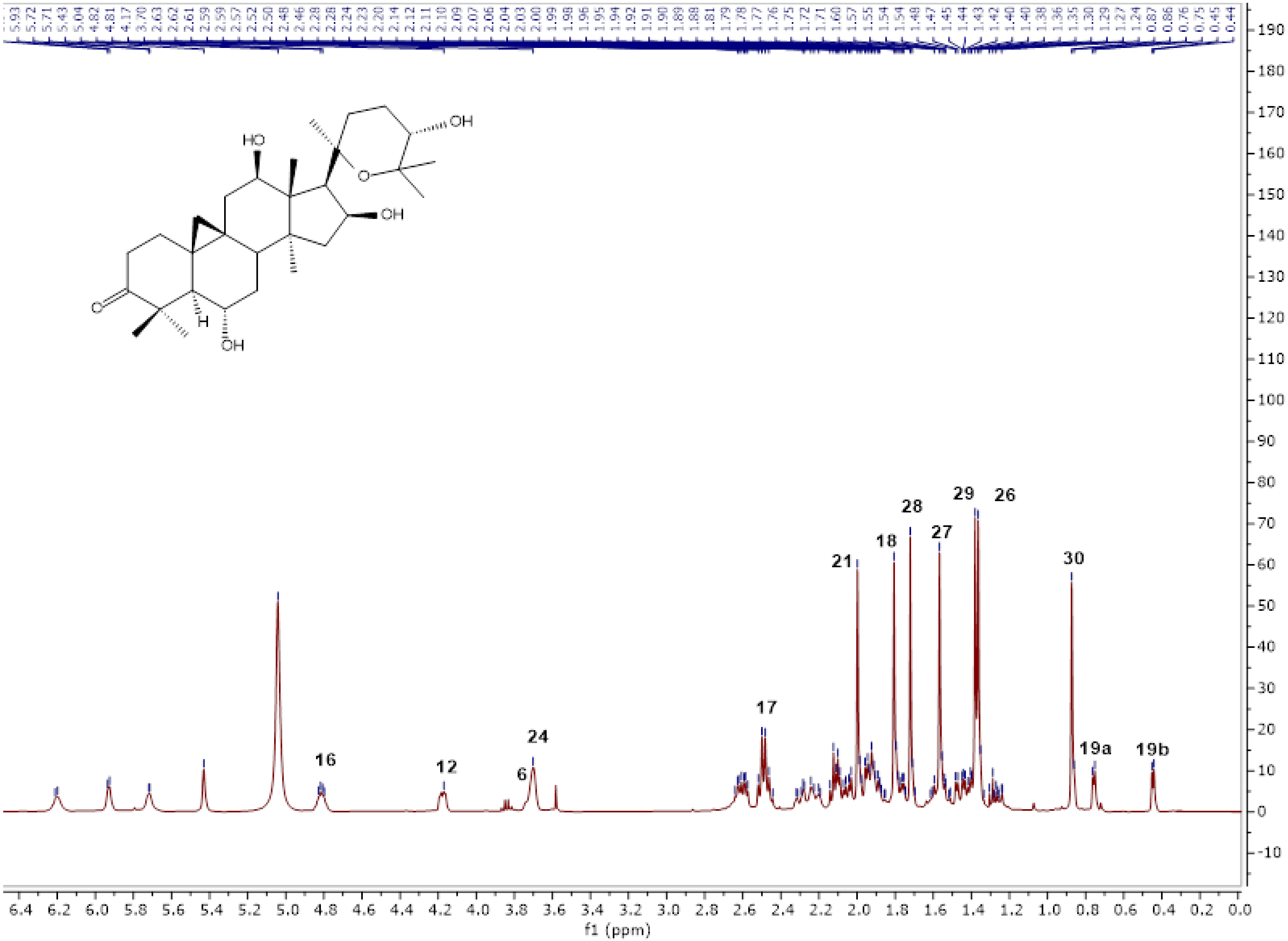

Figure S 21 ^{1}H-NMR spectrum of compound **3** (400 MHz, C_5D_5N)

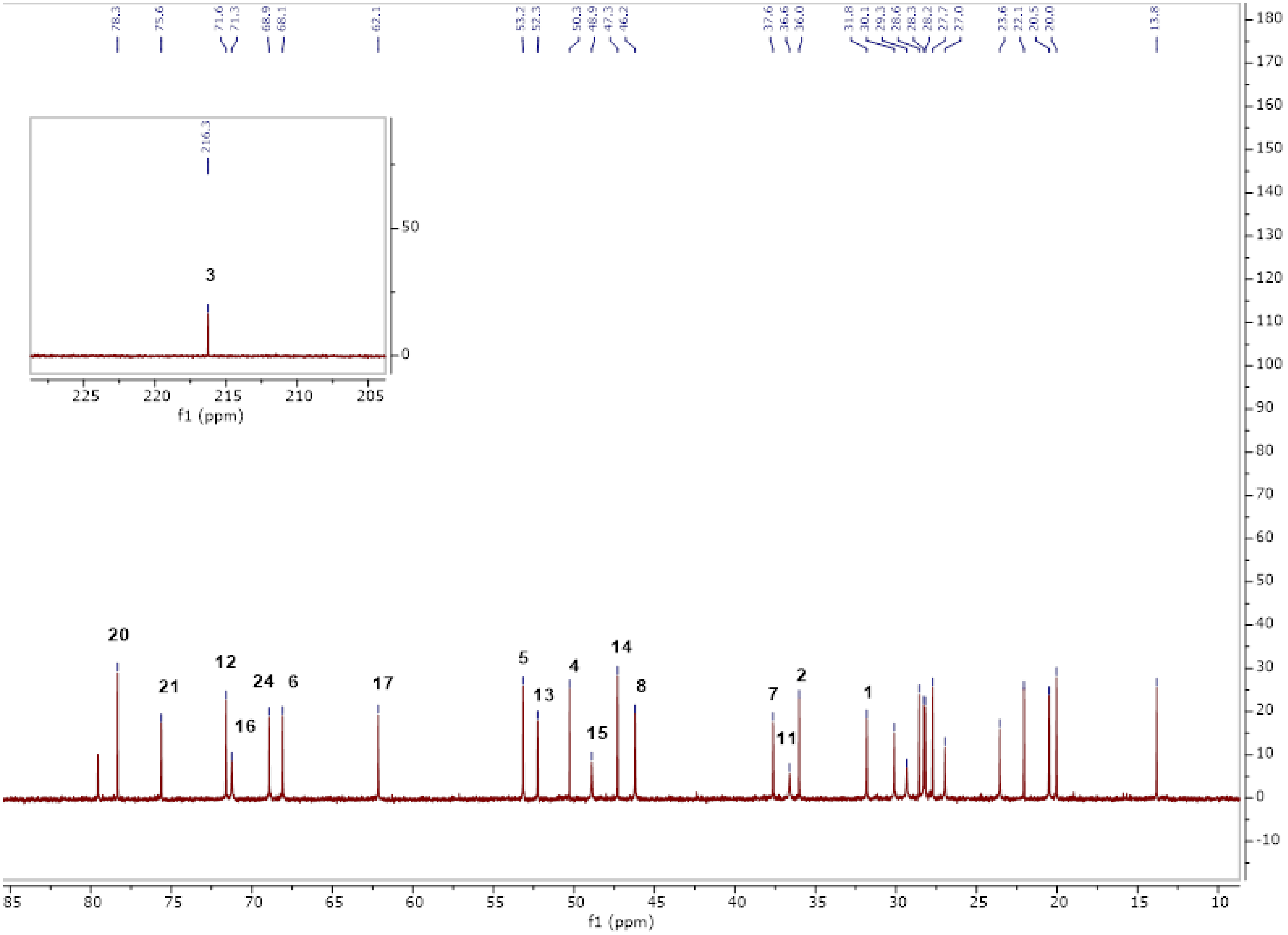

Figure S 22 ^{13}C-NMR spectrum of compound **3** (100 MHz, C_5D_5N)

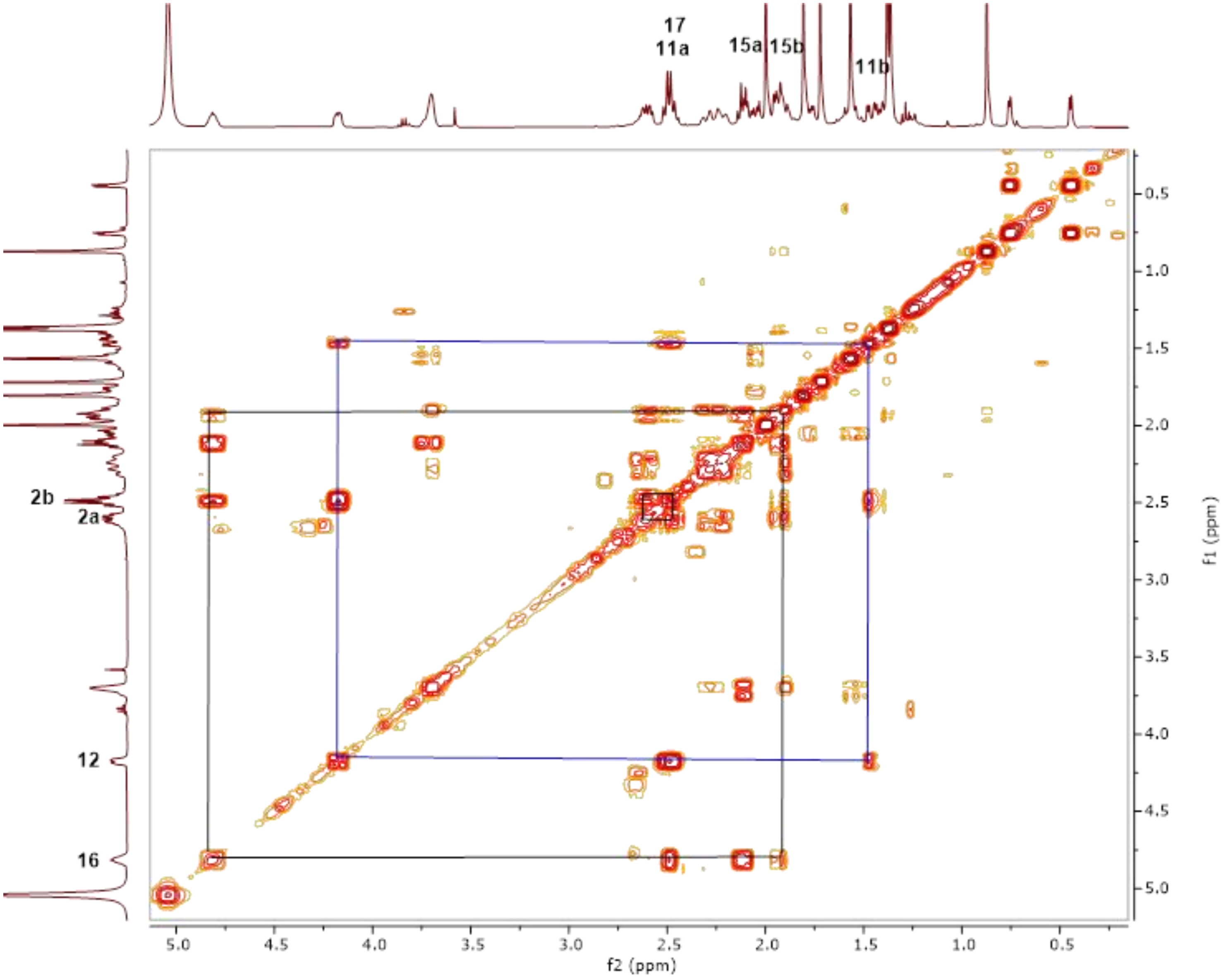

Figure S 23 COSY spectrum of compound **3**

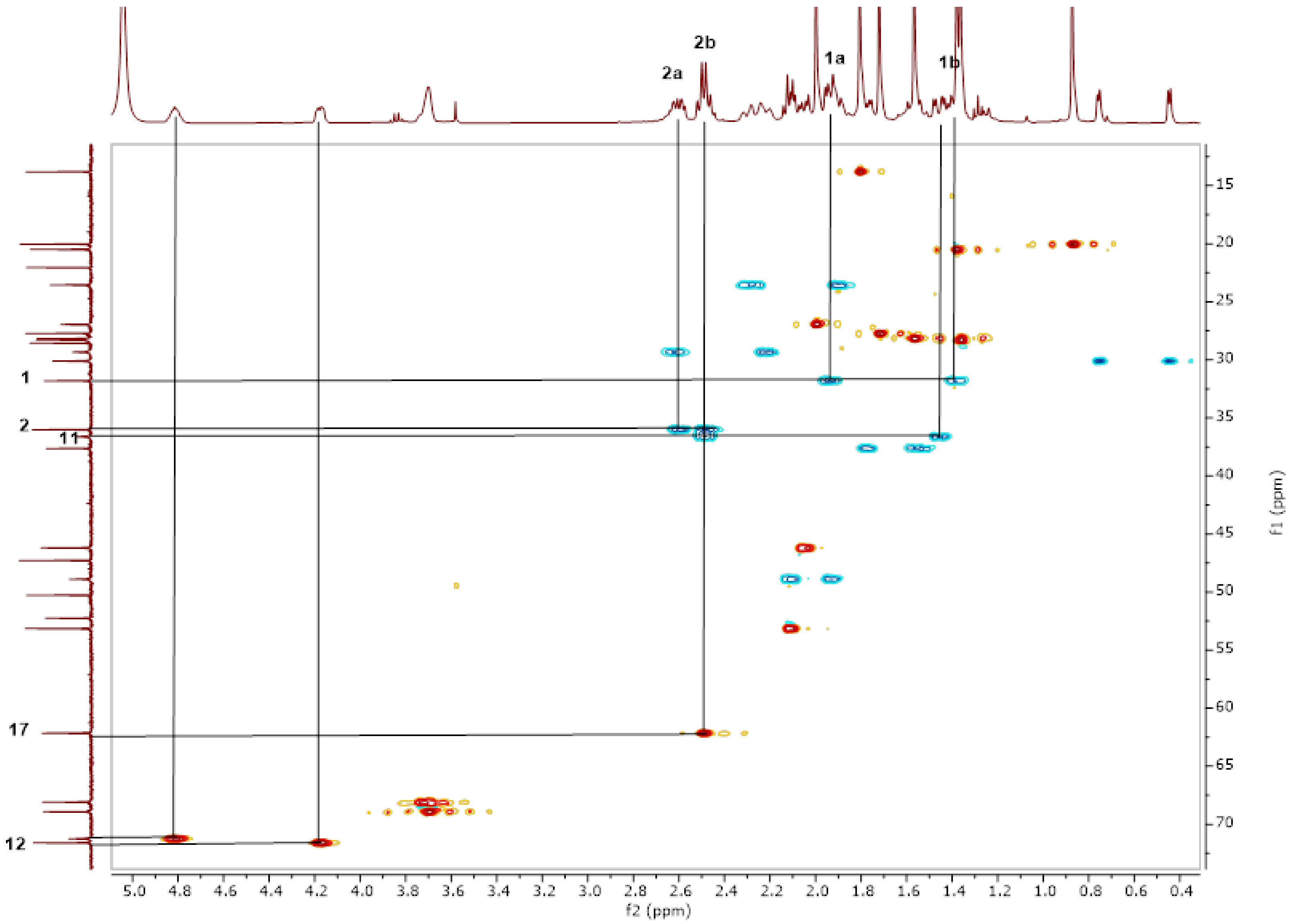

Figure S 24 HSQC spectrum of compound **3**

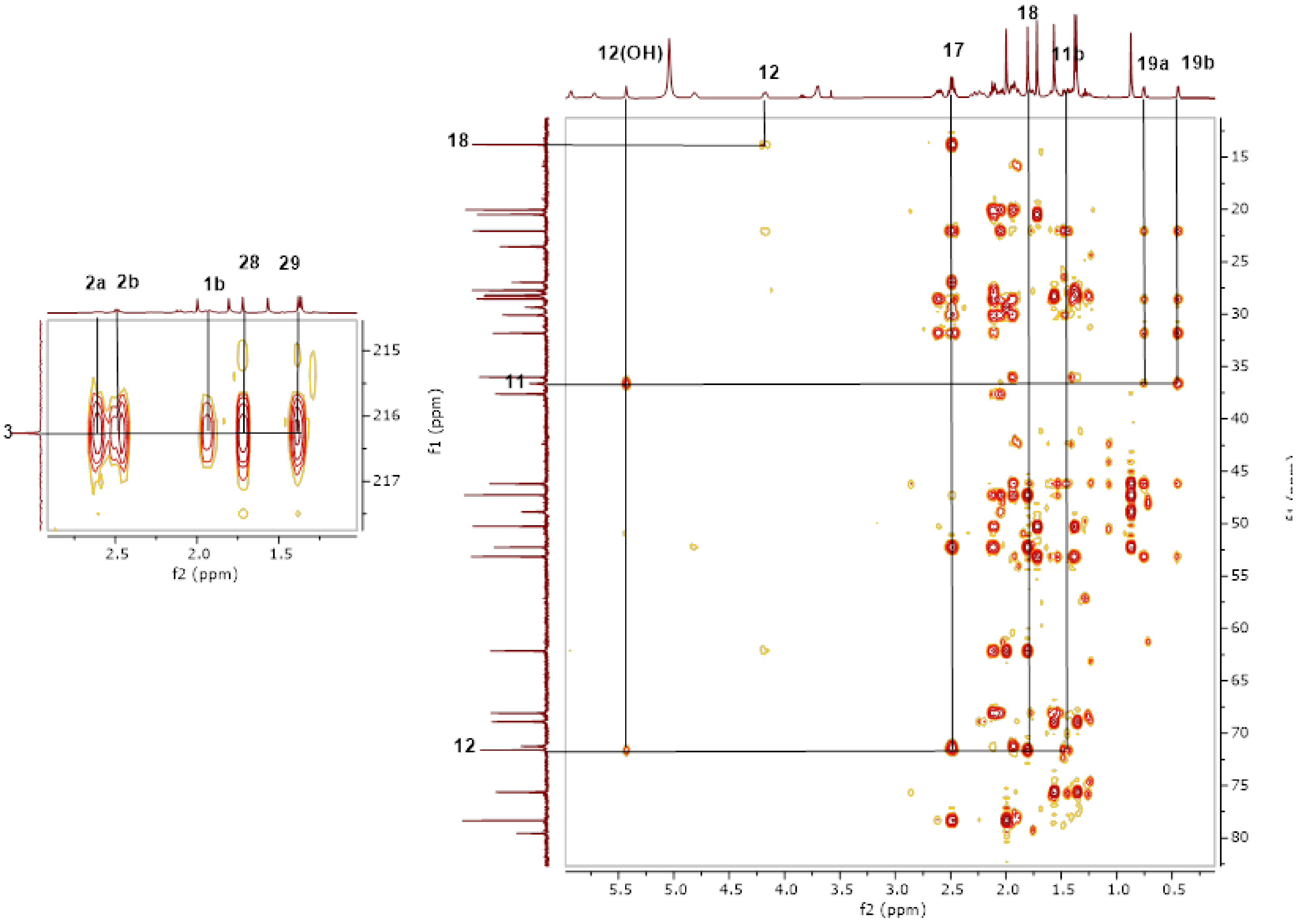

Figure S 25 HMBC spectrum of compound **3**

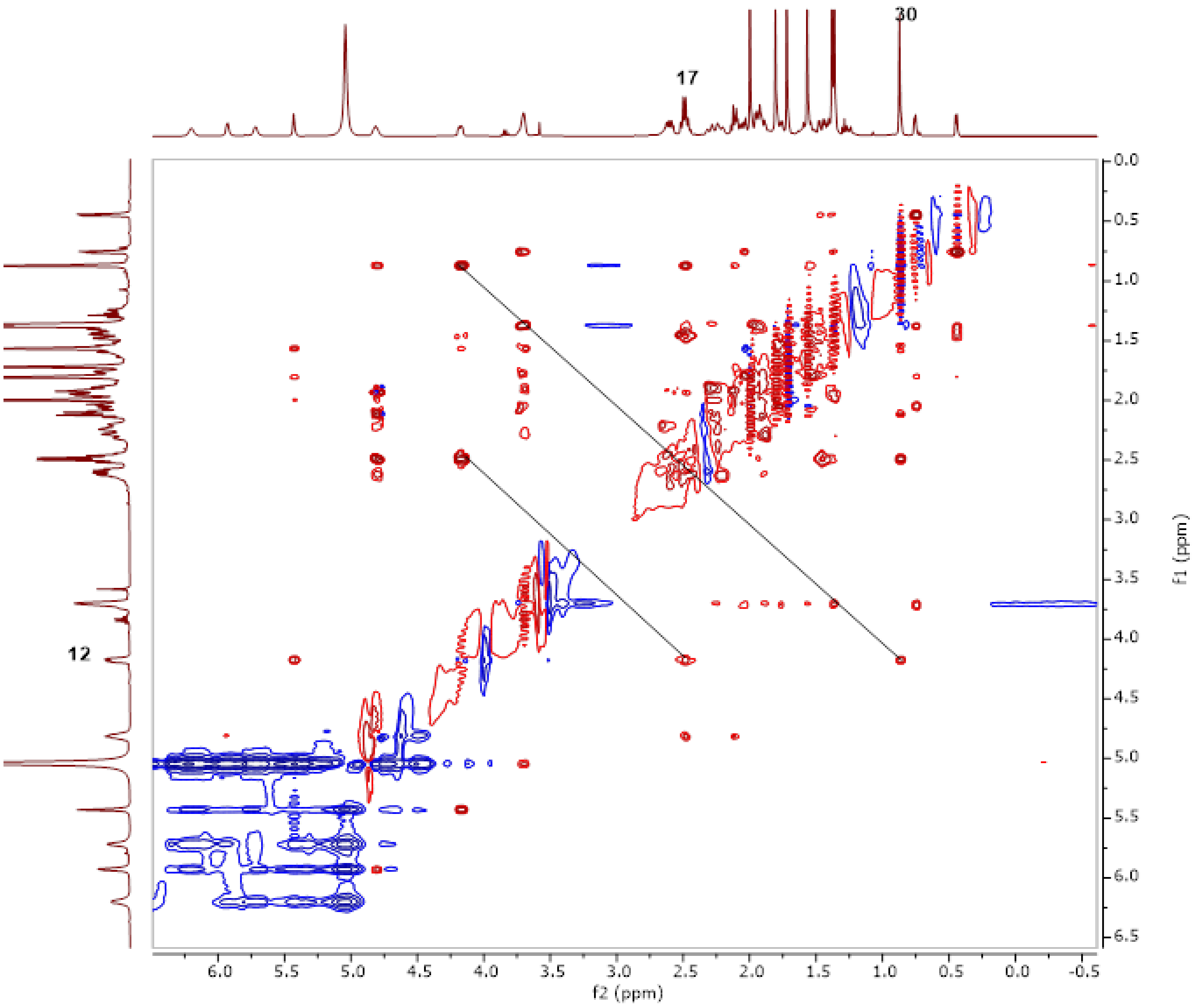

Figure S 26 NOESY spectrum of compound **3**

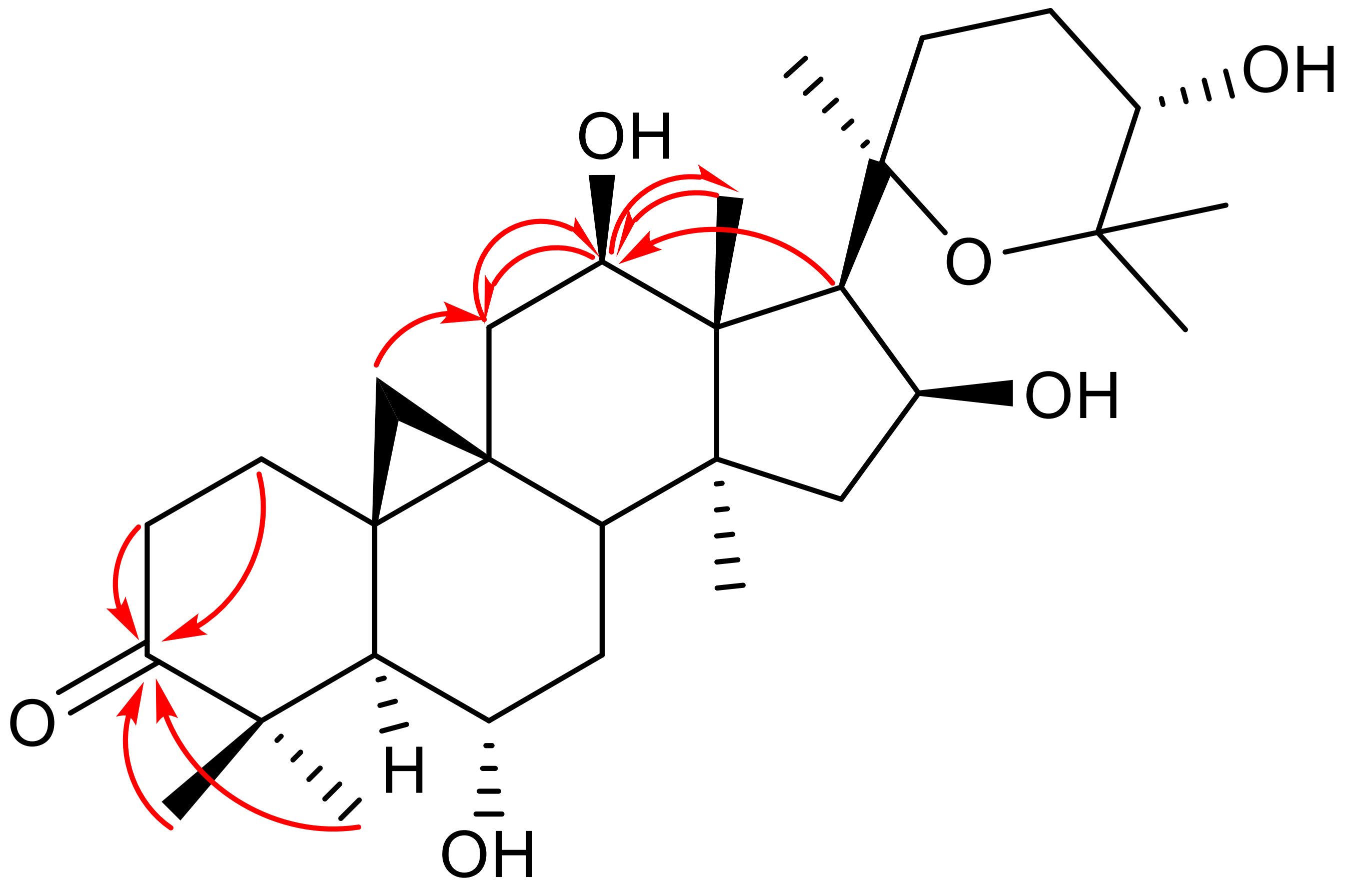

Figure S 27 Key HMBC correlations of compound **3** (arrows from H to C)

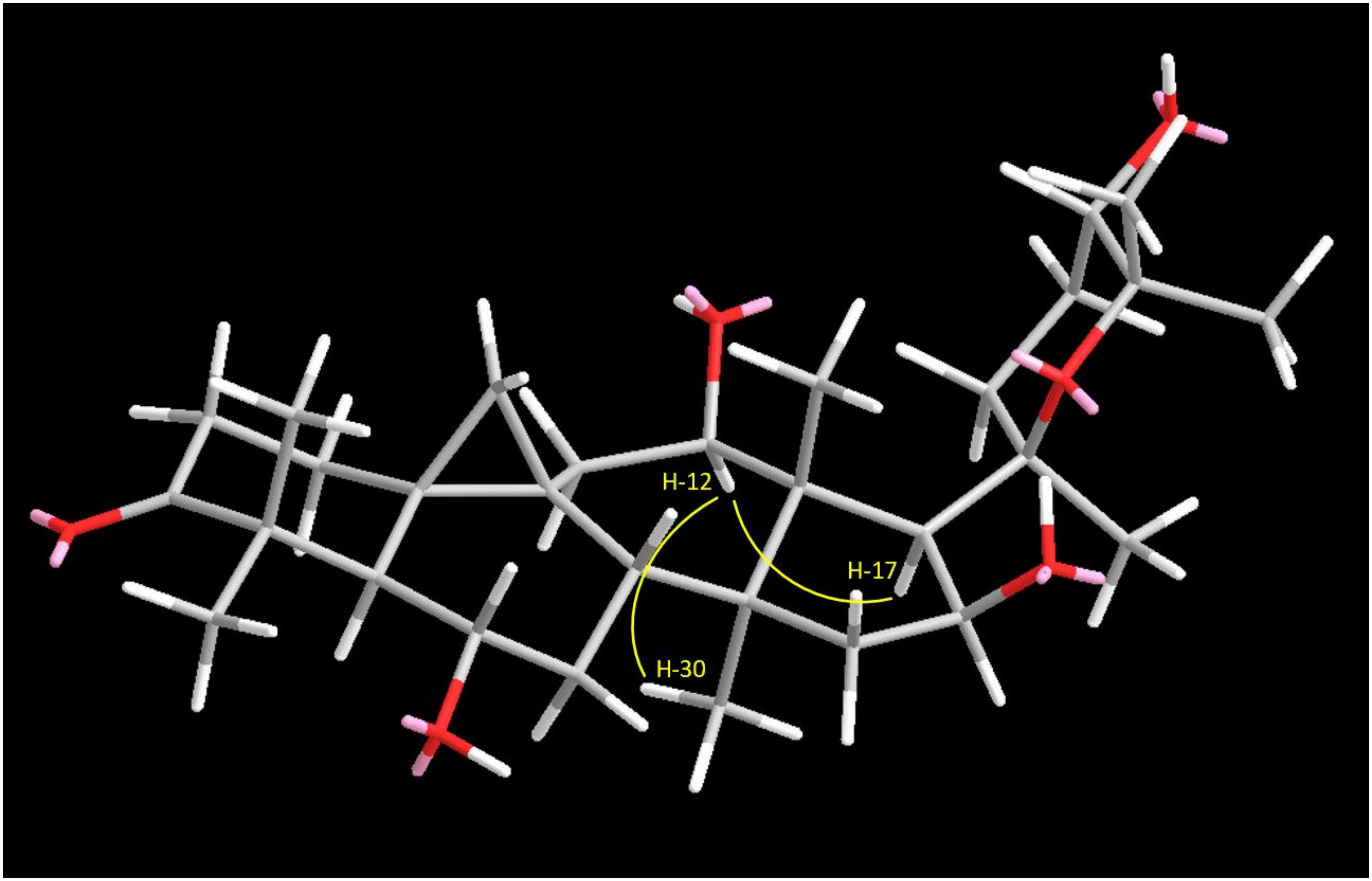

Figure S 28 Key NOE correlations of compound **3**

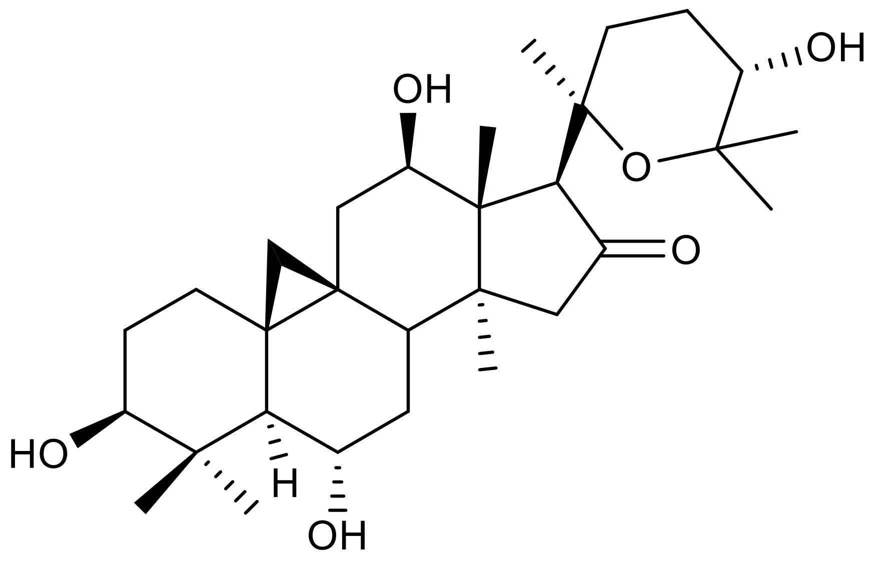

Figure S 29 Structure of compound **4**

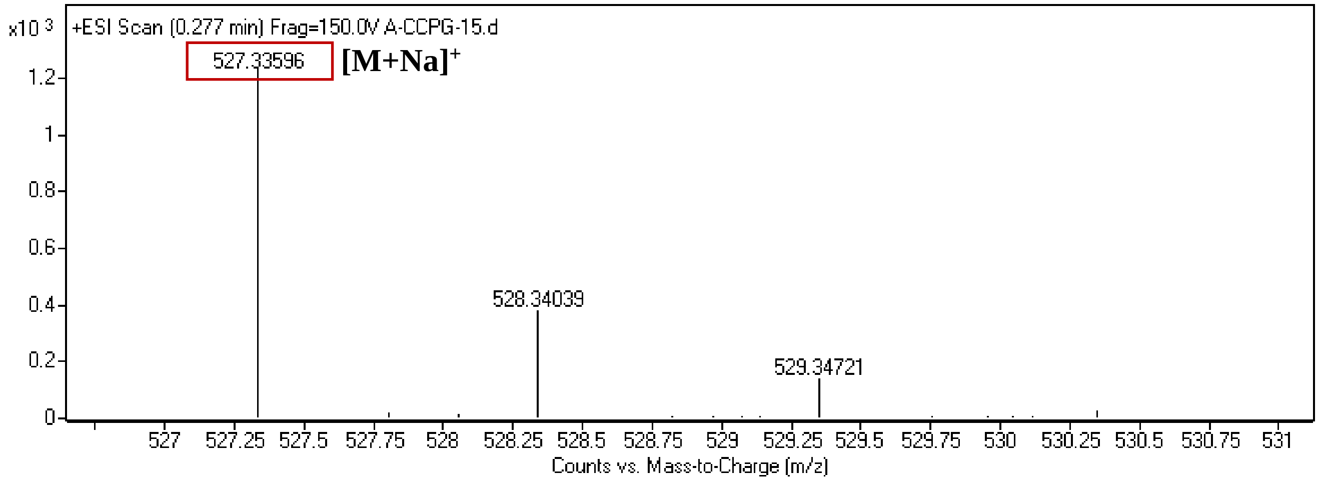

Figure S 30 HR-ESI-MS spectrum of compound **4**

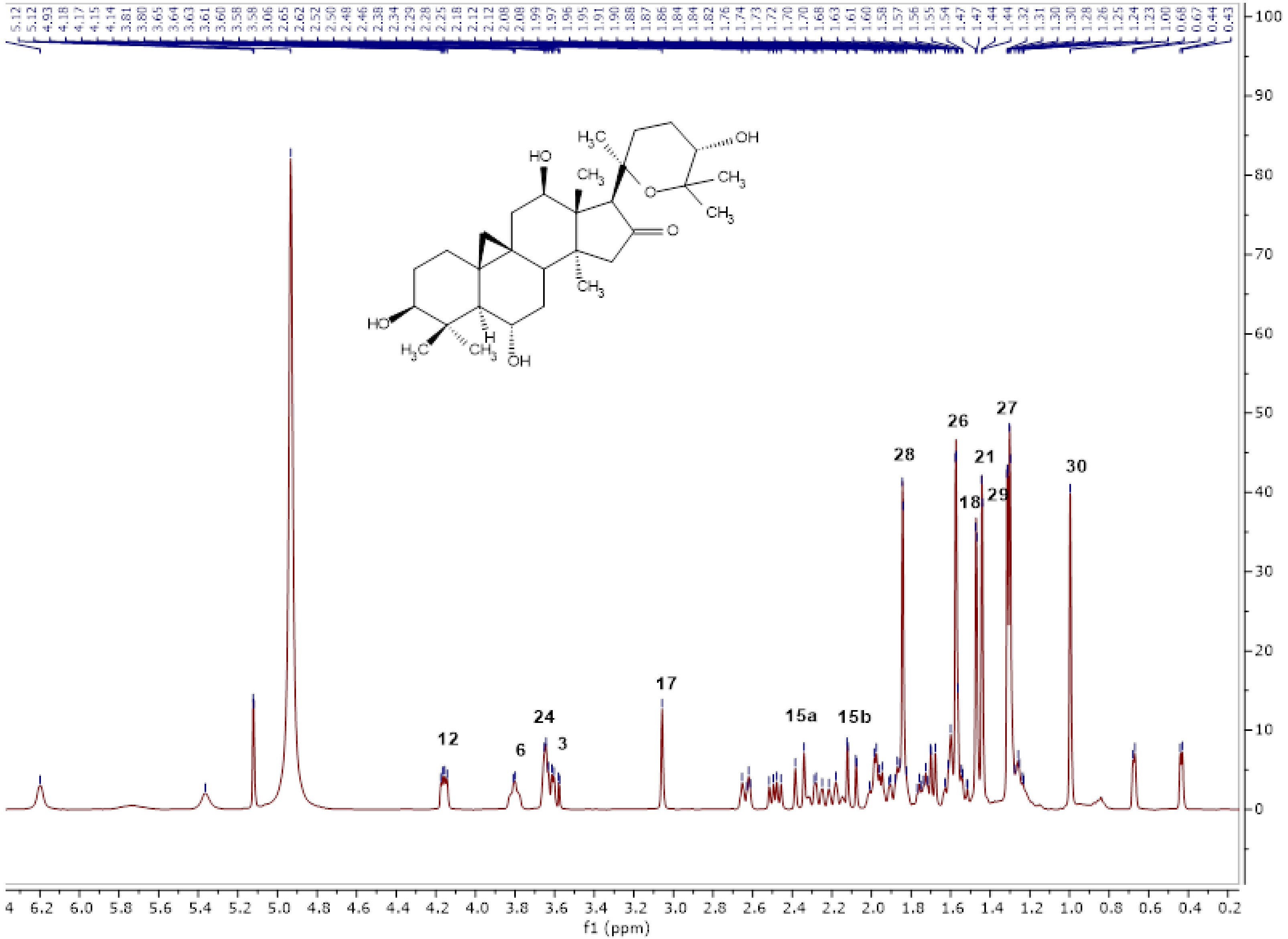

Figure S 31 ^{1}H-NMR spectrum of compound **4** (400 MHz, C_5D_5N)

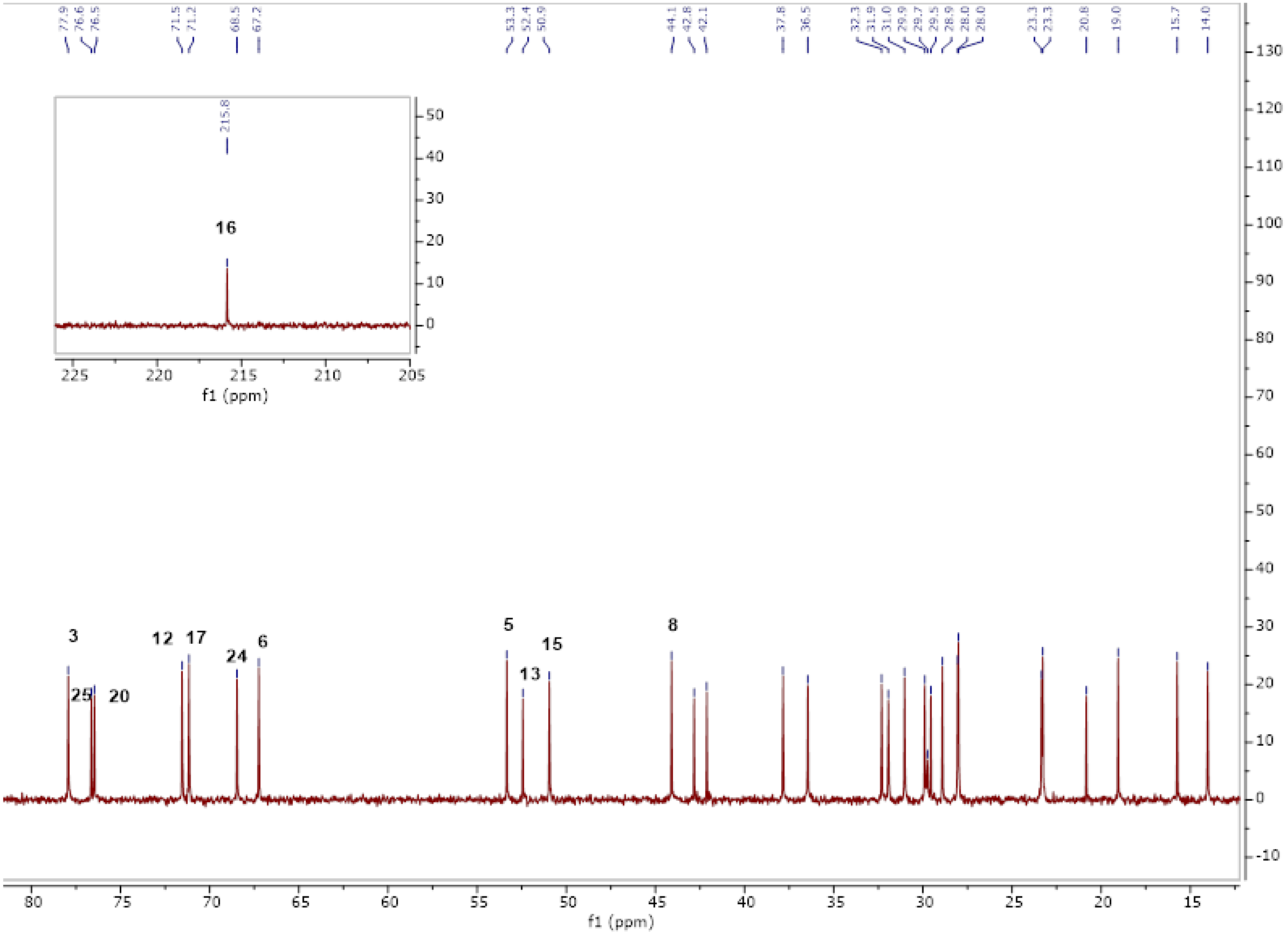

Figure S 32 ^{13}C-NMR spectrum of compound **4** (100 MHz, C_5D_5N)

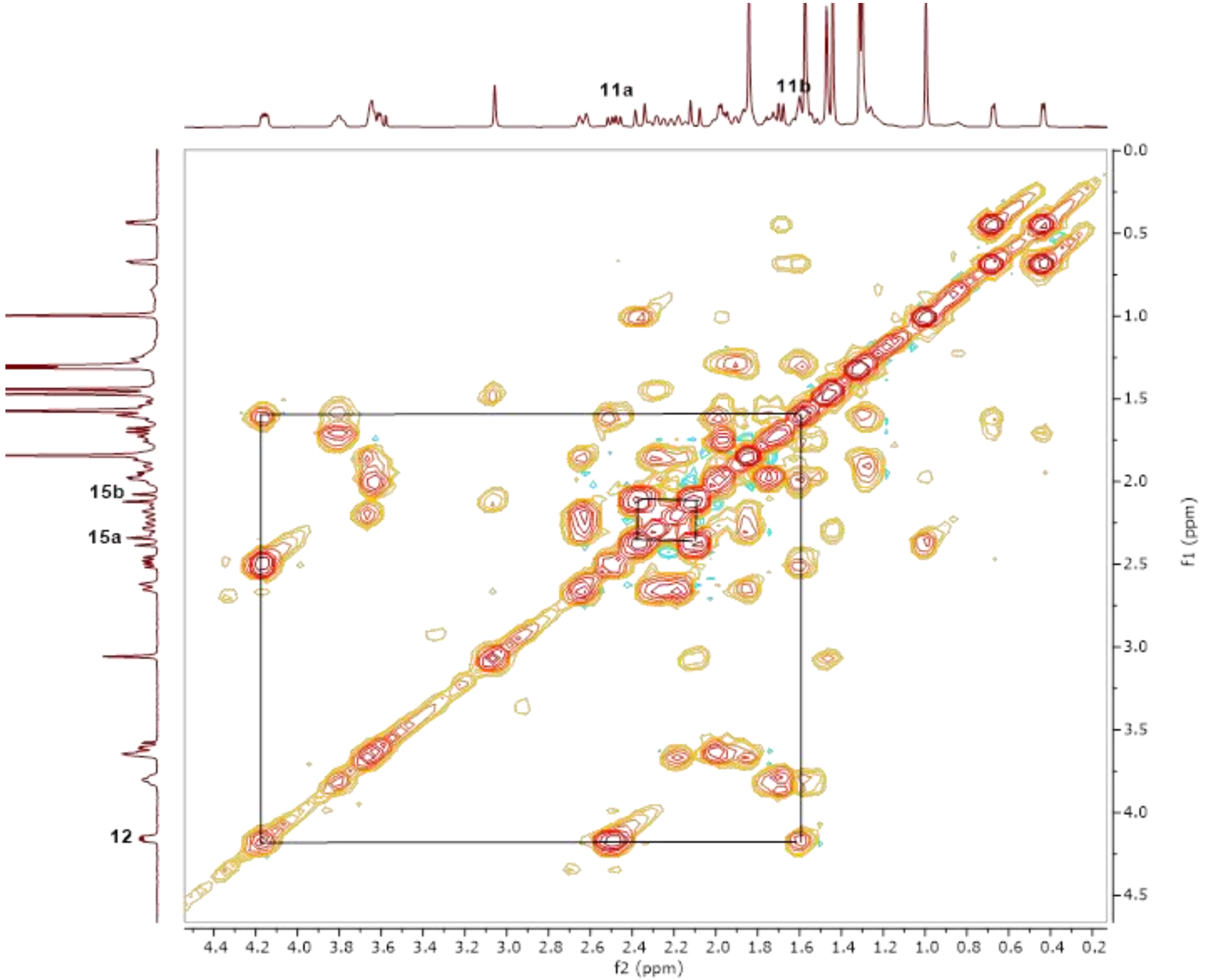

Figure S 33 COSY spectrum of compound **4**

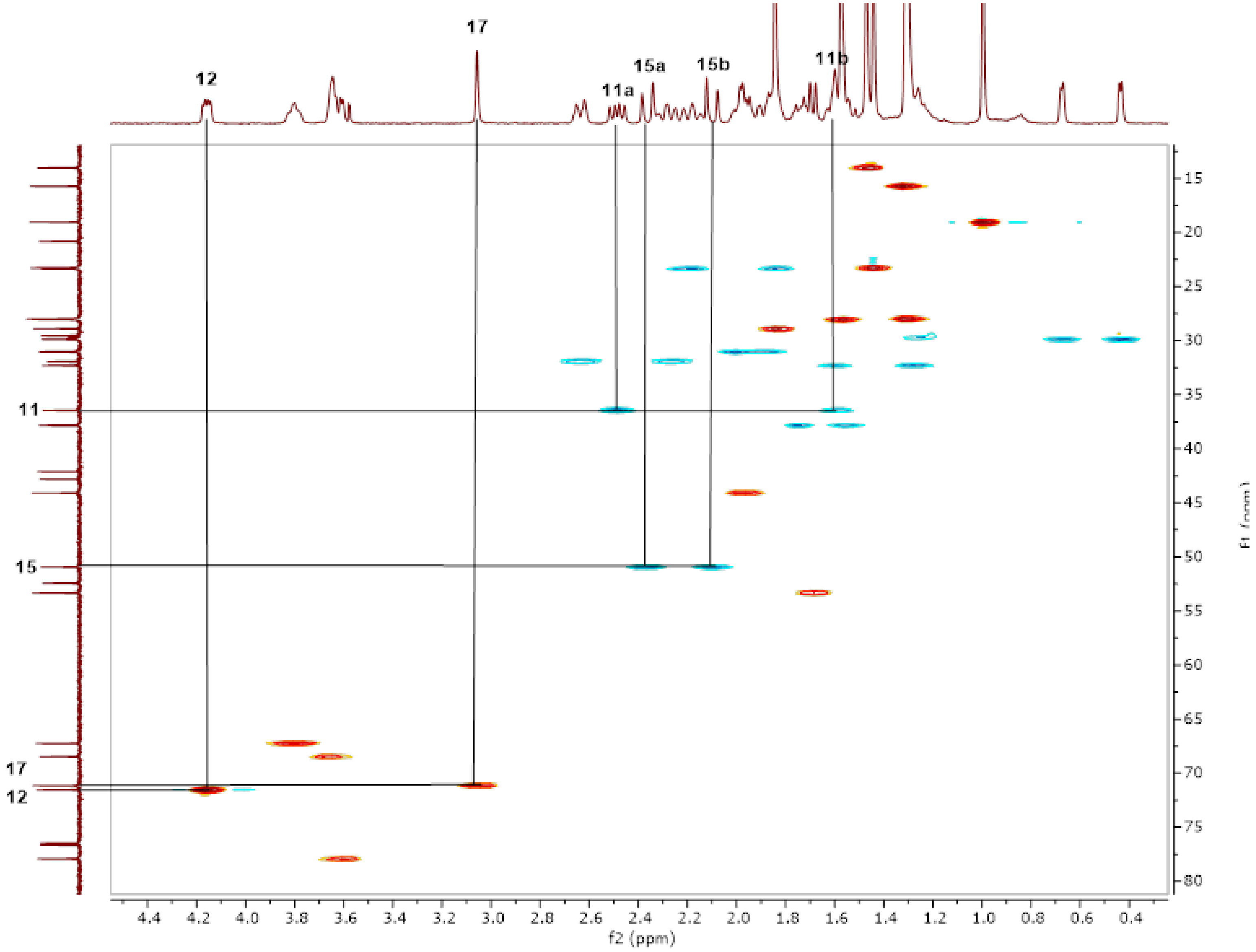

Figure S 34 HSQC spectrum of compound **4**

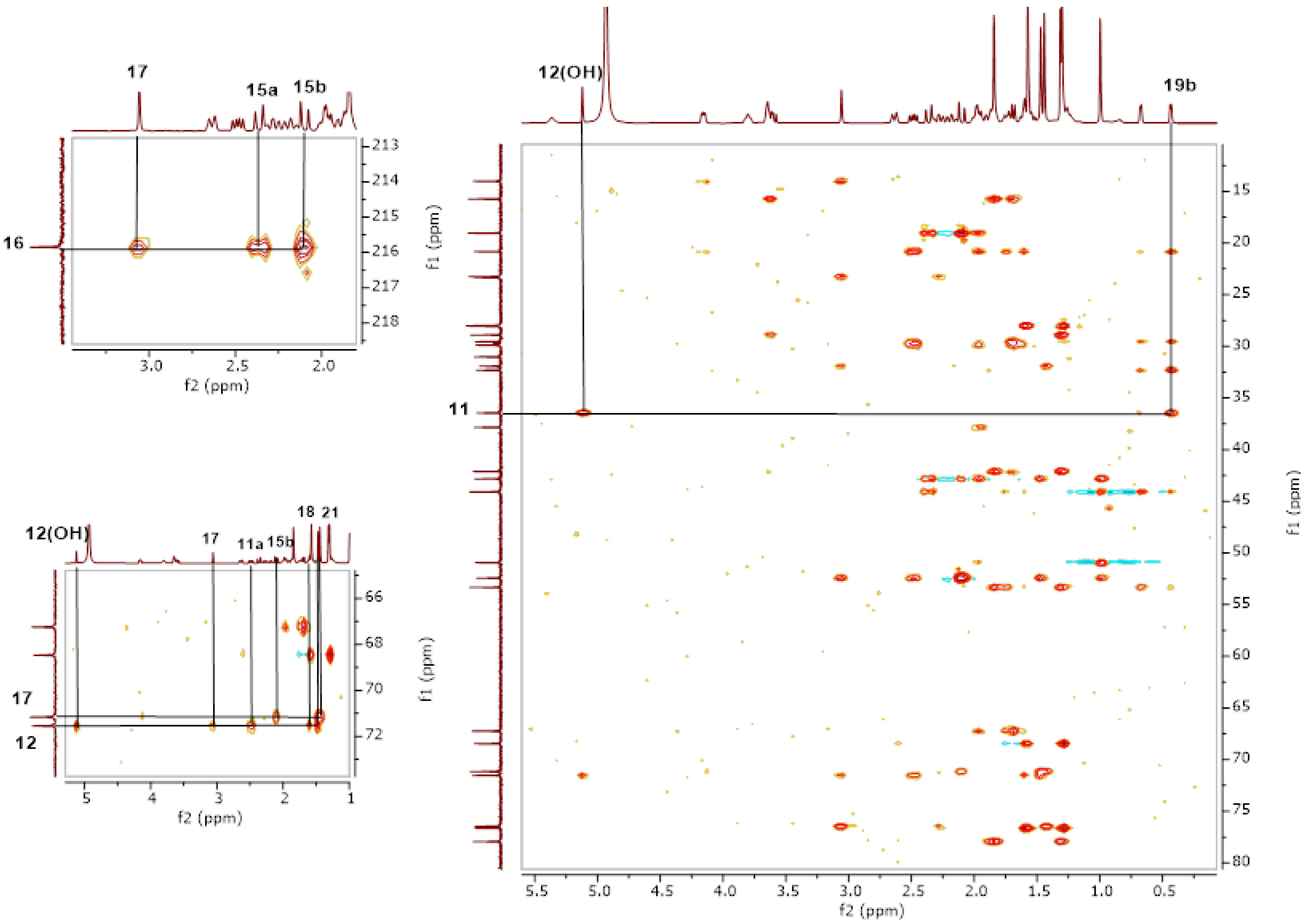

Figure S 35 HMBC spectrum of compound **4**

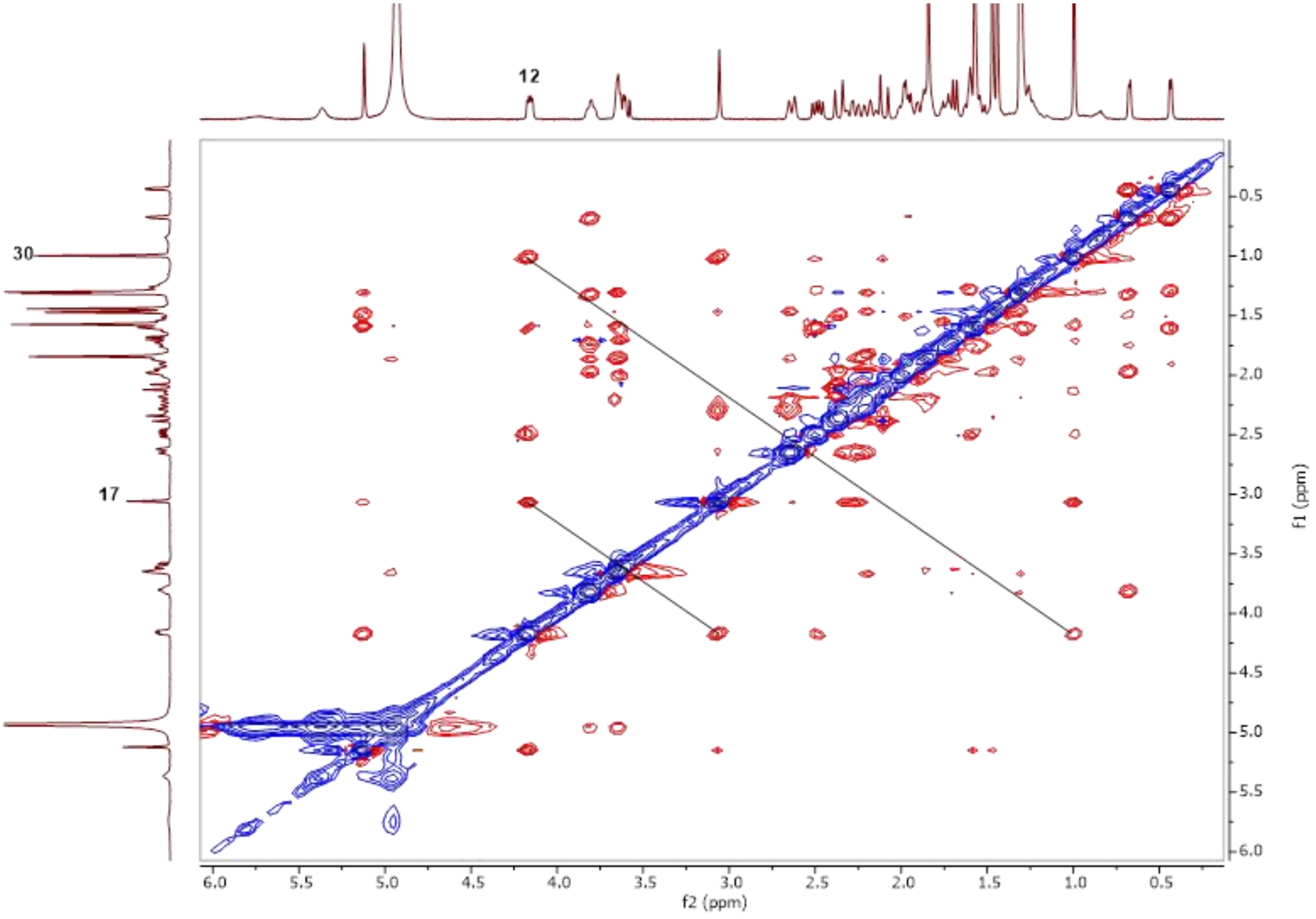

Figure S 36 NOESY spectrum of compound **4**

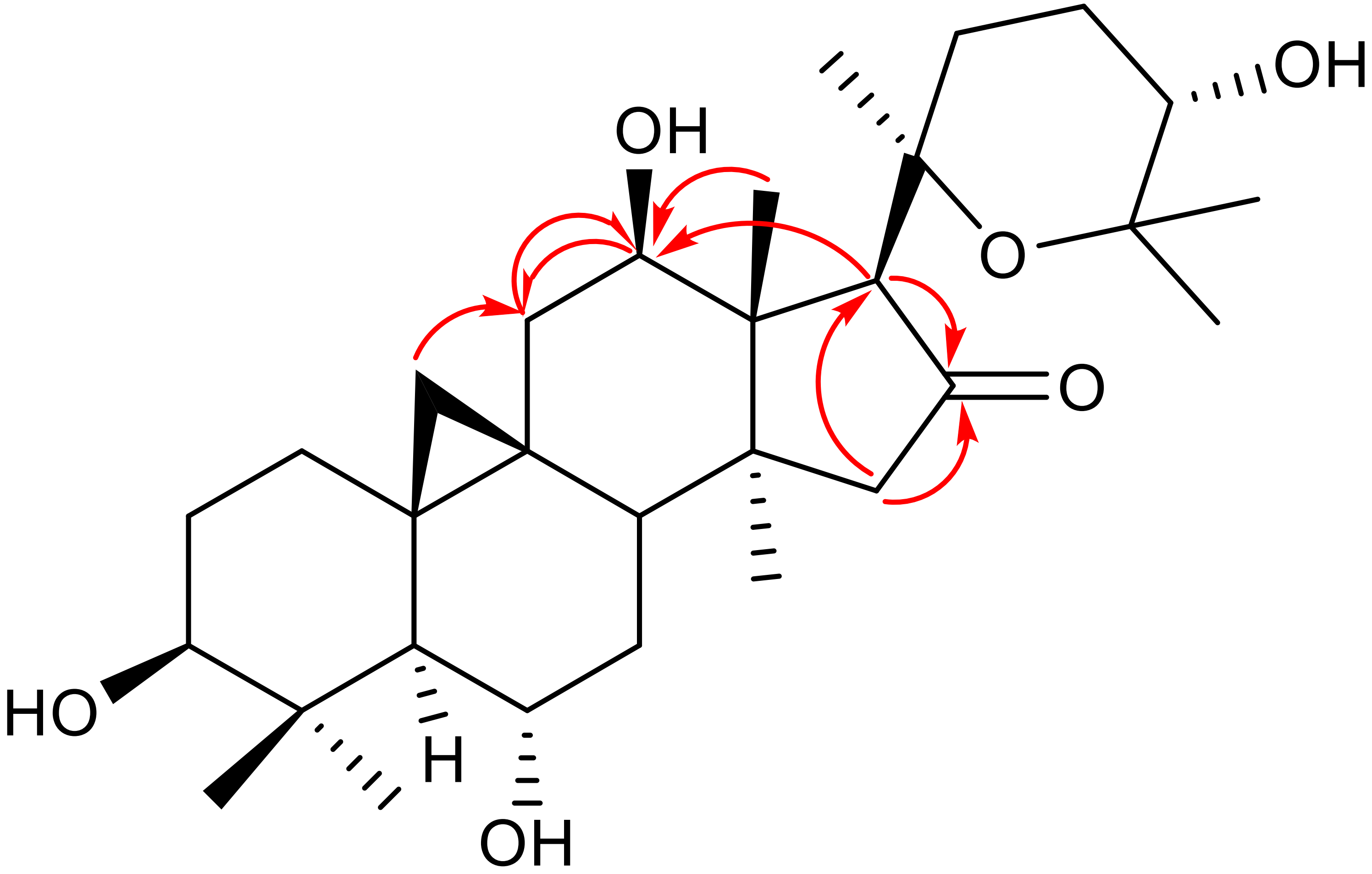

Figure S 37 Key HMBC correlations of compound **4** (arrows from H to C)

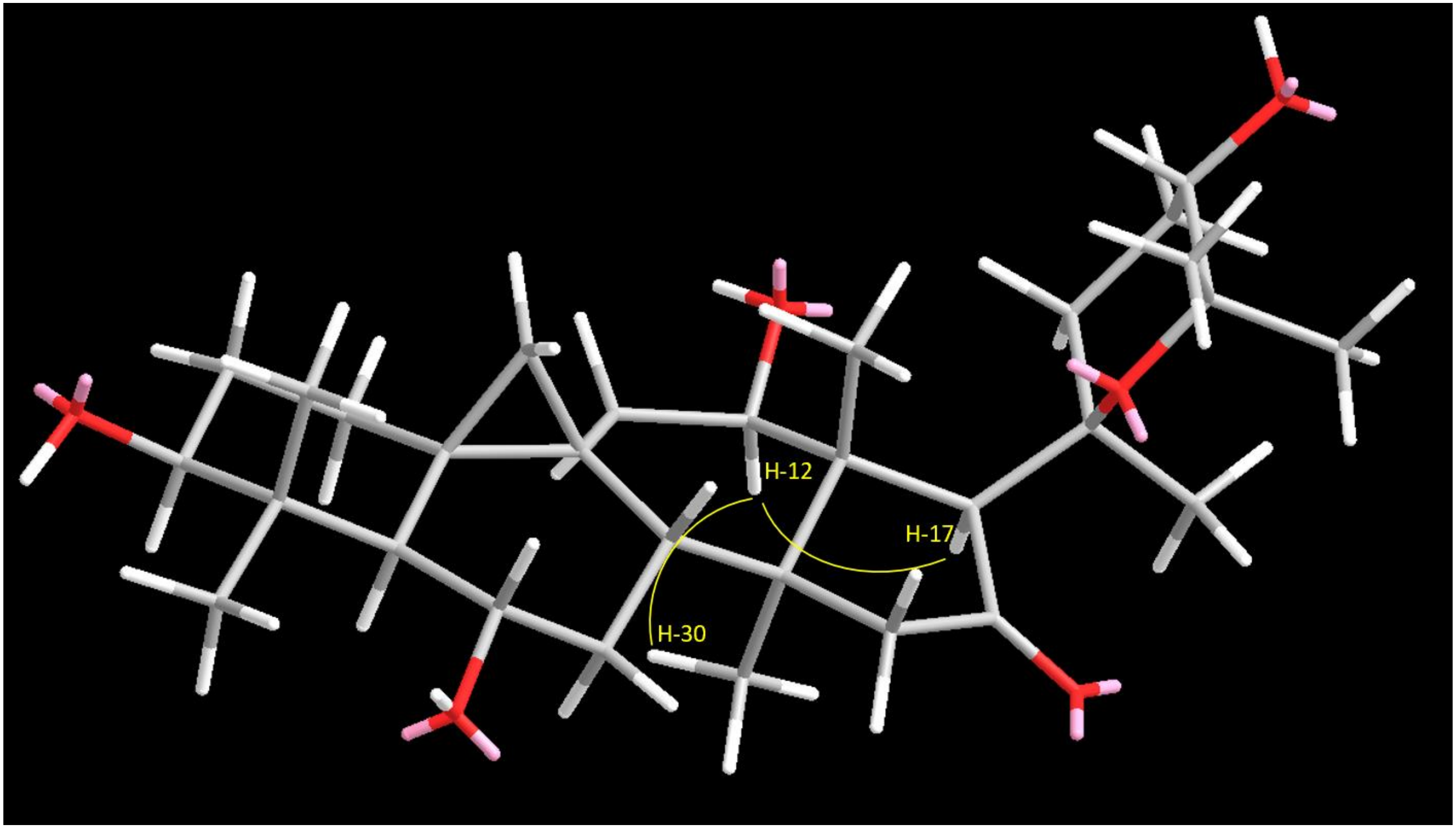

Figure S 38 Key NOE correlations of compound **4**

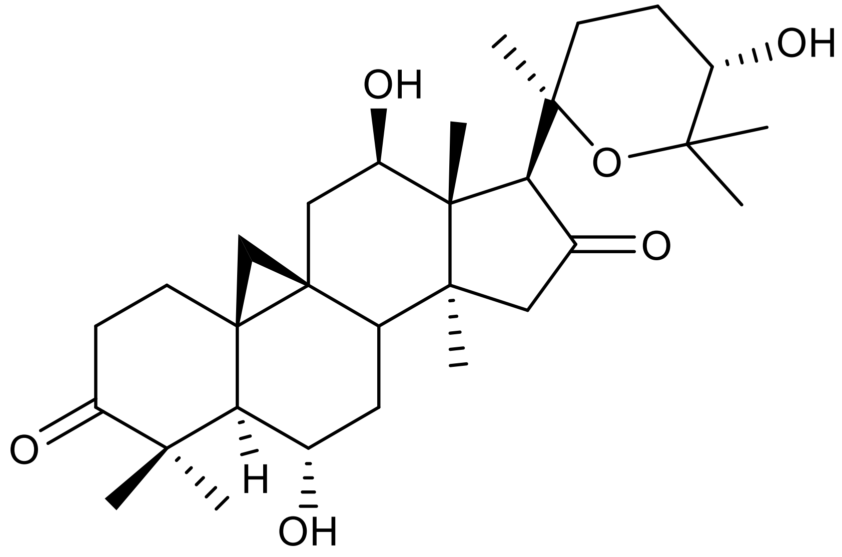

Figure S 39 Structure of compound **5**

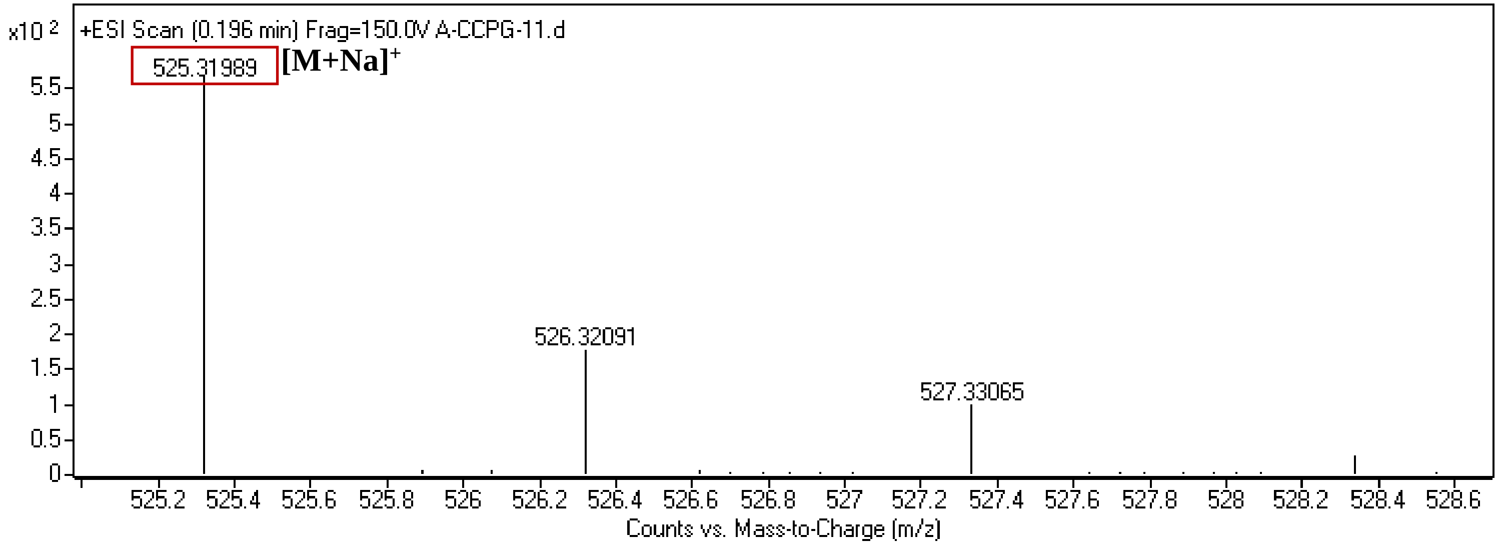

Figure S 40 HR-ESI-MS spectrum of compound **5**

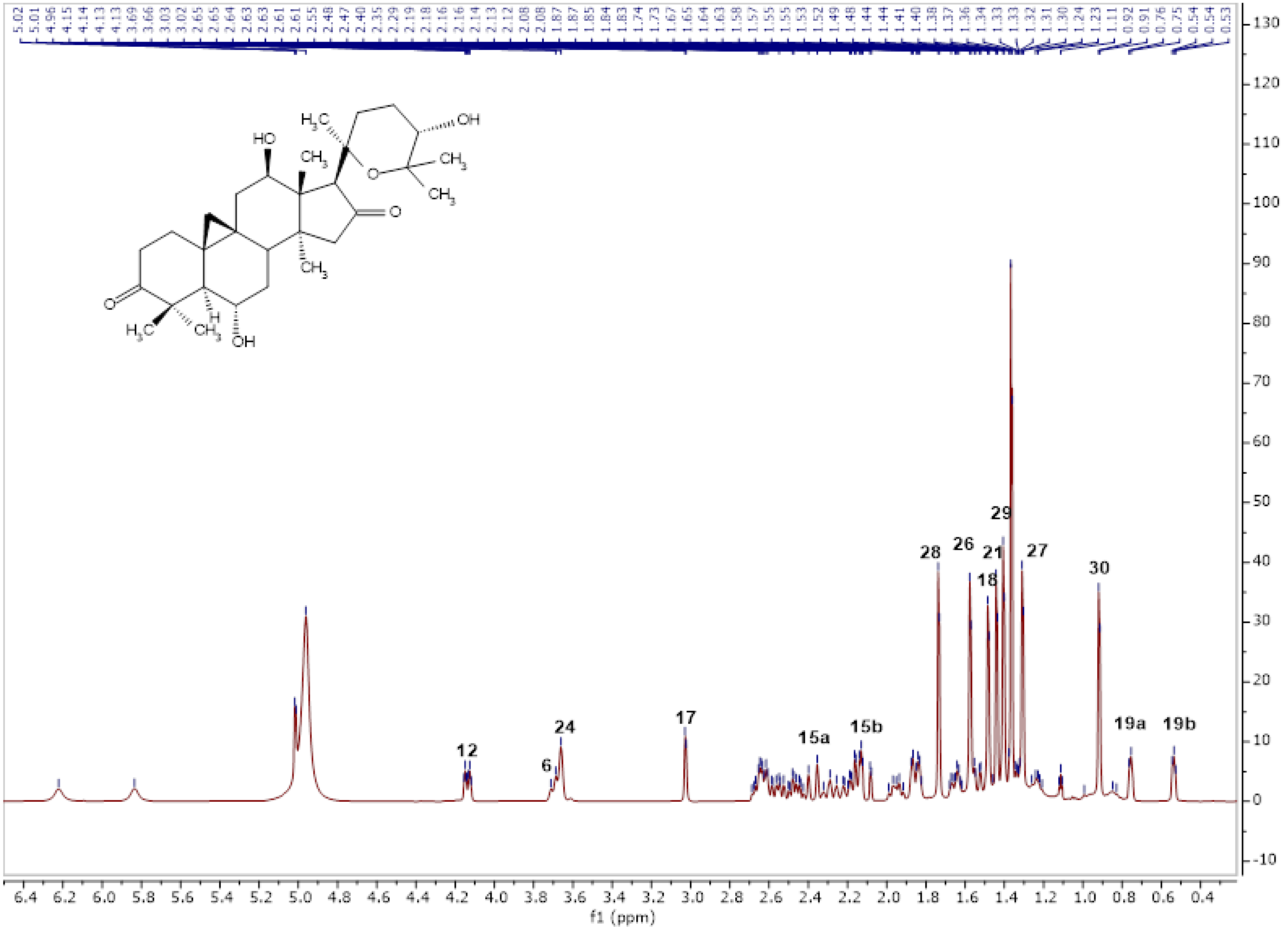

Figure S 41 ^{1}H-NMR spectrum of compound **5** (400 MHz, C_5D_5N)

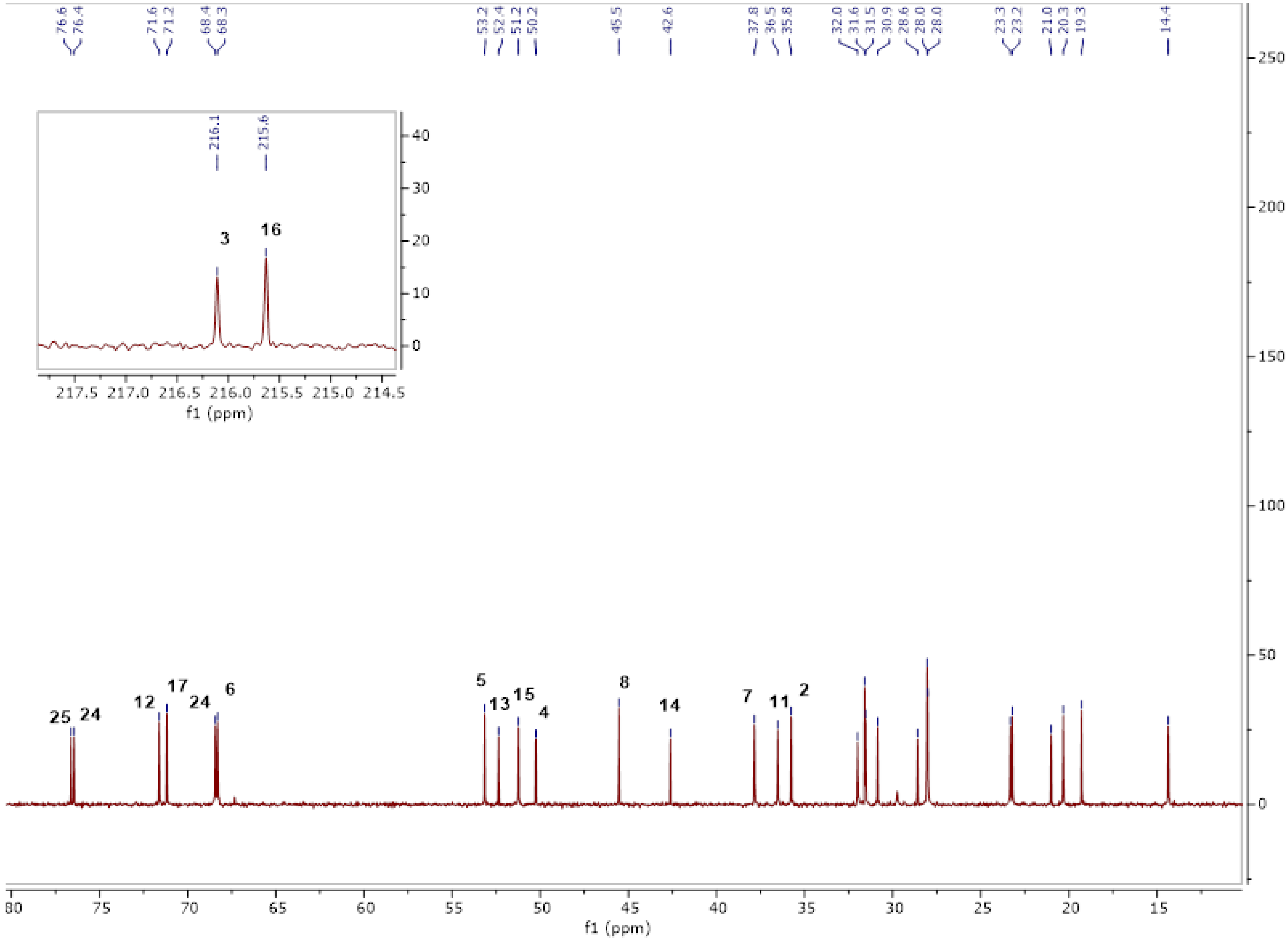

Figure S 42 ^{13}C-NMR spectrum of compound **5** (100 MHz, C_5D_5N)

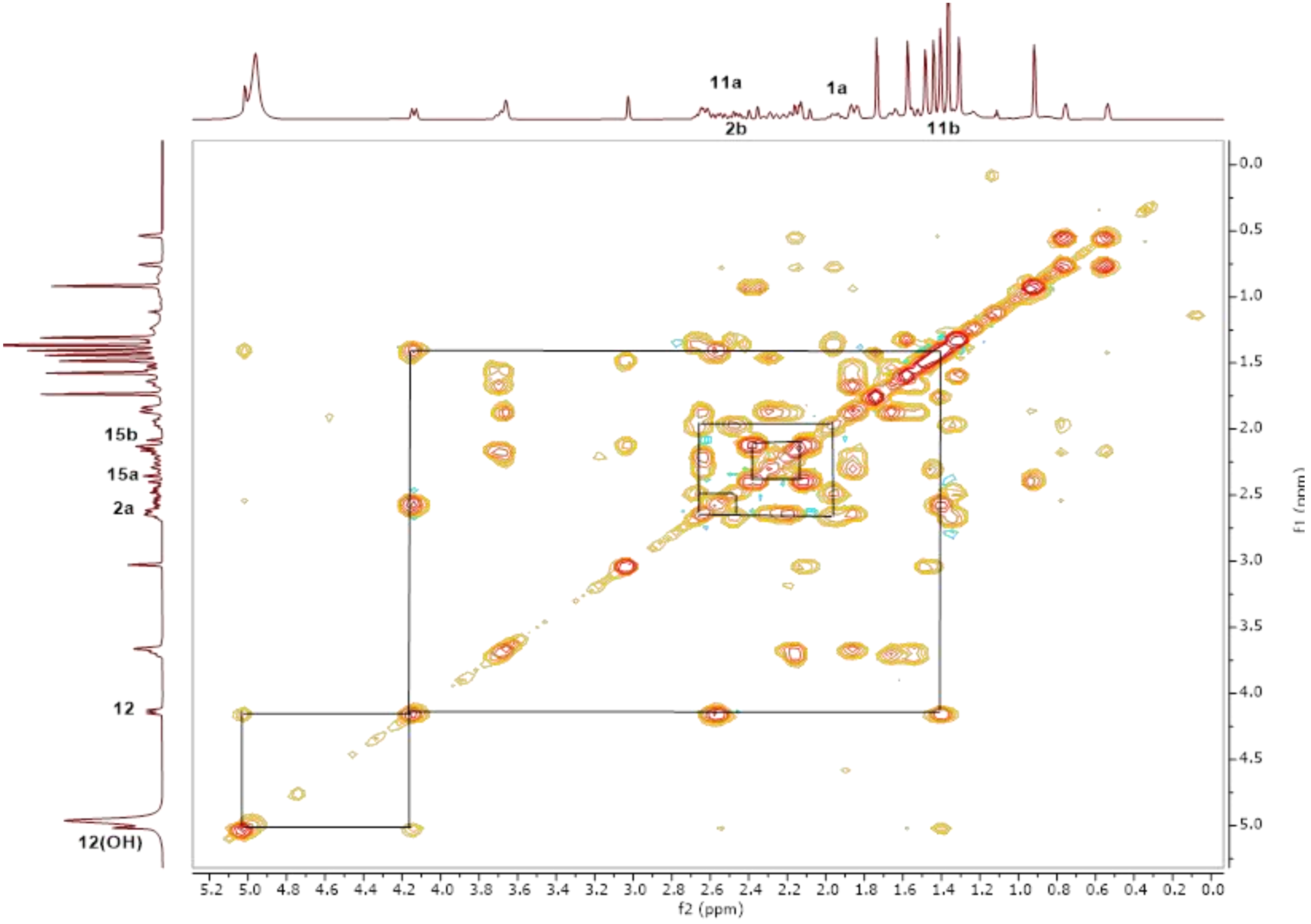

Figure S 43 COSY spectrum of compound **5**

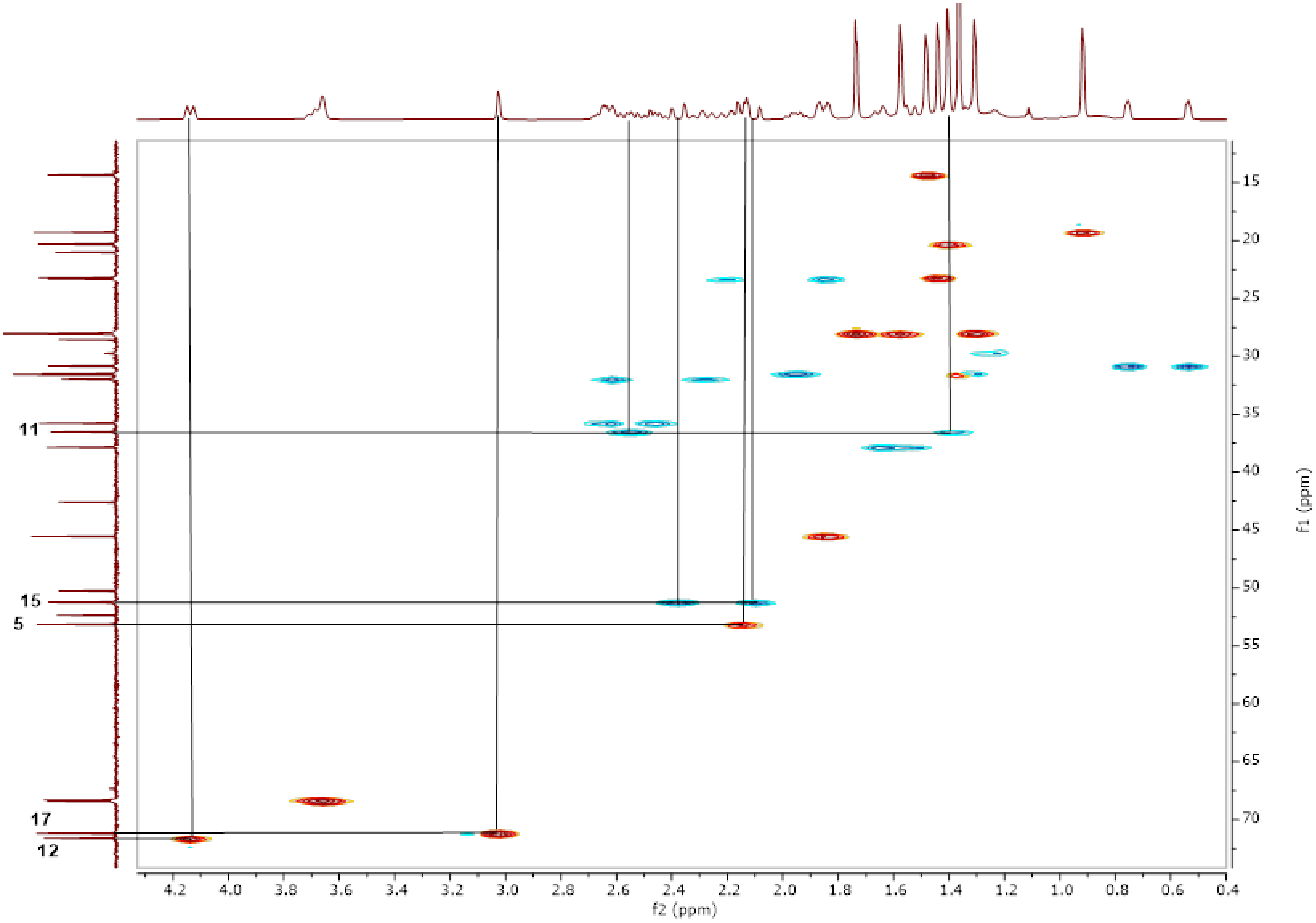

Figure S 44 HSQC spectrum of compound **5**

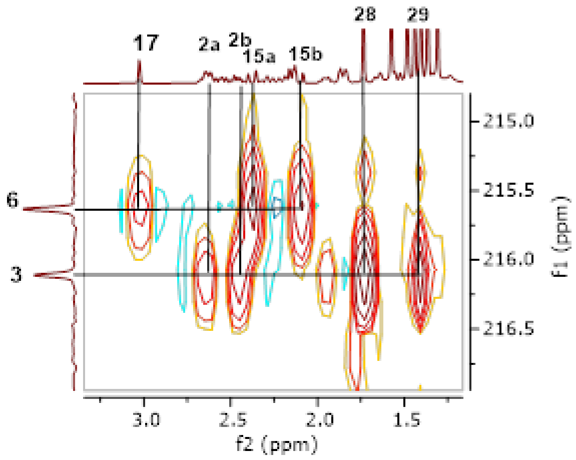

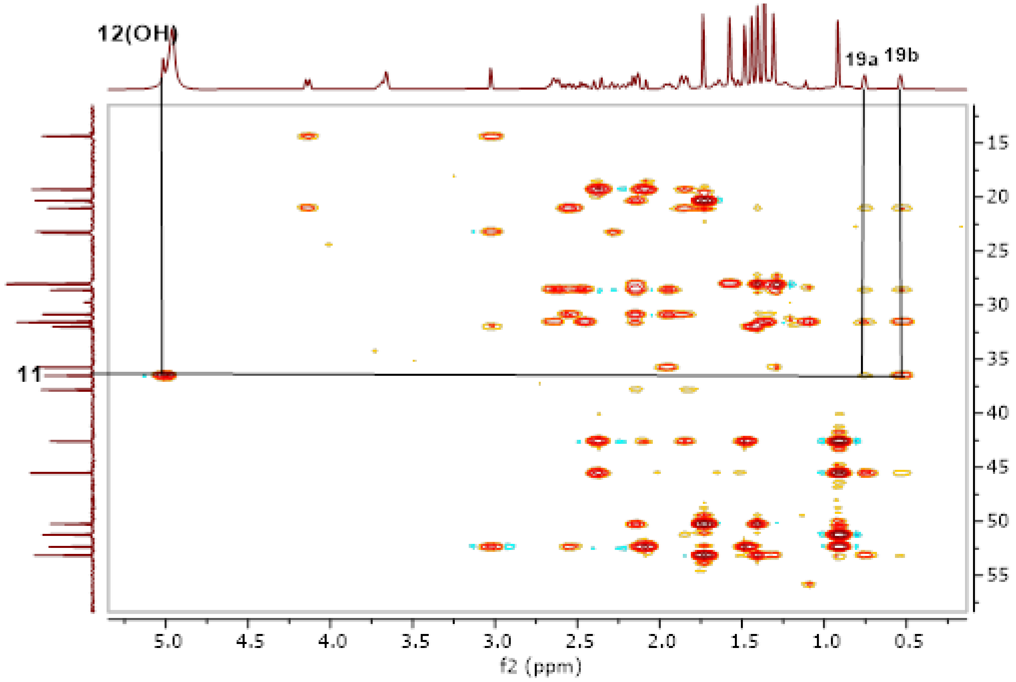

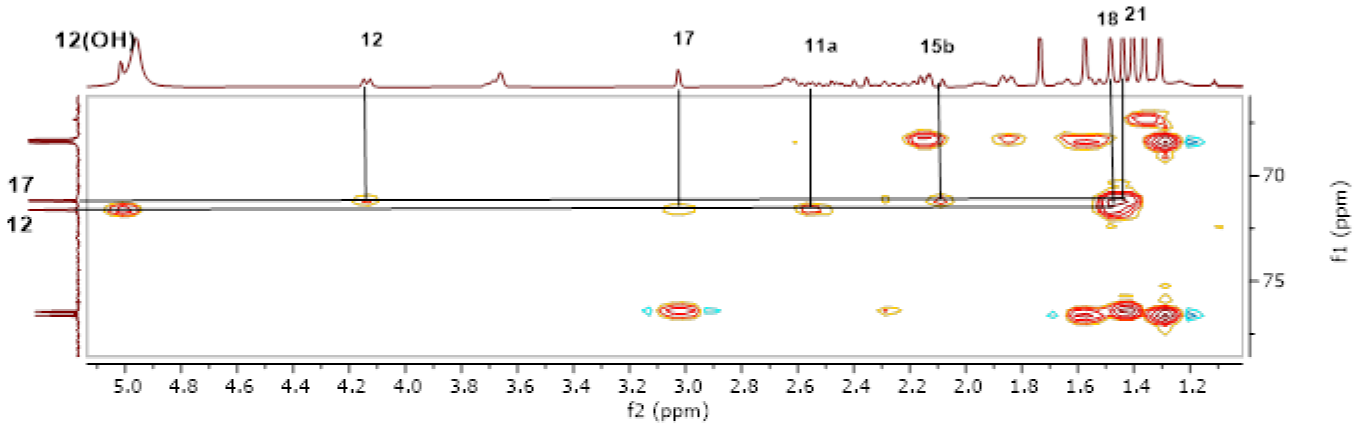

Figure S 45 HMBC spectrum of compound **5**

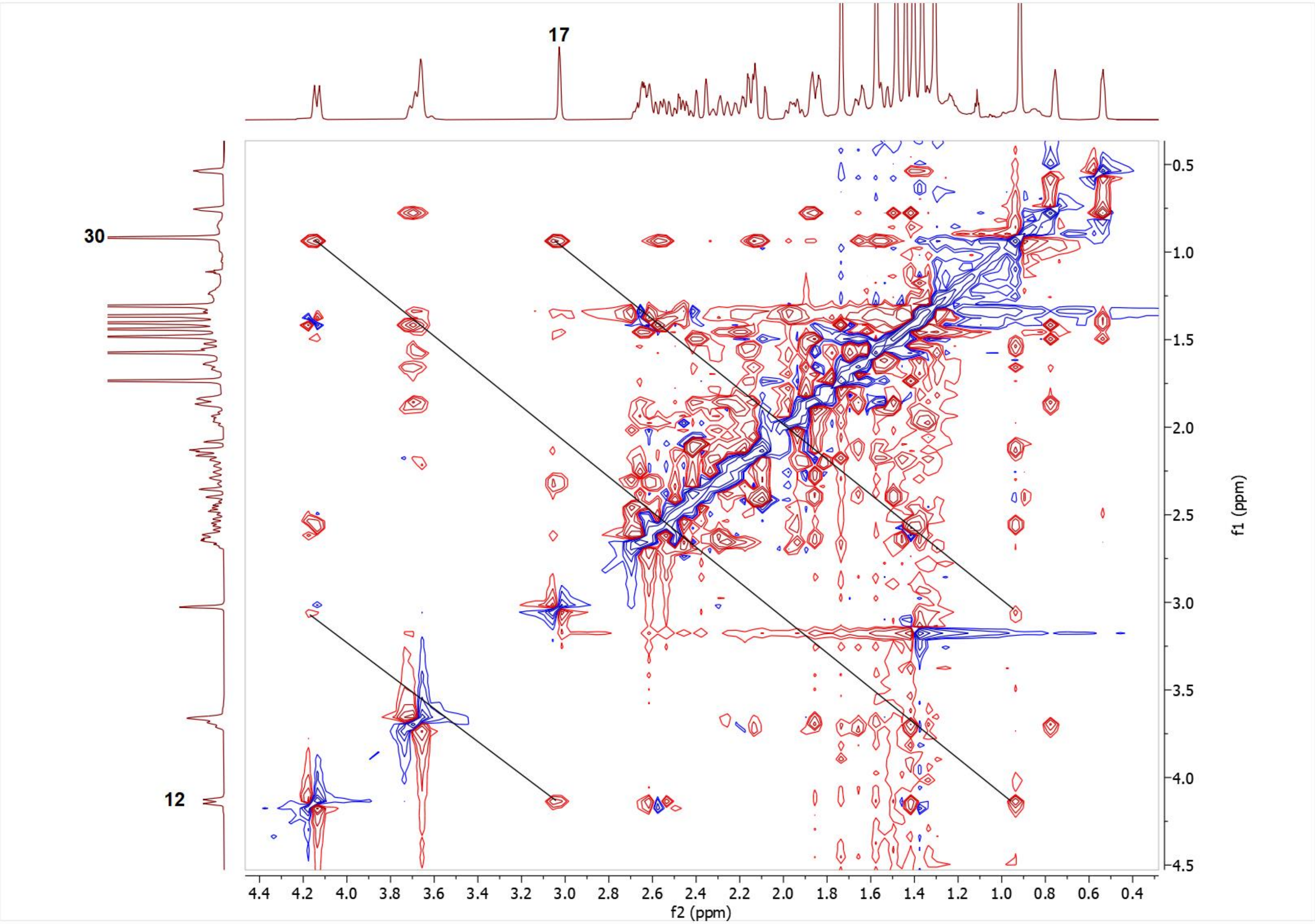

Figure S 46 NOESY spectrum of compound **5**

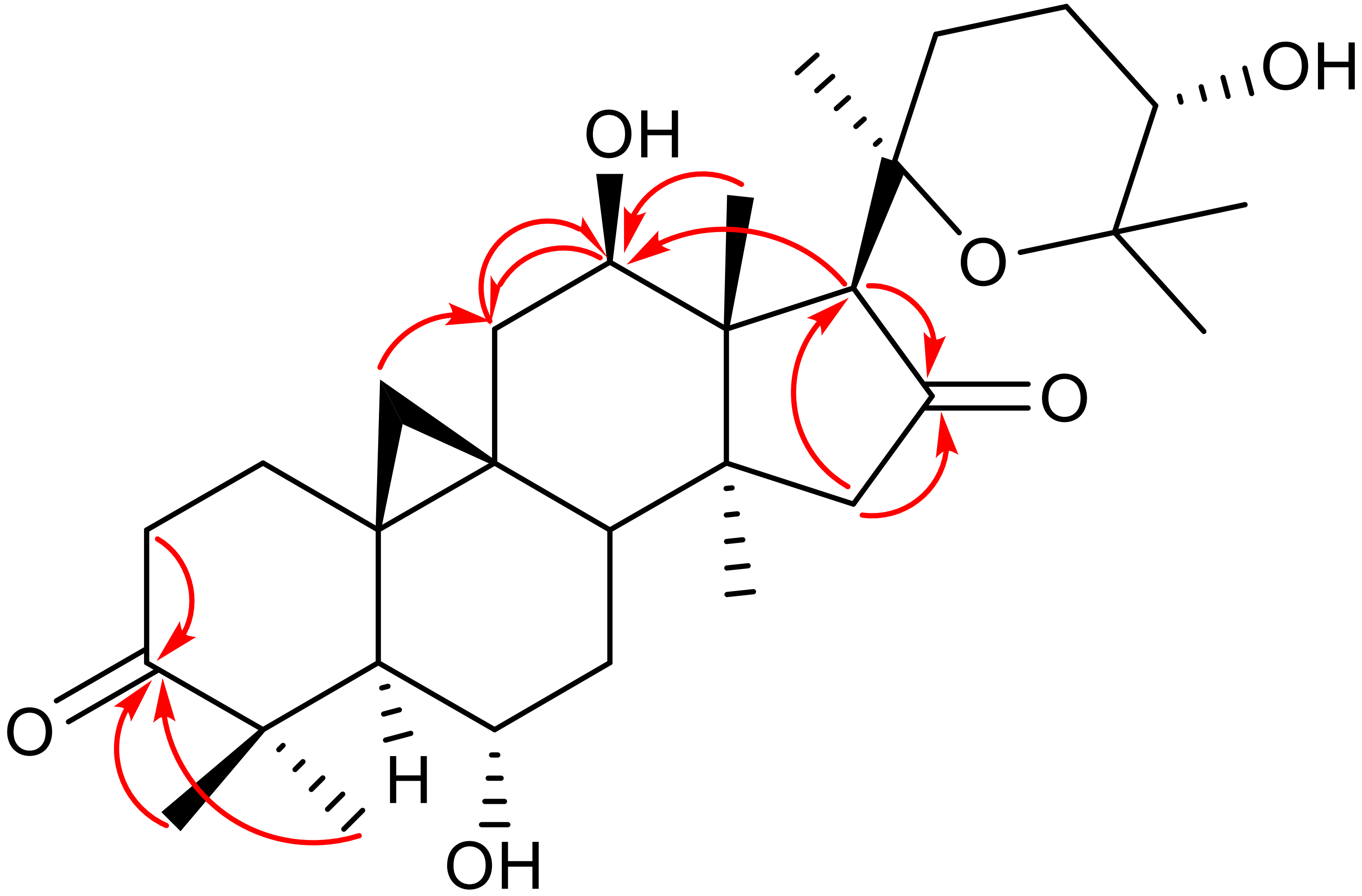

Figure S 47 Key HMBC correlations of compound **5** (arrows from H to C)

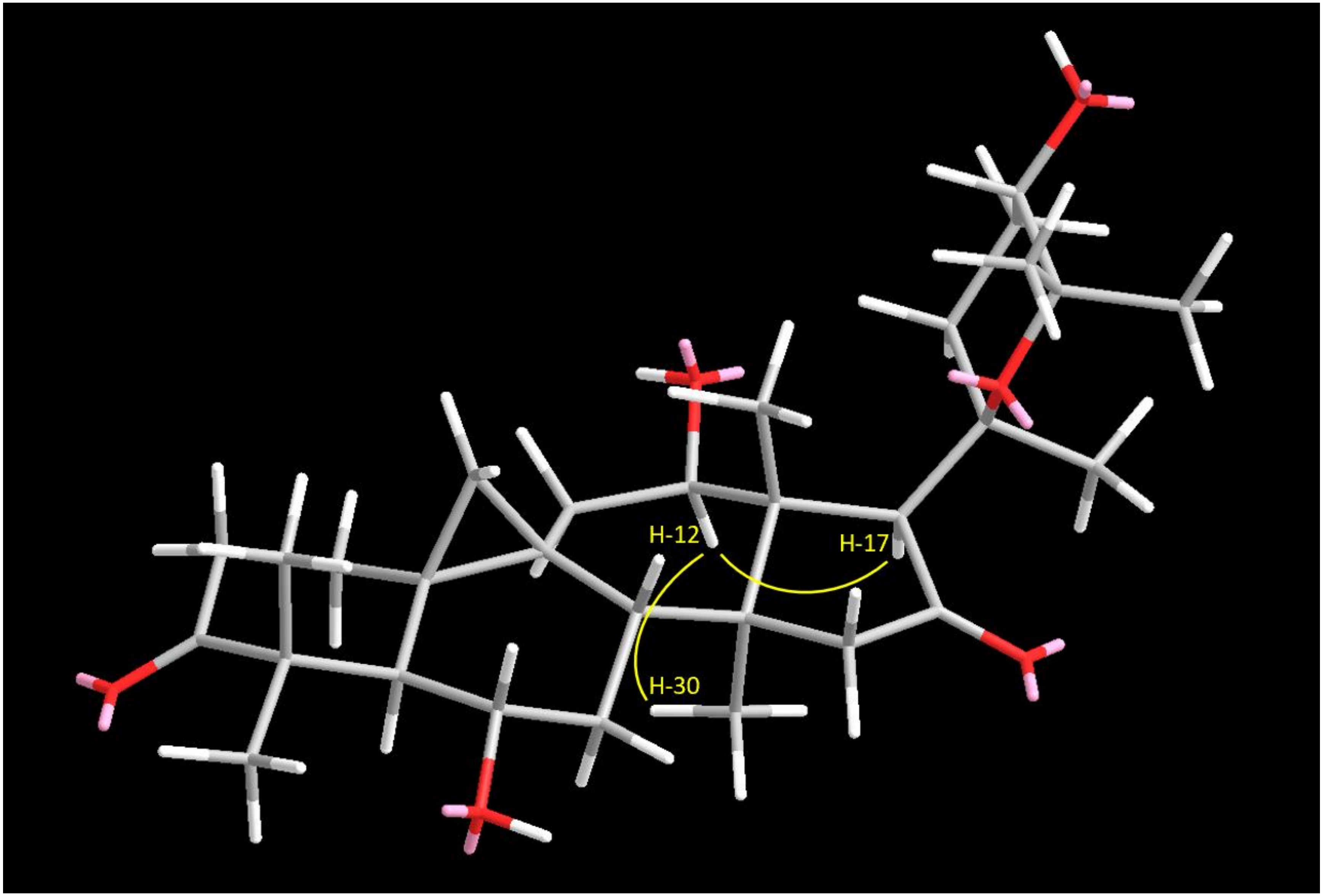

Figure S 48 Key NOE correlations of compound **5**

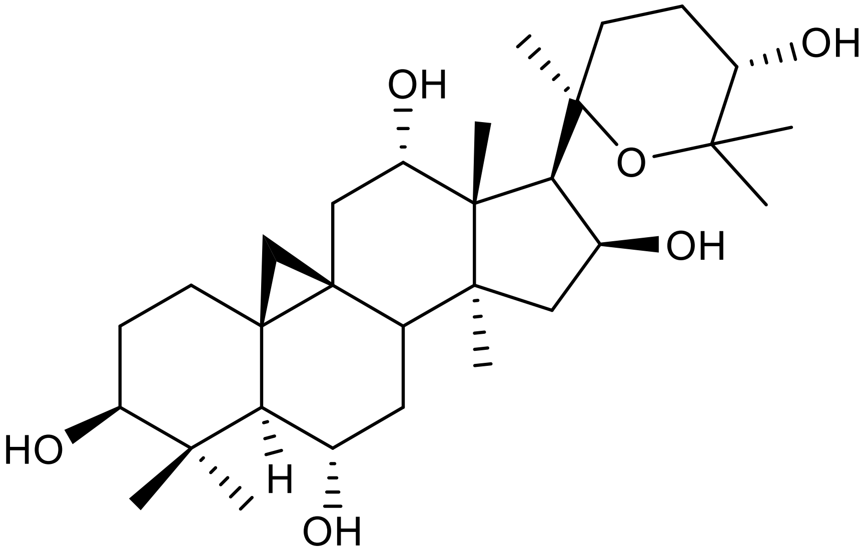

Figure S 49 Structure of compound **6**

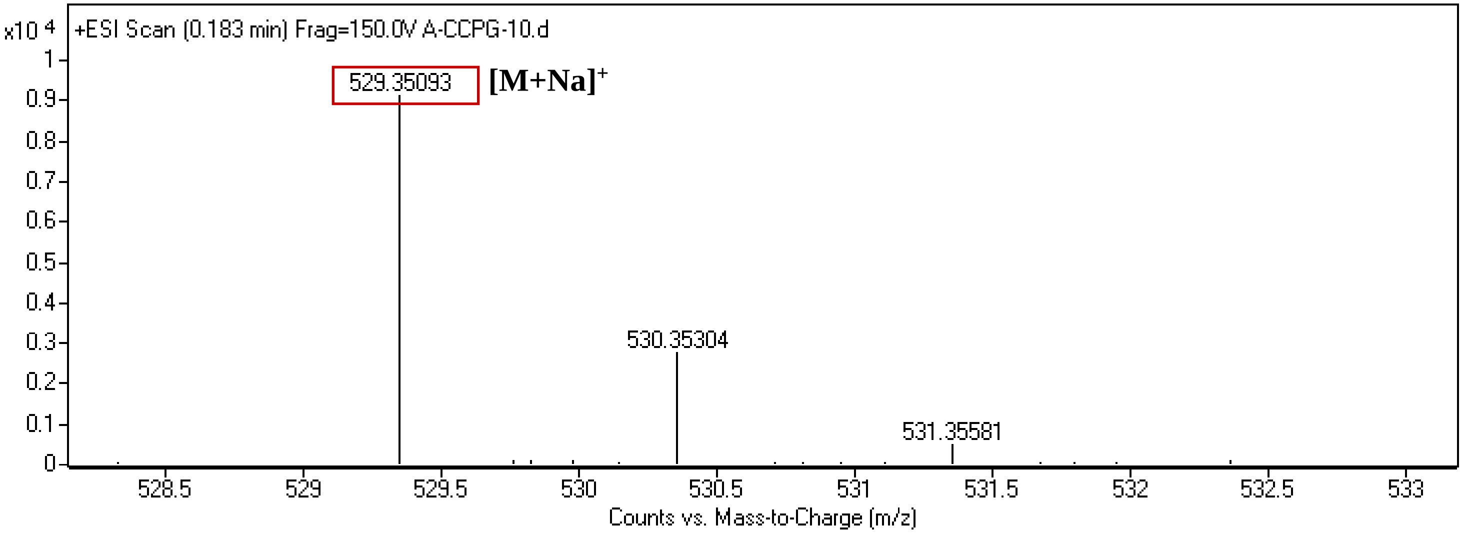

Figure S 50 HR-ESI-MS spectrum of compound **6**

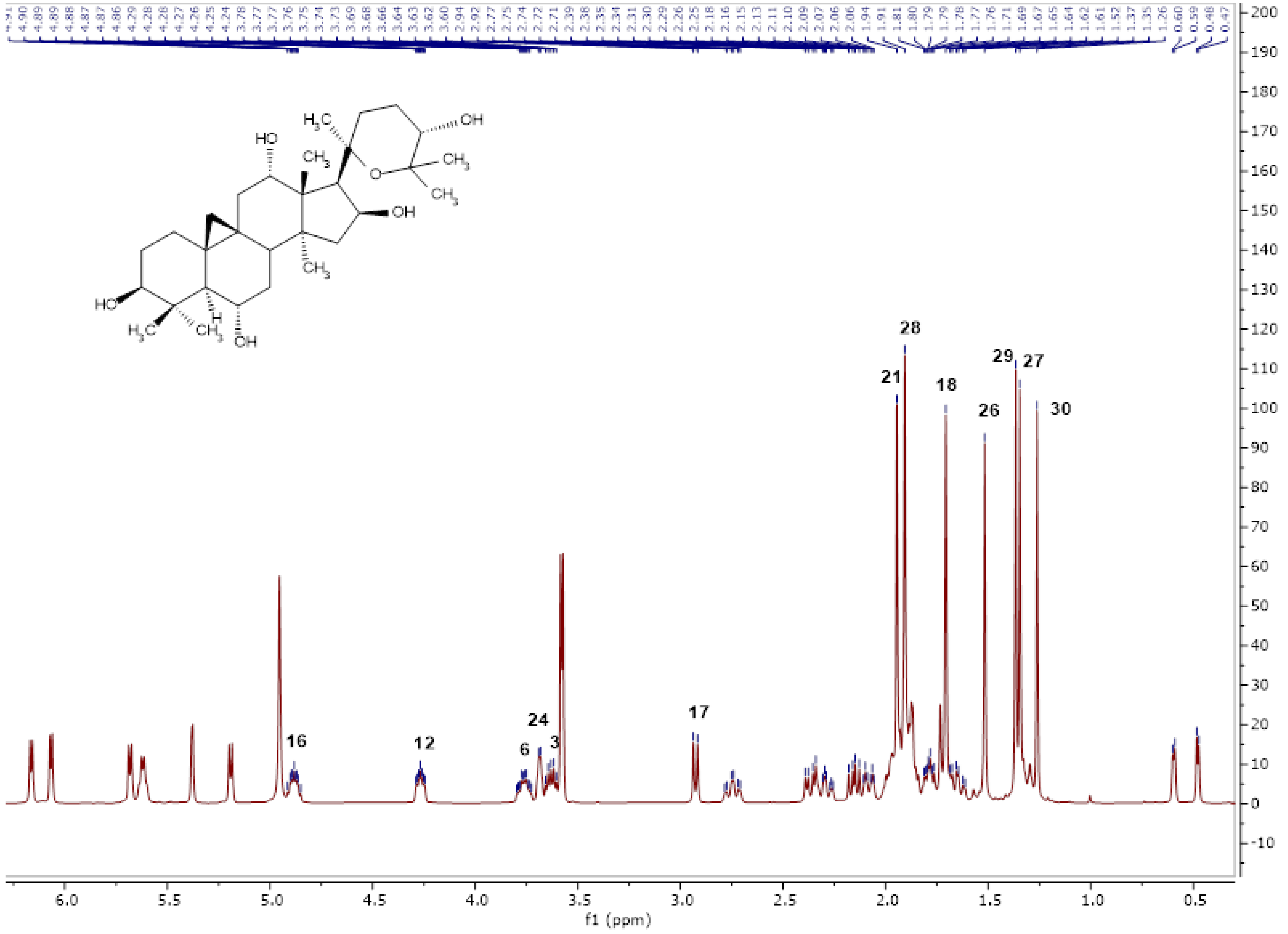

Figure S 51 ^{1}H-NMR spectrum of compound **6** (400 MHz, C_5D_5N)

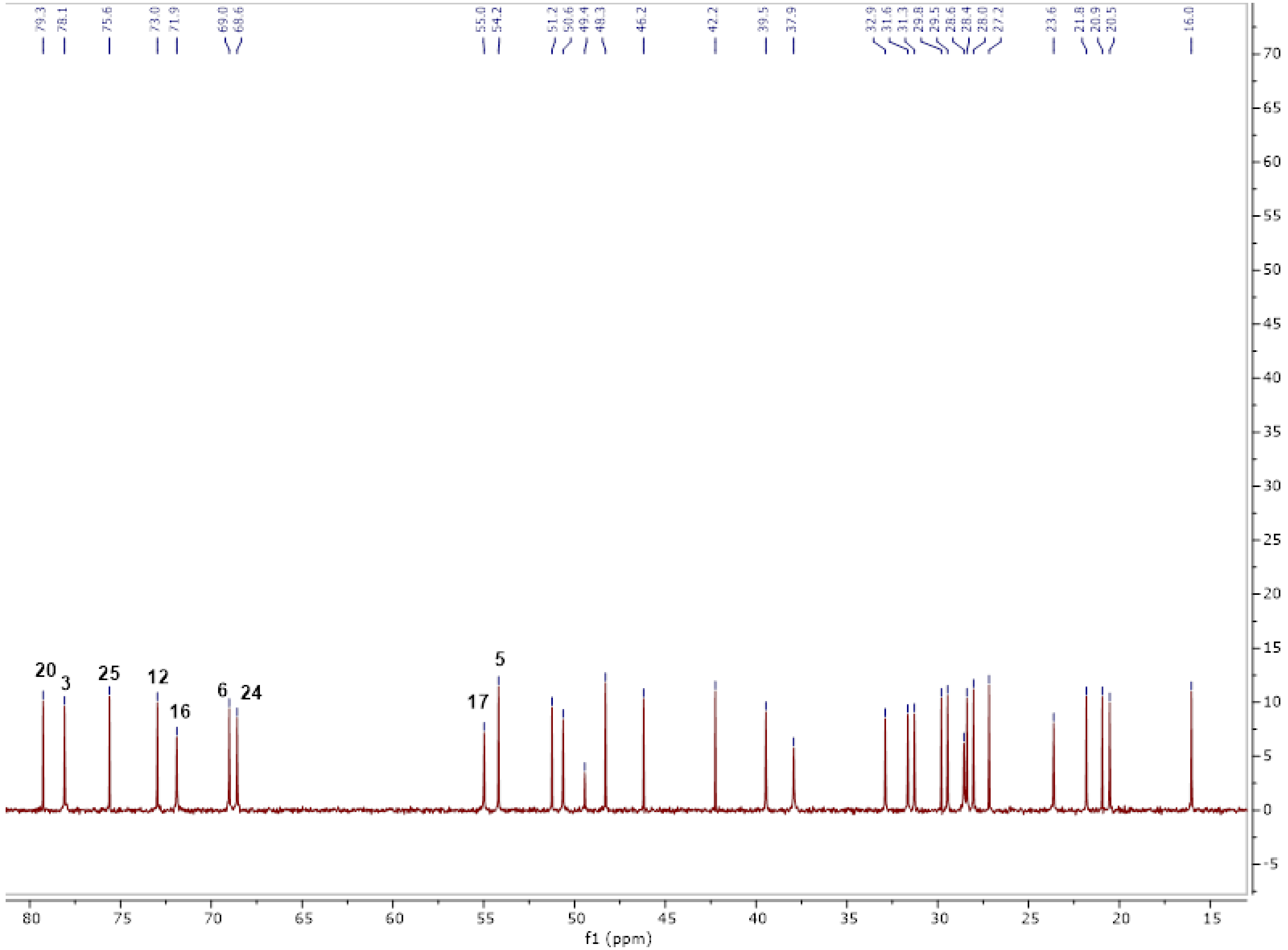

Figure S 52 ^{13}C-NMR spectrum of compound **6** (100 MHz, C_5D_5N)

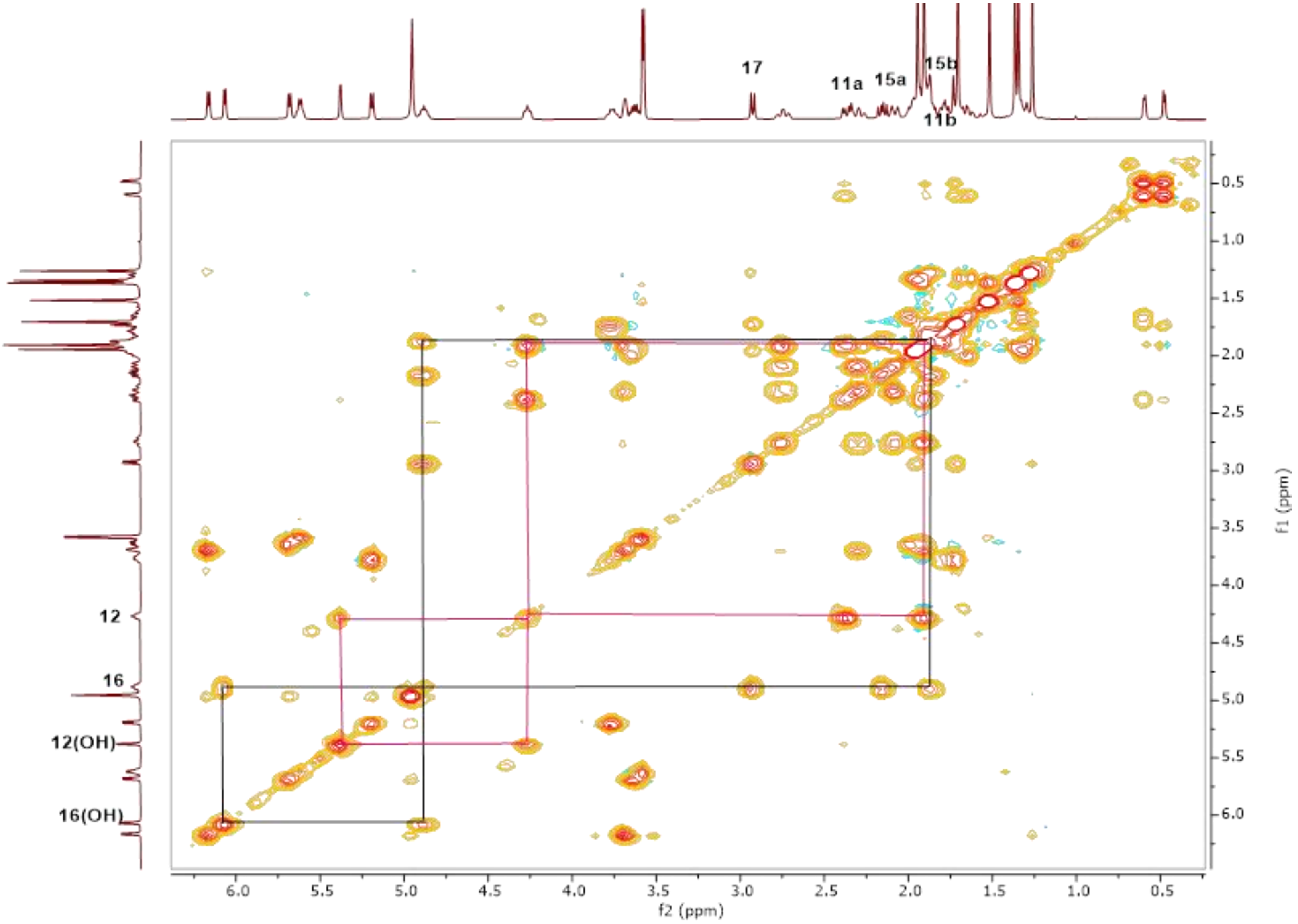

Figure S 53 COSY spectrum of compound **6**

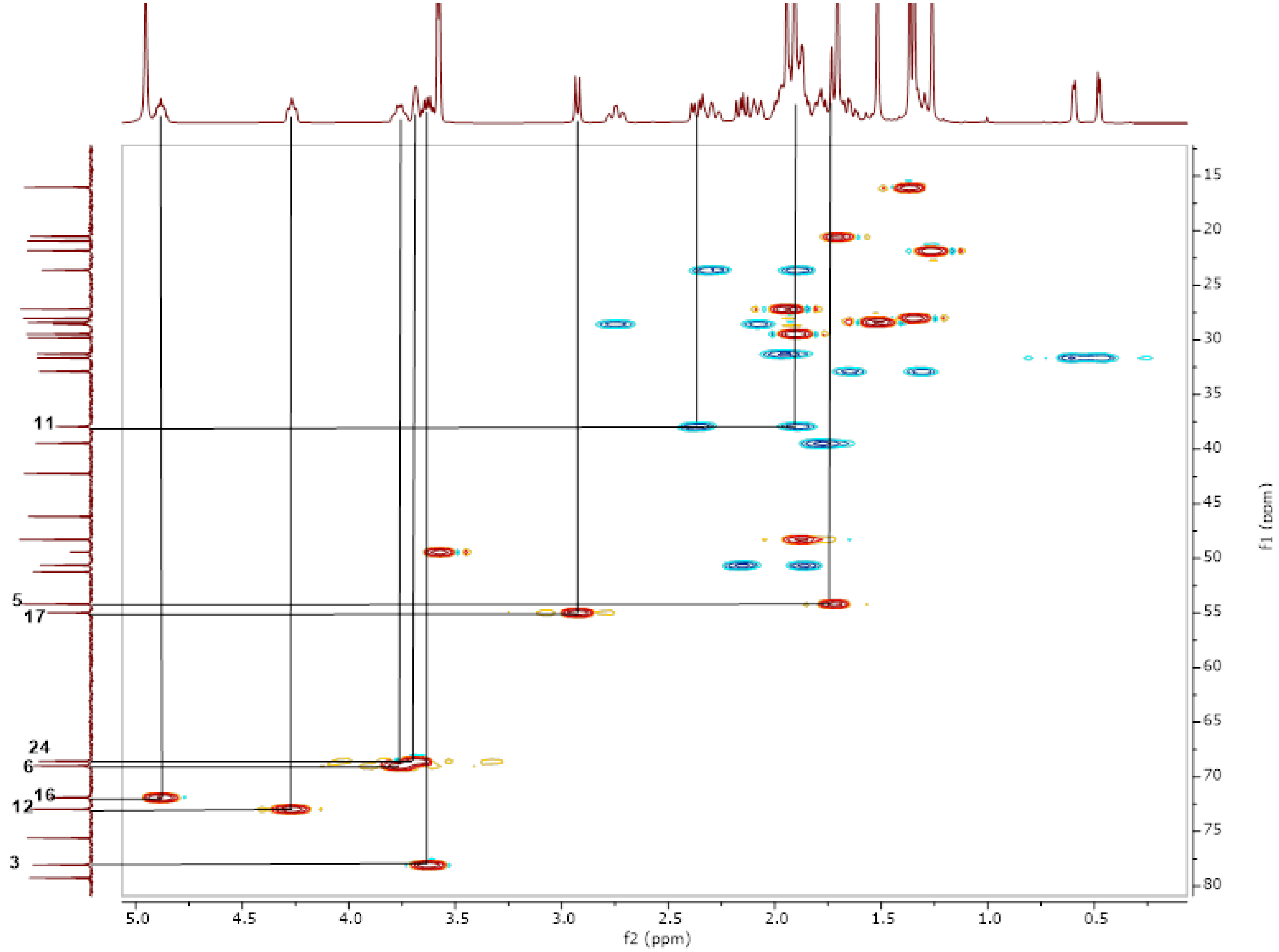

Figure S 54 HSQC spectrum of compound **6**

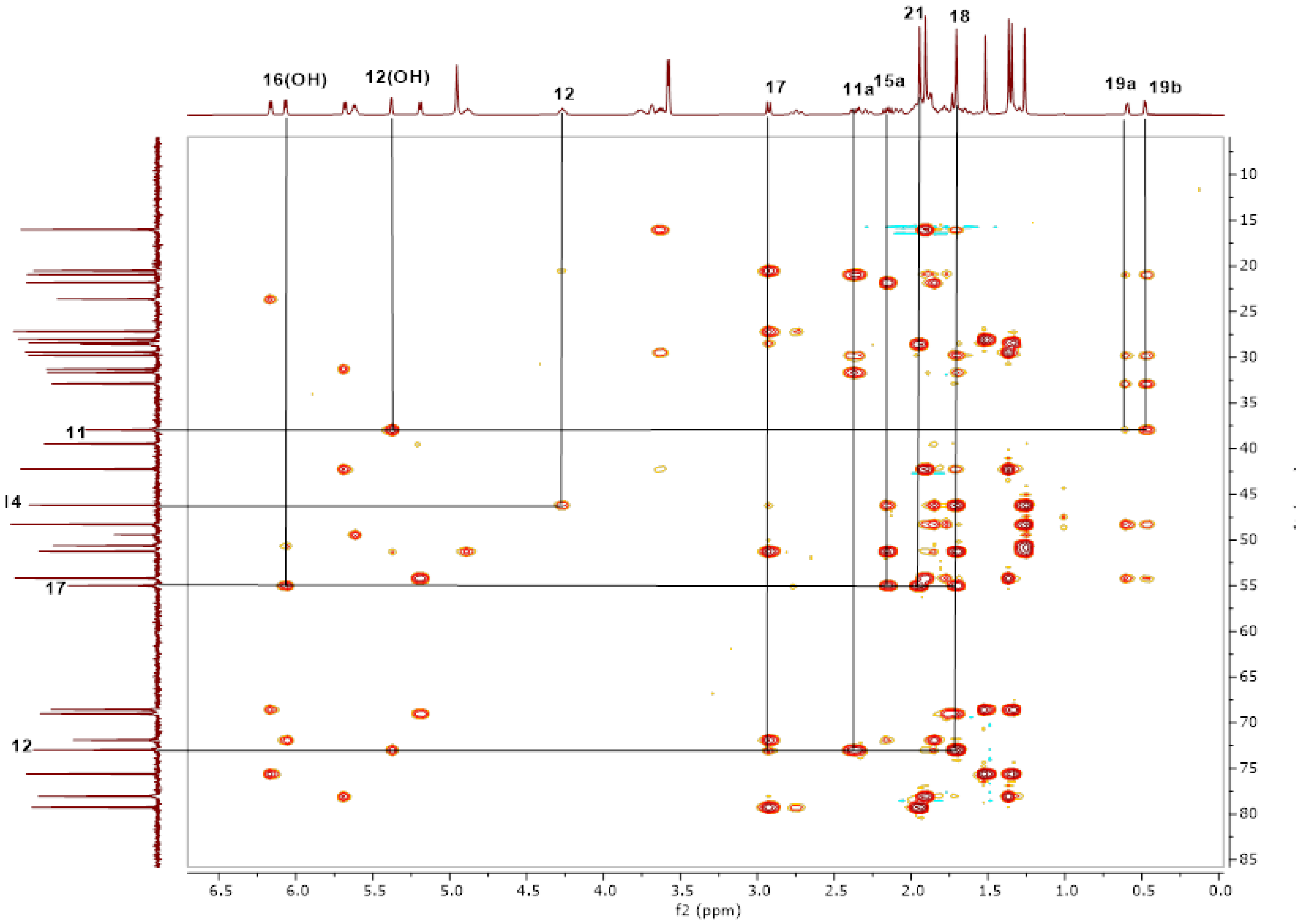

Figure S 55 HMBC correlations of compound **6**

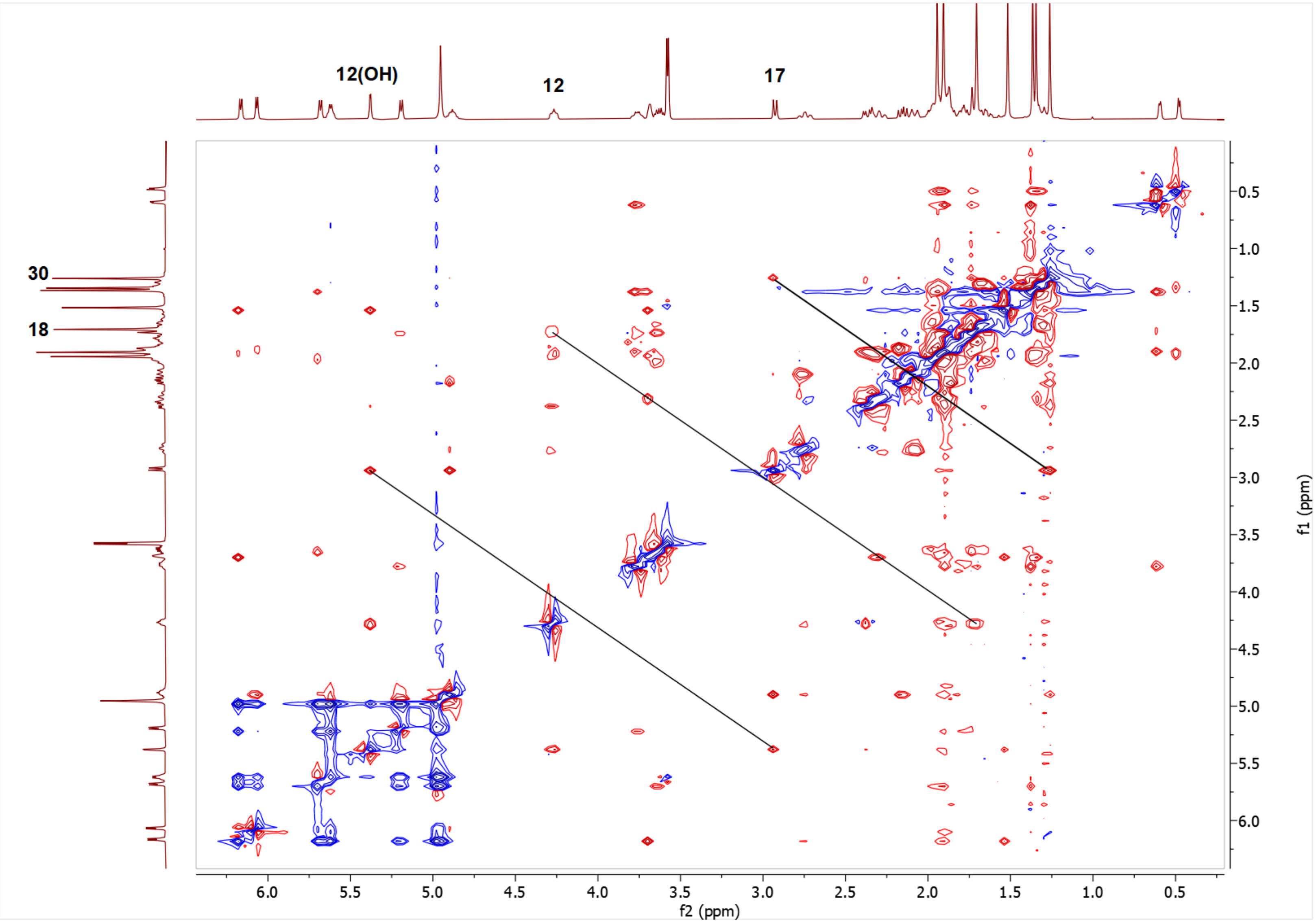

Figure S 56 NOESY spectrum of compound **6**

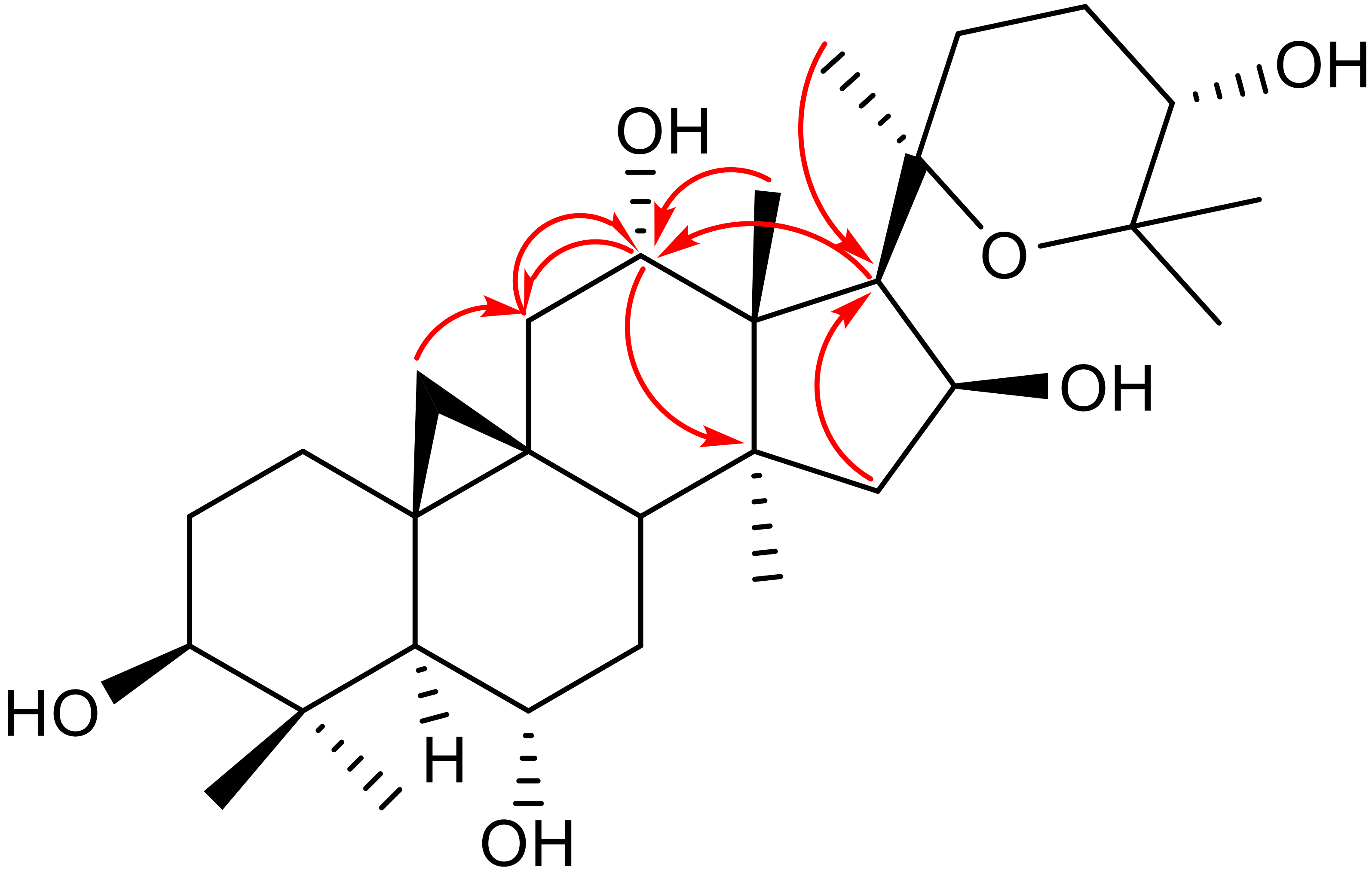

Figure S 57 Key HMBC correlations of compound **6** (arrows from H to C)

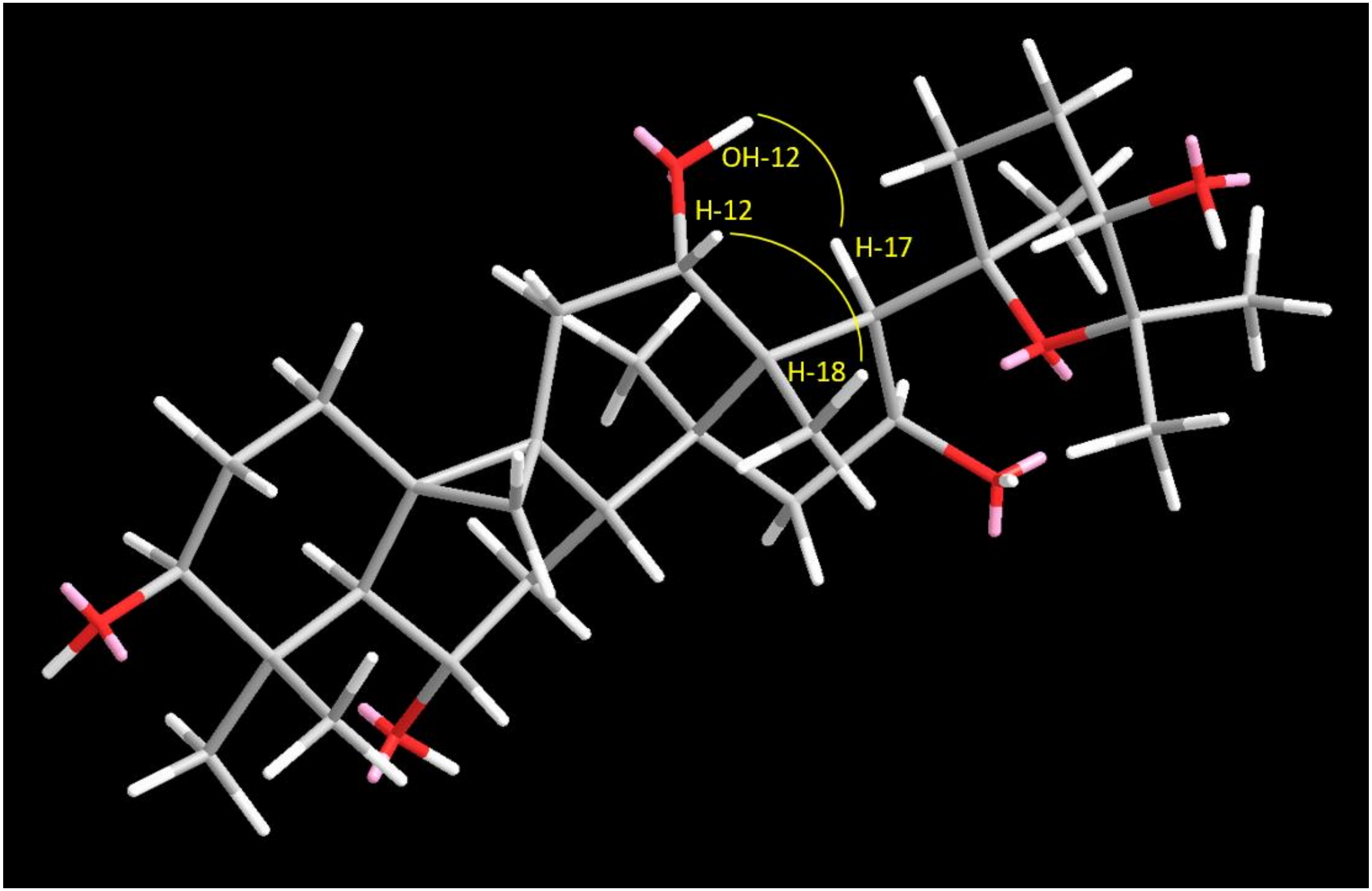

Figure S 58 Key NOE correlations of compound **6**

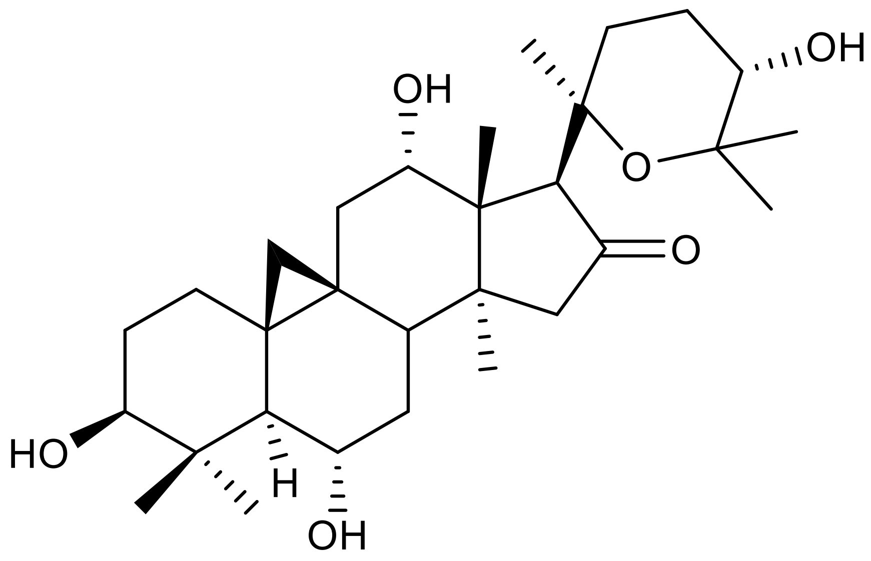

Figure S 59 Structure of compound **7**

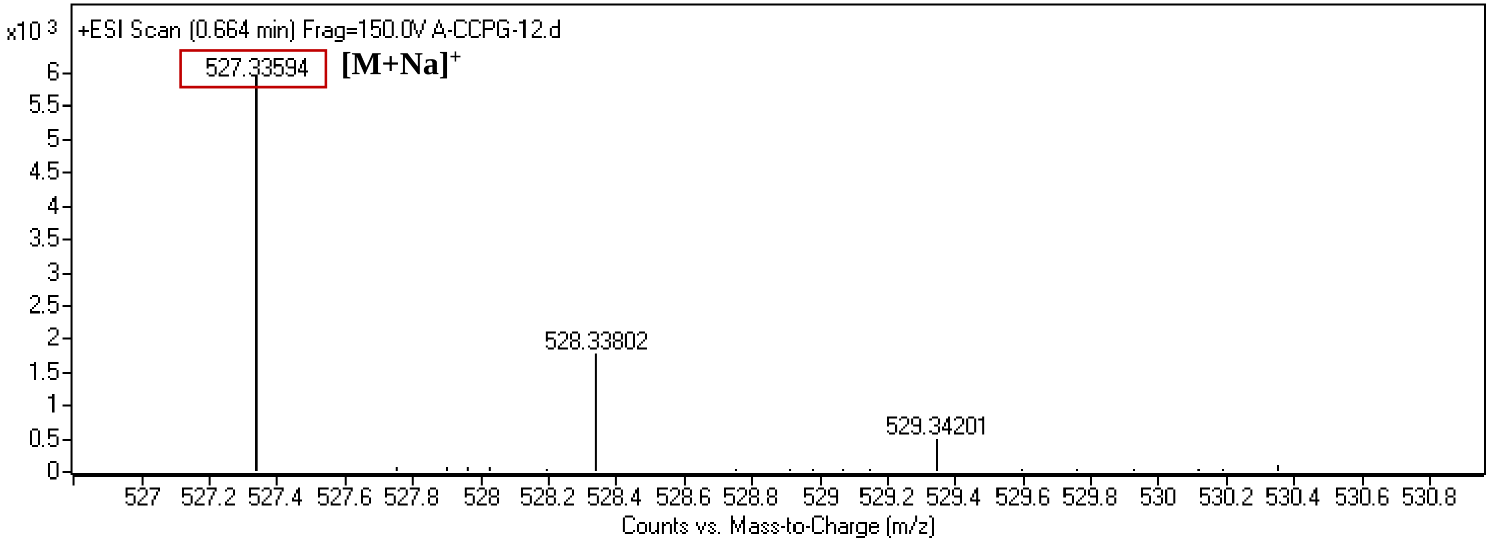

Figure S 60 HR-ESI-MS spectrum of compound **7**

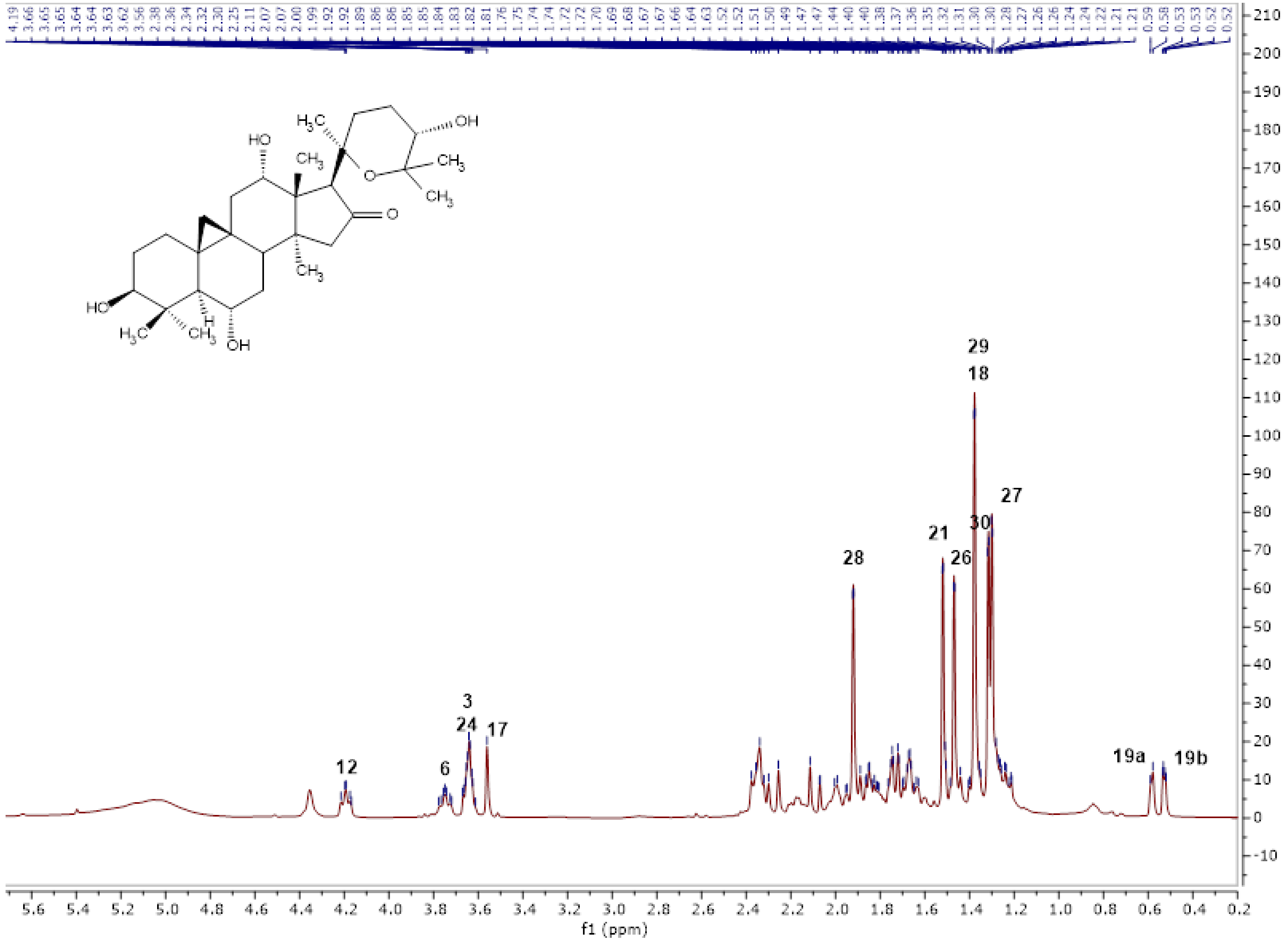

Figure S 61 ^{1}H-NMR spectrum of compound **7** (400 MHz, C_5D_5N)

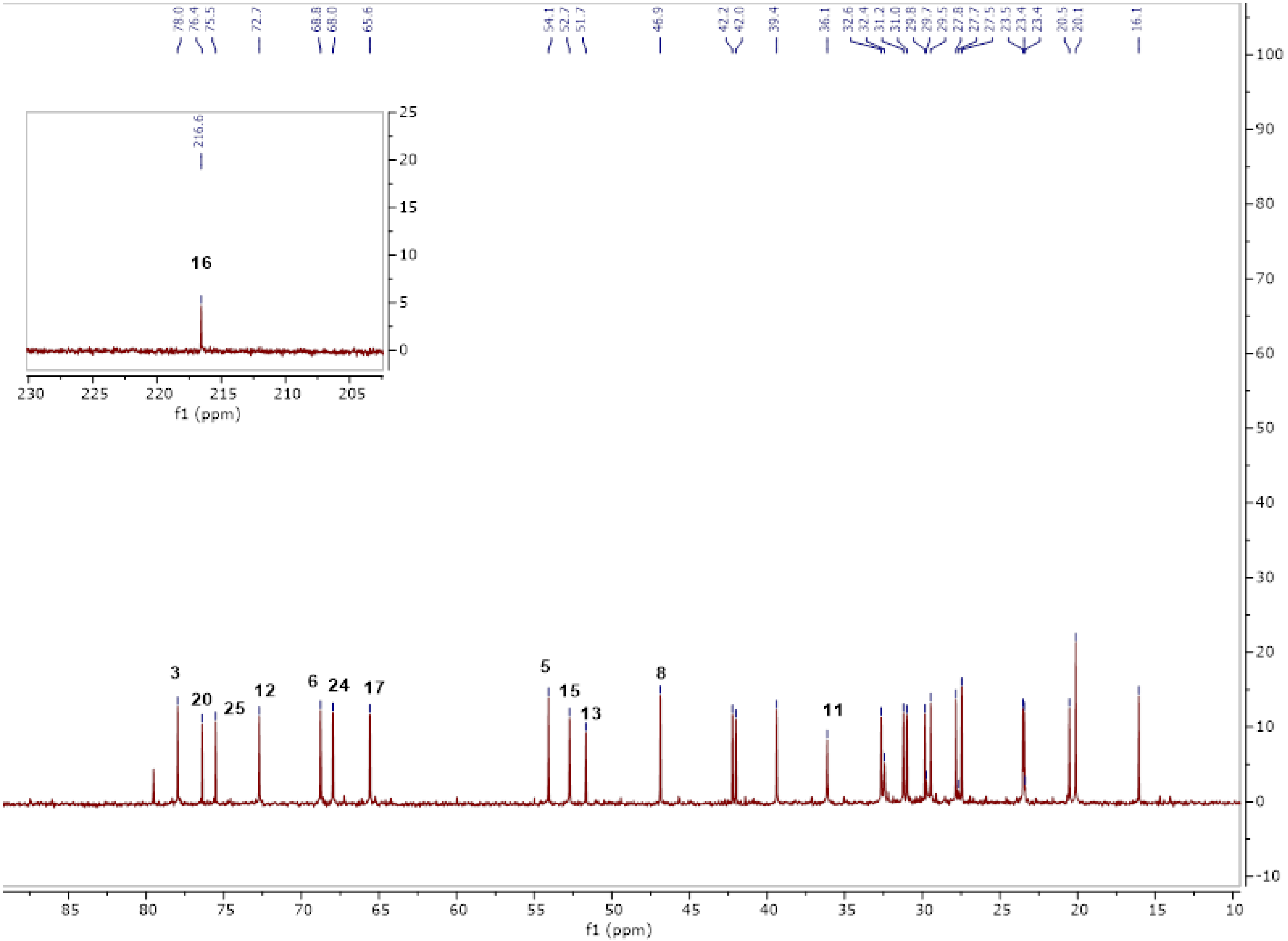

Figure S 62 ^{13}C-NMR spectrum of compound **7** (100 MHz, C_5D_5N)

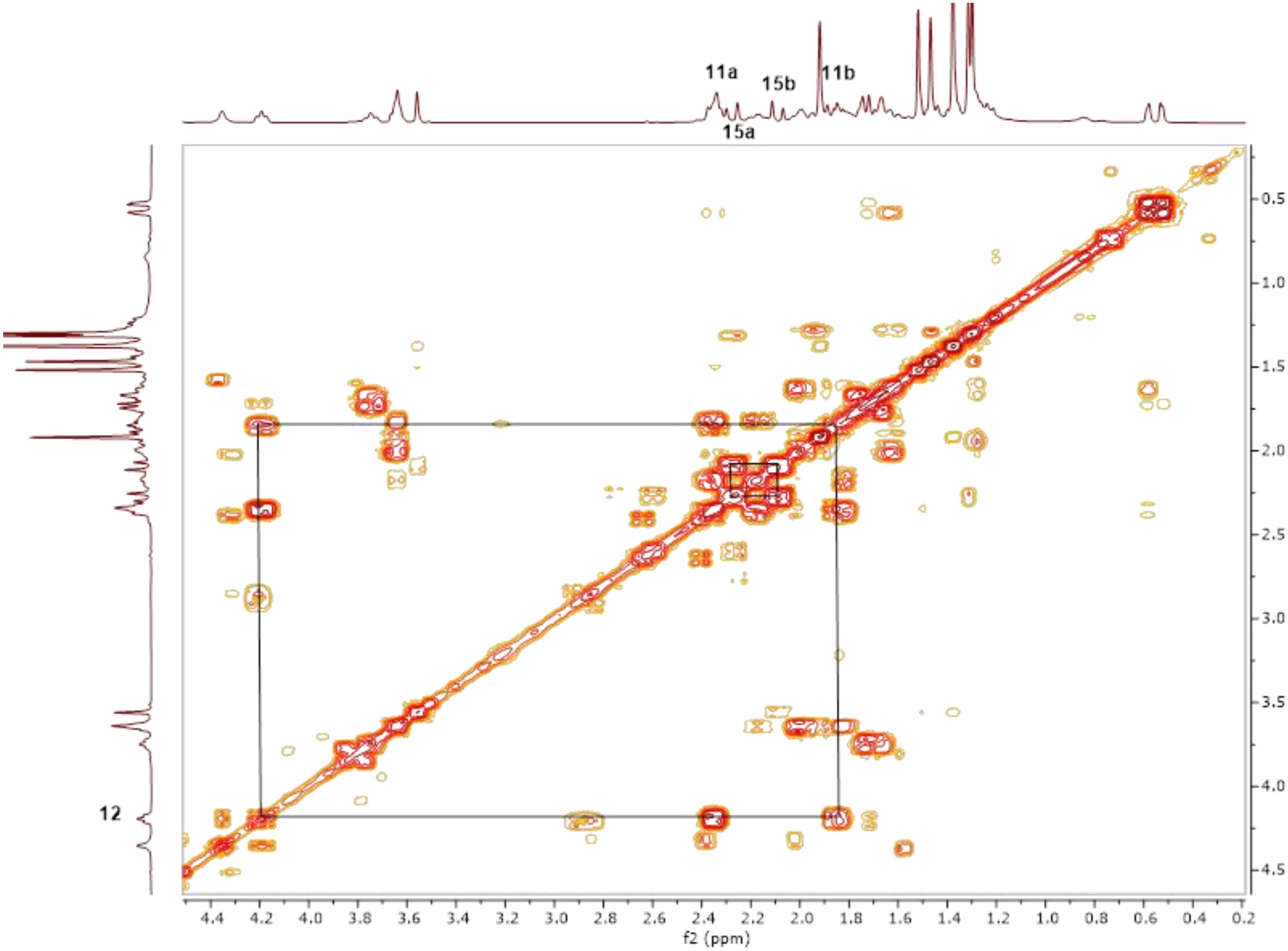

Figure S 63 COSY spectrum of compound **7**

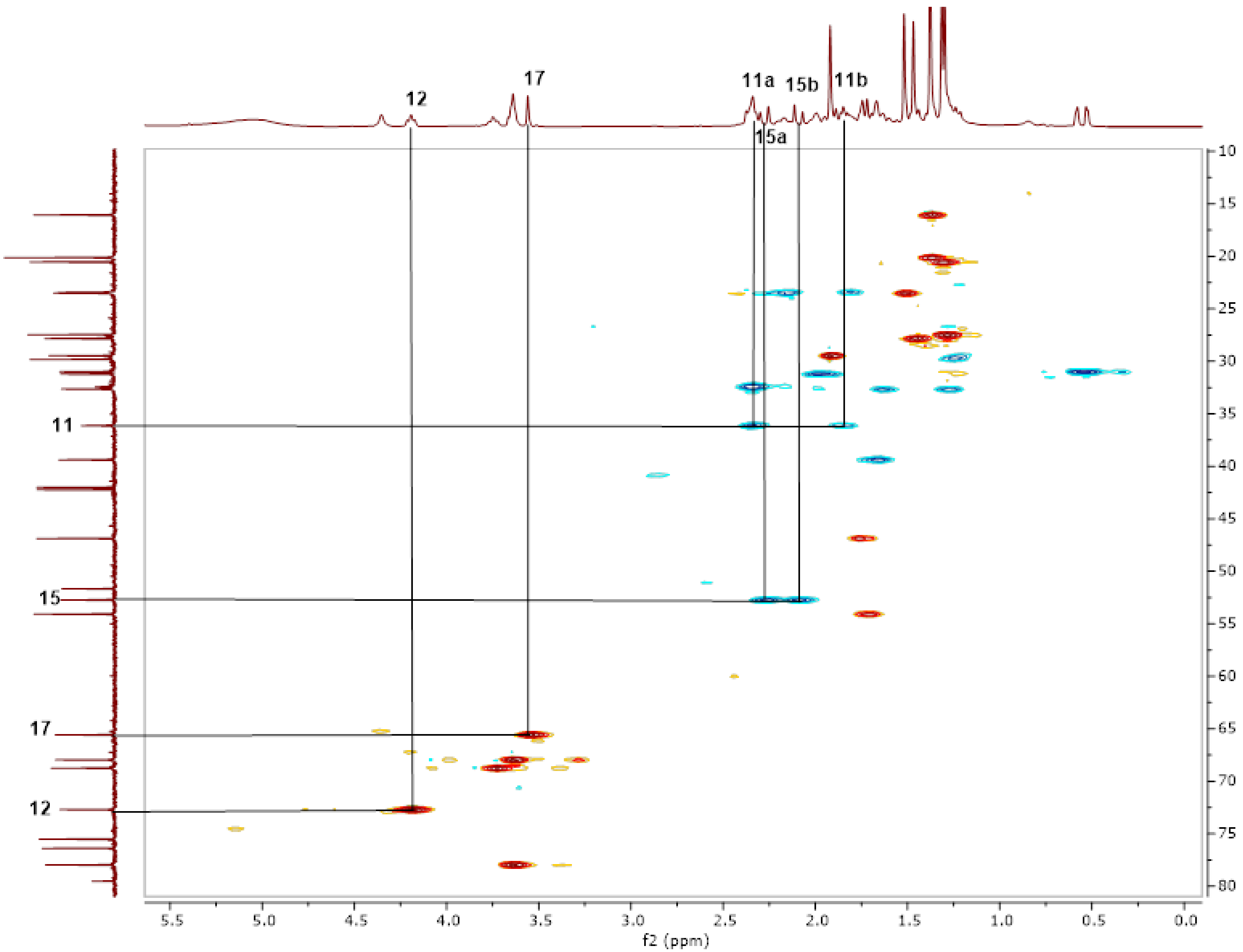

Figure S 64 HSQC spectrum of compound **7**

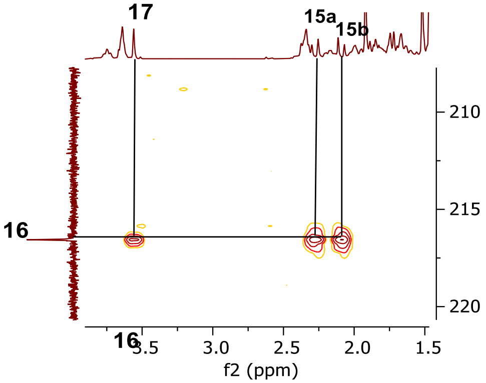

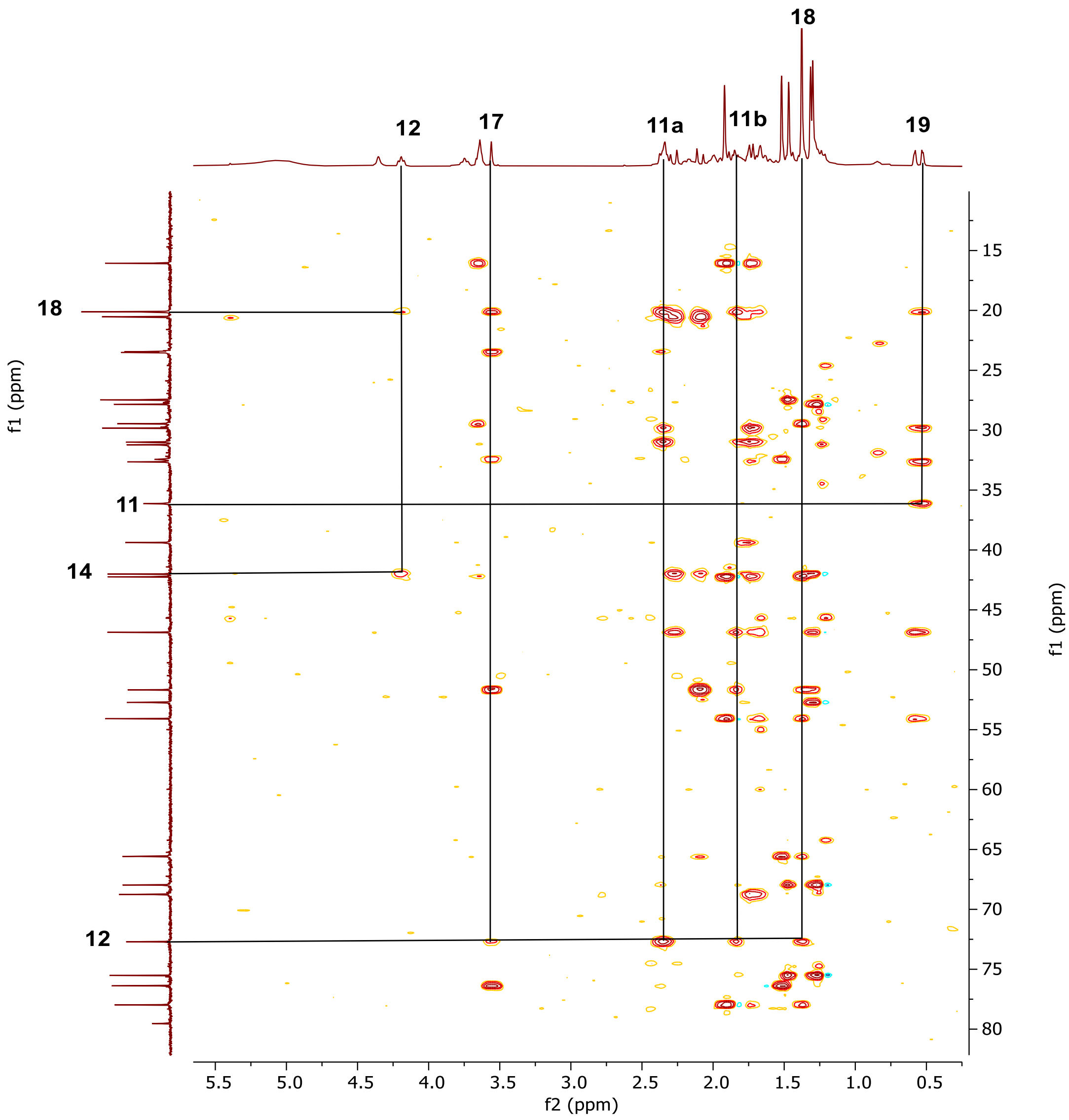

Figure S 65 HMBC spectrum of compound **7**

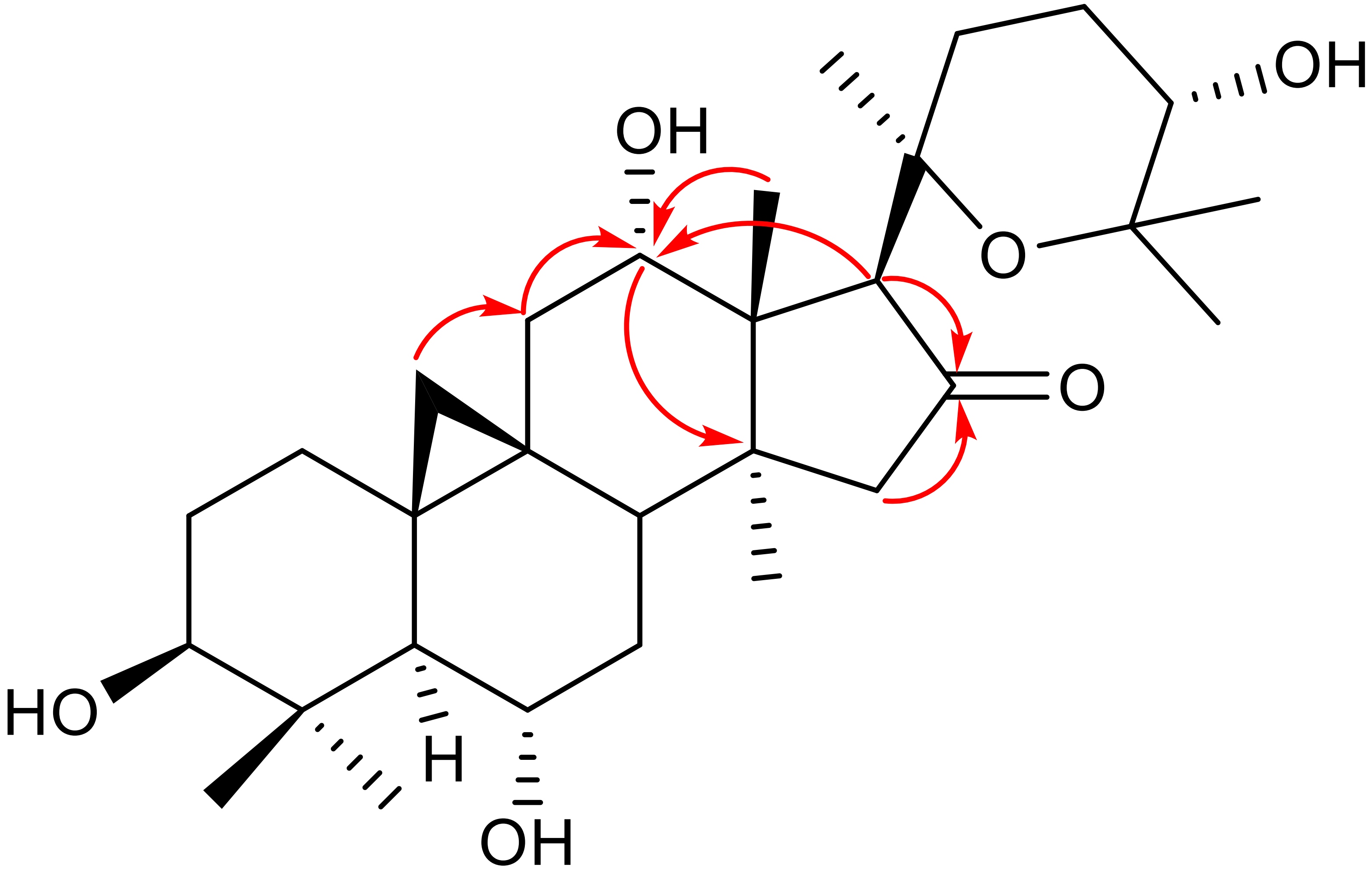

Figure S 66 Key HMBC correlations of compound **7** (arrows from H to C)

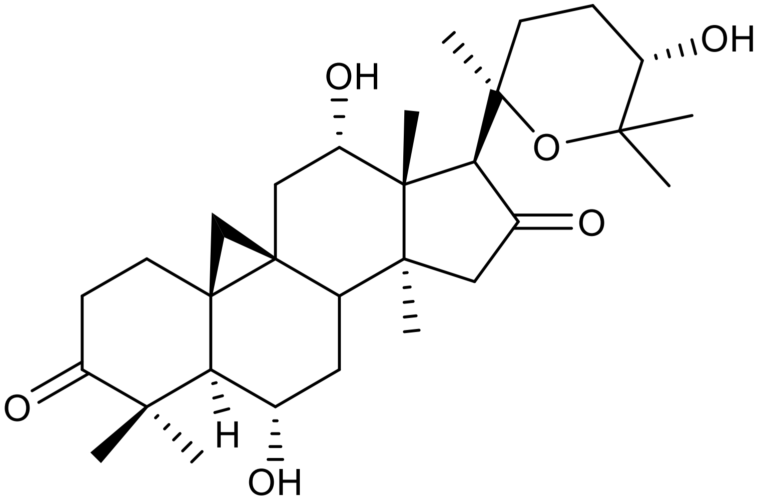

Figure S 67 Structure of compound **8**

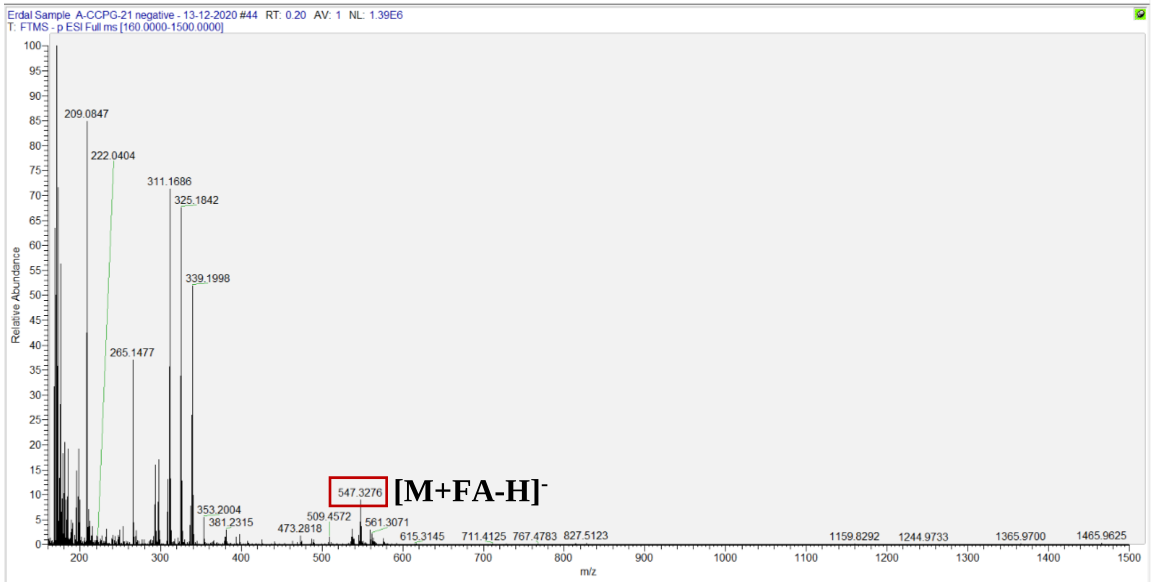

Figure S 68 HR-ESI-MS spectrum of compound **8**

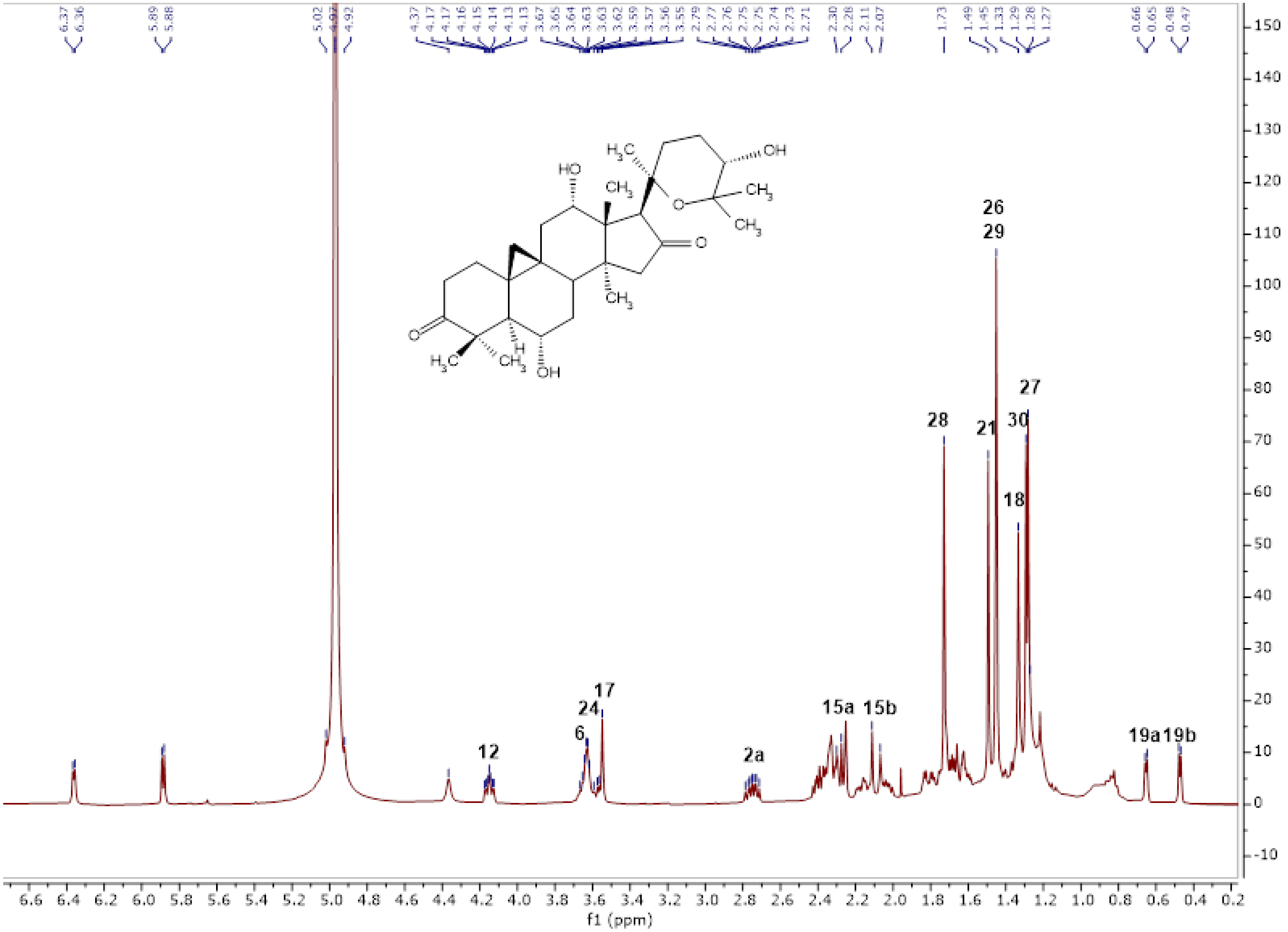

Figure S 69 ^{1}H-NMR spectrum of compound **8** (400 MHz, C_5D_5N)

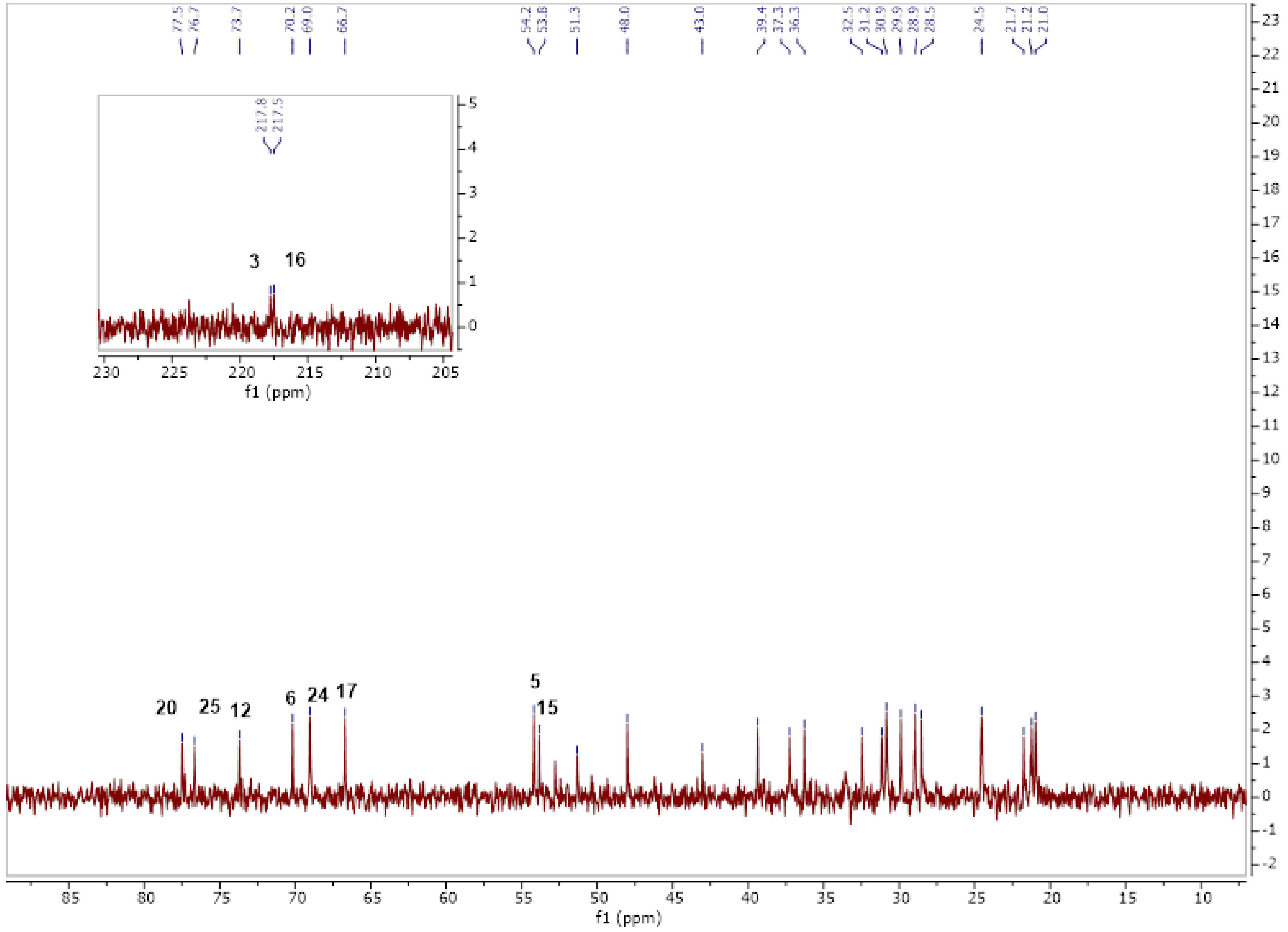

Figure S 70 ^{13}C-NMR spectrum of compound **8** (100 MHz, C_5D_5N)

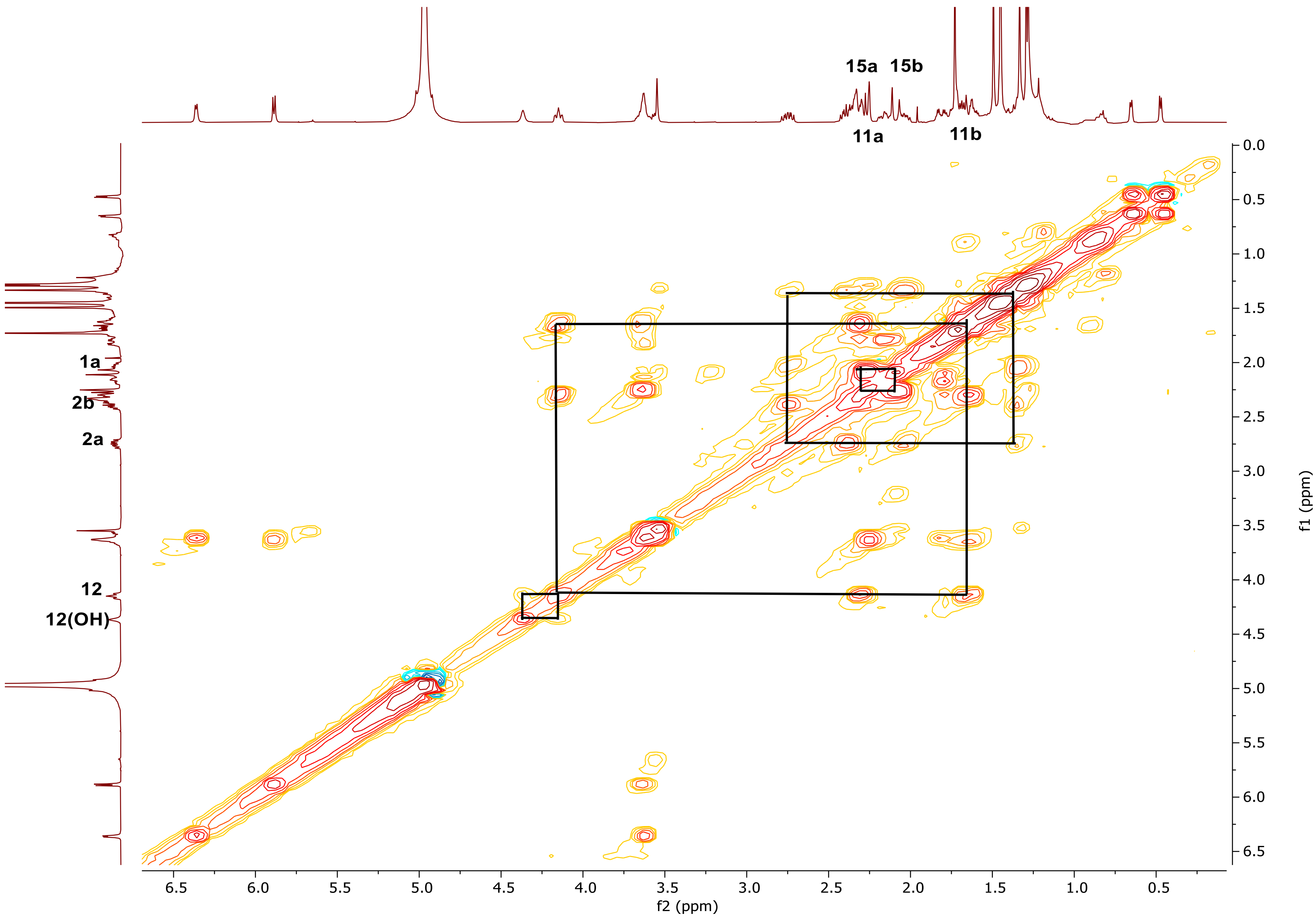

Figure S 71 COSY spectrum of compound **8**

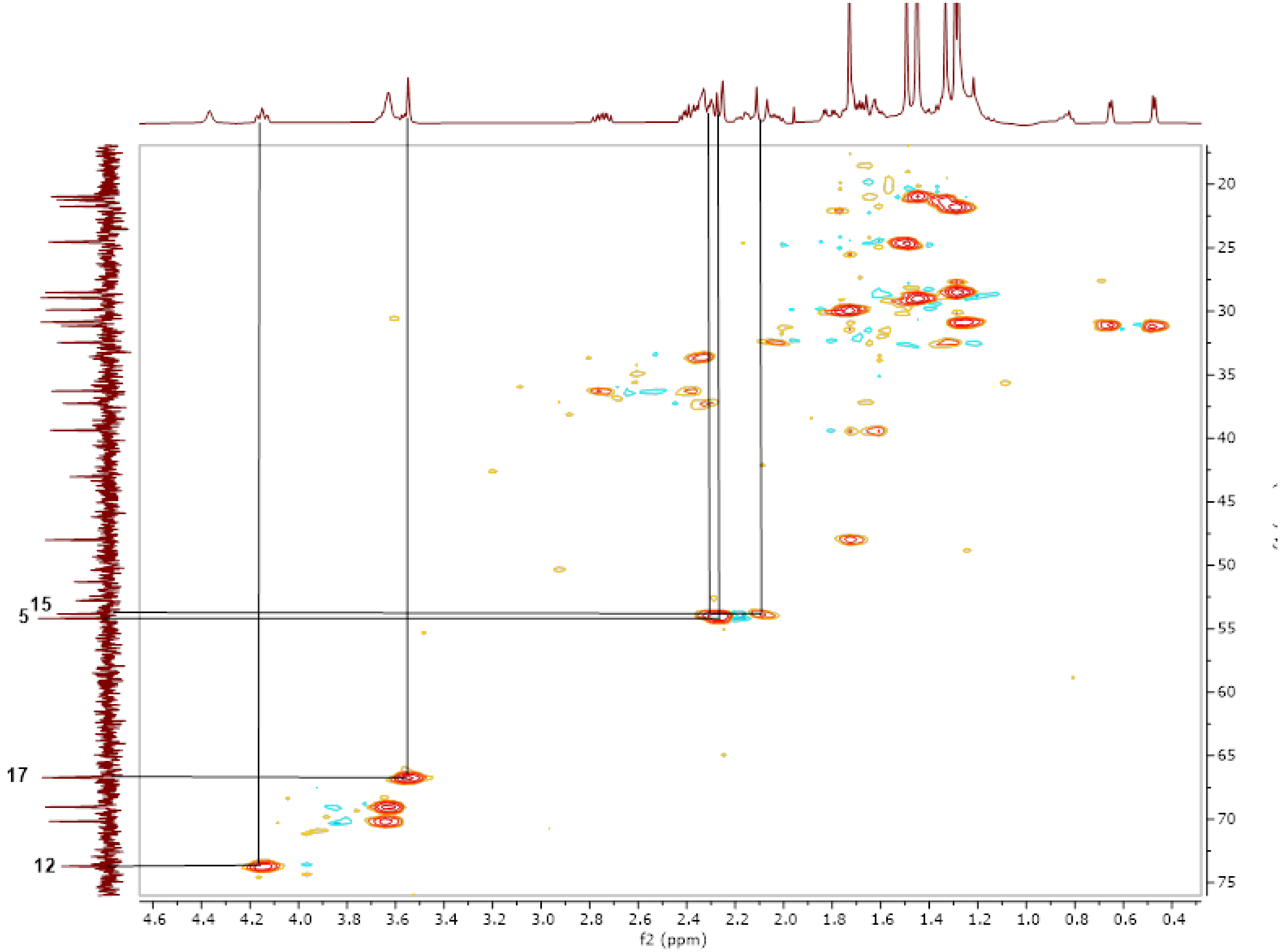

Figure S 72 HSQC spectrum of compound **8**

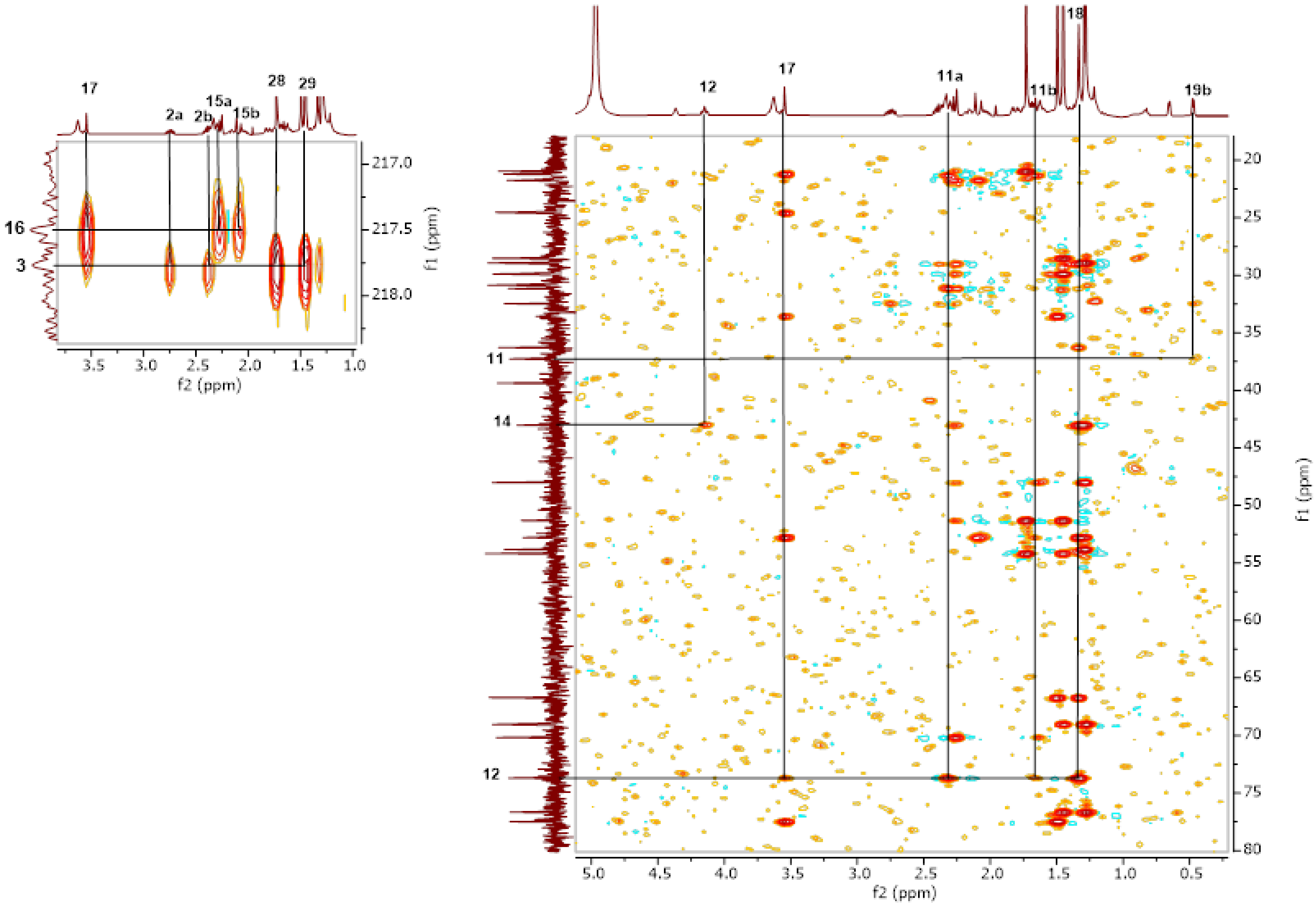

Figure S 73 HMBC spectrum of compound **8**

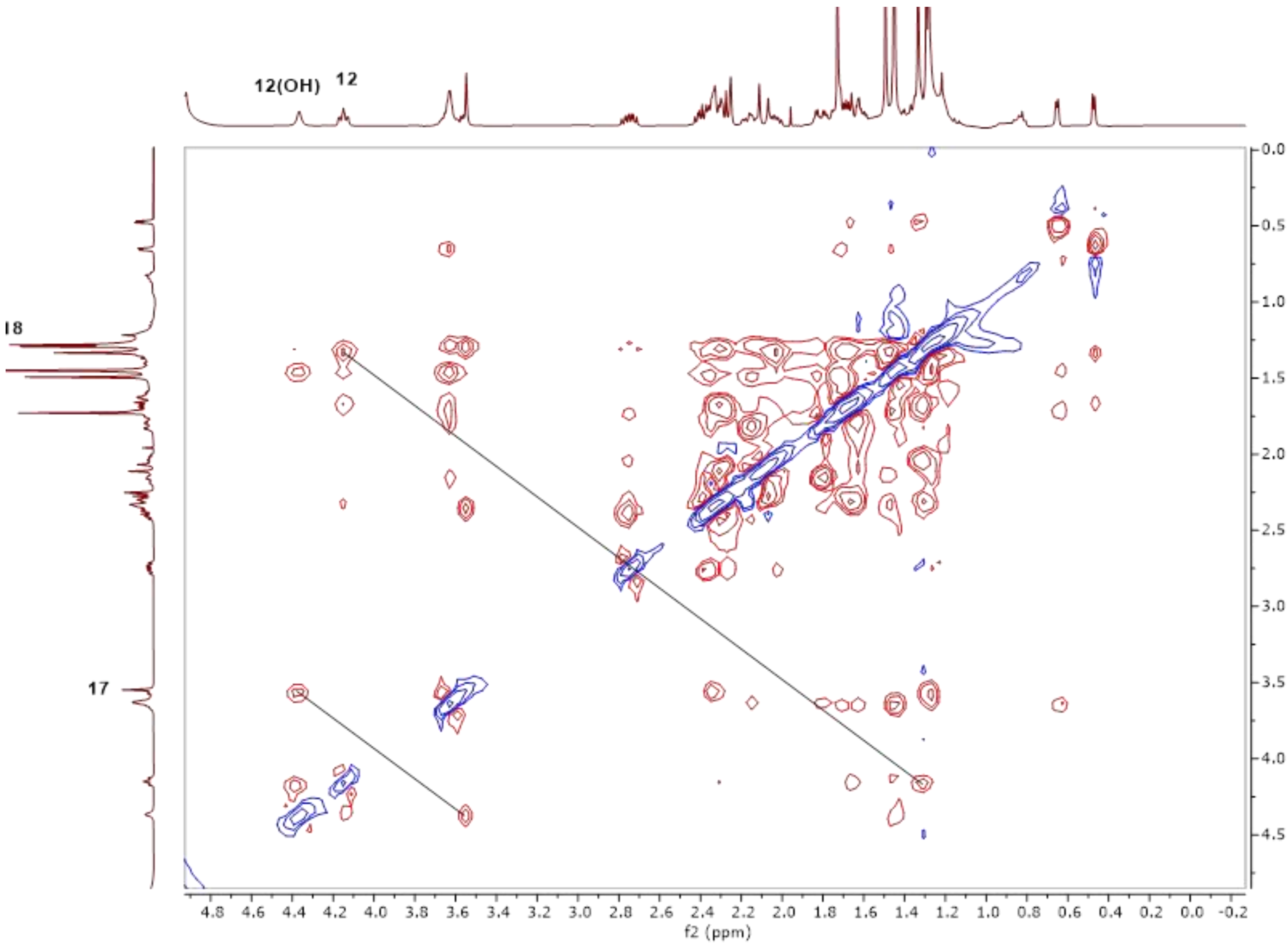

Figure S 74 NOESY spectrum of compound **8**

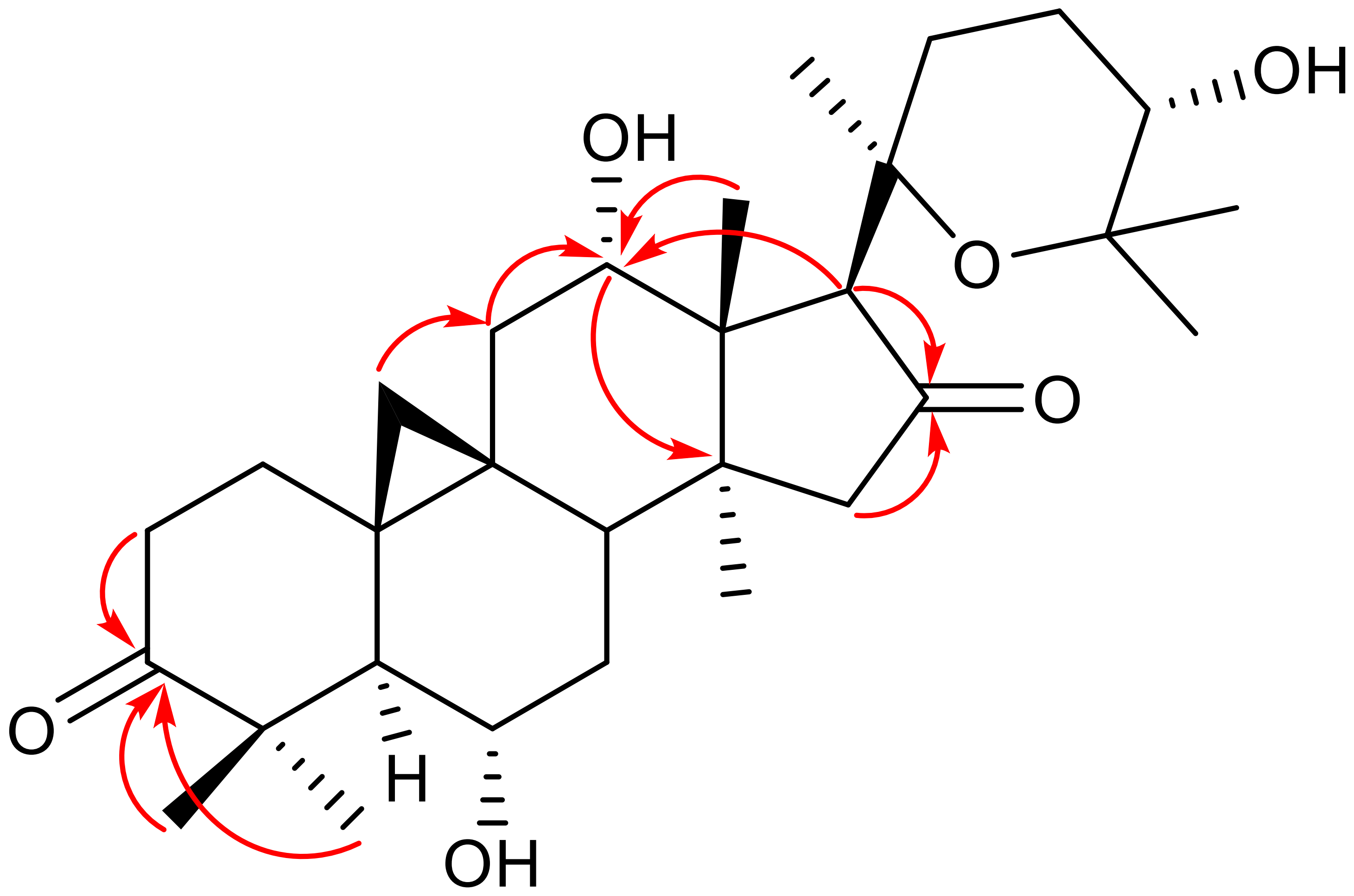

Figure S 75 Key HMBC correlations of compound **8** (arrows from H to C)

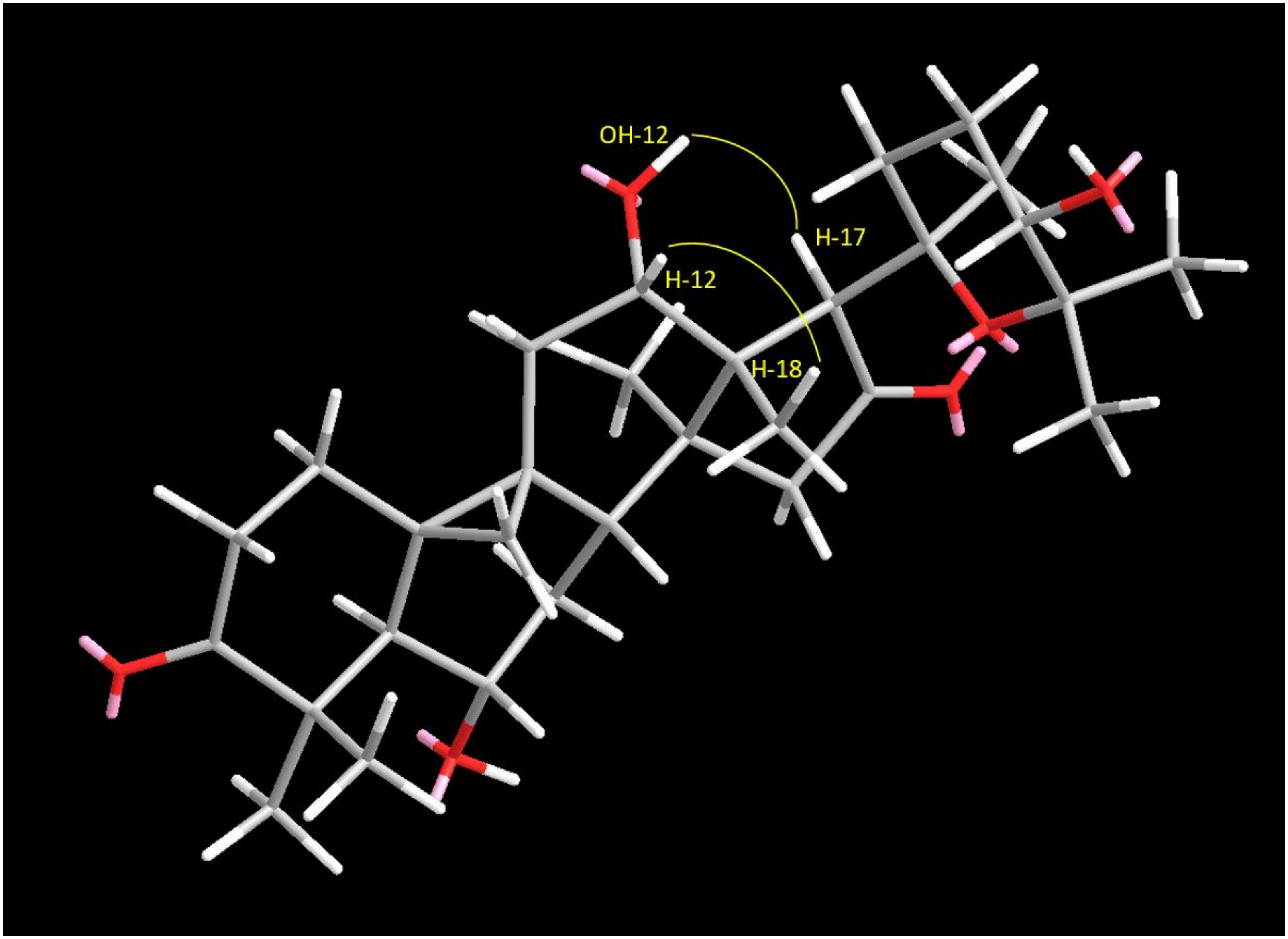

Figure S 76 Key NOE correlations of compound **8**

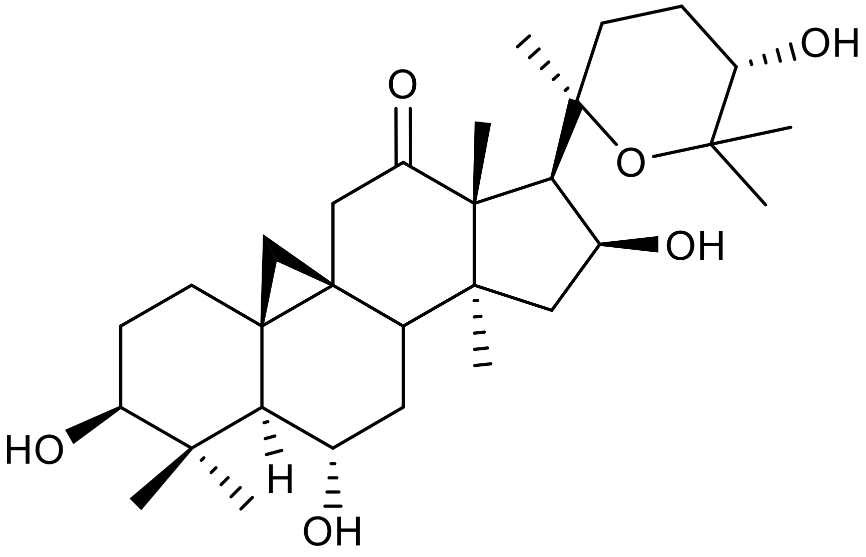

Figure S 77 Structure of compound **9**

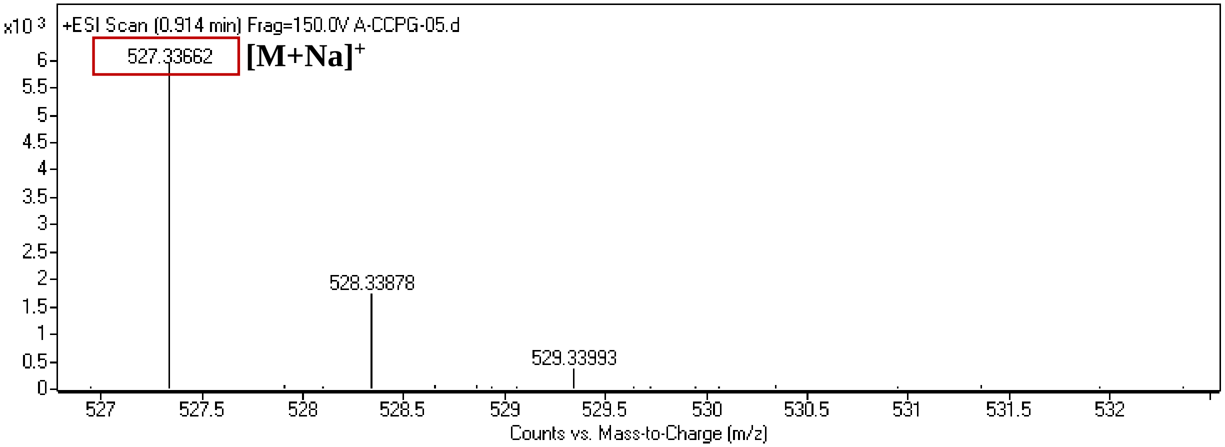

Figure S 78 HR-ESI-MS spectrum of compound **9**

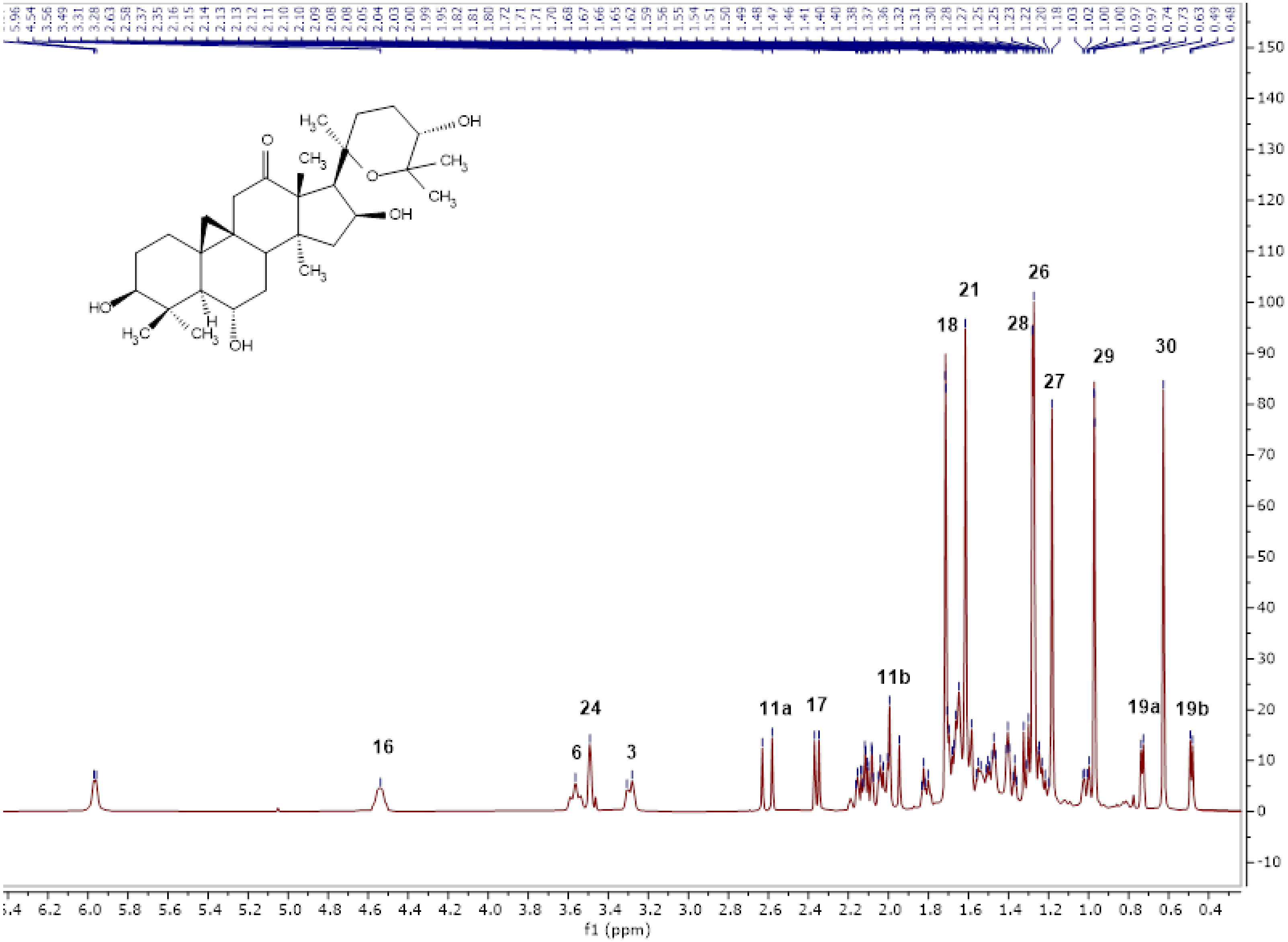

Figure S 79 ^{1}H-NMR spectrum of compound **9** (400 MHz, $CDCl_3$)

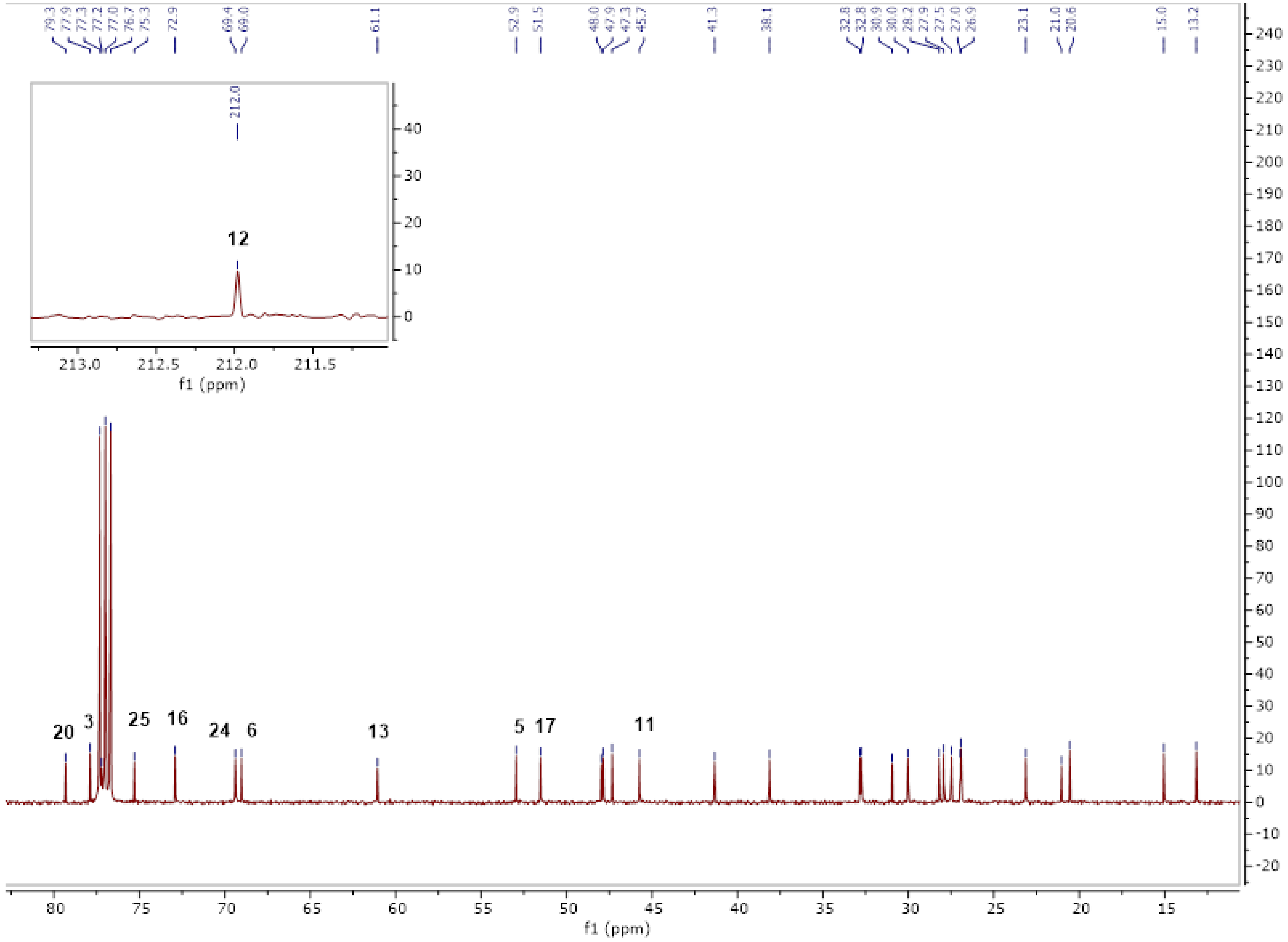

Figure S 80 ^{13}C-NMR spectrum of compound **9** (100 MHz, $CDCl_3$)

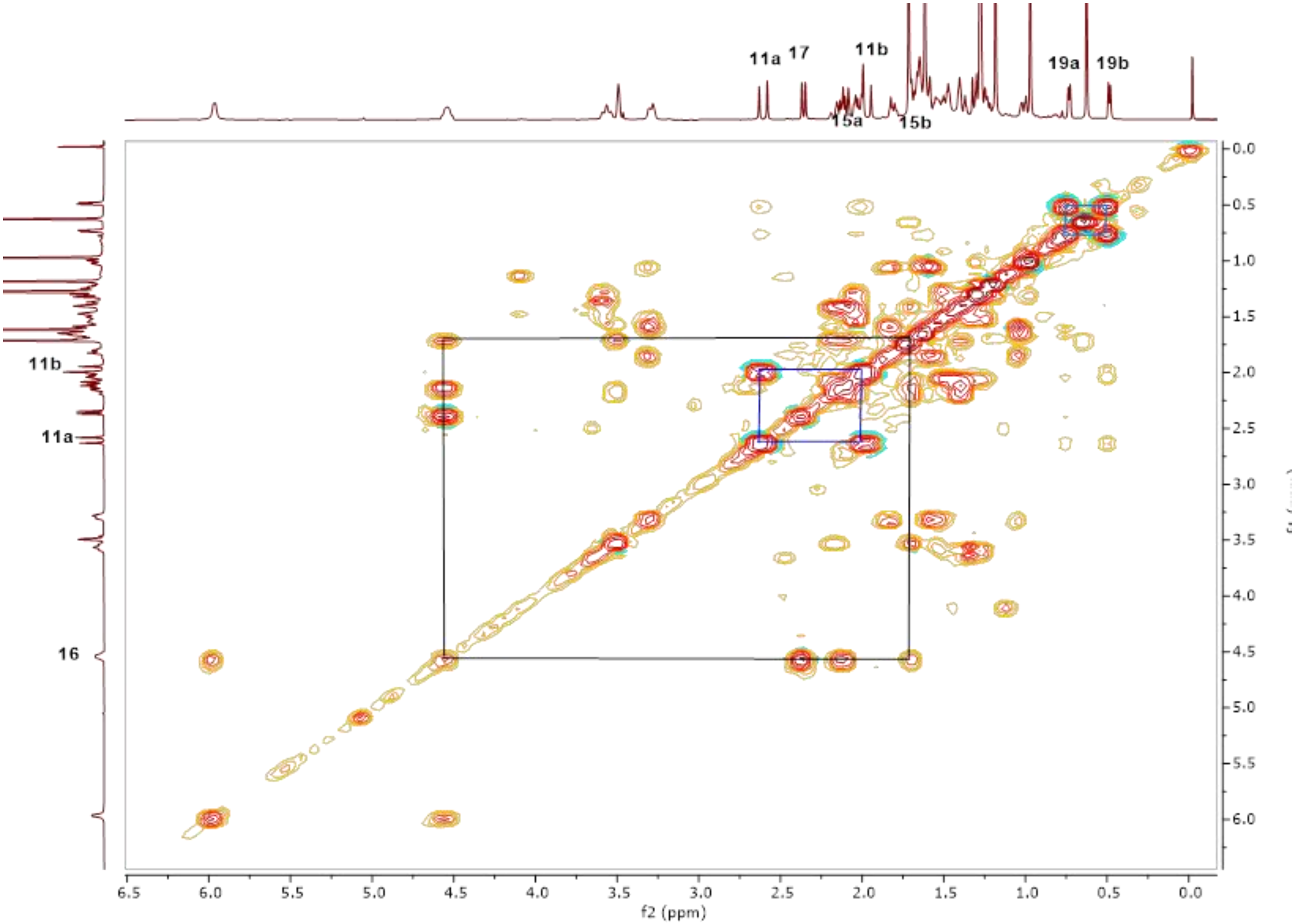

Figure S 81 COSY spectrum of compound **9**

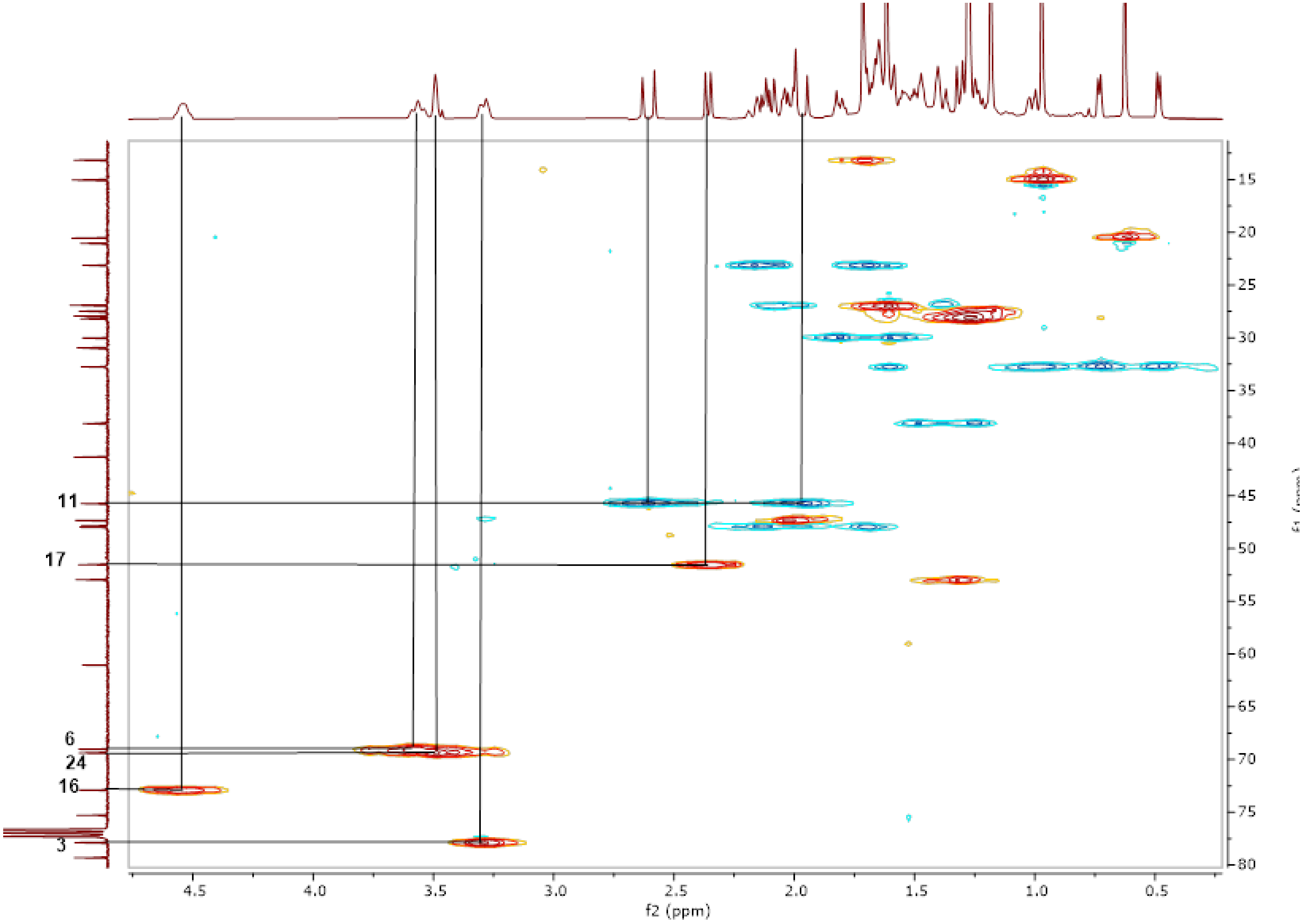

Figure S 82 HSQC spectrum of compound **9**

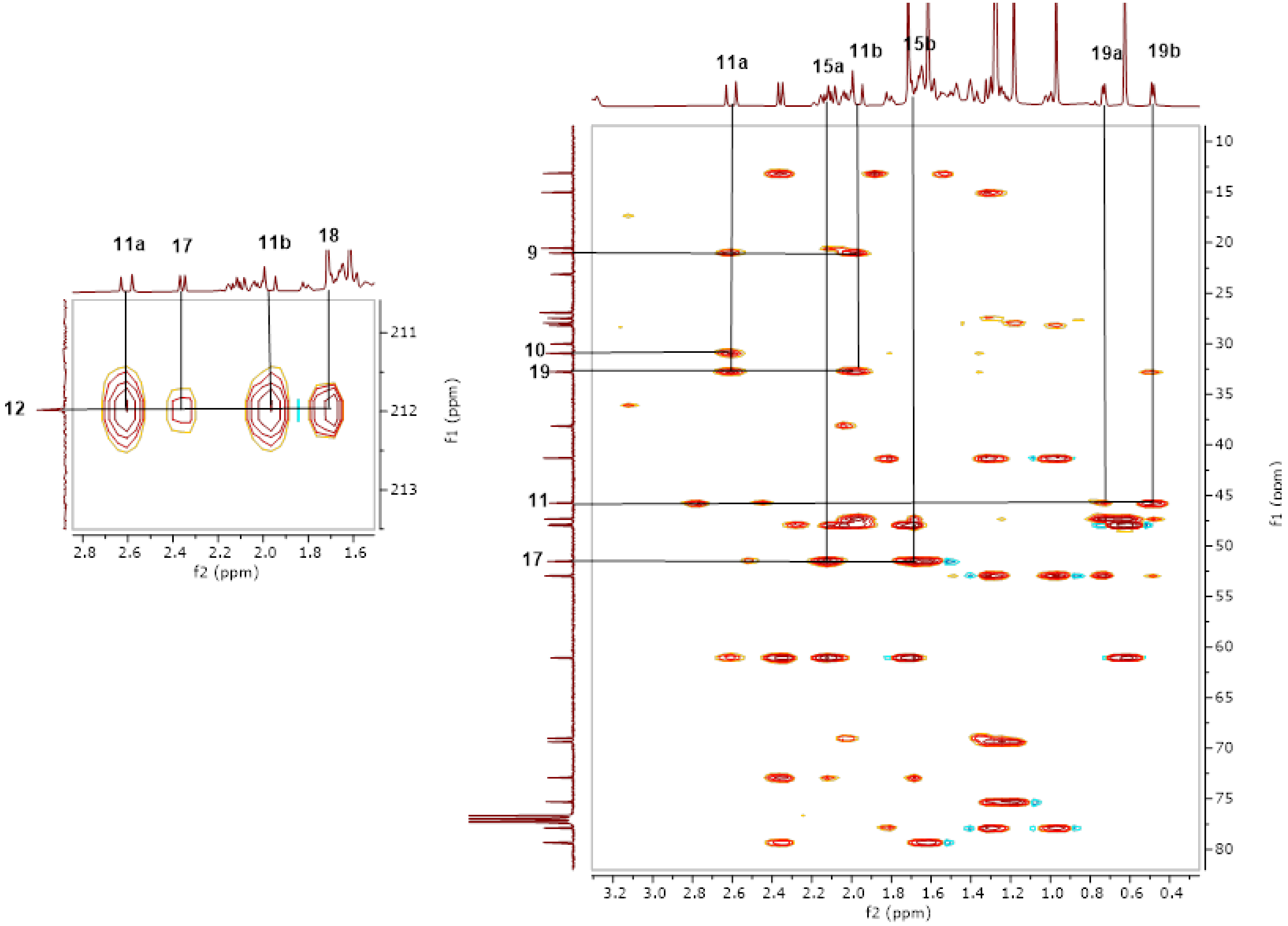

Figure S 83 HMBC spectrum of compound **9**

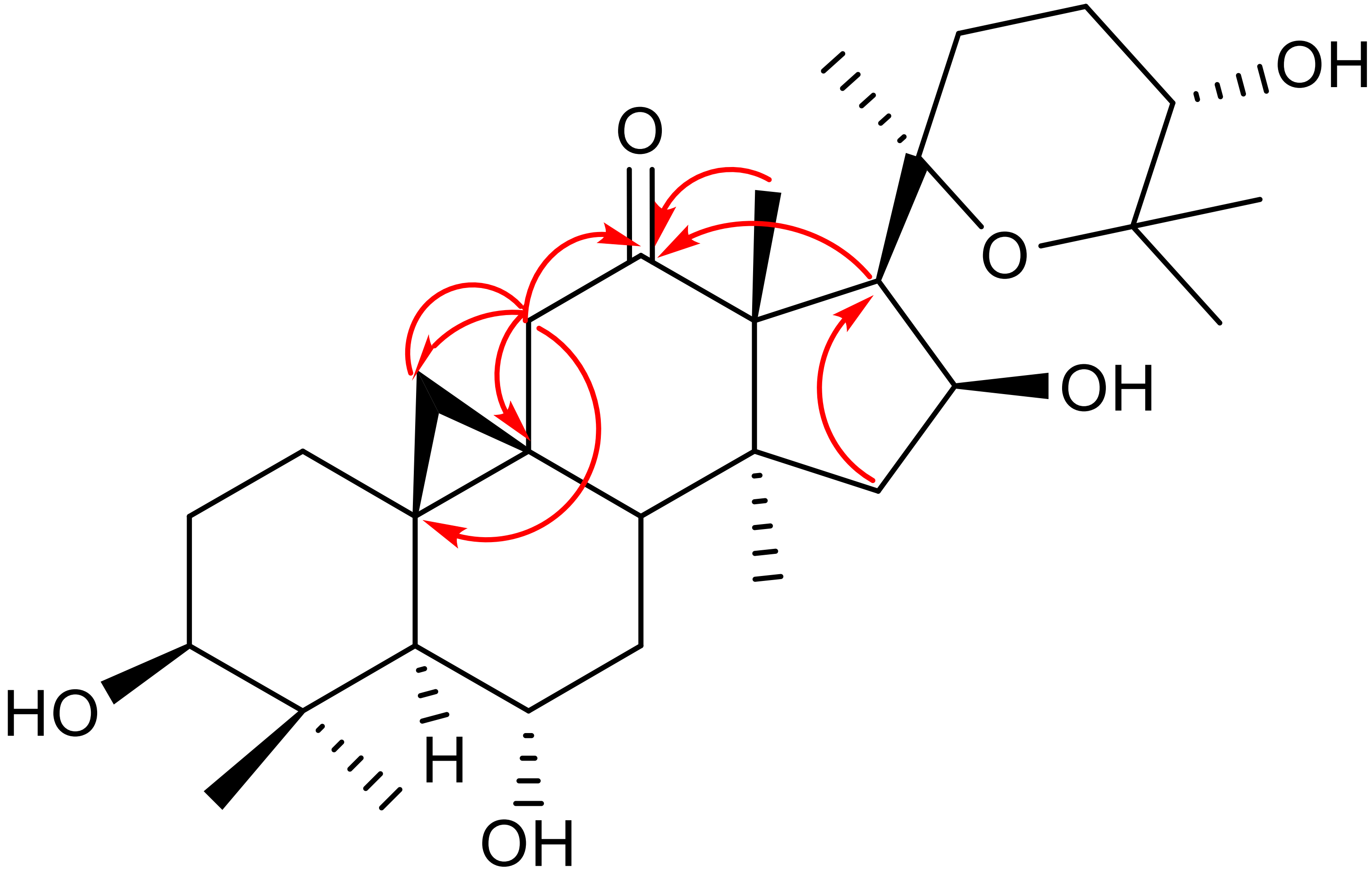

Figure S 84 Key HMBC correlations of compound **9** (arrows from H to C)

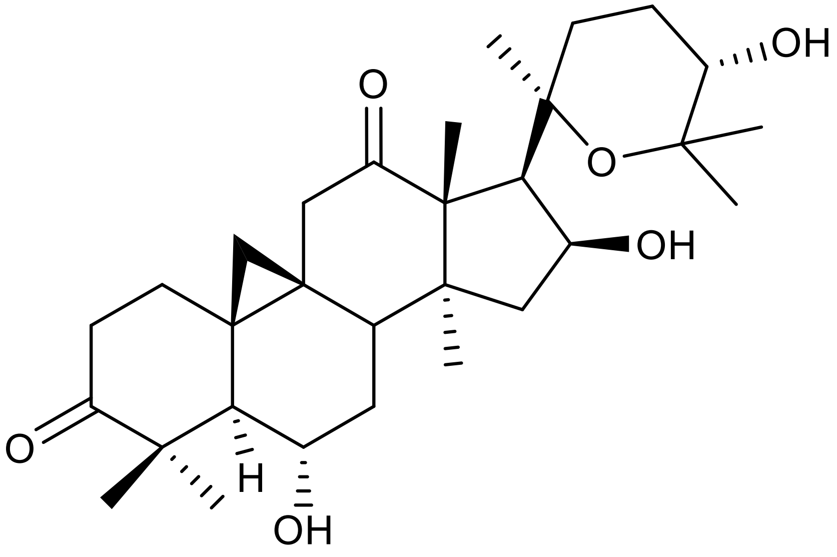

Figure S 85 Structure of compound **10**

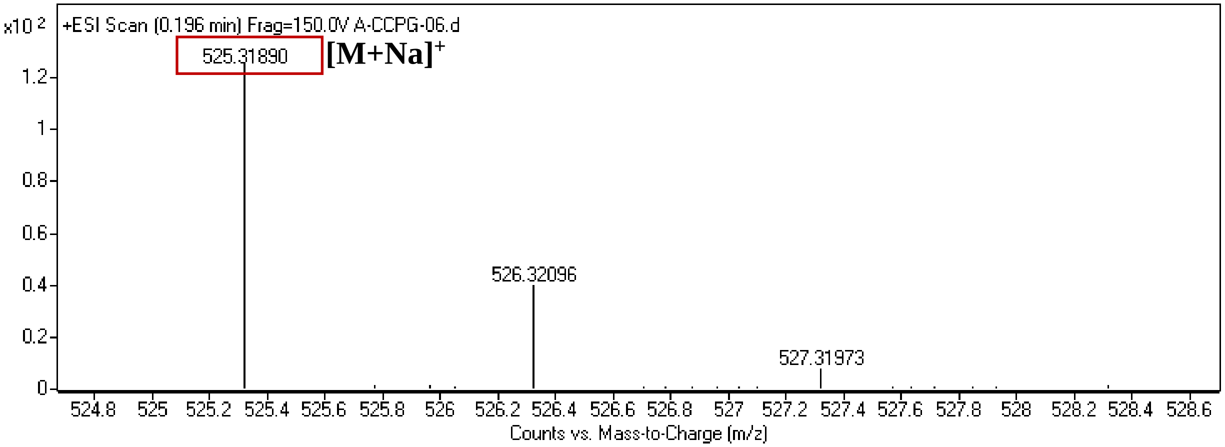

Figure S 86 HR-ESI-MS spectrum of compound **10**

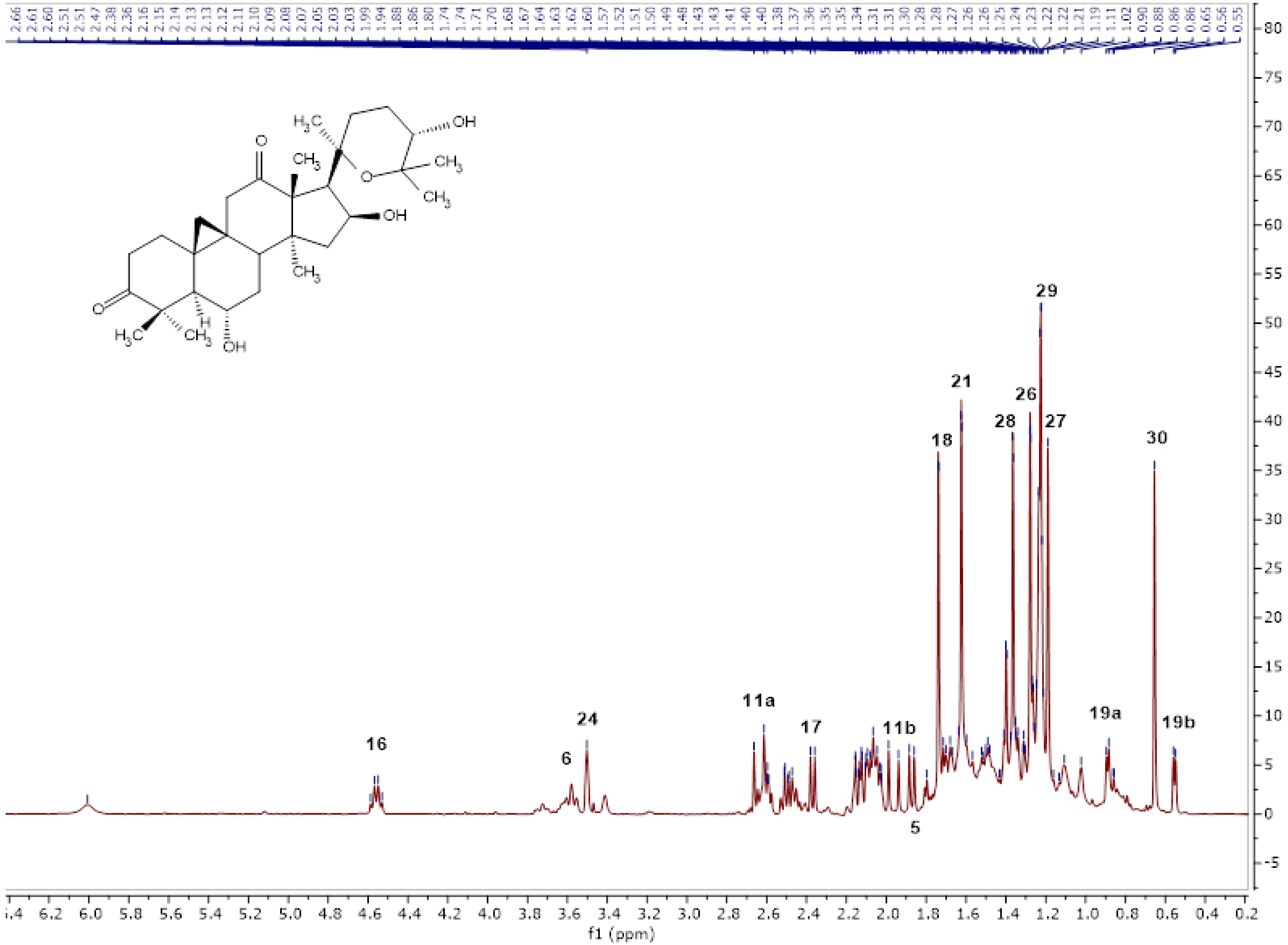

Figure S 87 ^{1}H-NMR spectrum of compound **10** (400 MHz, $CDCl_3$)

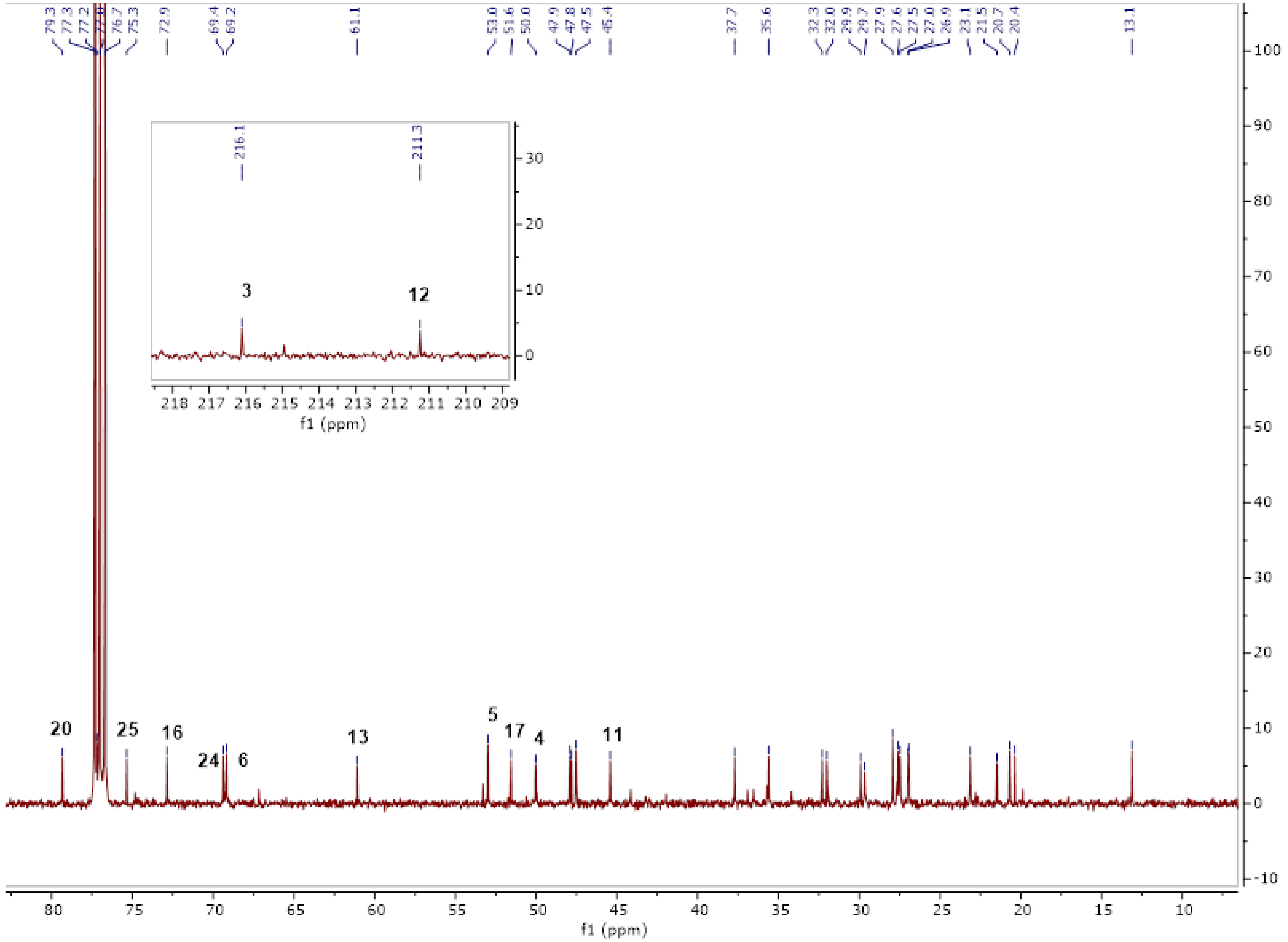

Figure S 88 ^{13}C-NMR spectrum of compound **10** (100 MHz, $CDCl_3$)

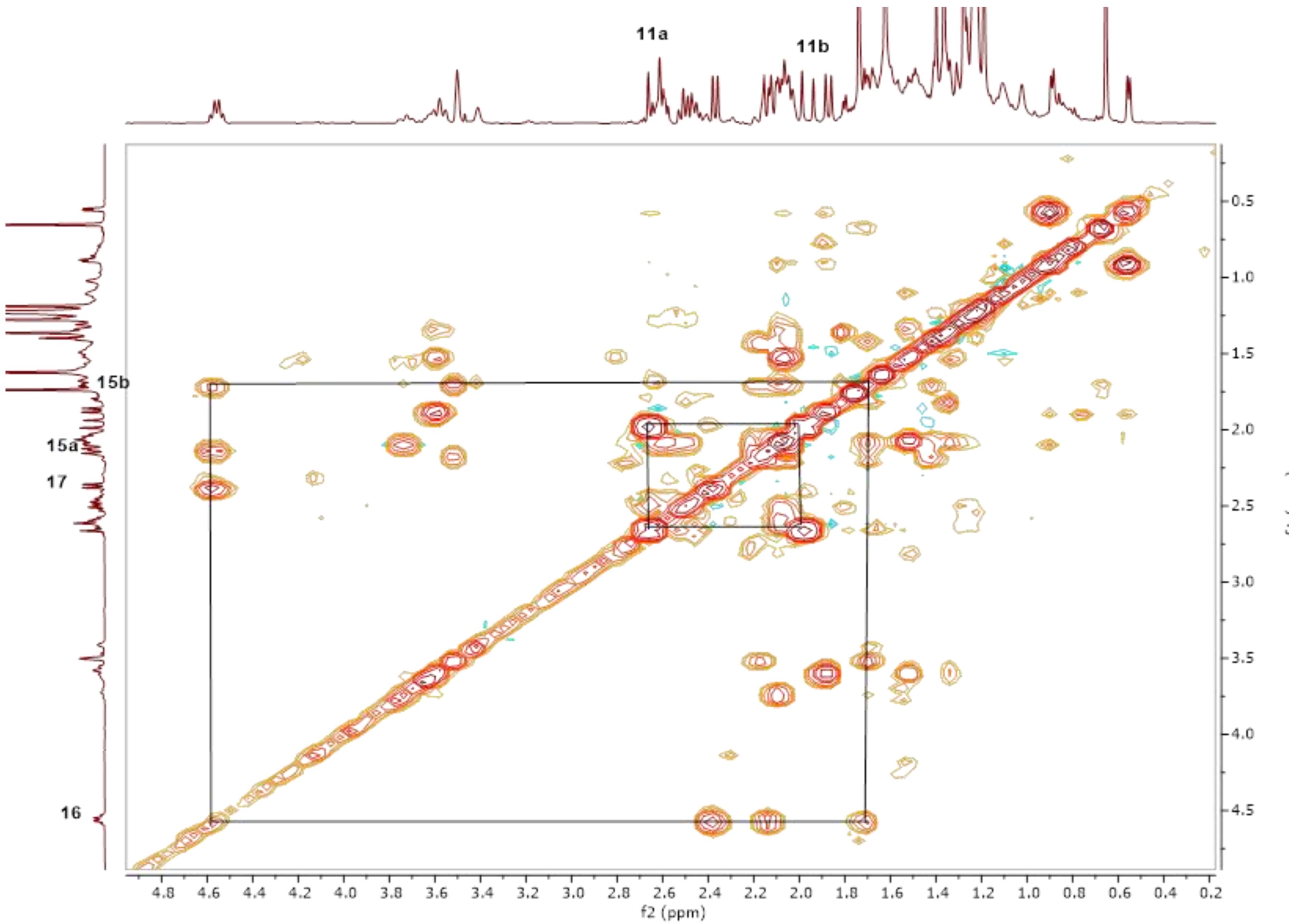

Figure S 89 COSY spectrum of compound **10**

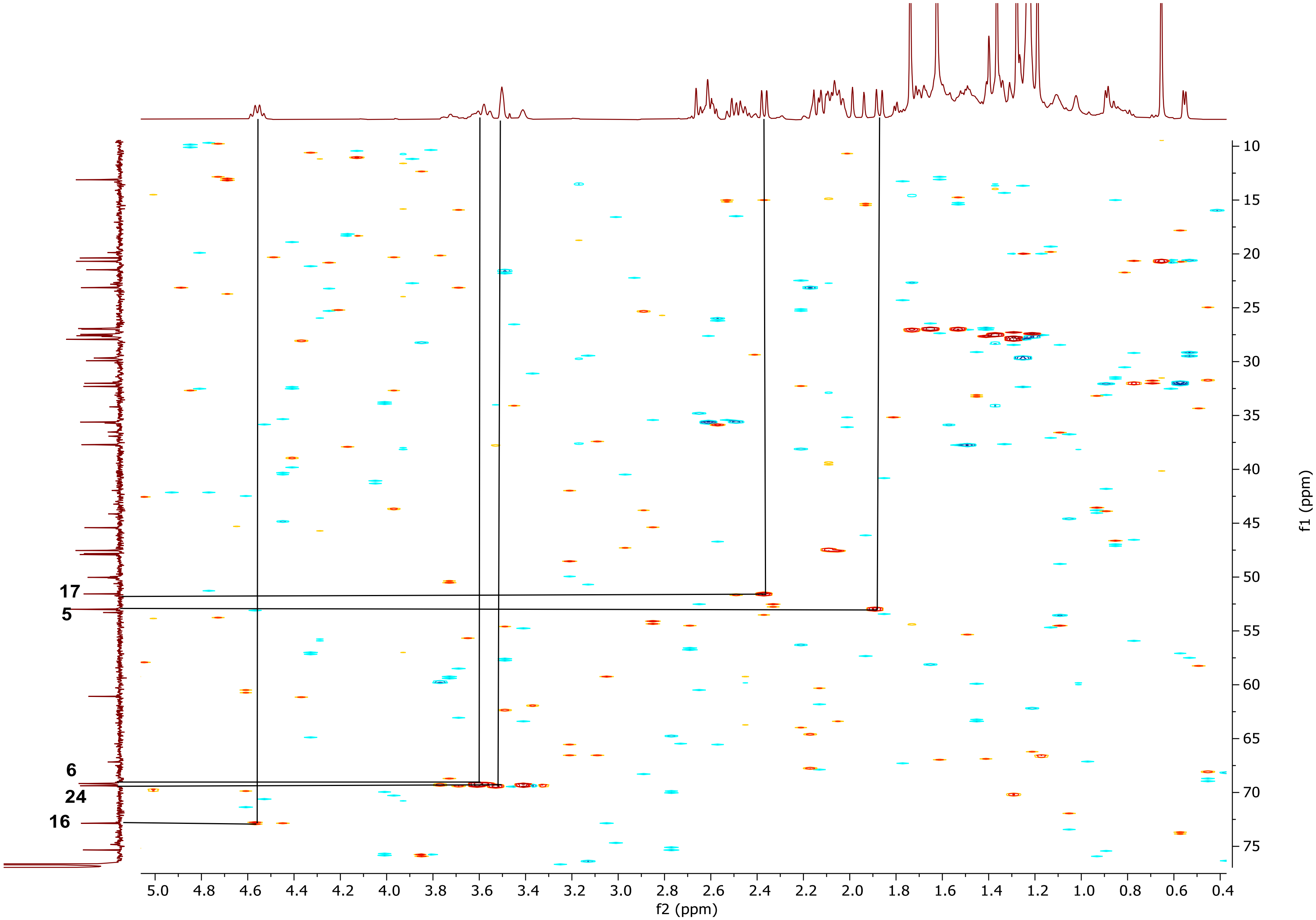

Figure S 90 HSQC spectrum of compound **10**

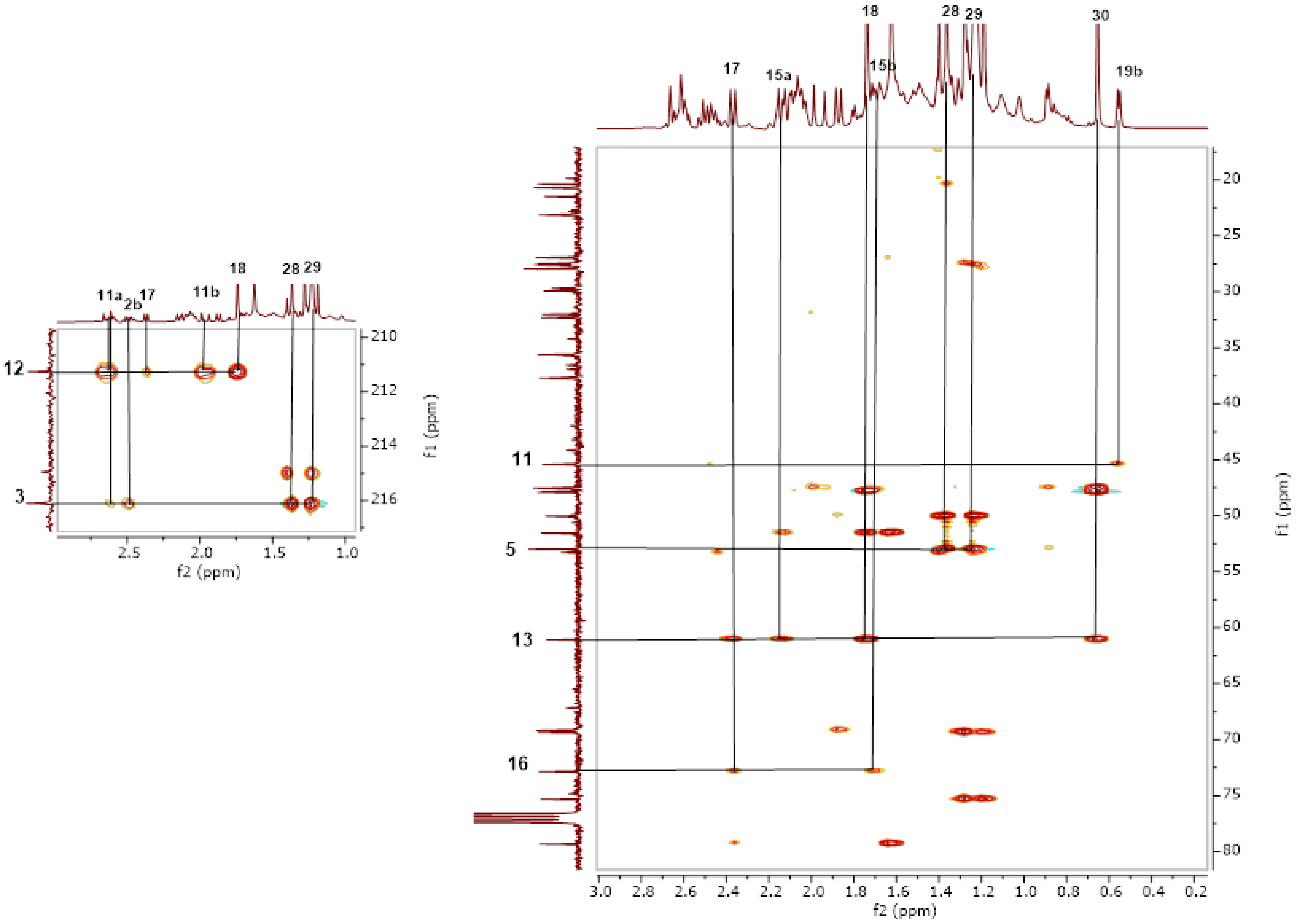

Figure S 91 HMBC spectrum of compound **10**

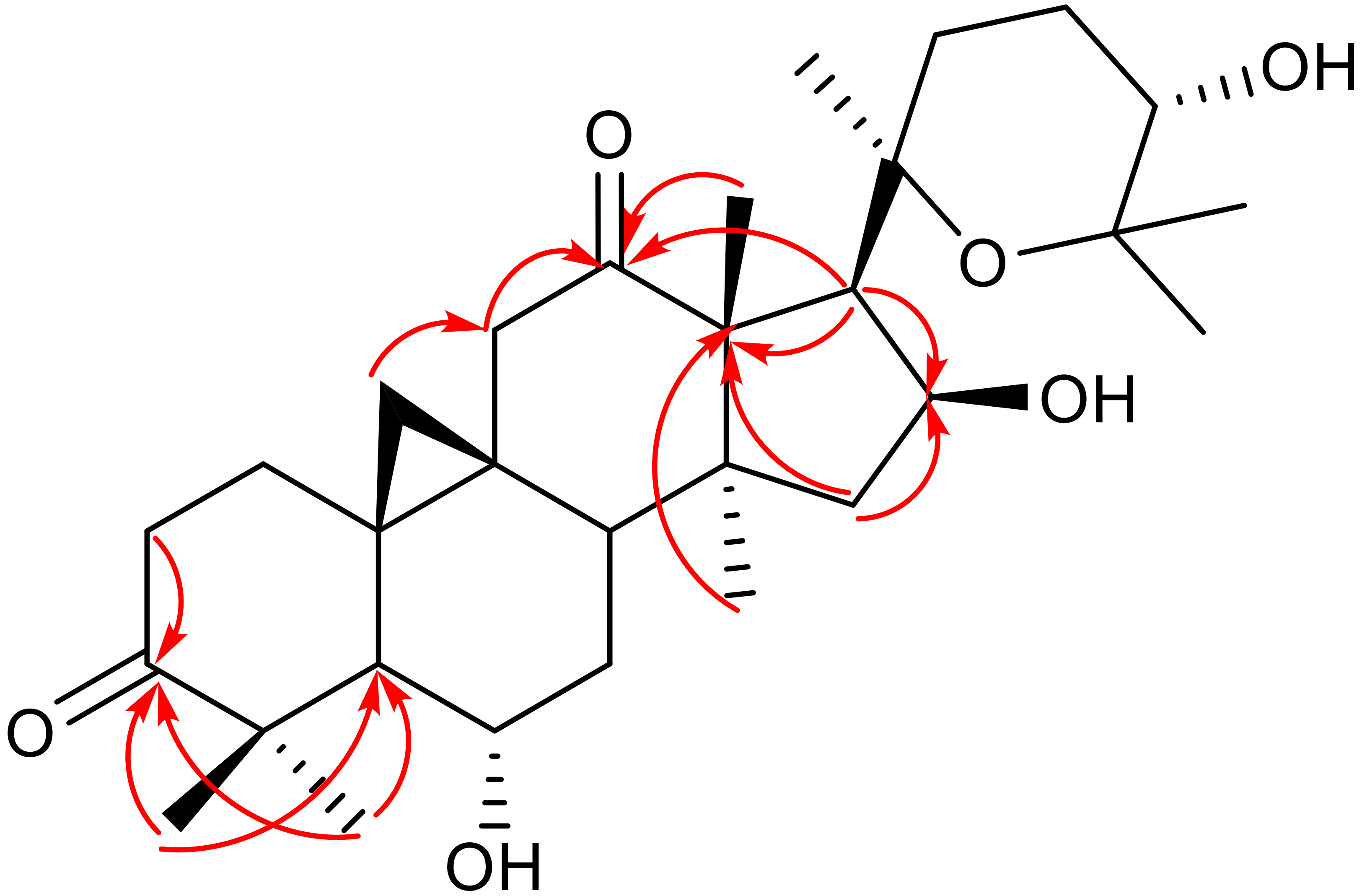

Figure S 92 Key HMBC correlations of compound **10** (arrows from H to C)

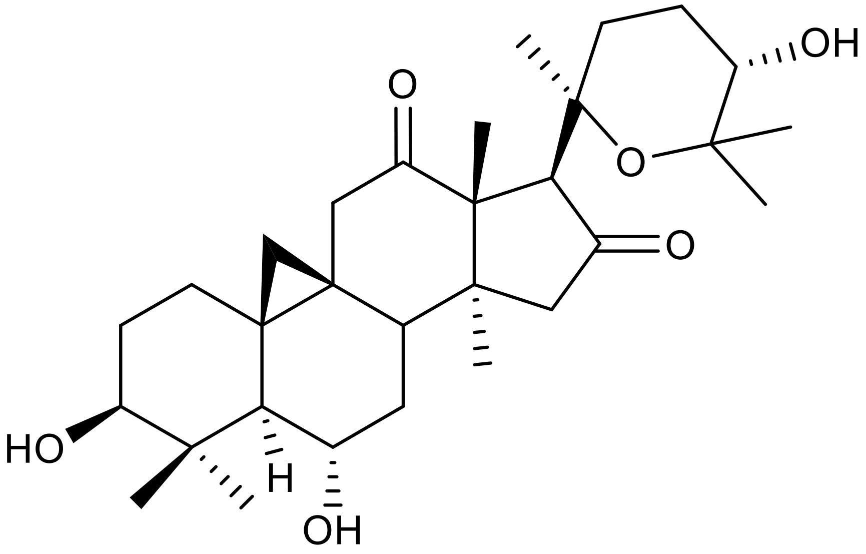

Figure S 93 Structure of compound **11**

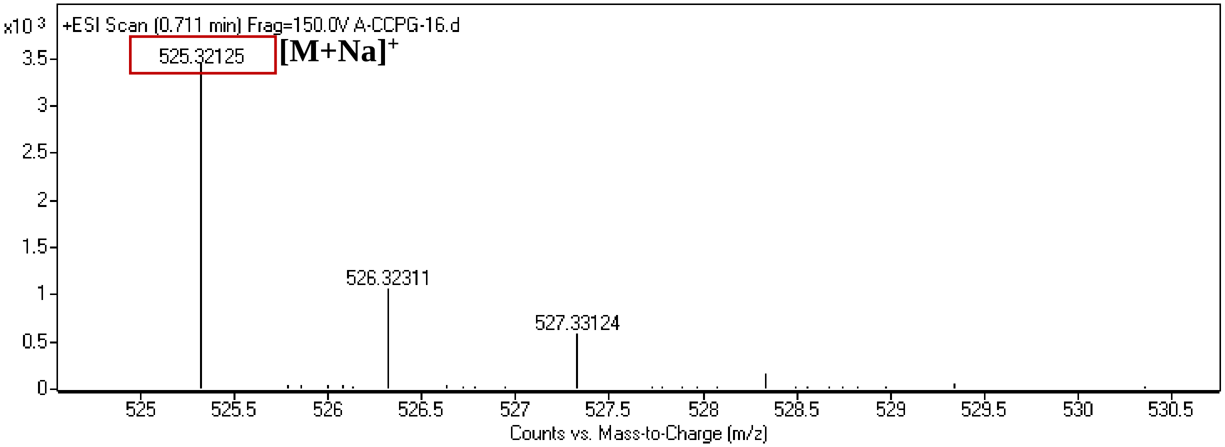

Figure S 94 HR-ESI-MS spectrum of compound **11**

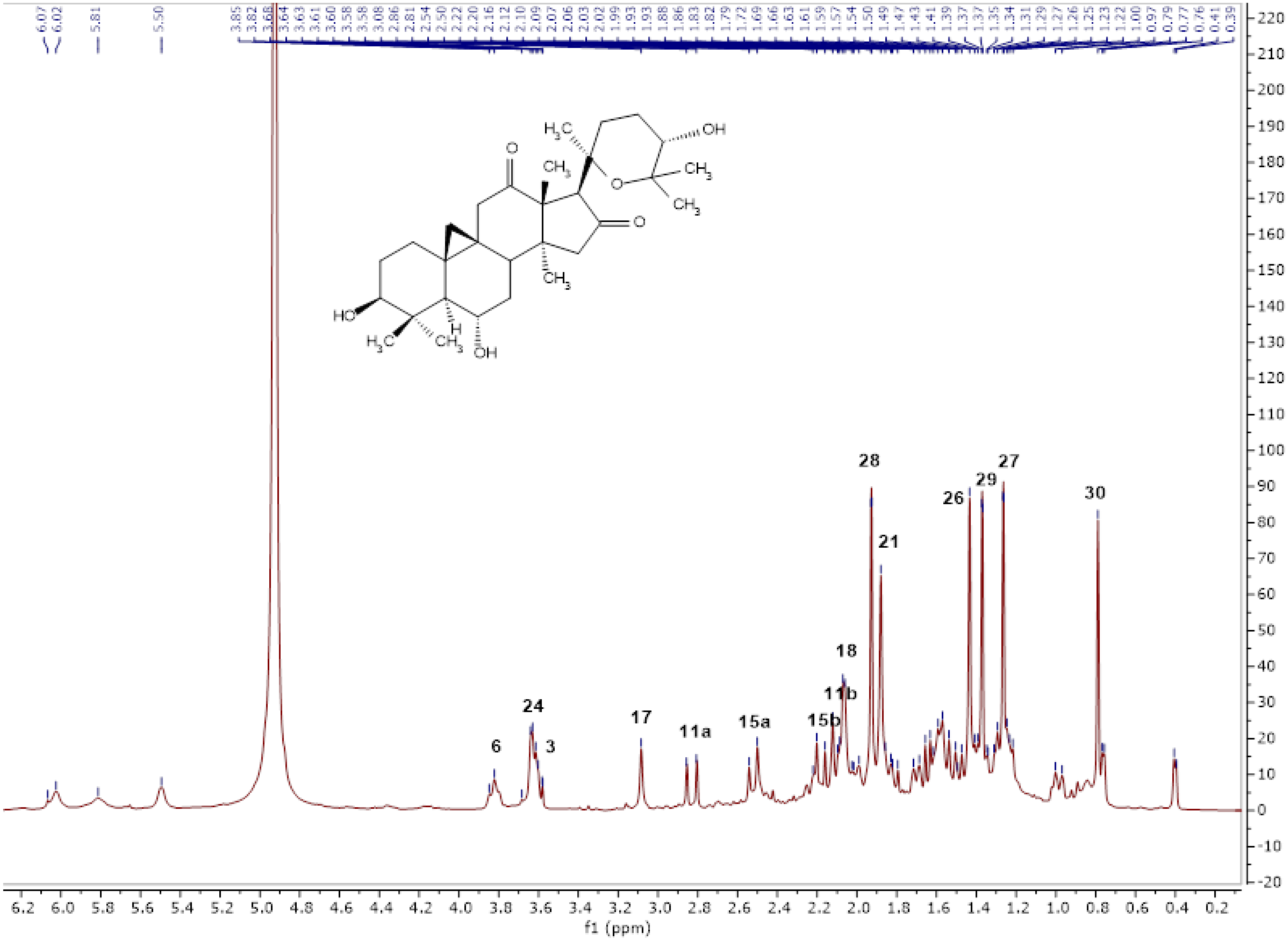

Figure S 95 ^{1}H-NMR spectrum of compound **11** (400 MHz, C_5D_5N)

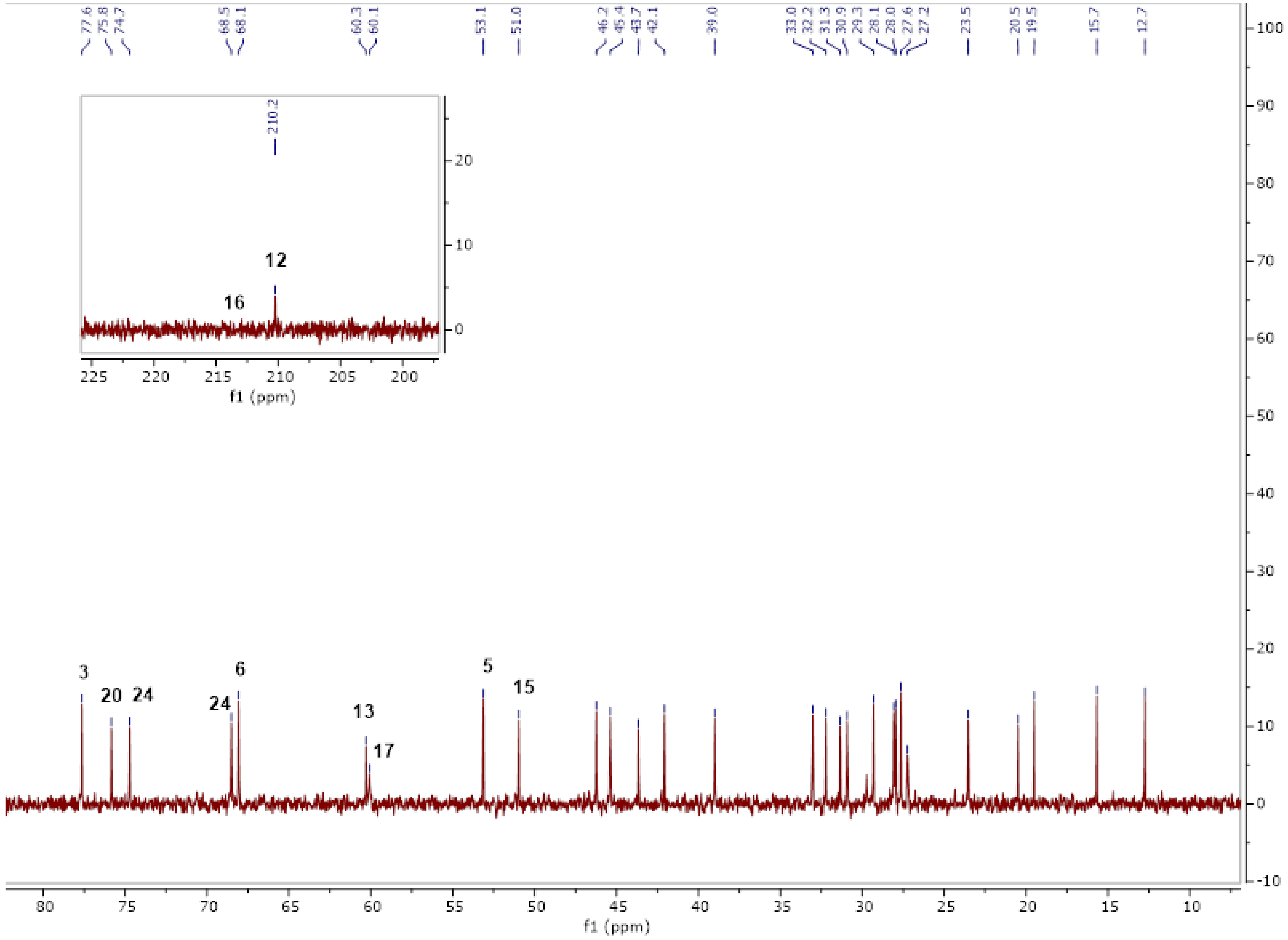

Figure S 96 ^{13}C-NMR spectrum of compound **11** (100 MHz, C_5D_5N)

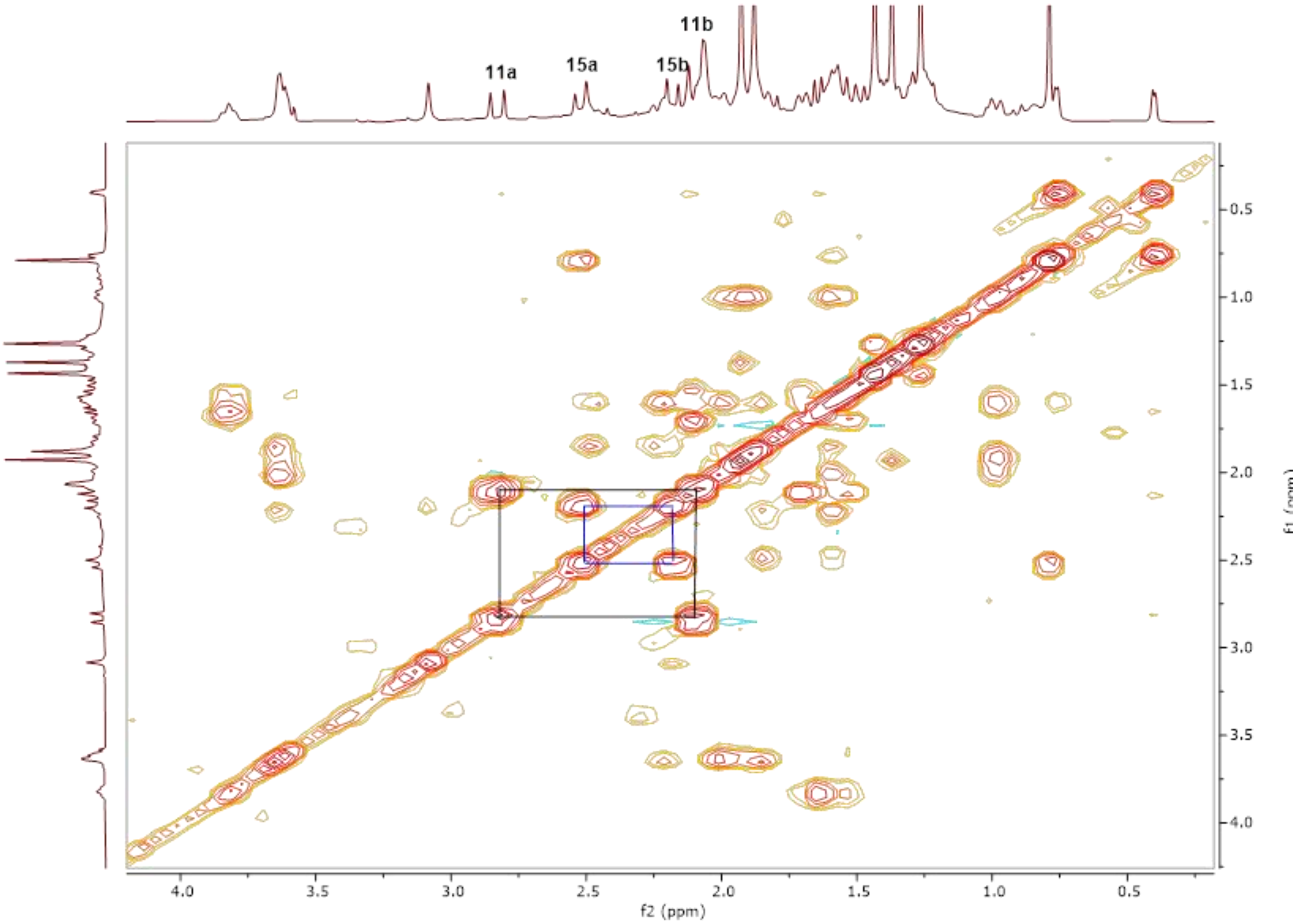

Figure S 97 COSY spectrum of compound **11**

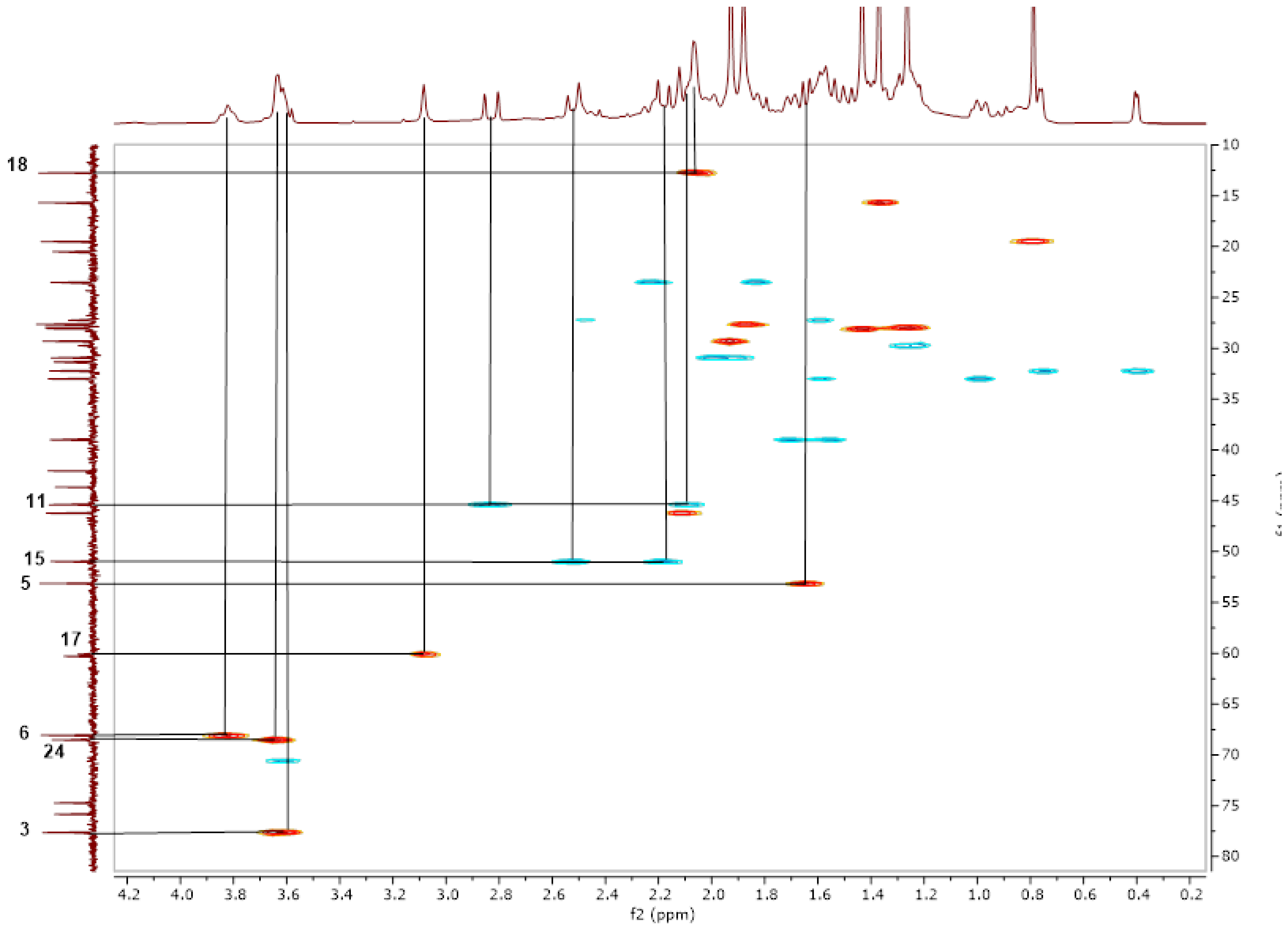

Figure S 98 HSQC spectrum of compound **11**

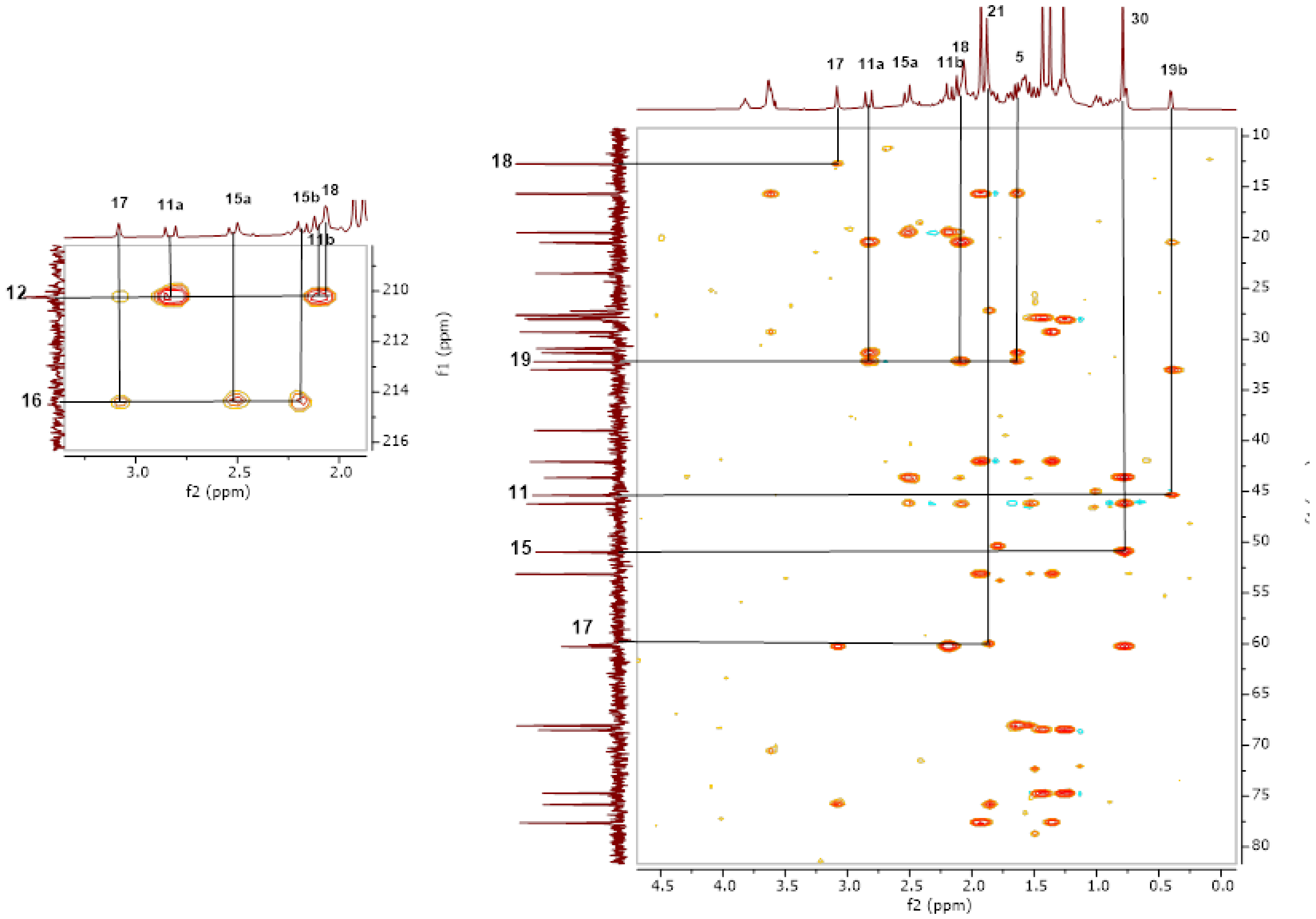

Figure S 99 HMBC spectrum of compound **11**

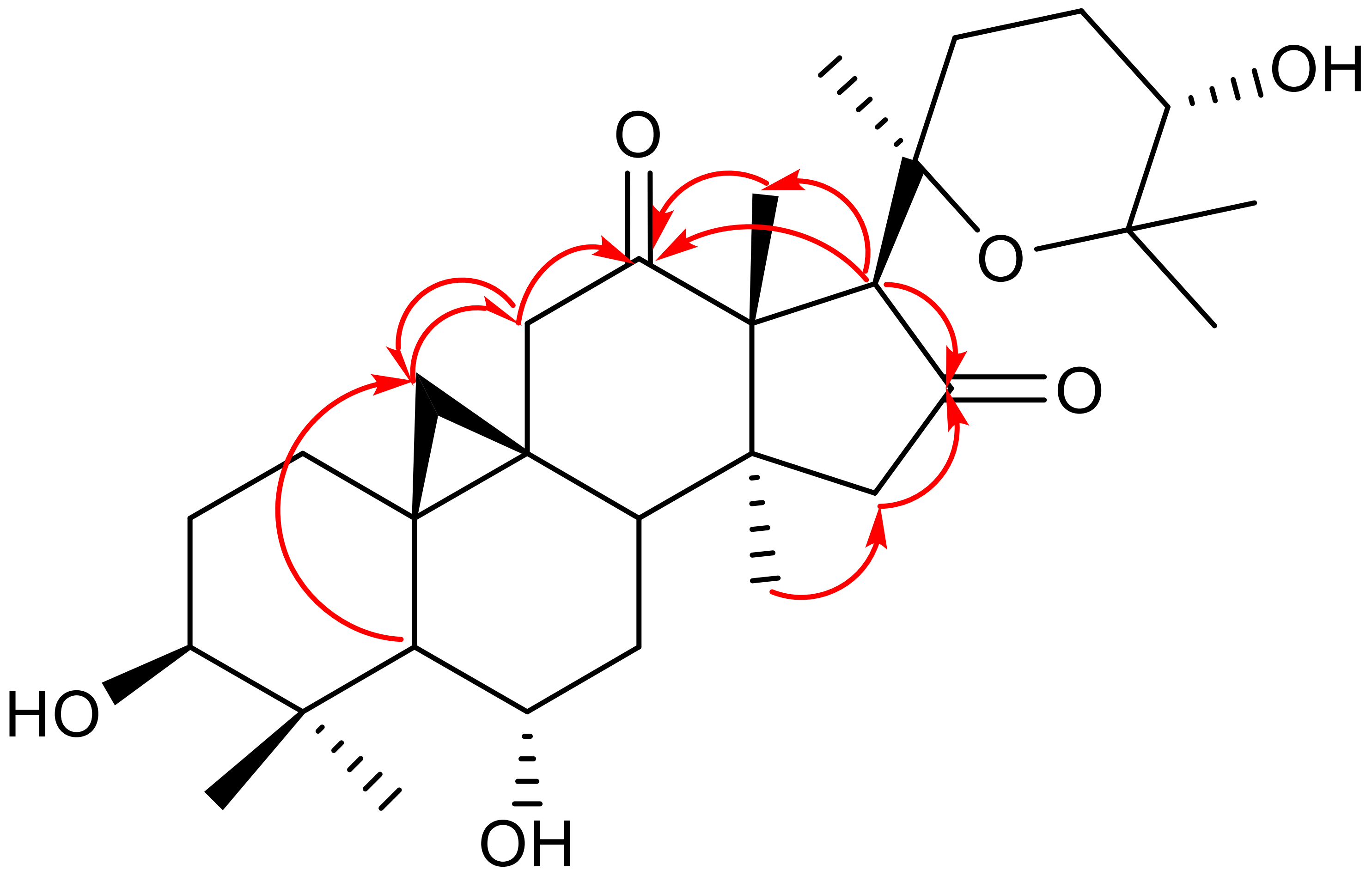

Figure S 100 Key HMBC correlations of compound **11** (arrows from H to C)

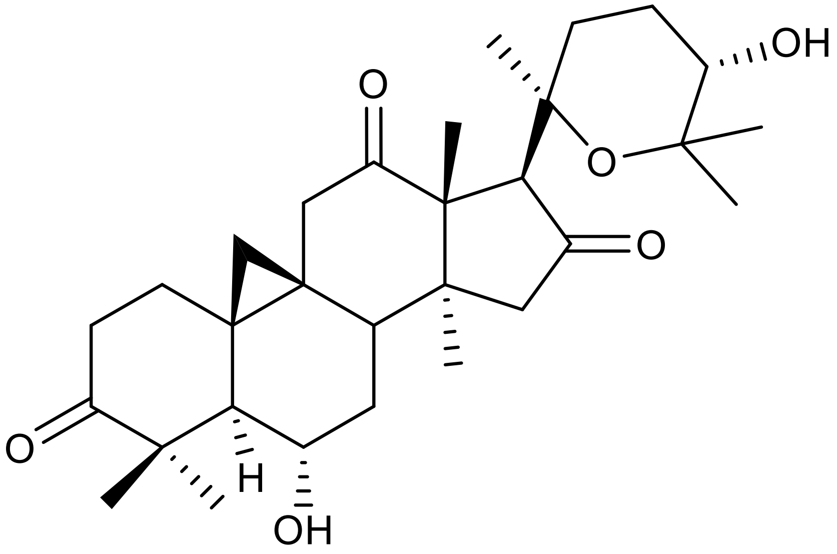

Figure S 101 Structure of compound **12**

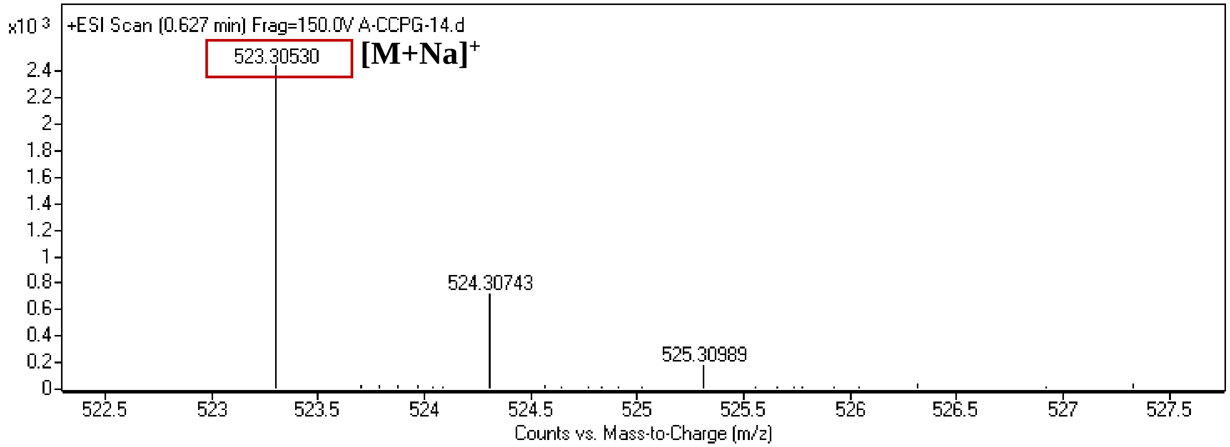

Figure S 102 HR-ESI-MS spectrum of compound **12**

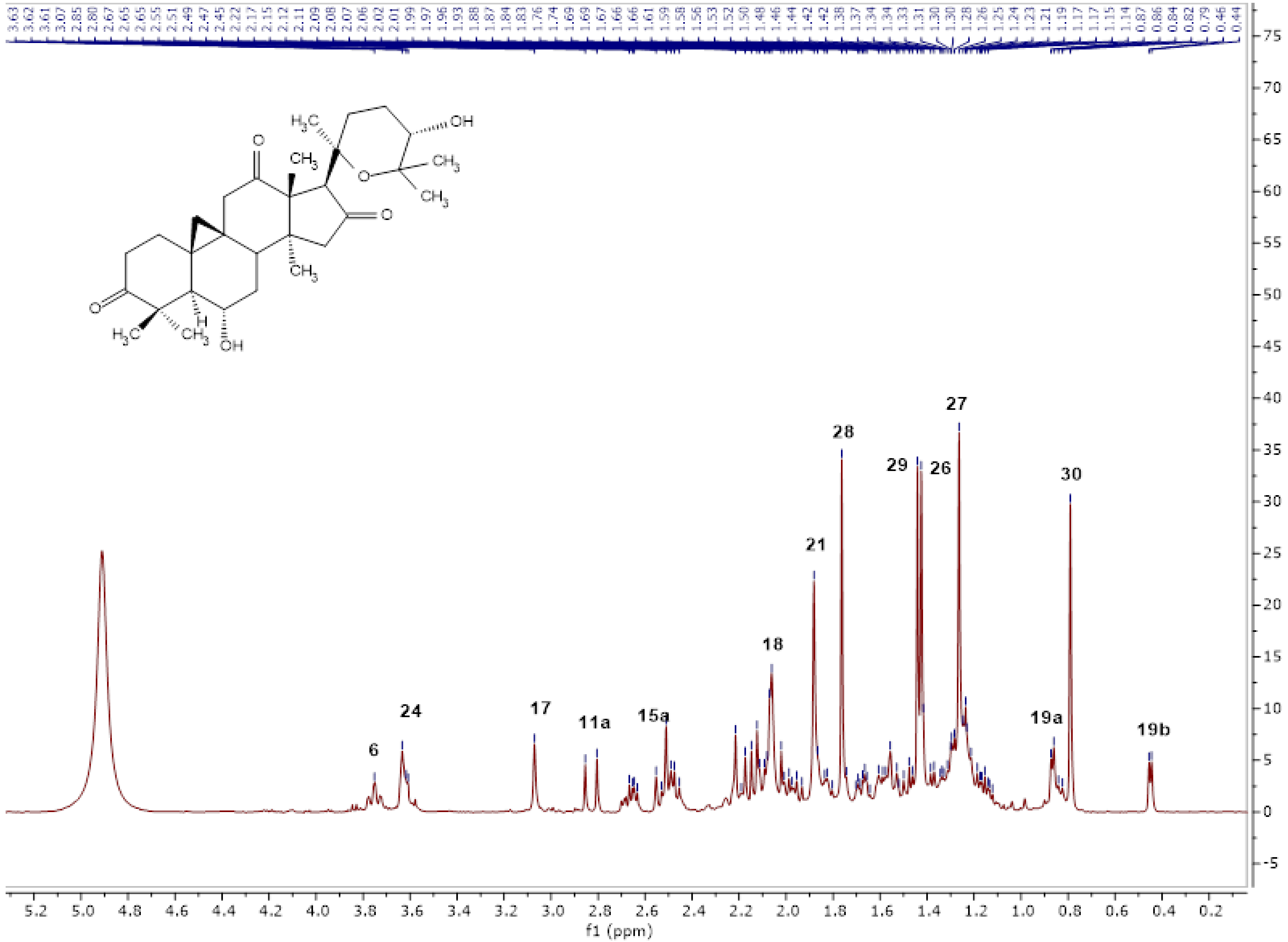

Figure S 103 ^{1}H-NMR spectrum of compound **12** (400 MHz, C_5D_5N)

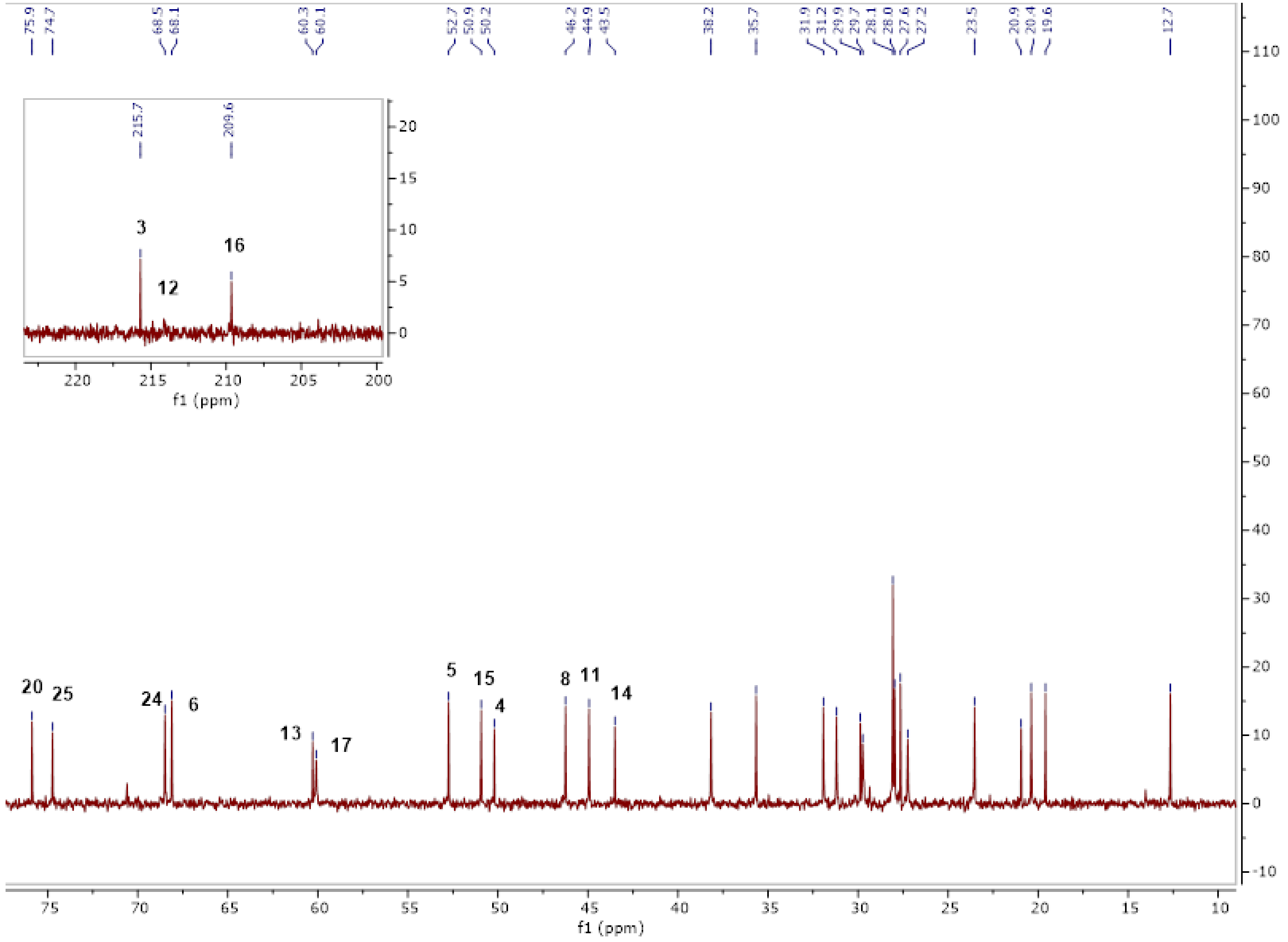

Figure S 104 ^{13}C-NMR spectrum of compound **12** (100 MHz, C_5D_5N)

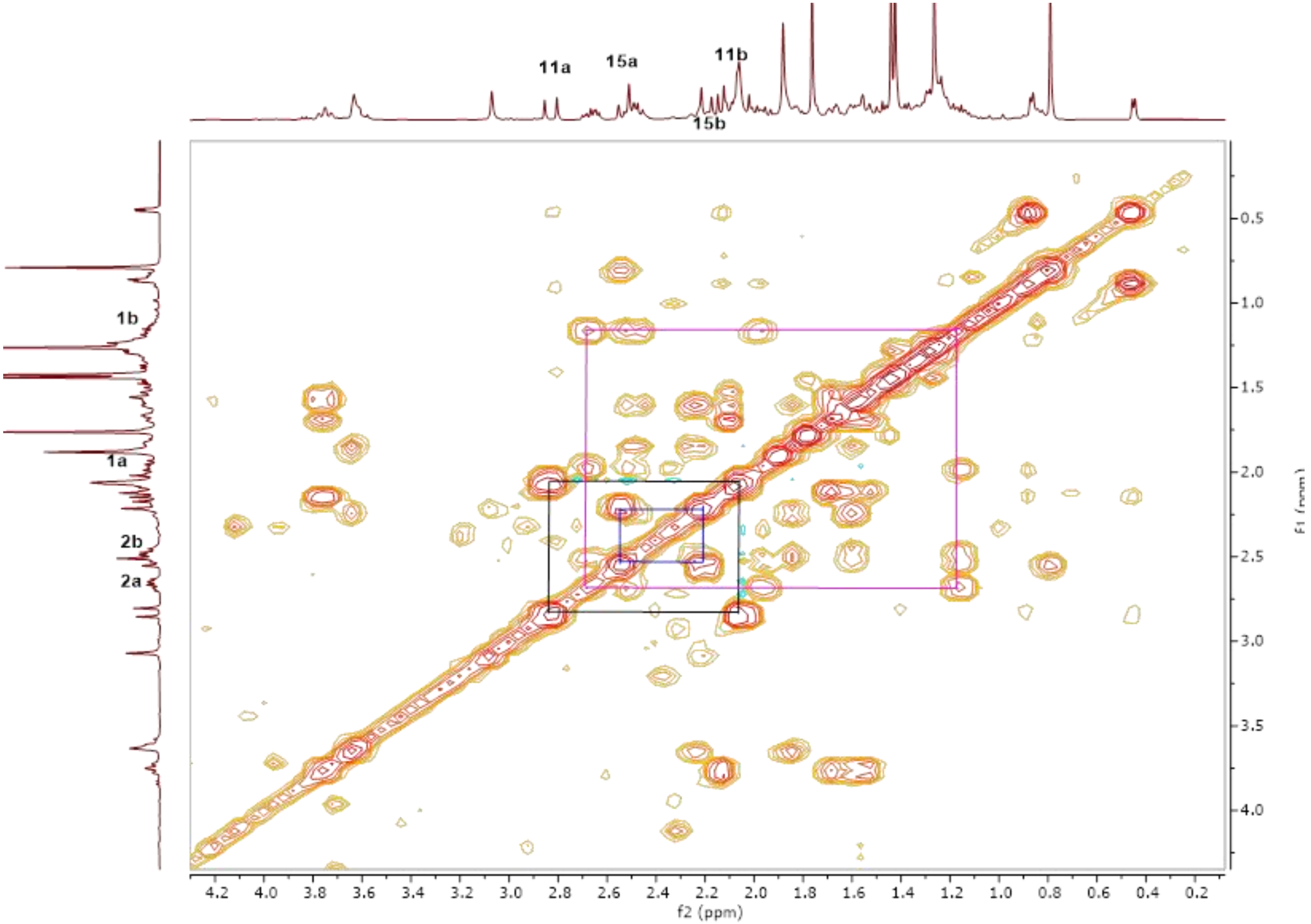

Figure S 105 COSY spectrum of compound **12**

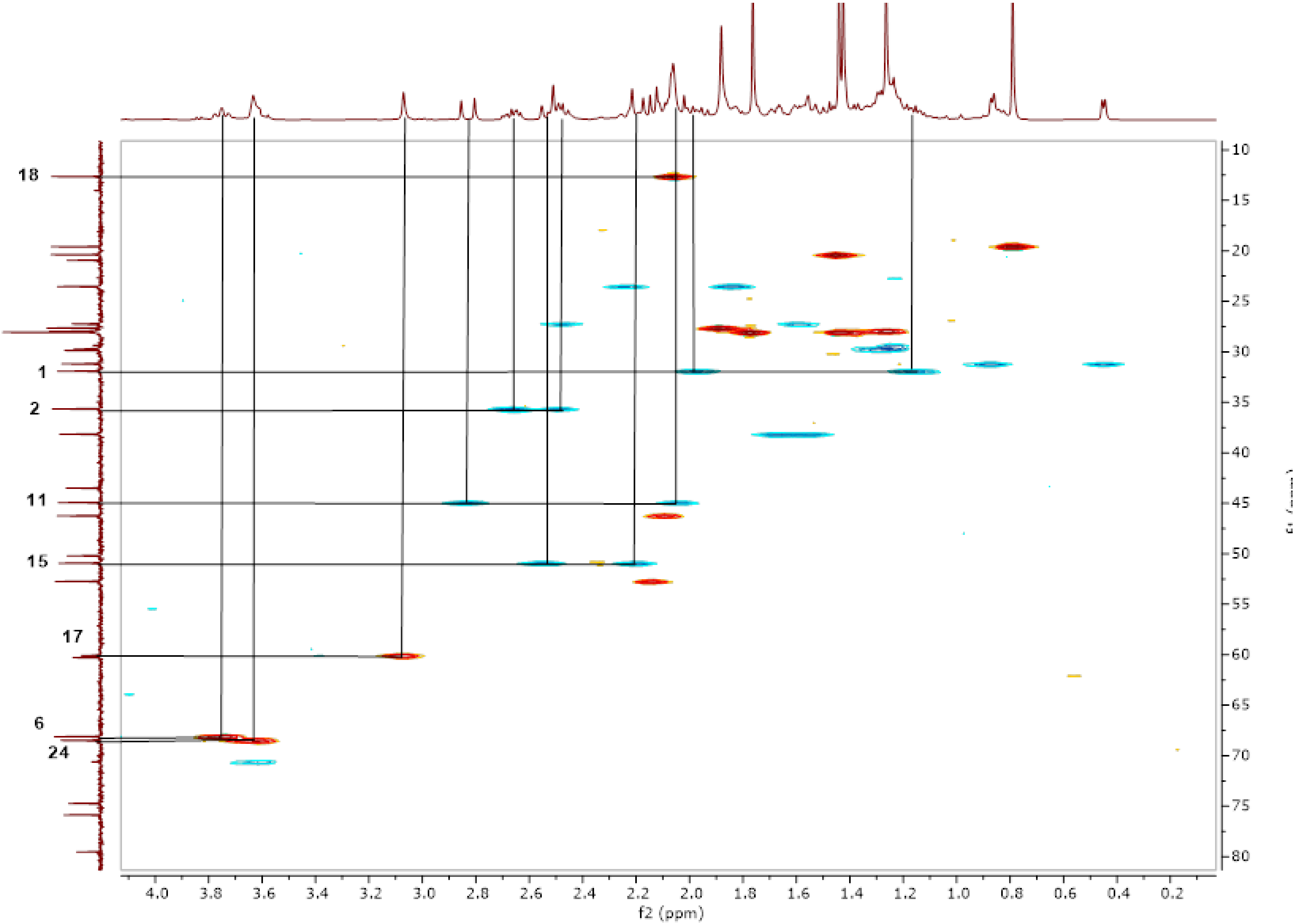

Figure S 106 HSQC spectrum of compound **12**

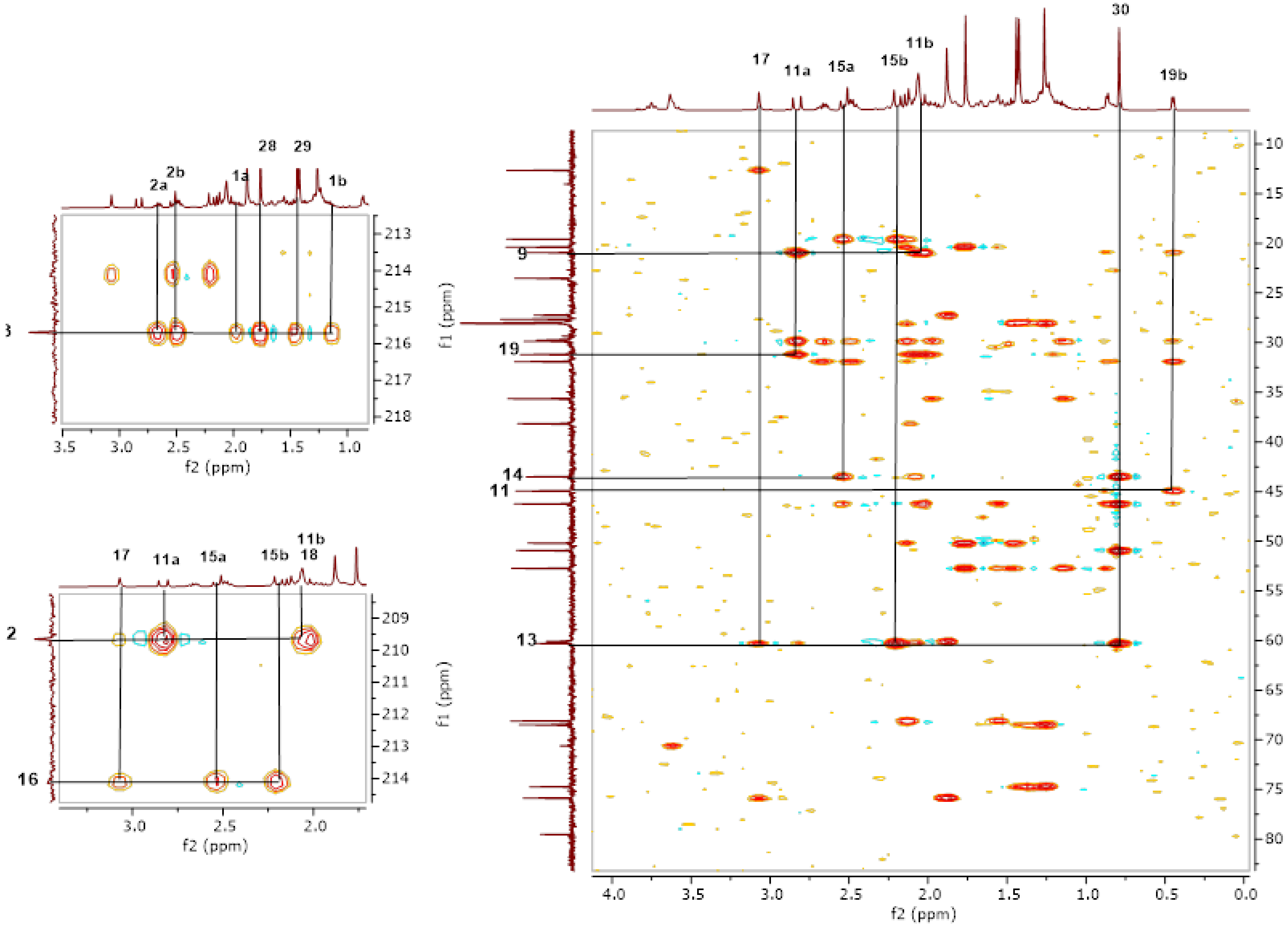

Figure S 107 HMBC spectrum of compound **12**

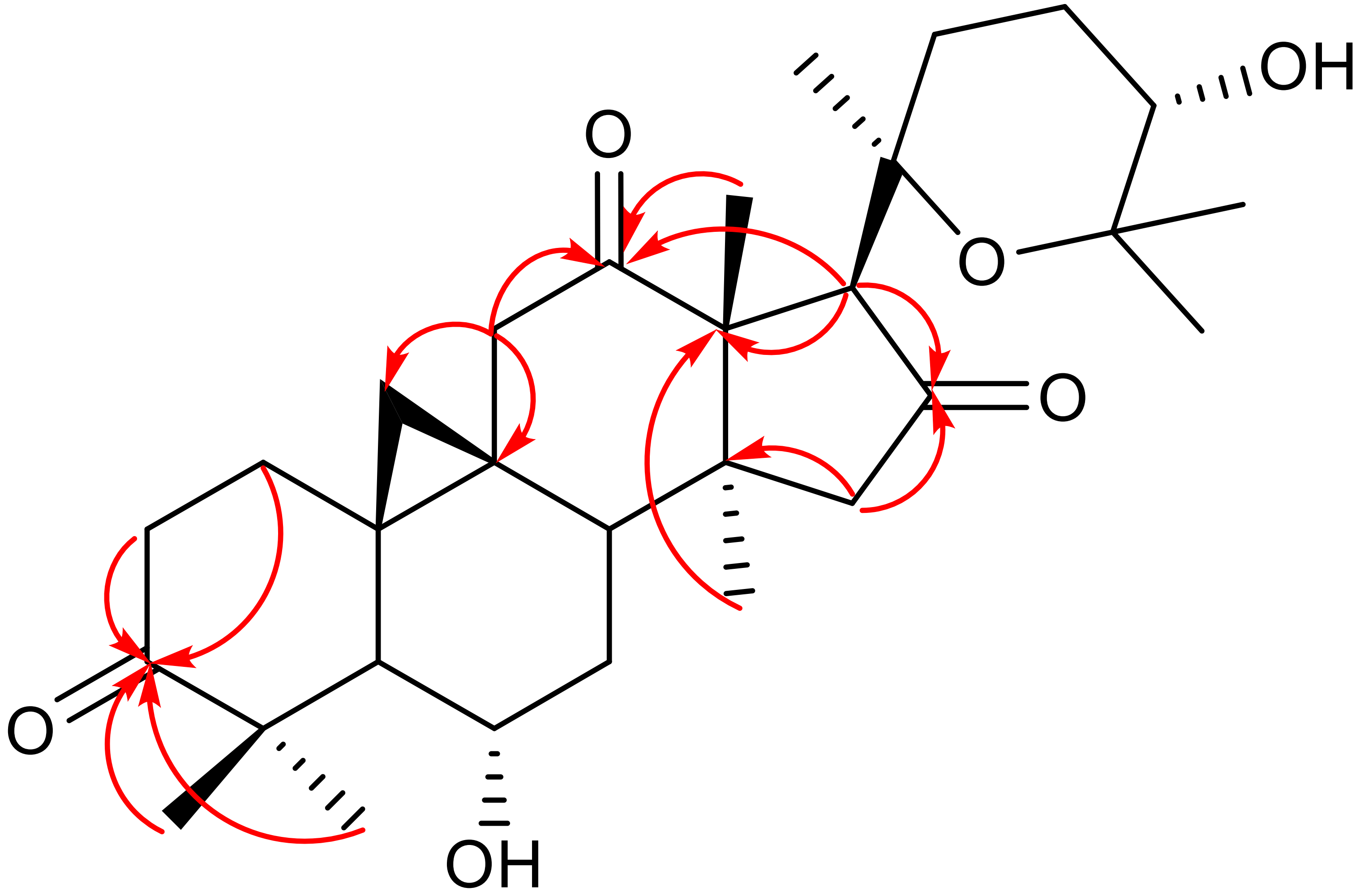

Figure S 108 Key HMBC correlations of compound **12** (arrows from H to C)

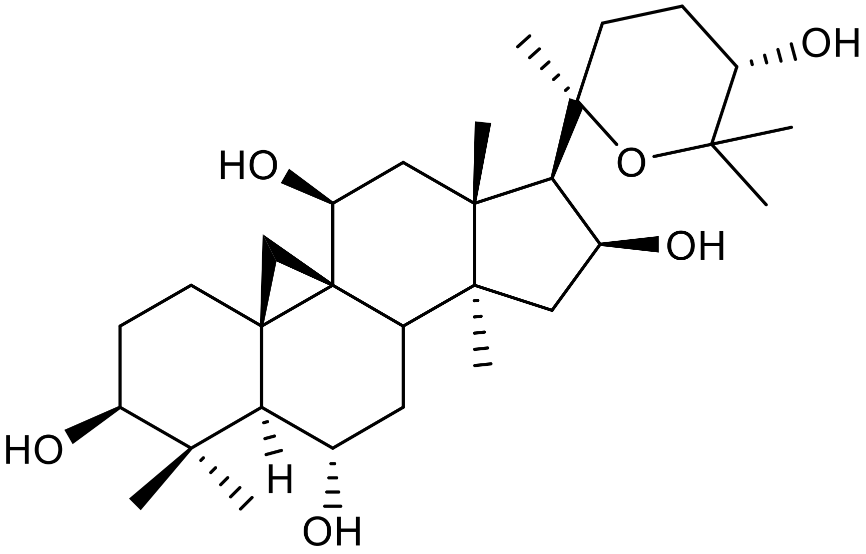

Figure S 109 Structure of compound **13**

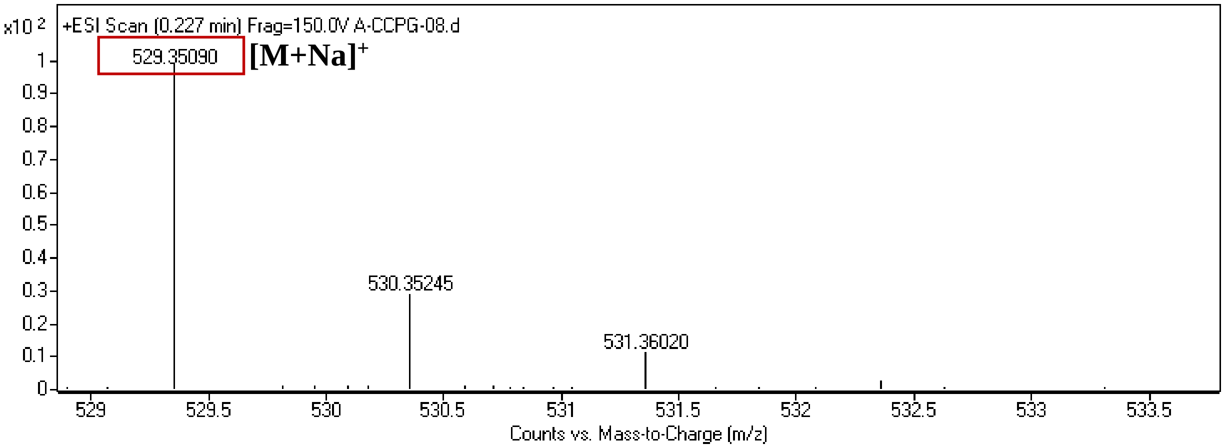

Figure S 110 HR-ESI-MS spectrum of compound **13**

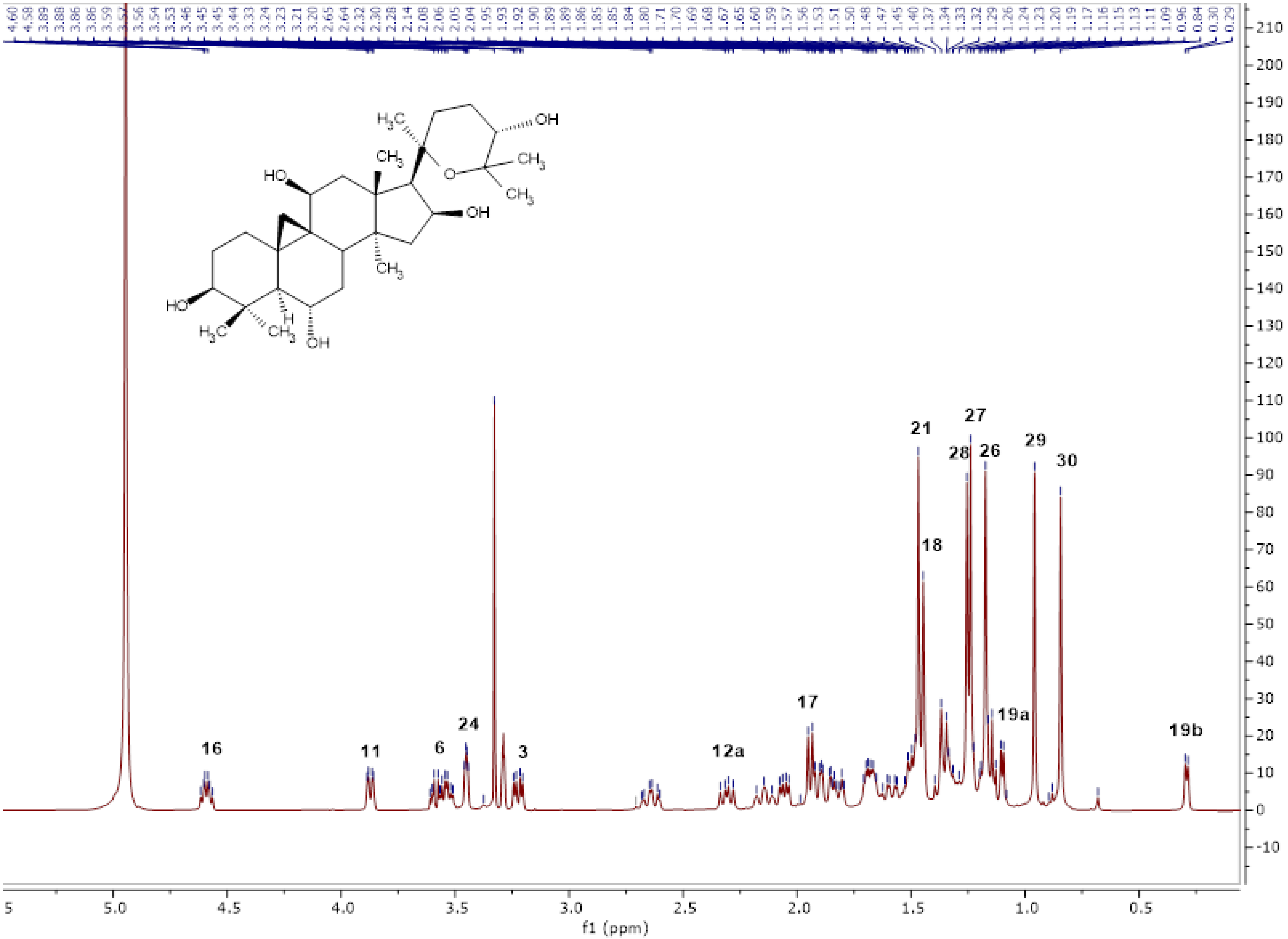

Figure S 111 ^{1}H-NMR spectrum of compound **13** (400 MHz, CD_3OD and a drop of C_5D_5N)

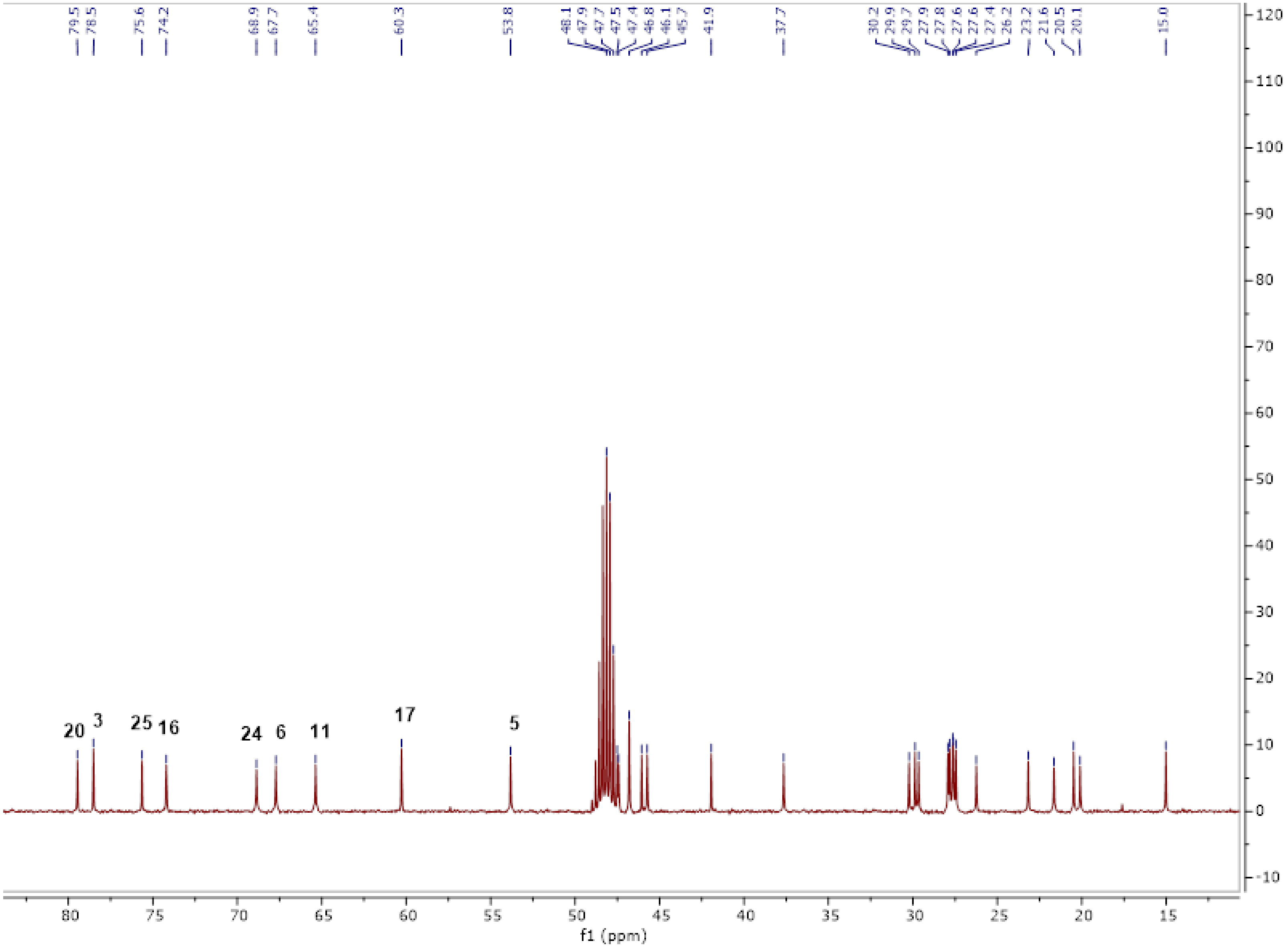

Figure S 112 ^{13}C-NMR spectrum of compound **13** (100 MHz, CD_3OD and a drop of C_5D_5N)

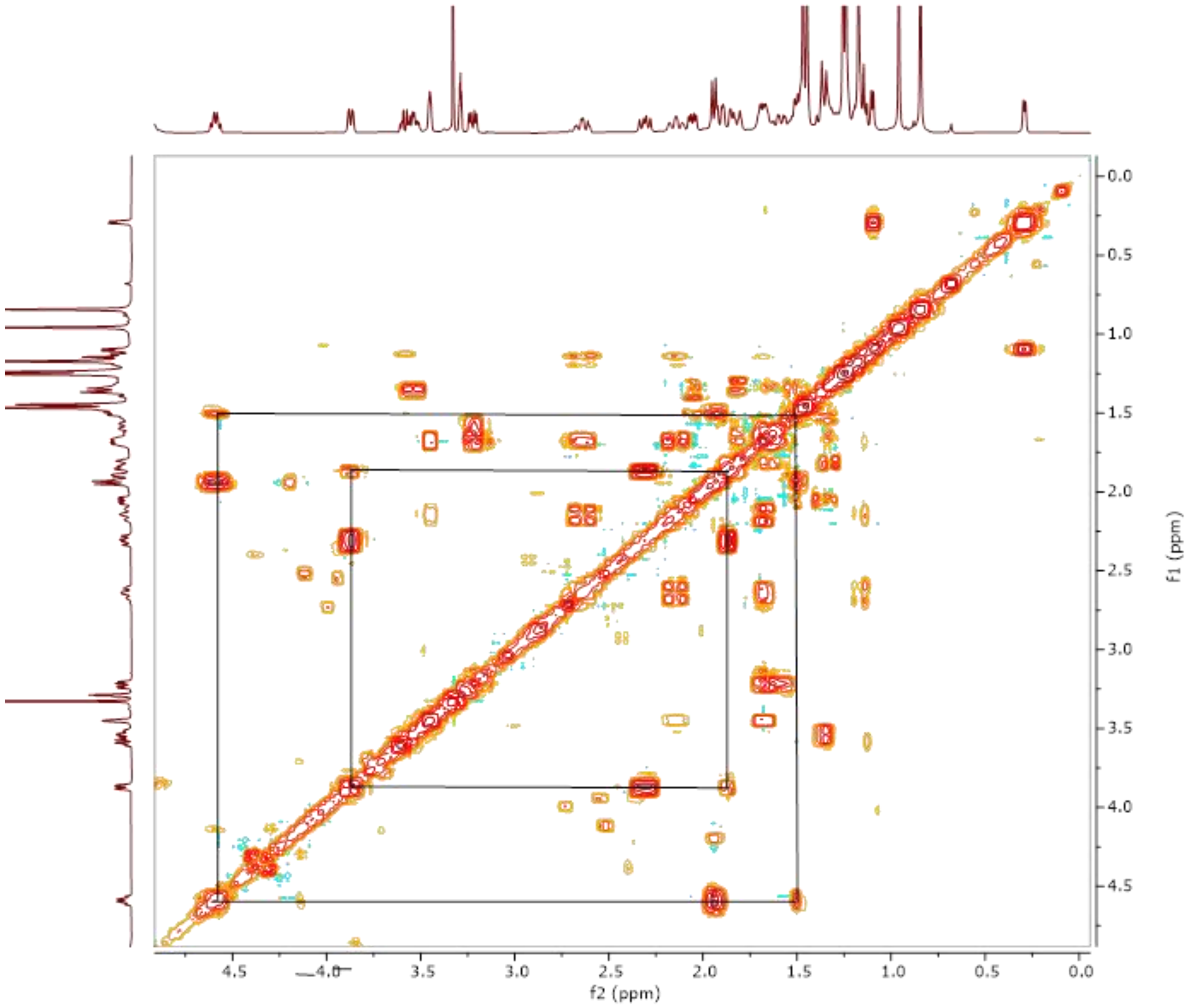

Figure S 113 COSY spectrum of compound **13**

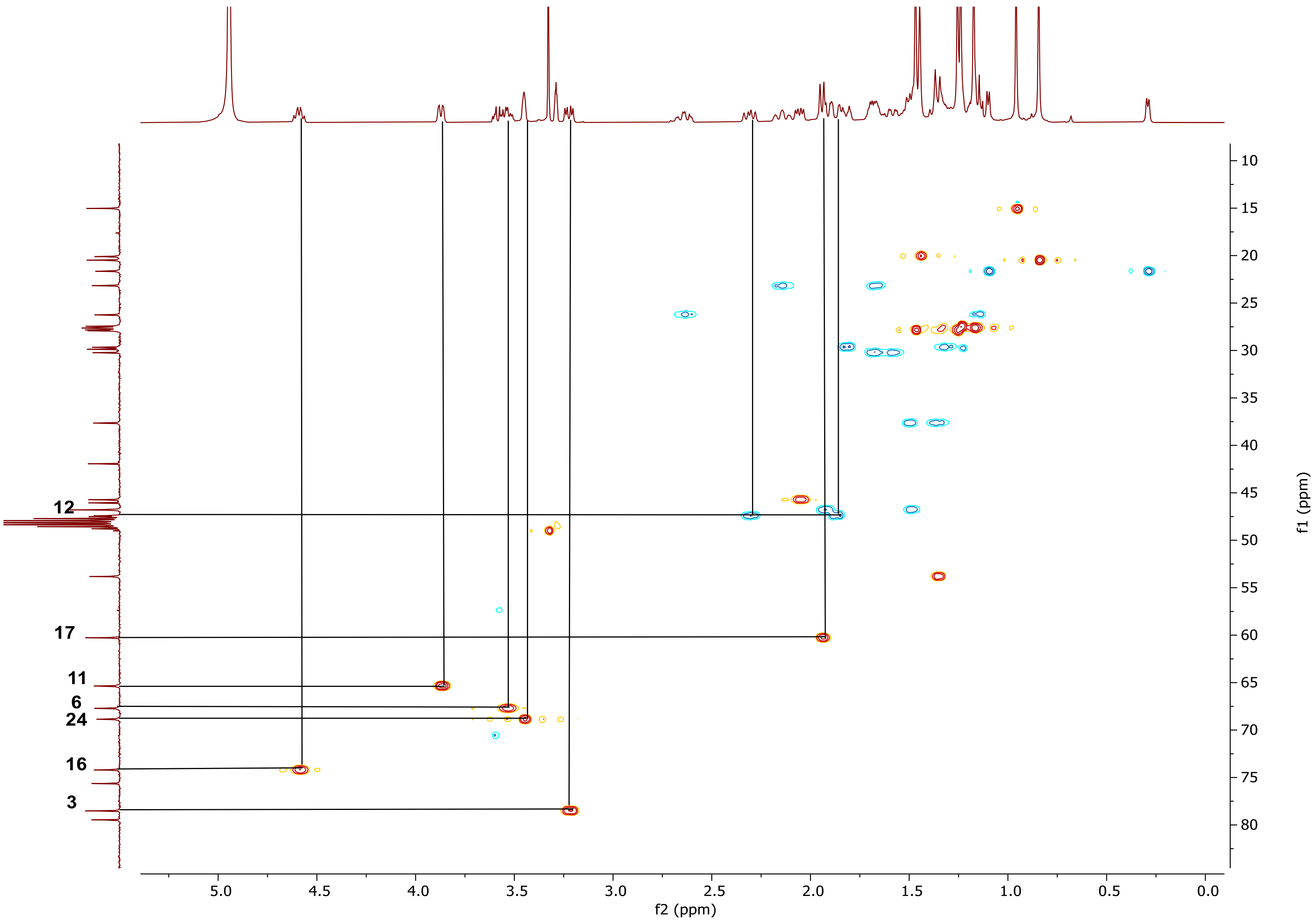

Figure S 114 HSQC spectrum of compound **13**

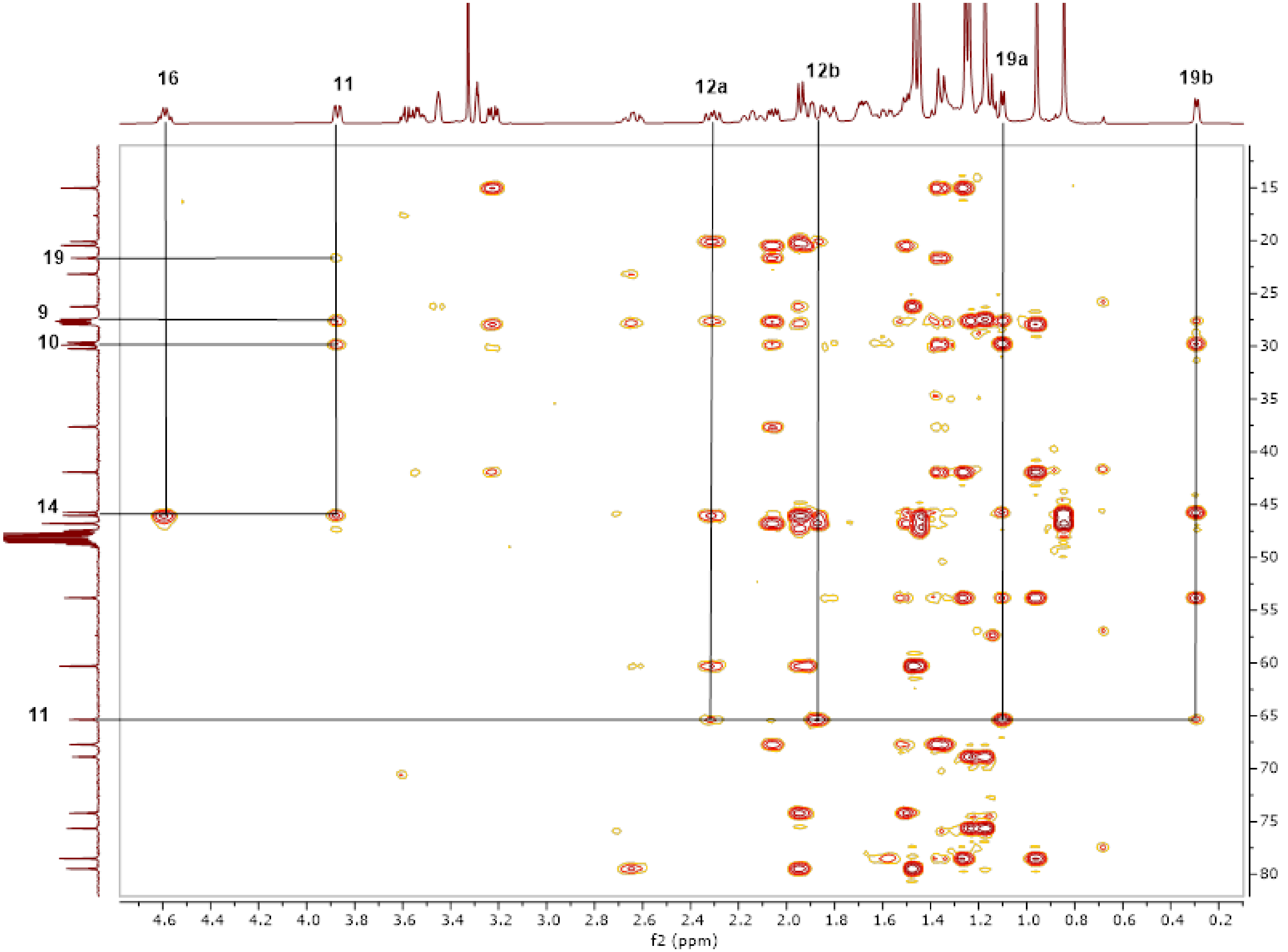

Figure S 115 HMBC spectrum of compound **13**

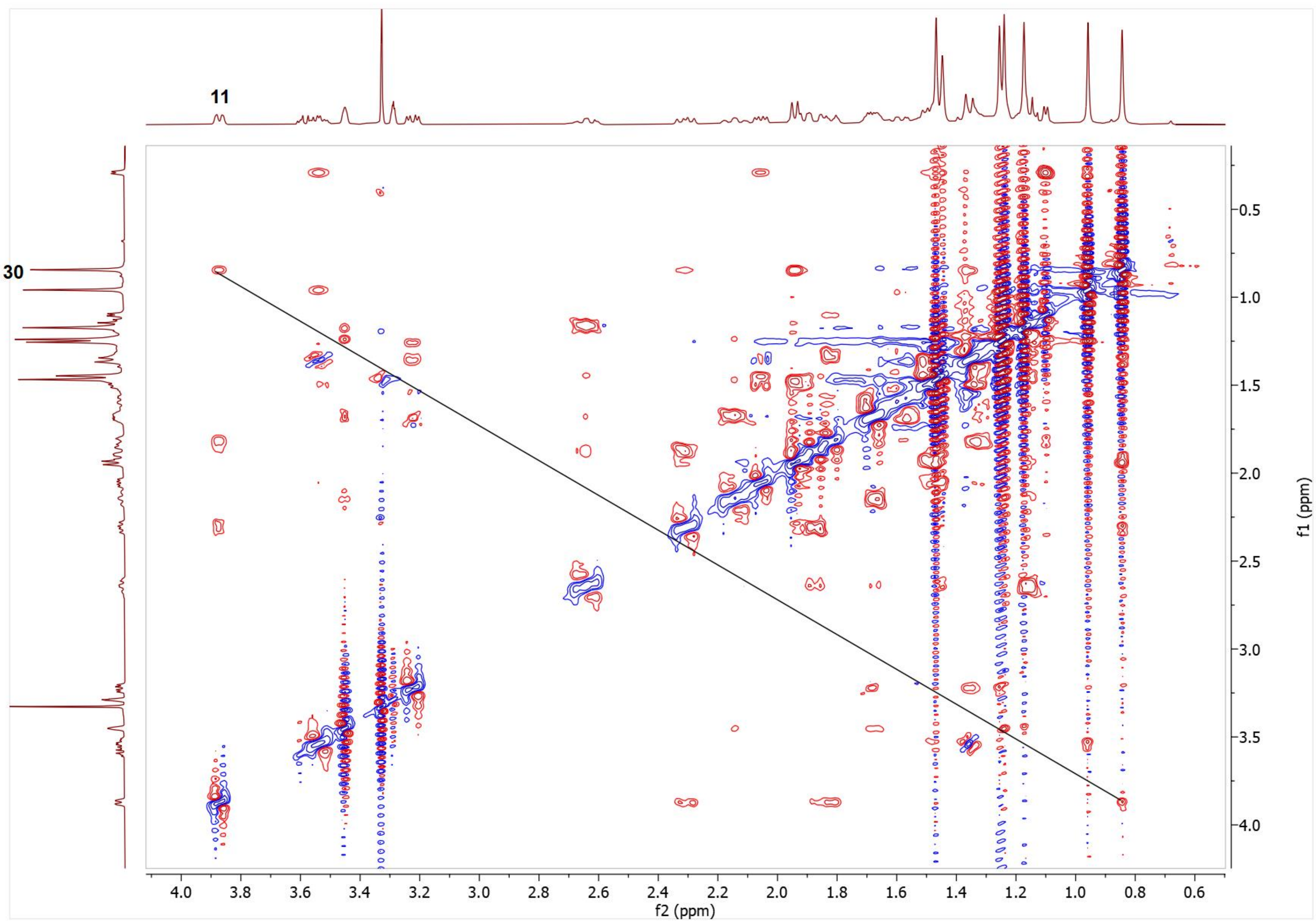

Figure S 116 NOESY spectrum of compound **13**

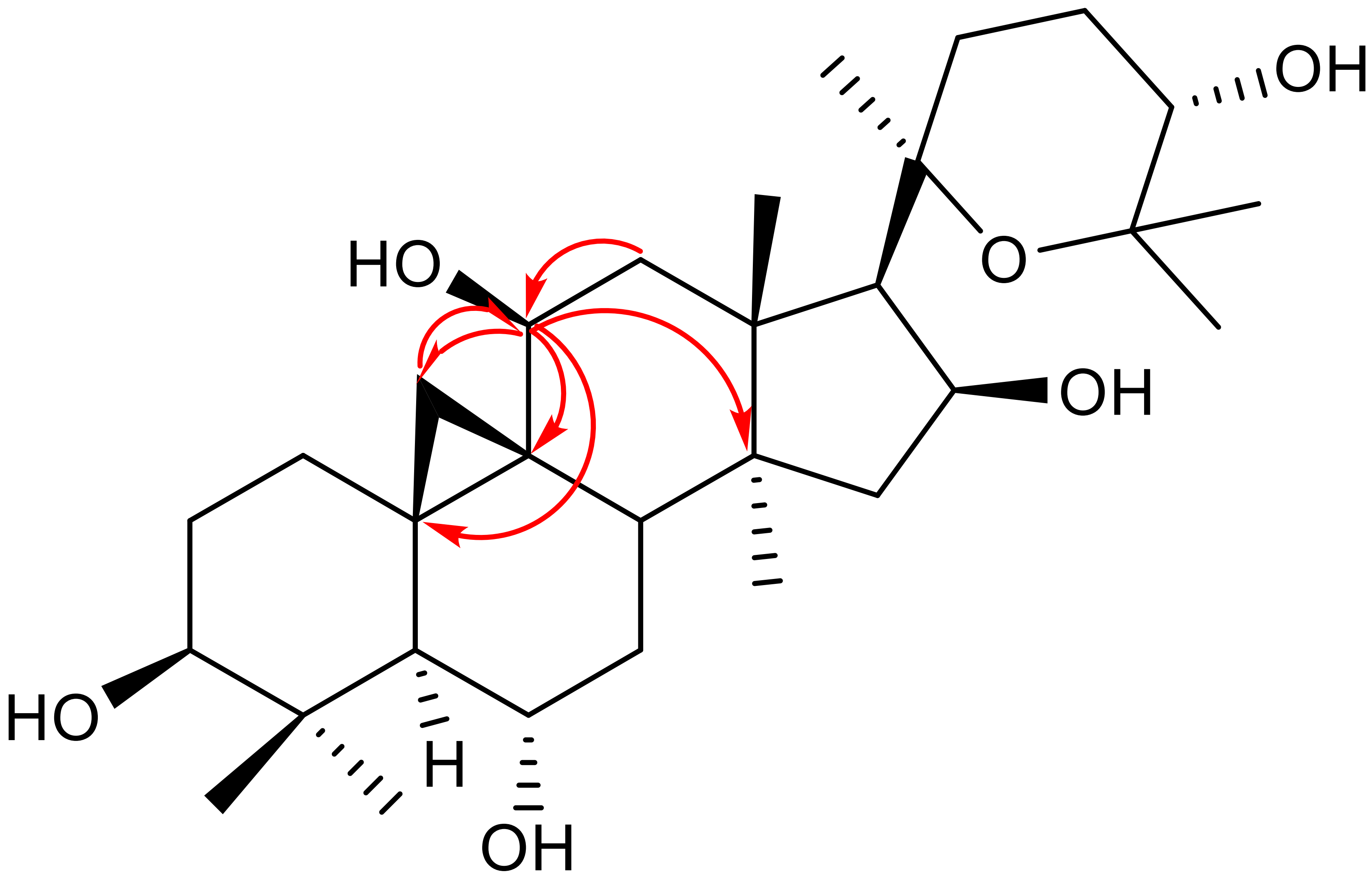

Figure S 117 Key HMBC correlations of compound **13** (arrows from H to C)

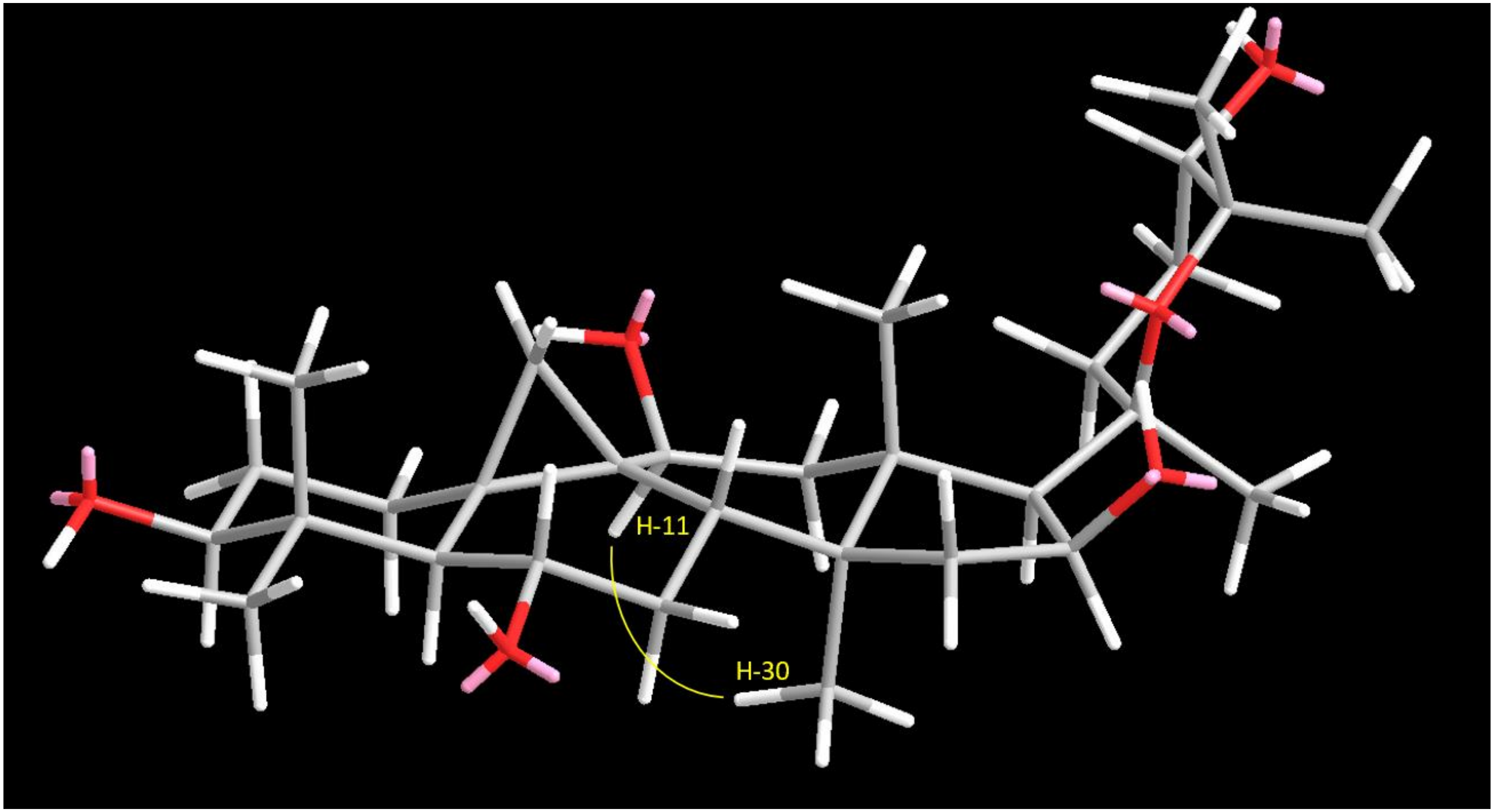

Figure S 118 Key NOE correlation of compound **13**

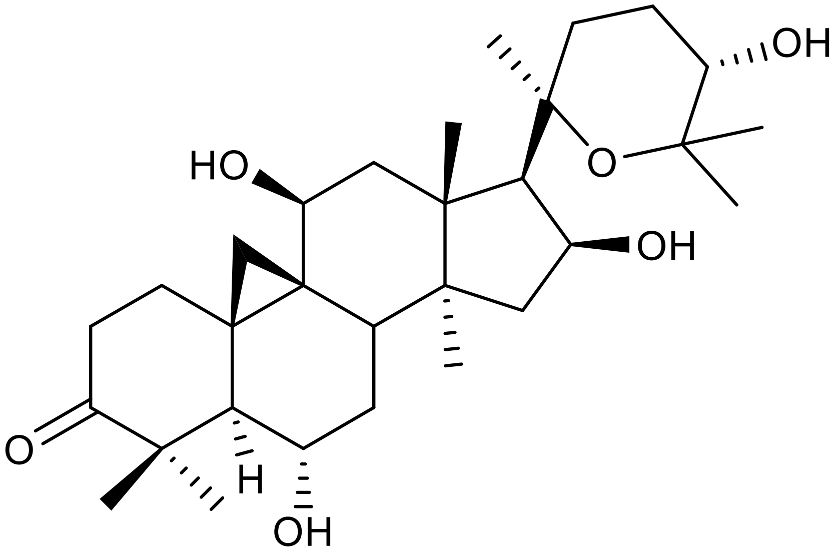

Figure S 119 Structure of compound **14**

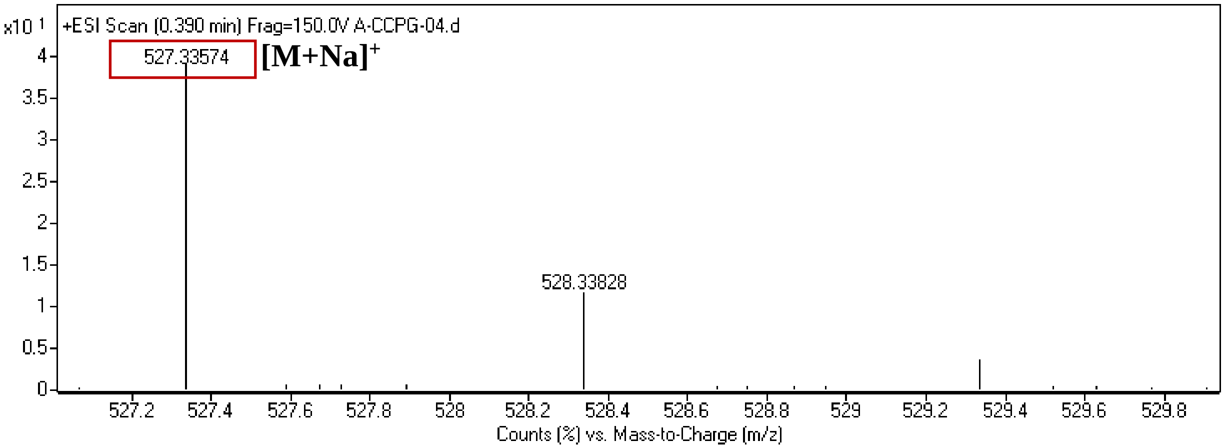

Figure S 120 HR-ESI-MS spectrum of compound **14**

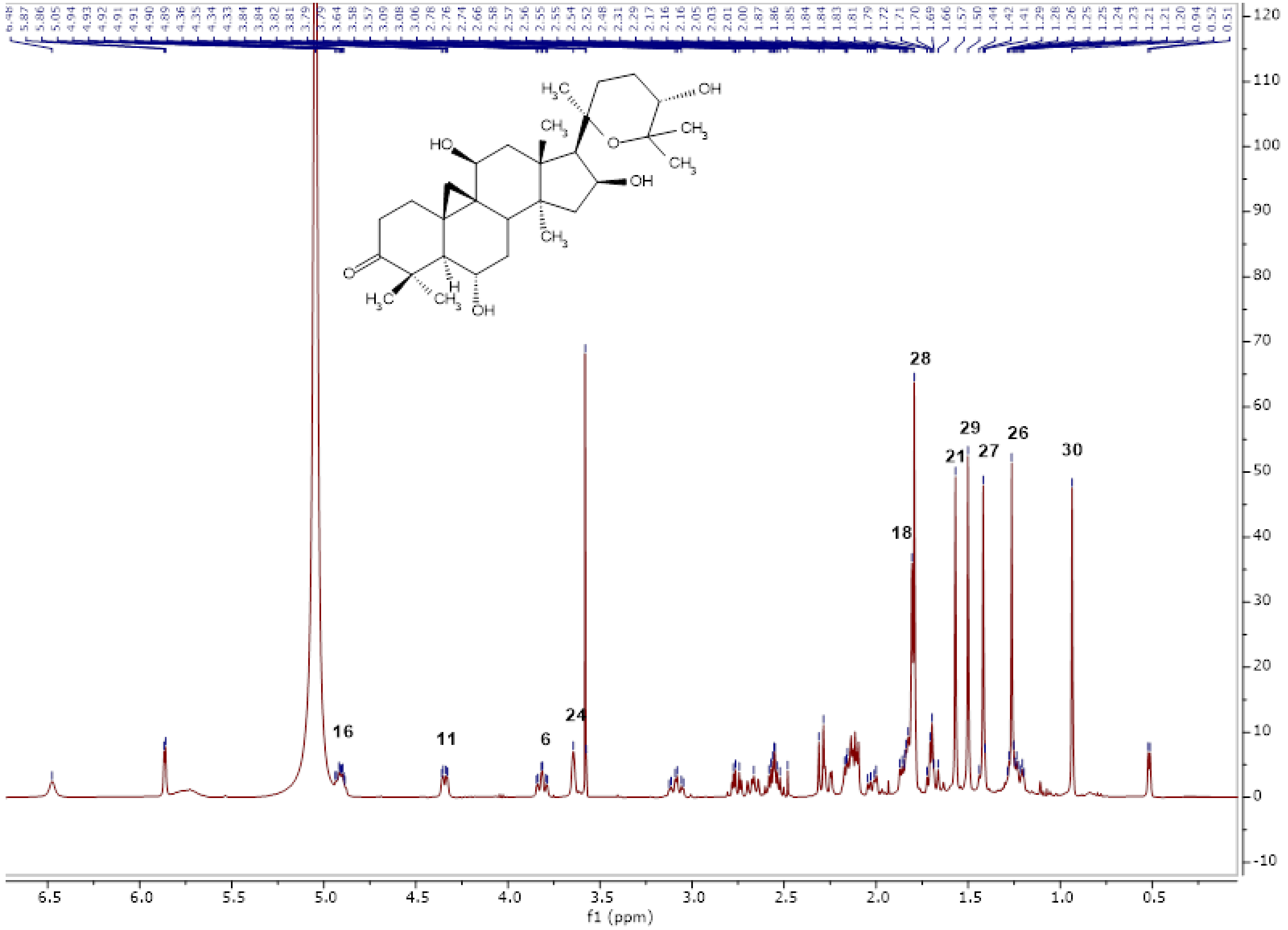

Figure S 121 ^{1}H-NMR spectrum of compound **14** (400 MHz, C_5D_5N)

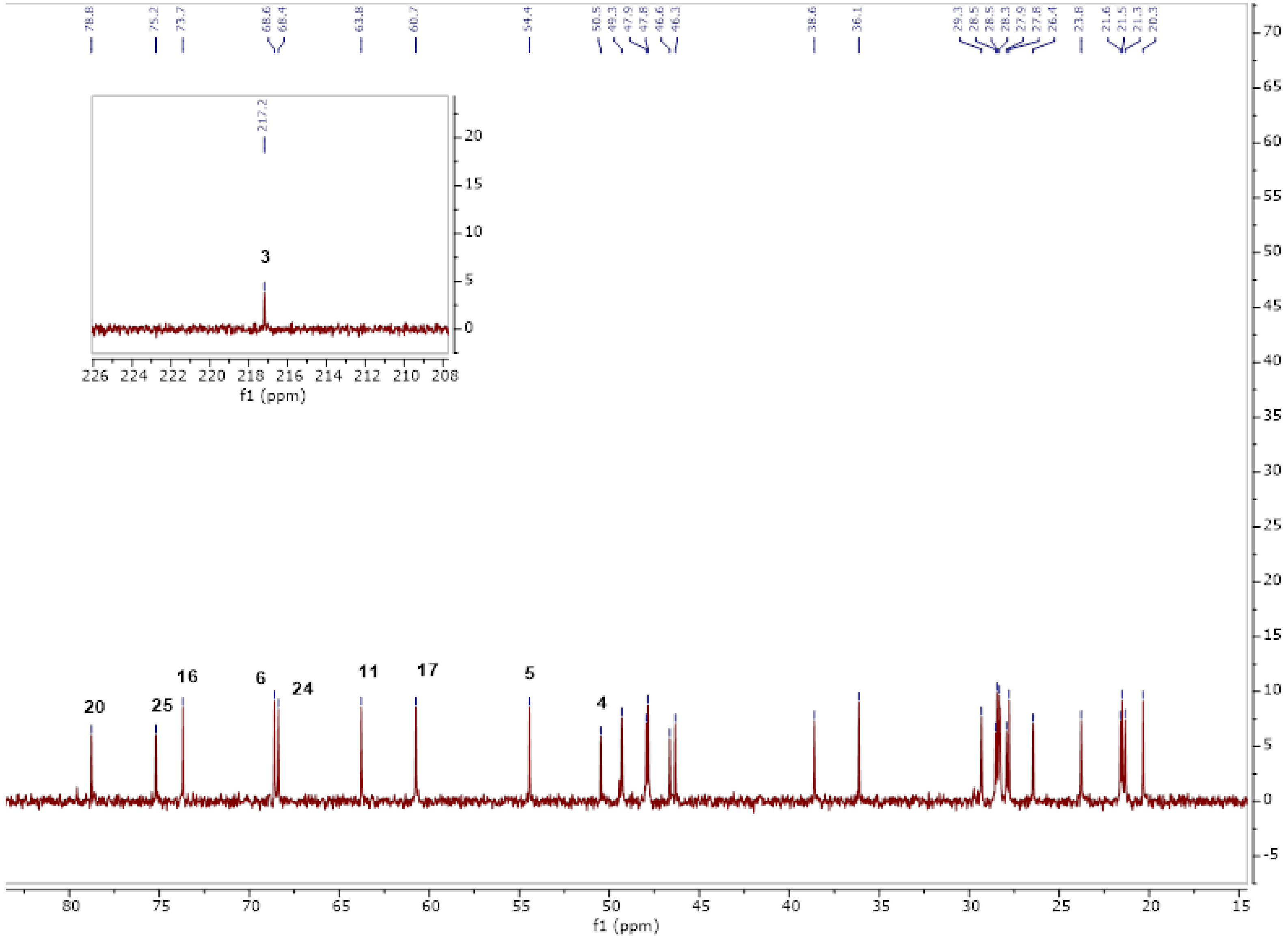

Figure S 122 ^{13}C-NMR spectrum of compound **14** (100 MHz, C_5D_5N)

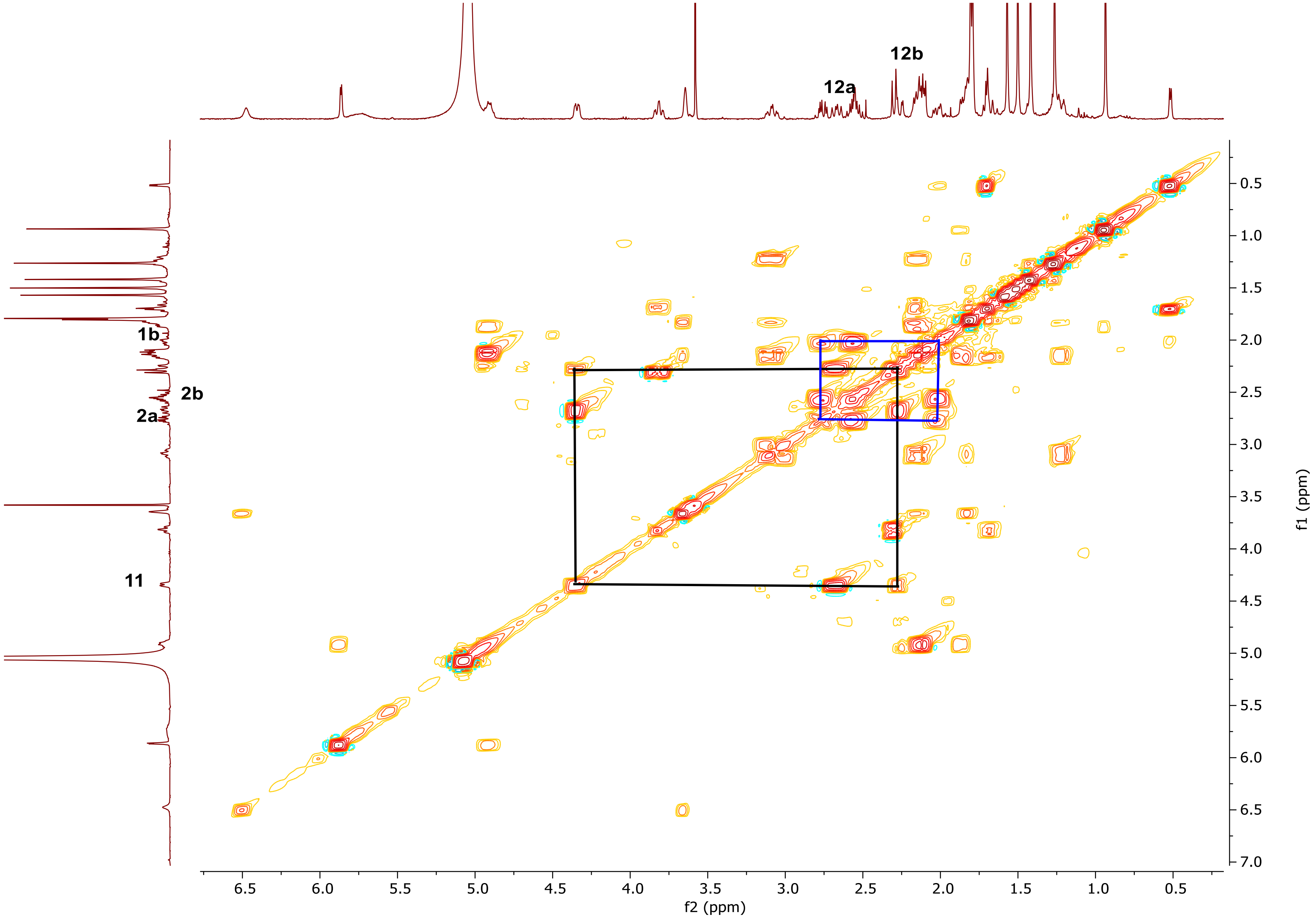

Figure S 123 COSY spectrum of compound **14**

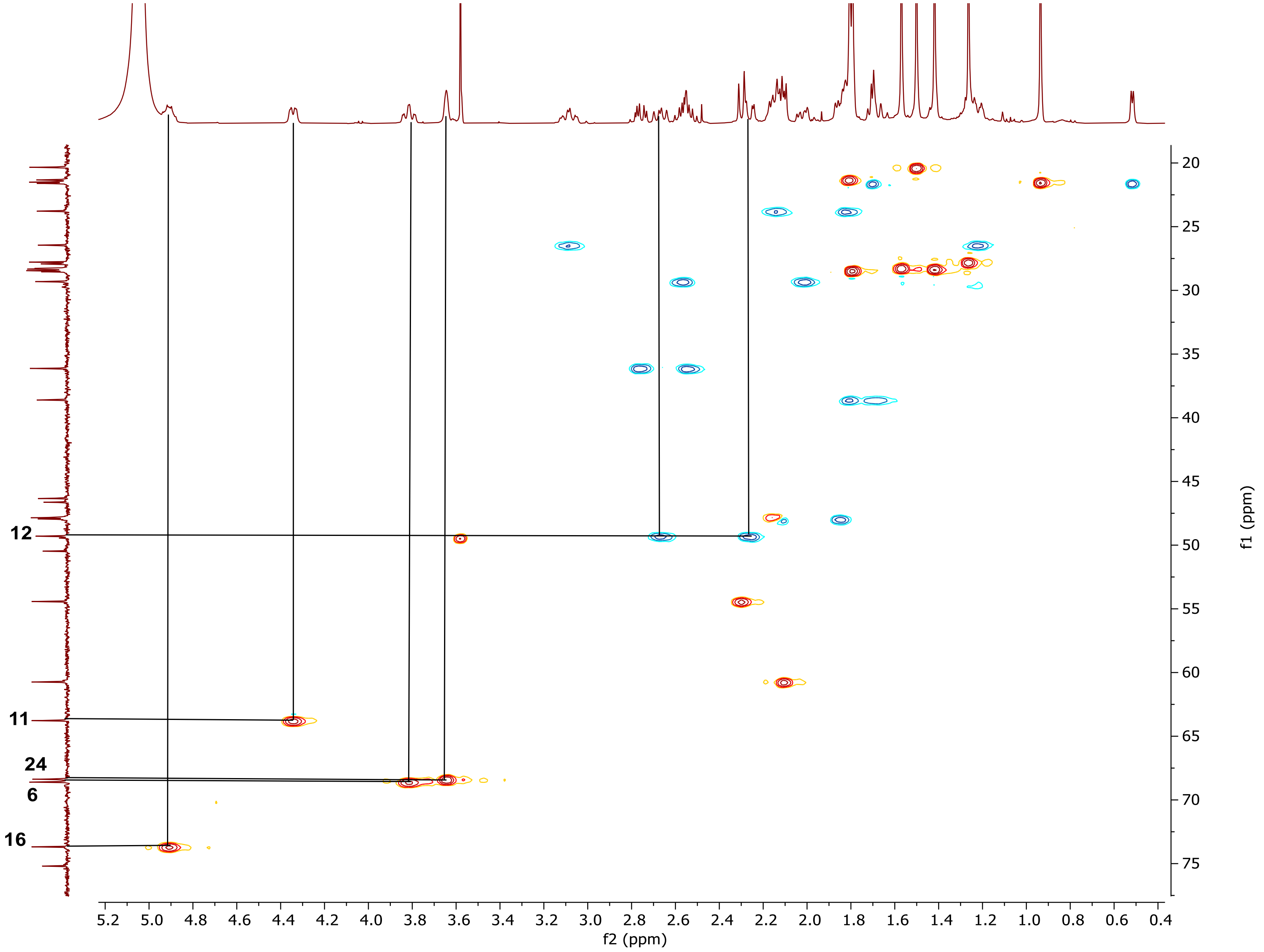

Figure S 124 HSQC spectrum of compound **14**

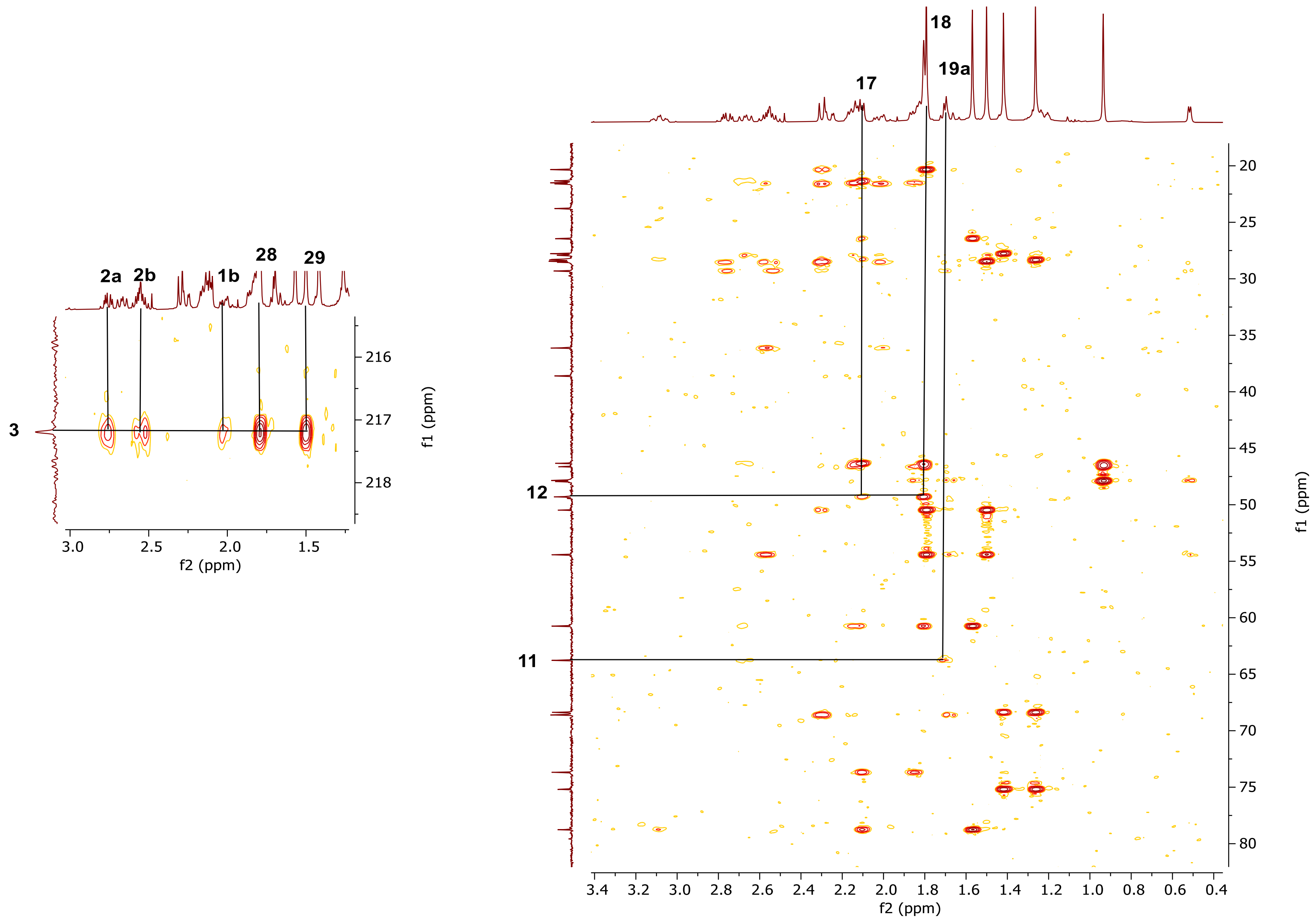

Figure S 125 HMBC spectrum of compound **14**

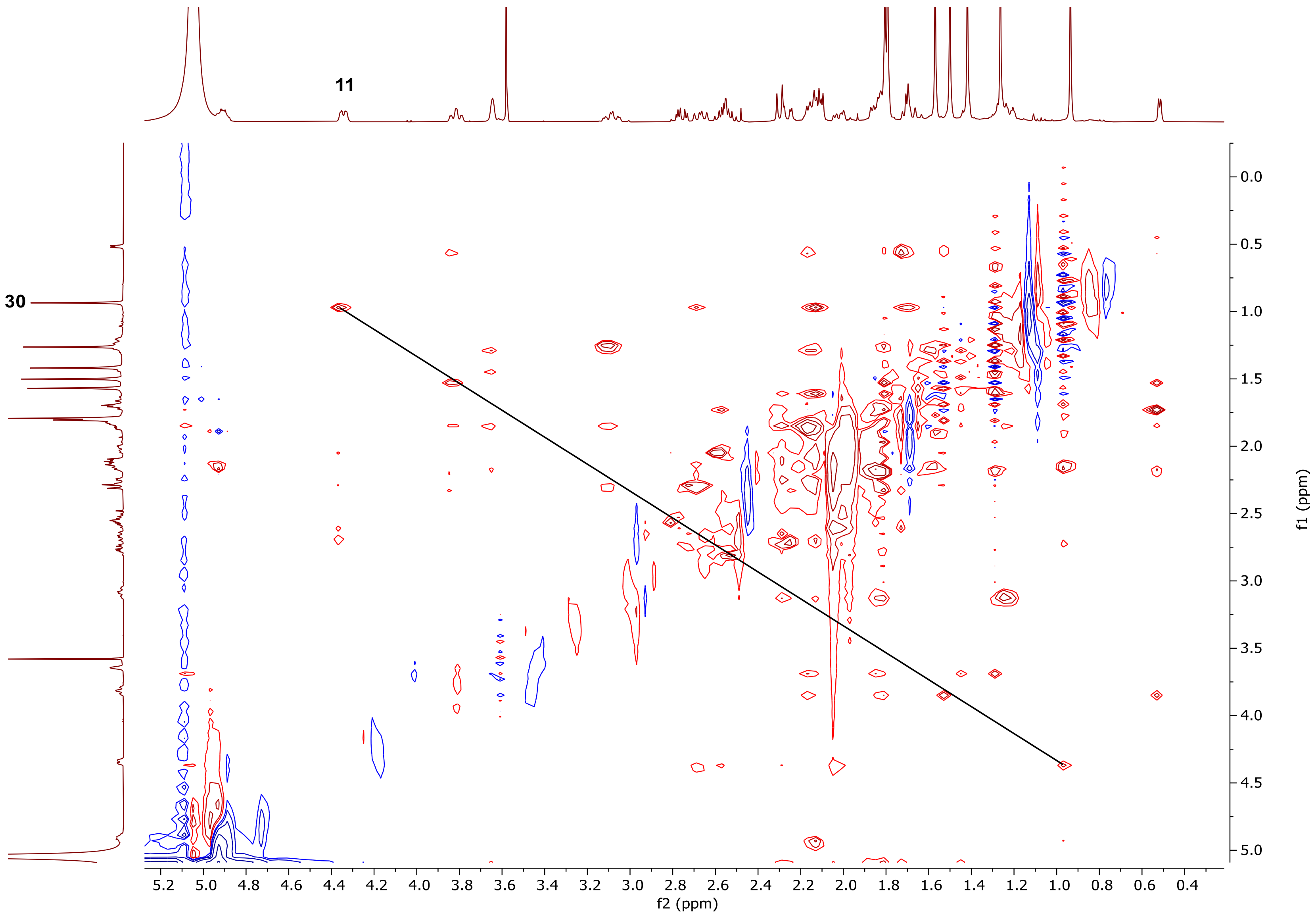

Figure S 126 NOESY spectrum of compound **14**

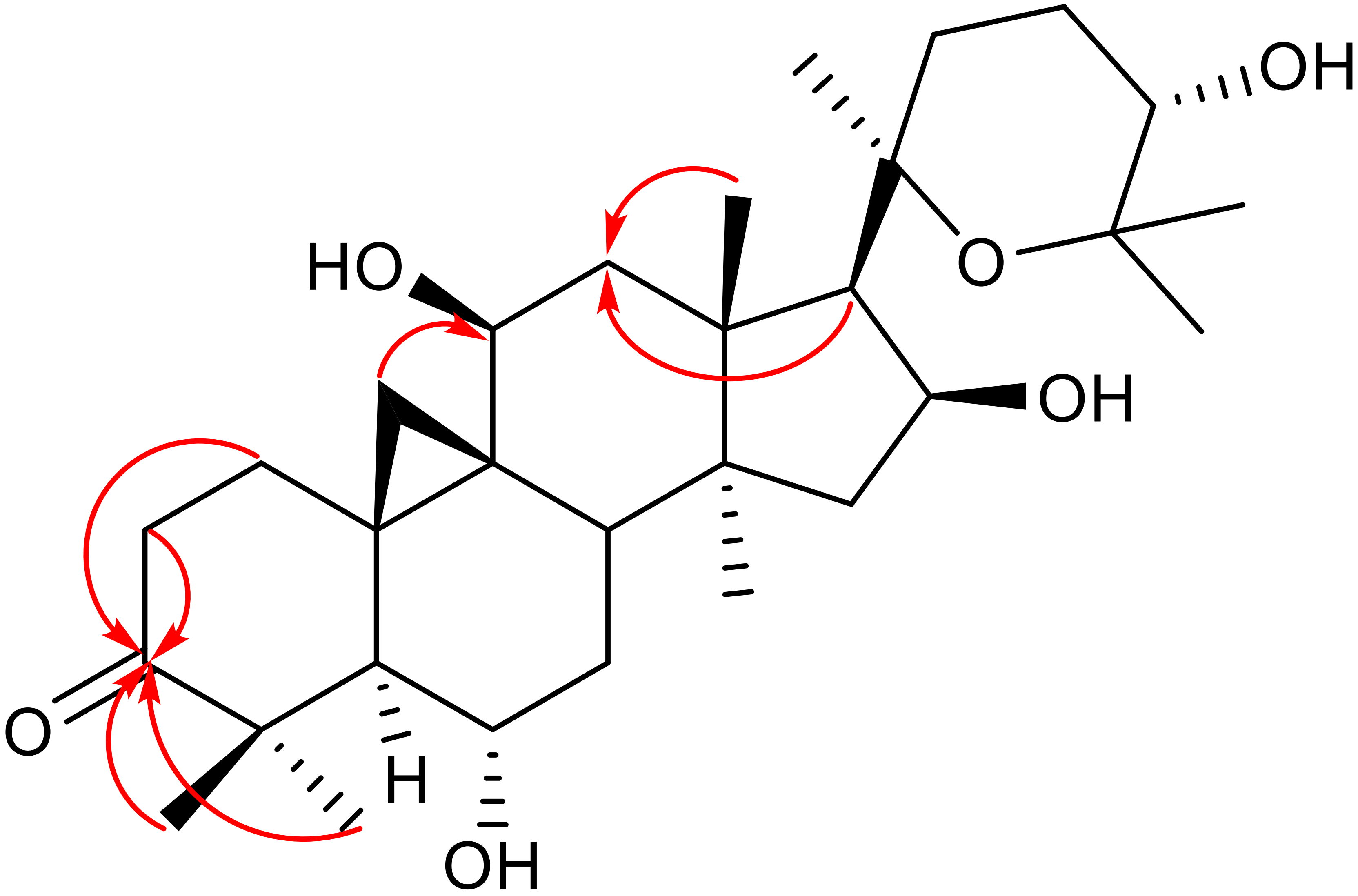

Figure S 127 Key HMBC correlations of compound **14** (arrows from H to C)

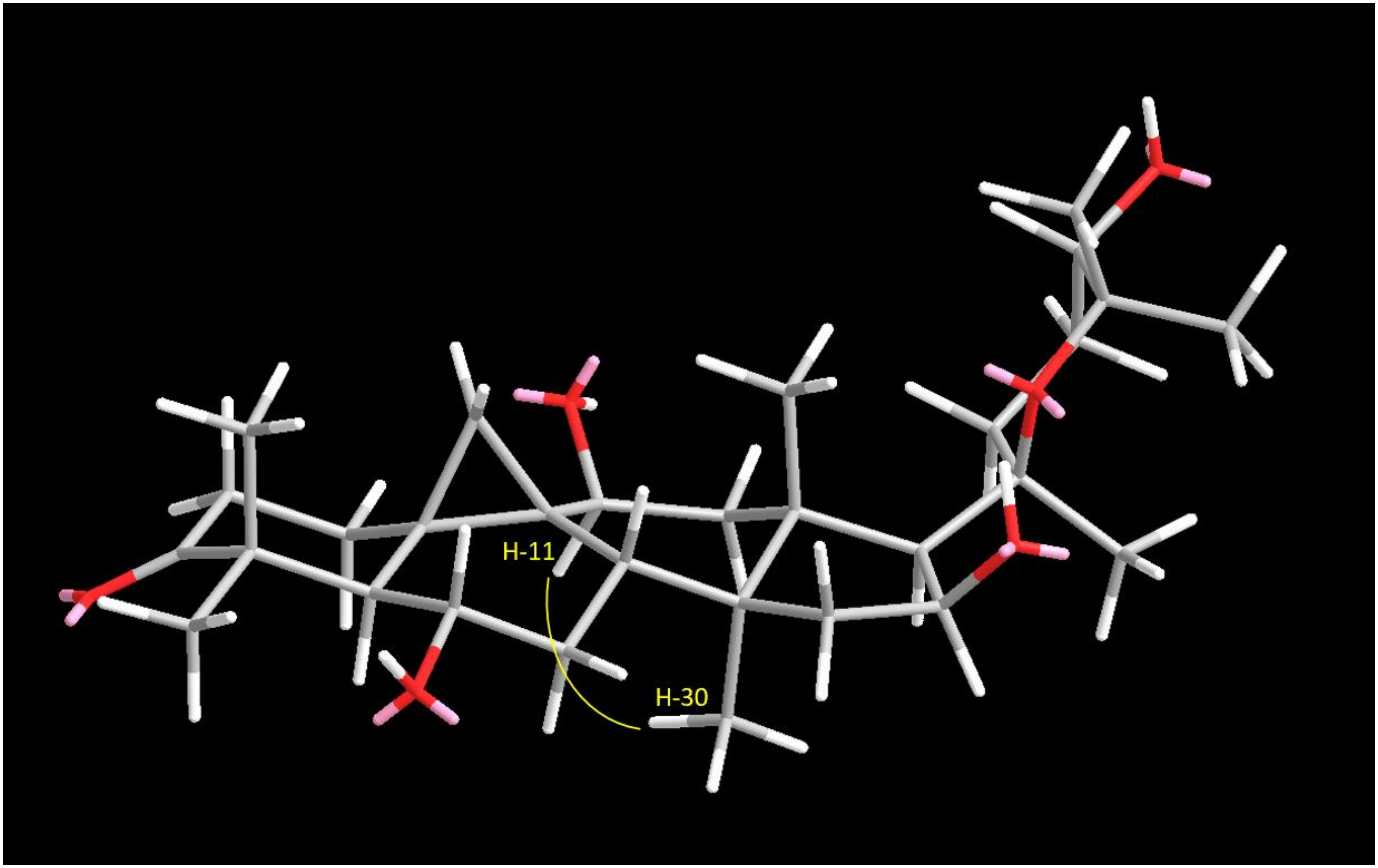

Figure S 128 Key NOE correlation of compound **14**

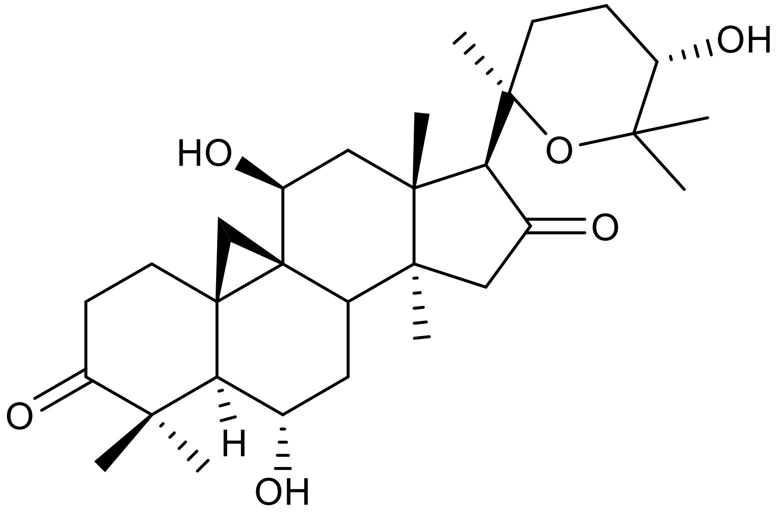

Figure S 129 Structure of compound **15**

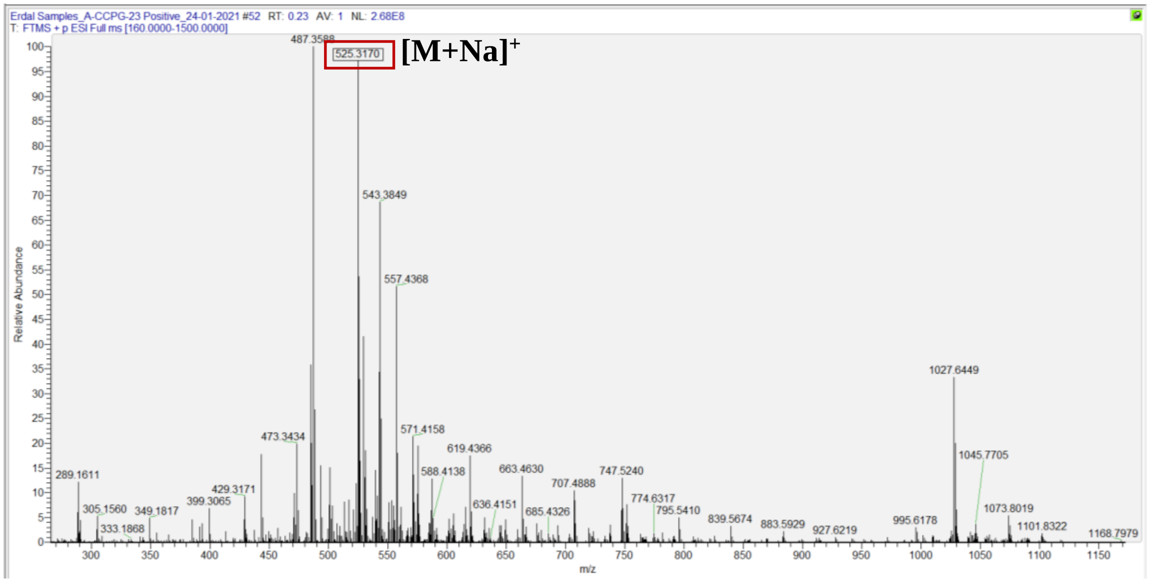

Figure S 130 HR-ESI-MS spectrum of compound **15**

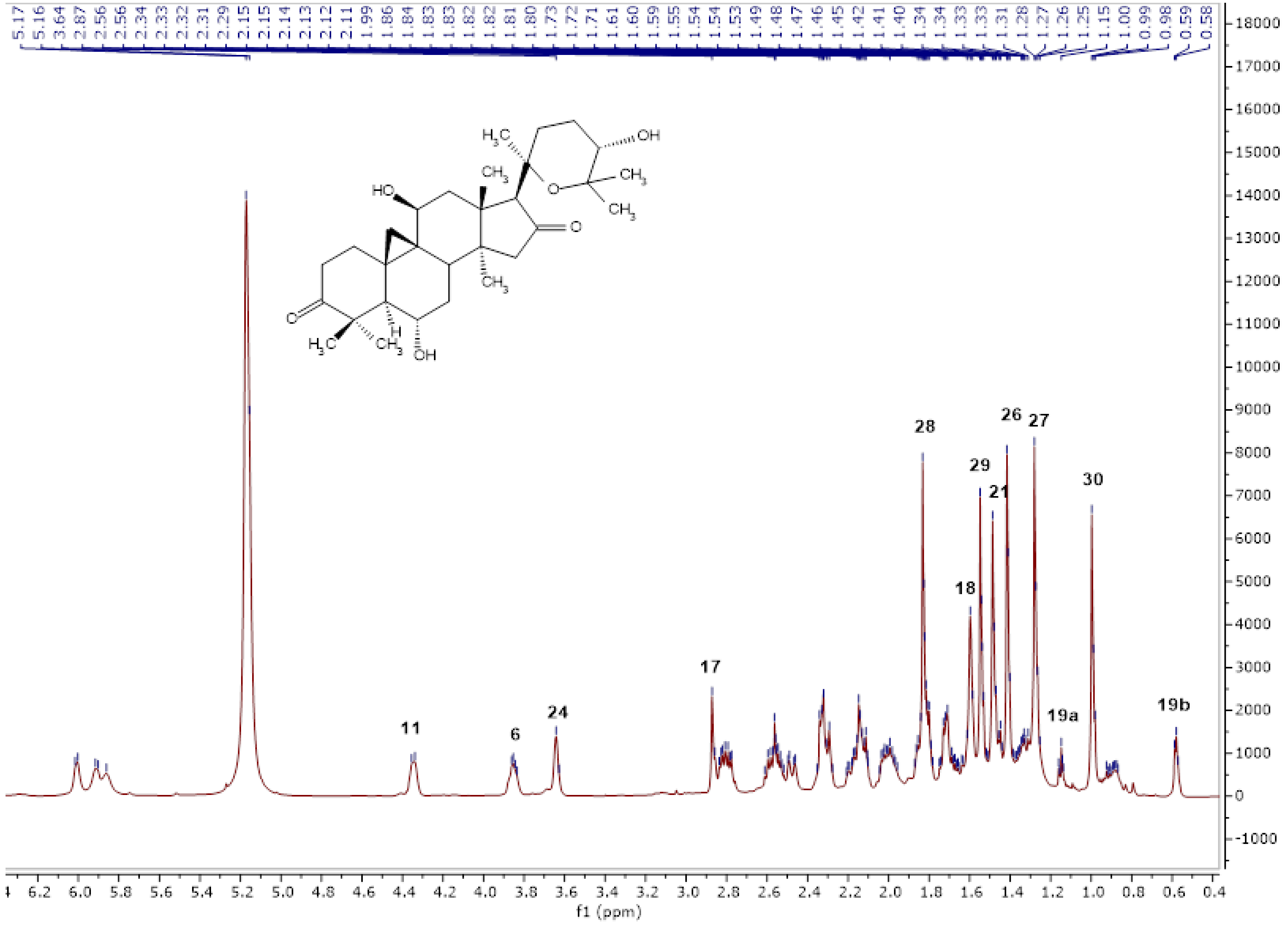

Figure S 131 ^{1}H-NMR spectrum of compound **15** (500 MHz, C_5D_5N)

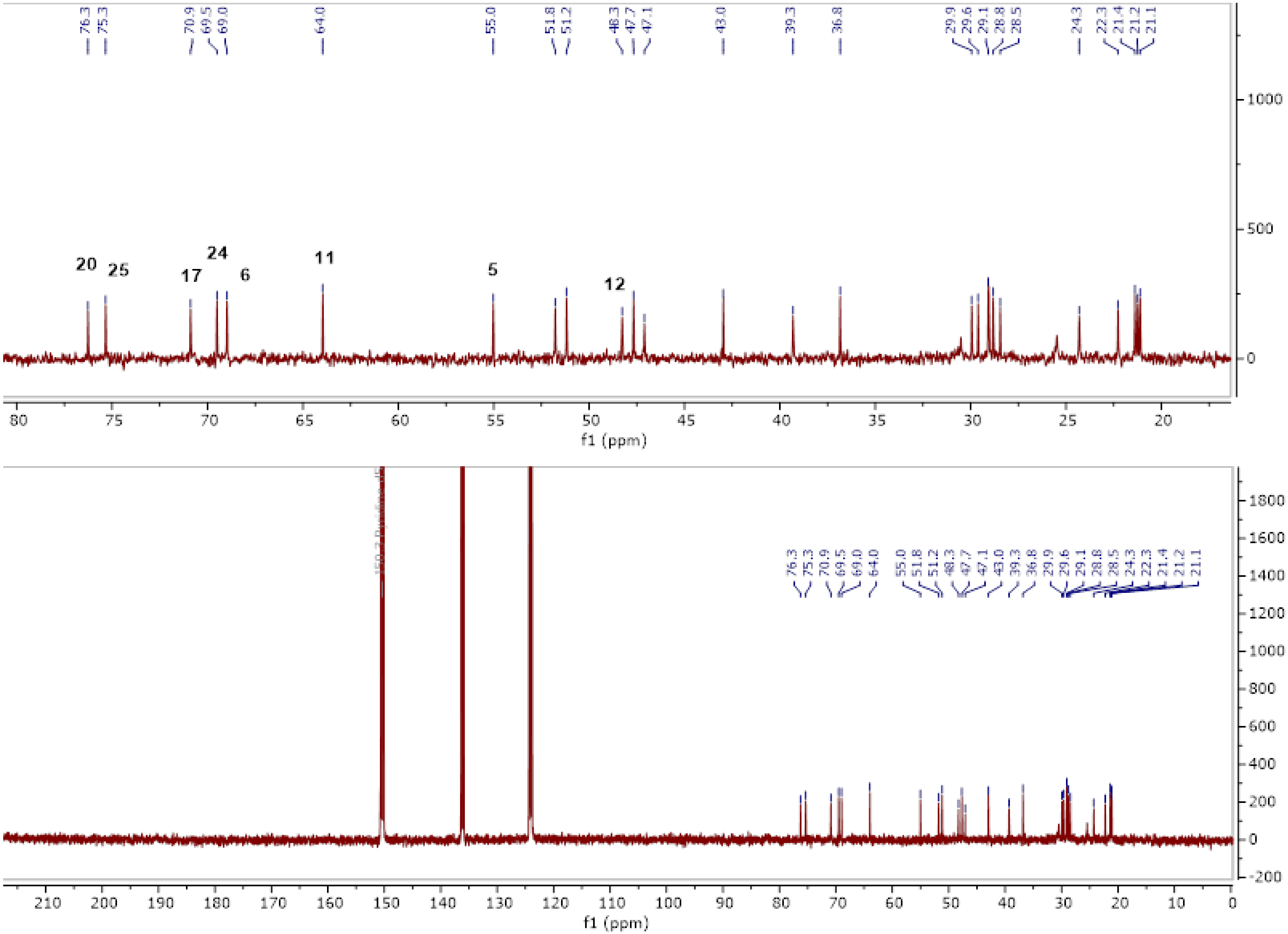

Figure S 132 ^{13}C-NMR spectrum of compound **15** (125 MHz, C_5D_5N)

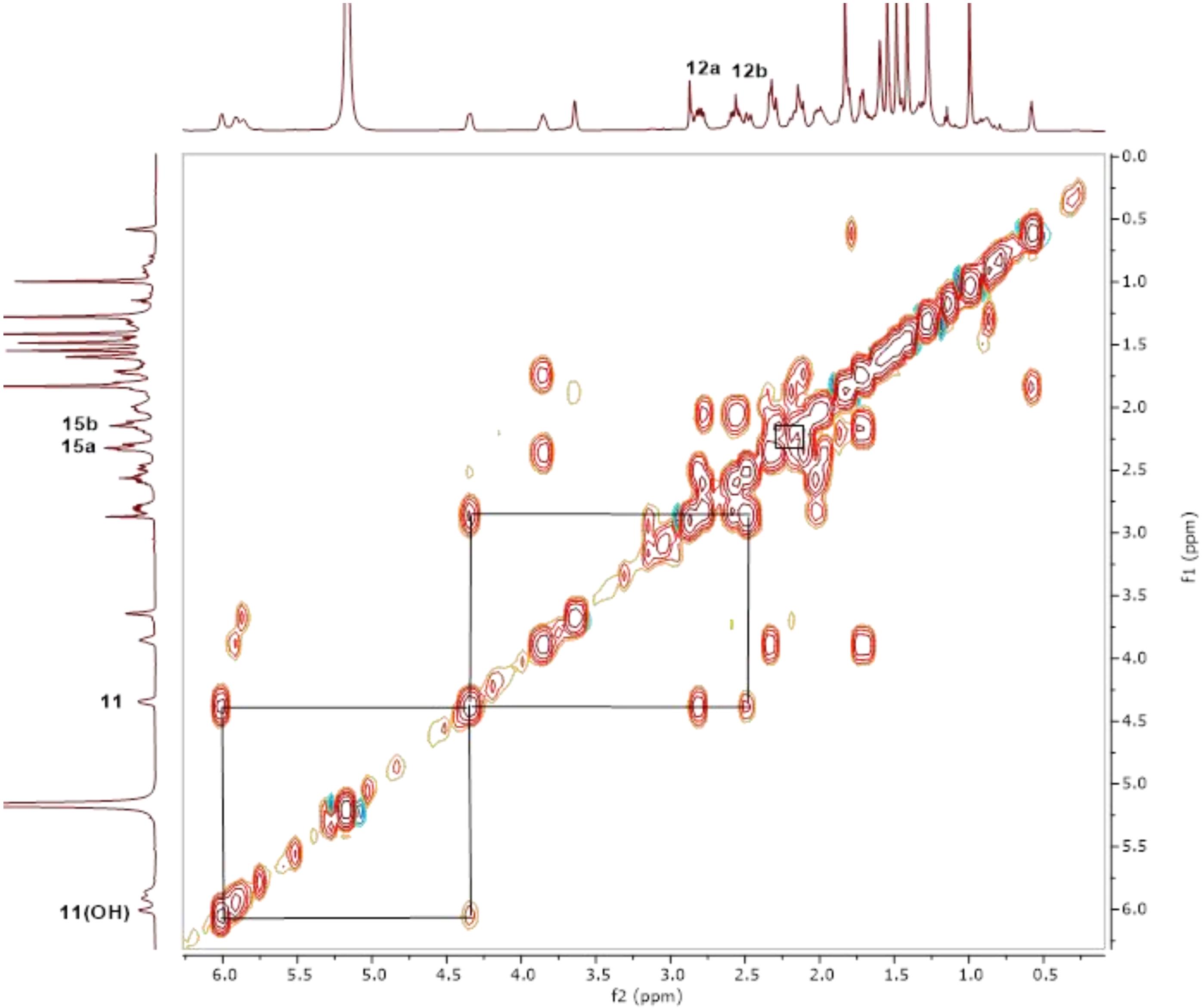

Figure S 133 COSY spectrum of compound **15**

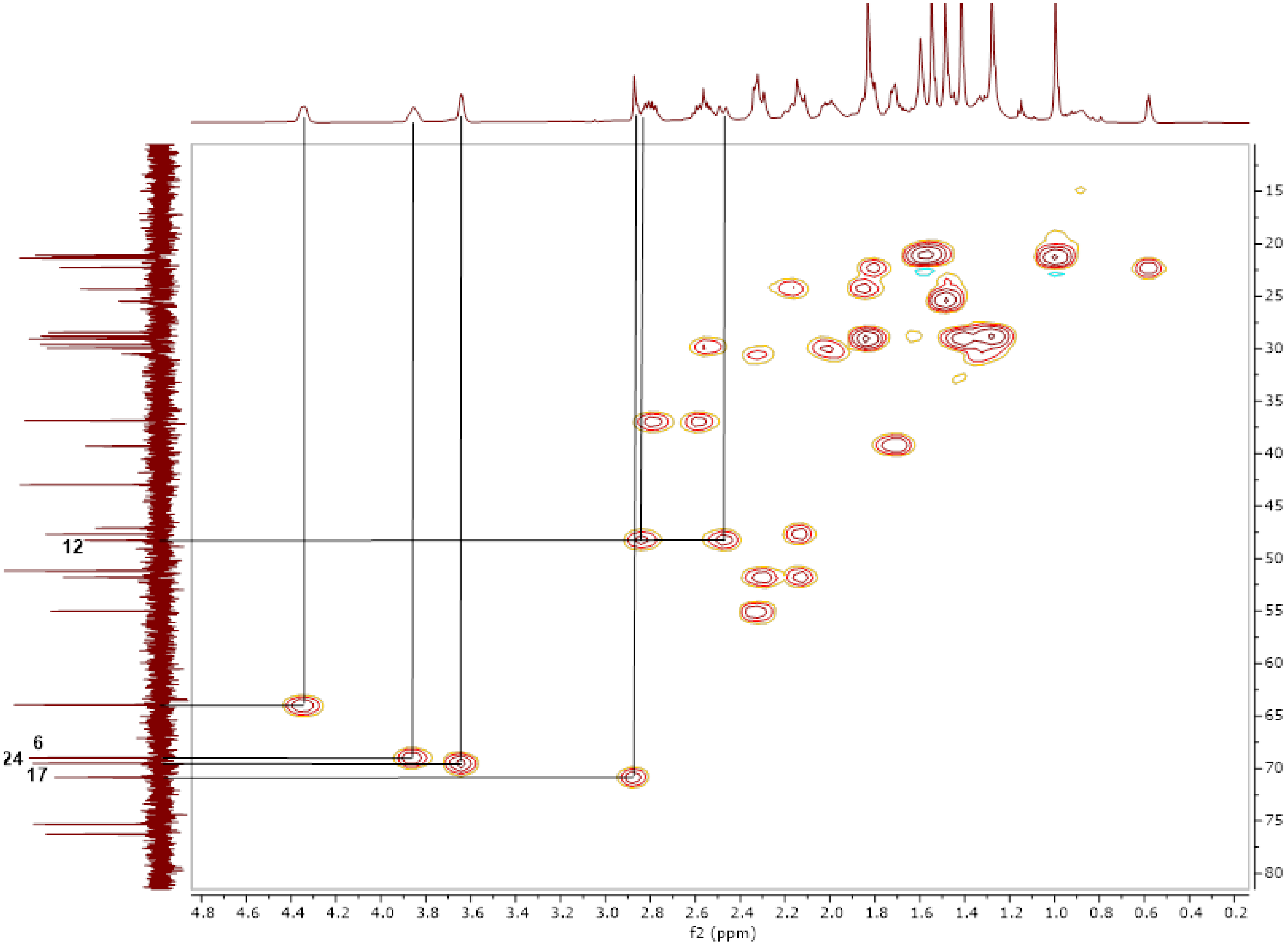

Figure S 134 HSQC spectrum of compound **15**

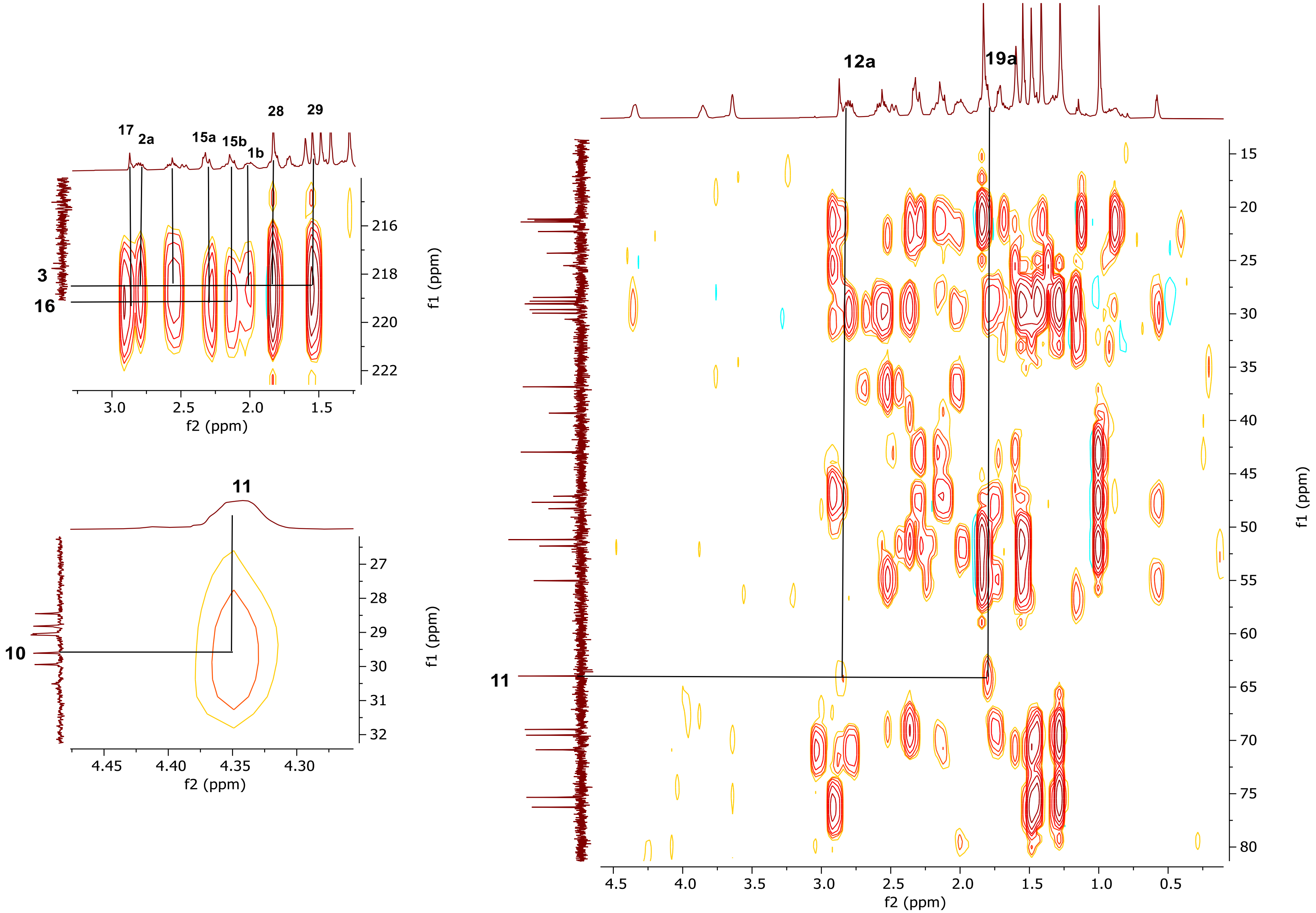

Figure S 135 HMBC spectrum of compound **15**

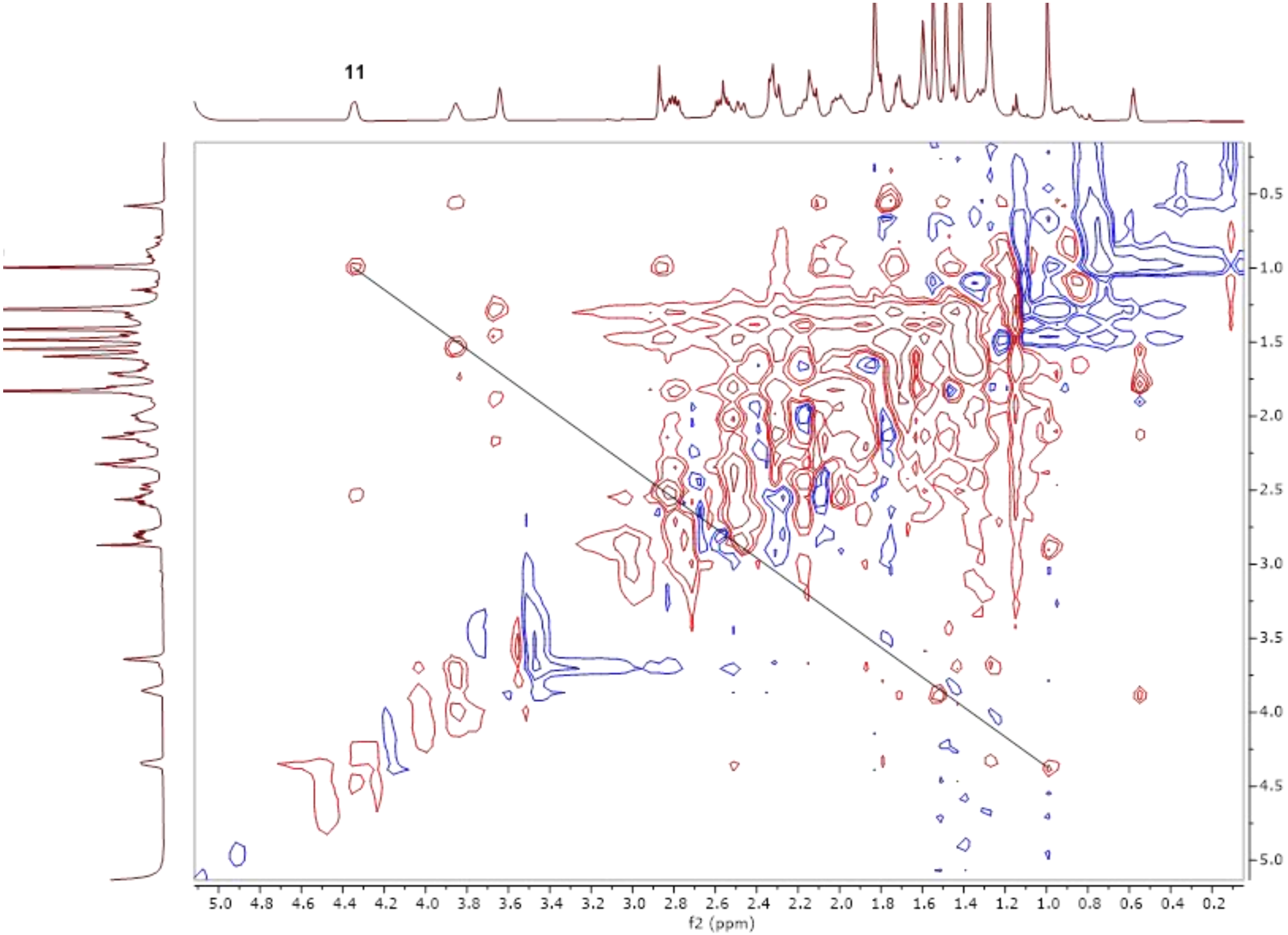

Figure S 136 NOESY spectrum of compound **15**

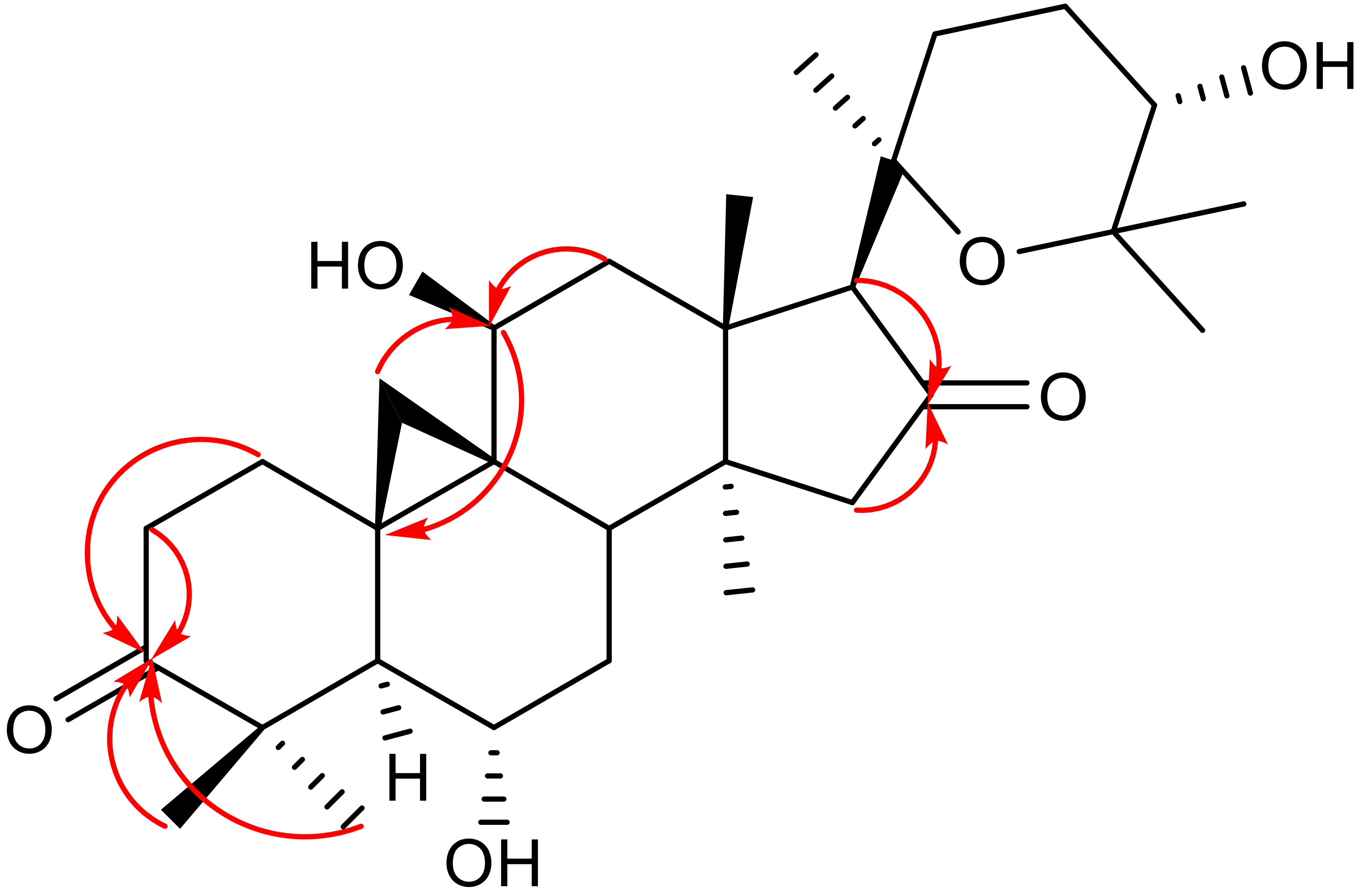

Figure S 137 Key HMBC correlations of compound **15** (arrows from H to C)

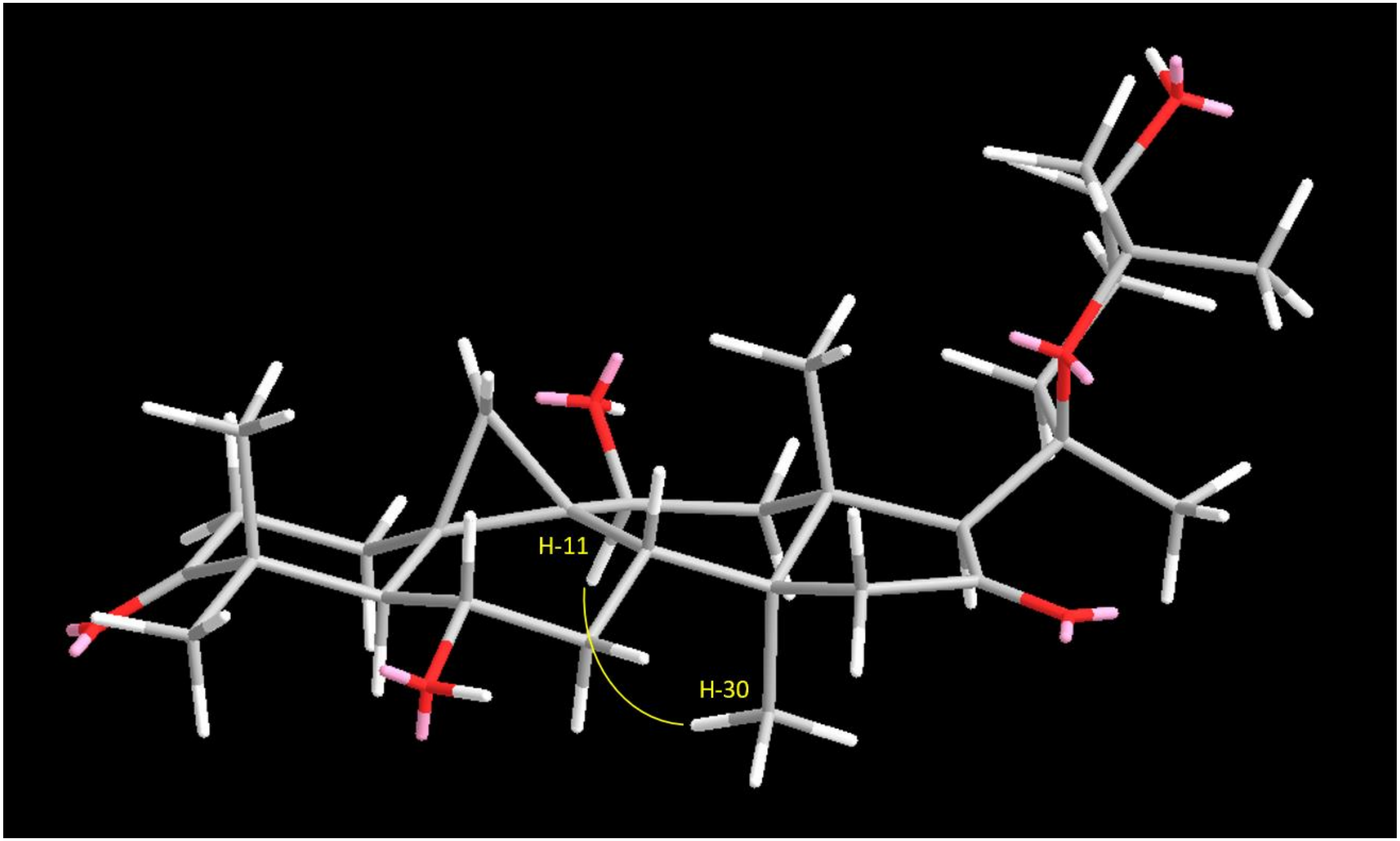

Figure S 138 Key NOE correlation of compound **15**

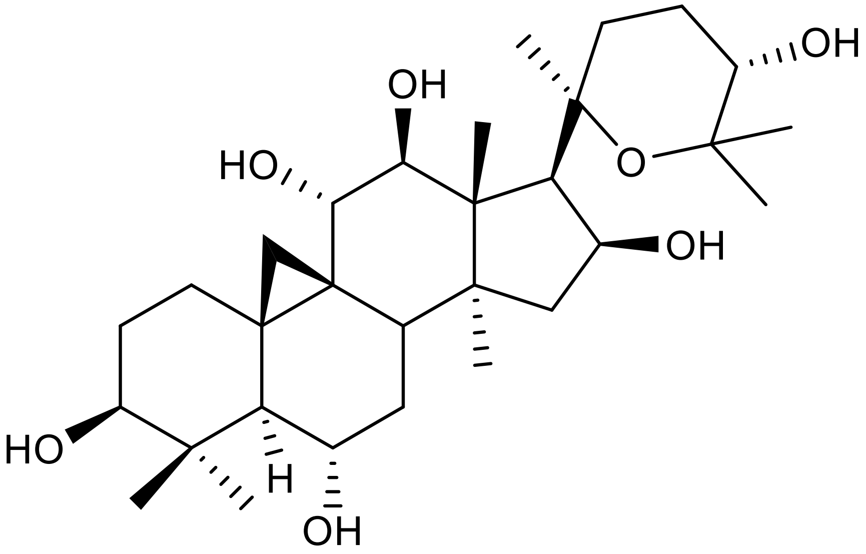

Figure S 139 Structure of compound **16**

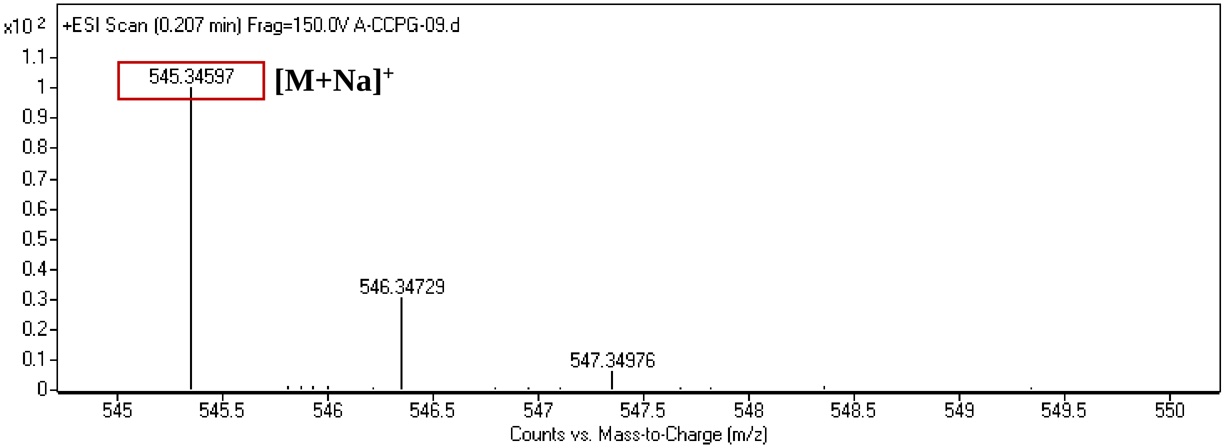

Figure S 140 HR-ESI-MS spectrum of compound **16**

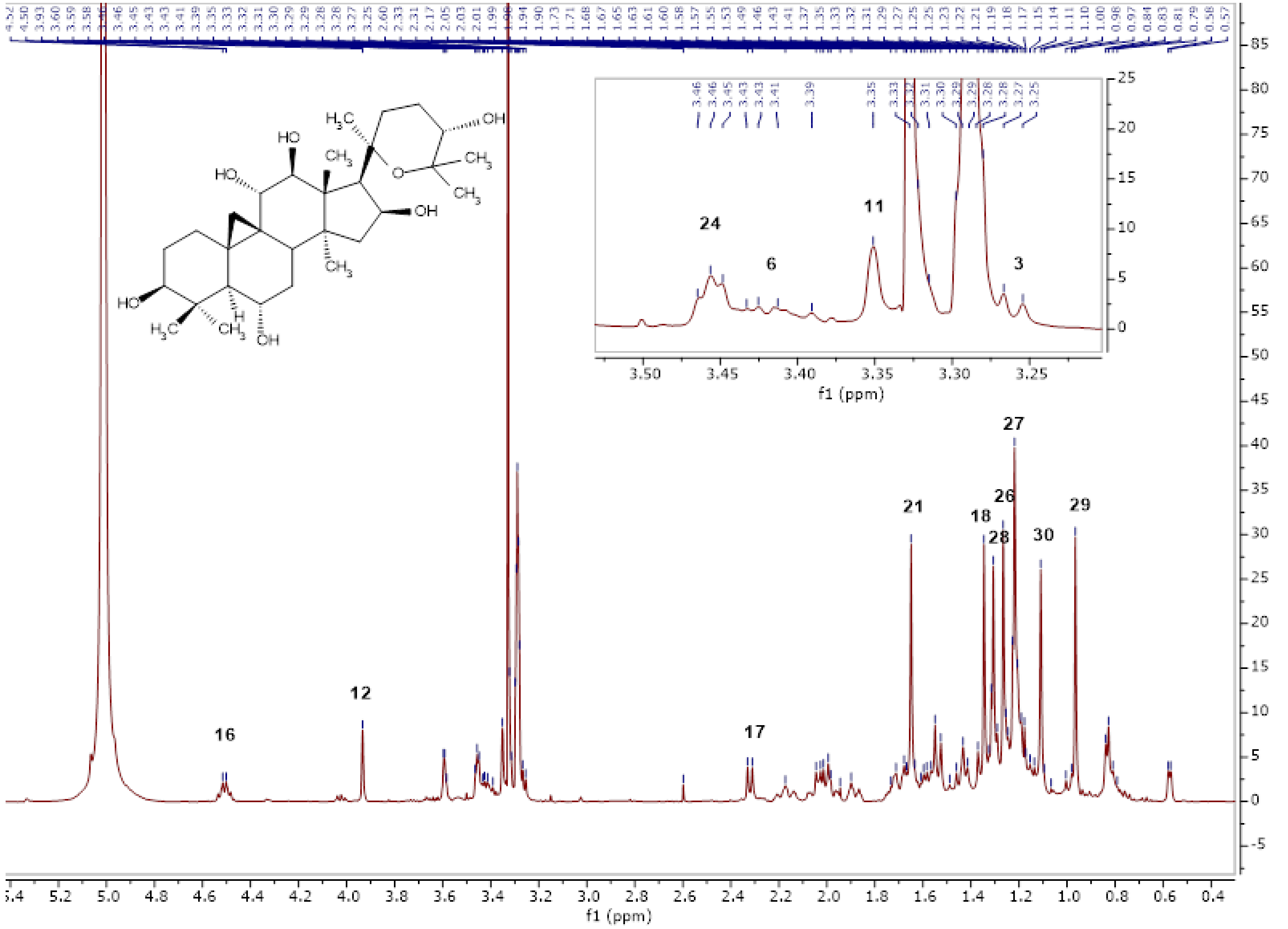

Figure S 141 ^{1}H-NMR spectrum of compound **16** (400 MHz, CD_3OD and a drop of C_5D_5N)

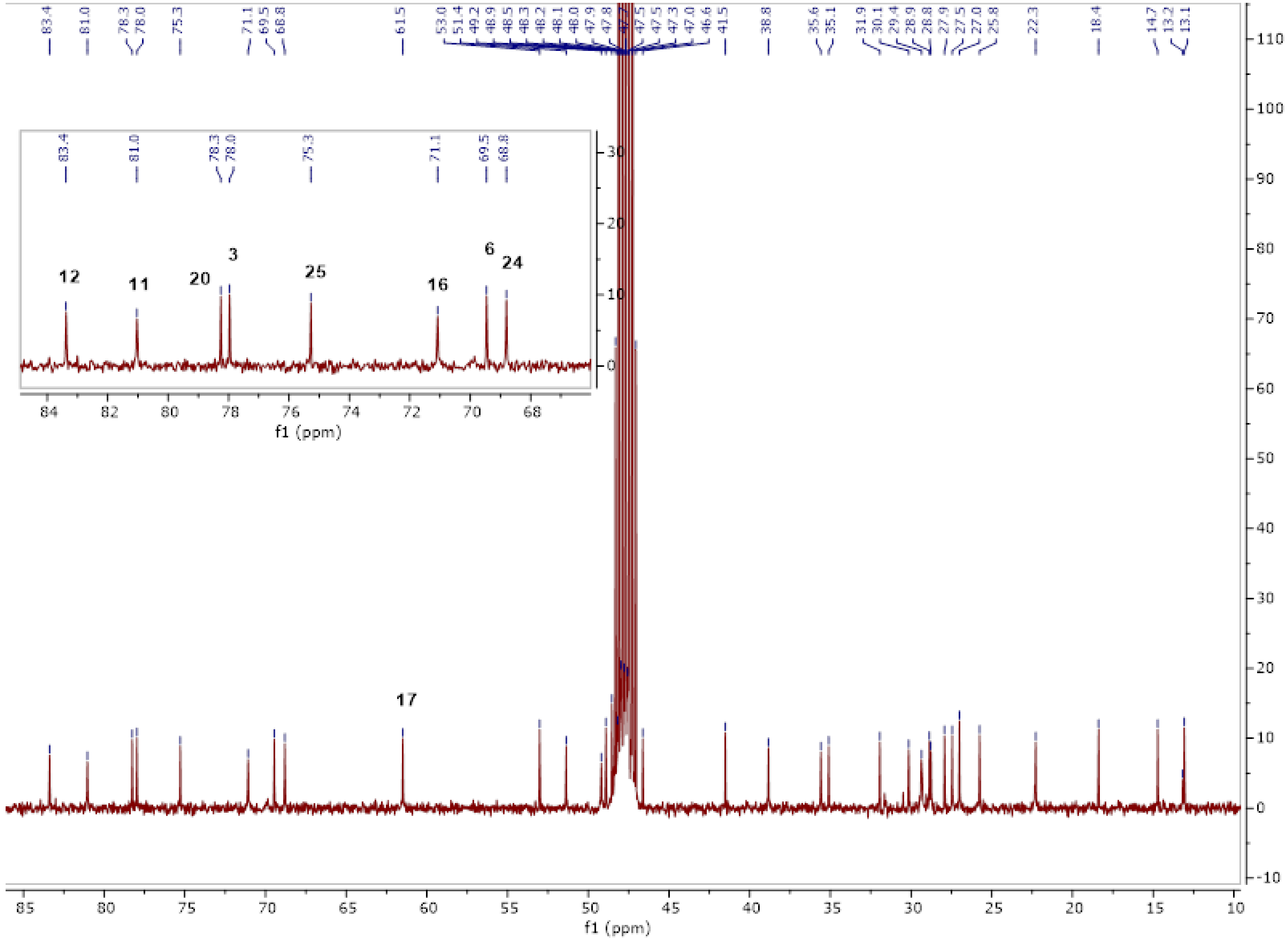

Figure S 142 ^{13}C-NMR spectrum of compound **16** (100 MHz, CD_3OD and a drop of C_5D_5N)

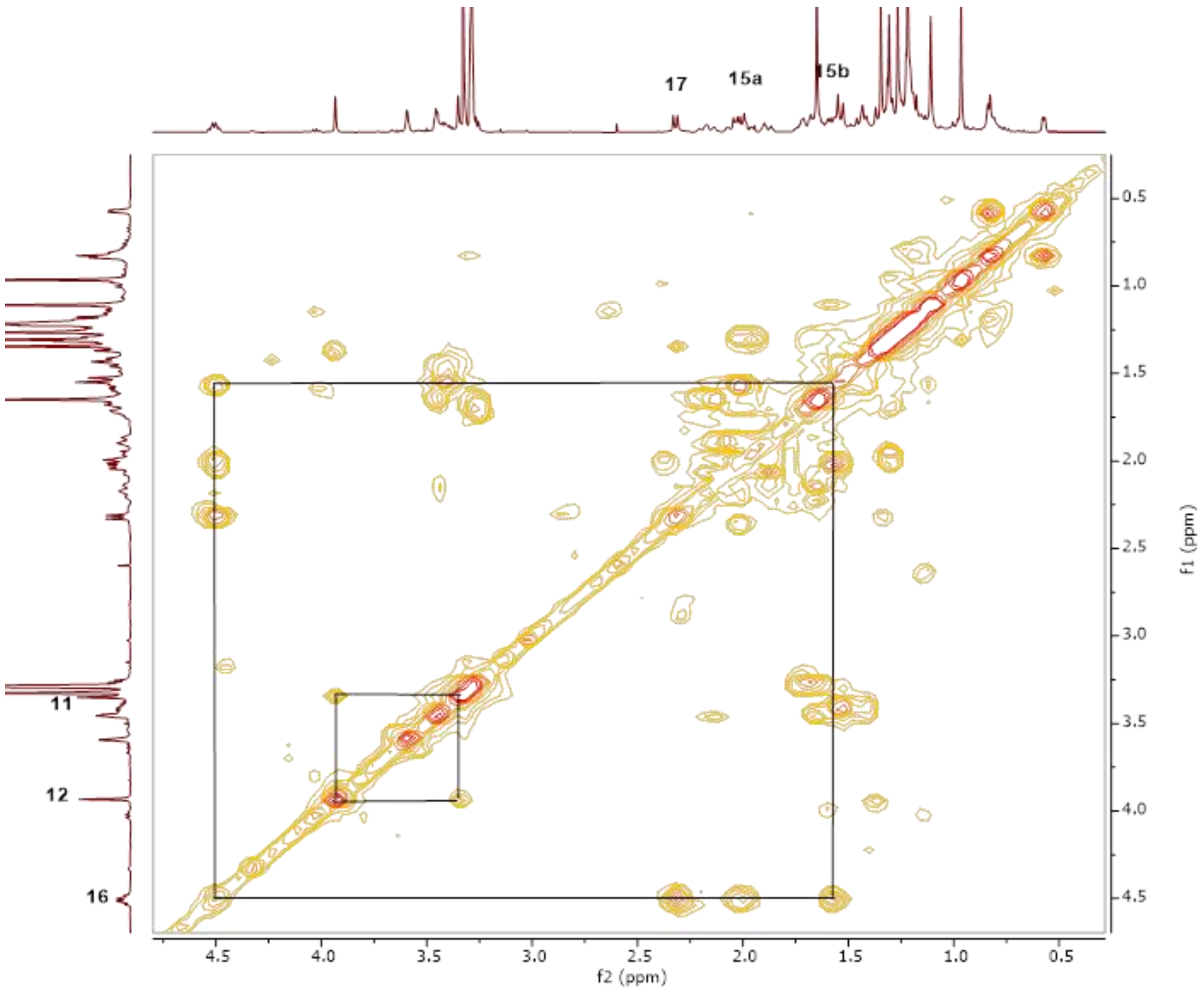

Figure S 143 COSY spectrum of compound **16**

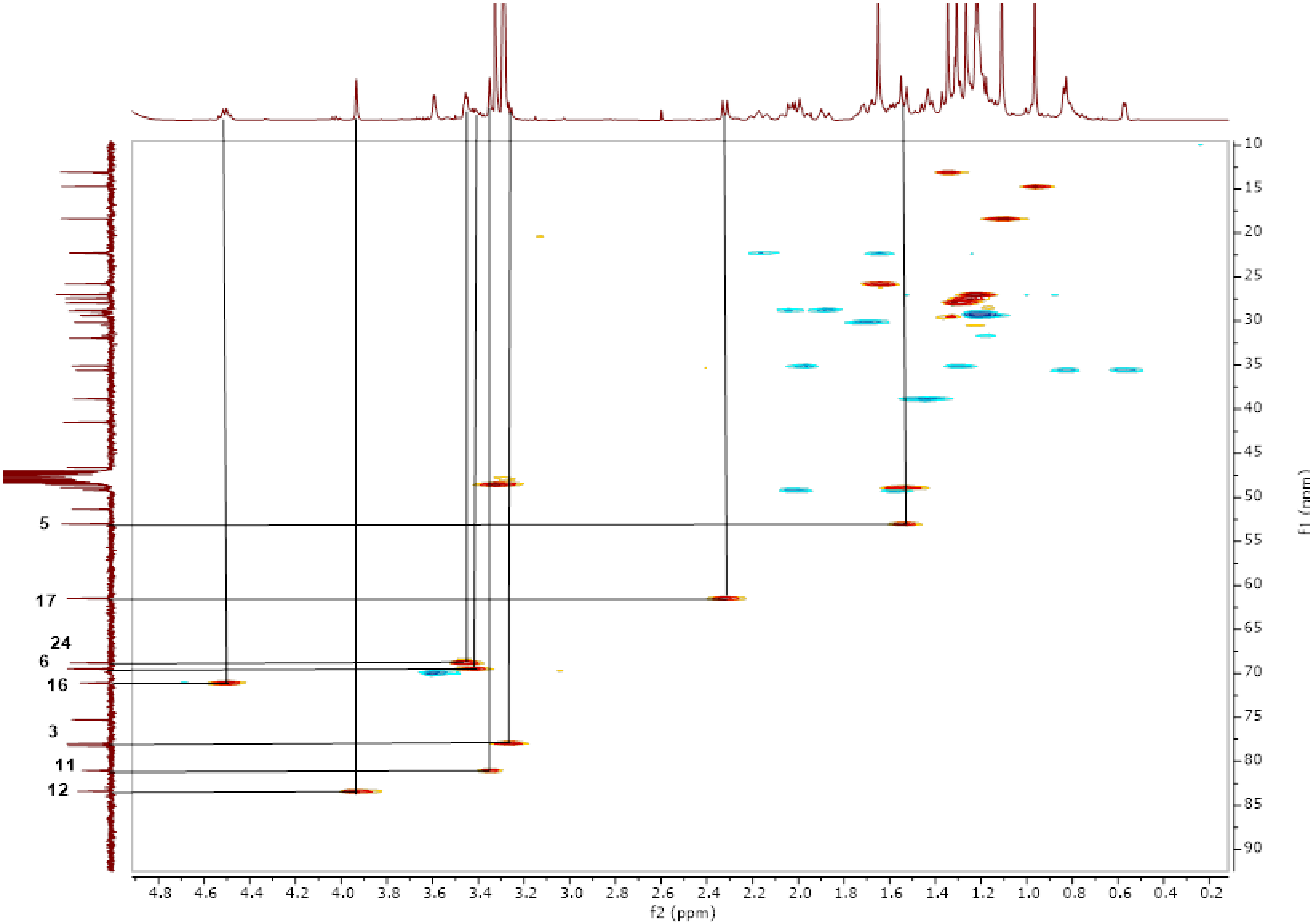

Figure S 144 HSQC spectrum of compound **16**

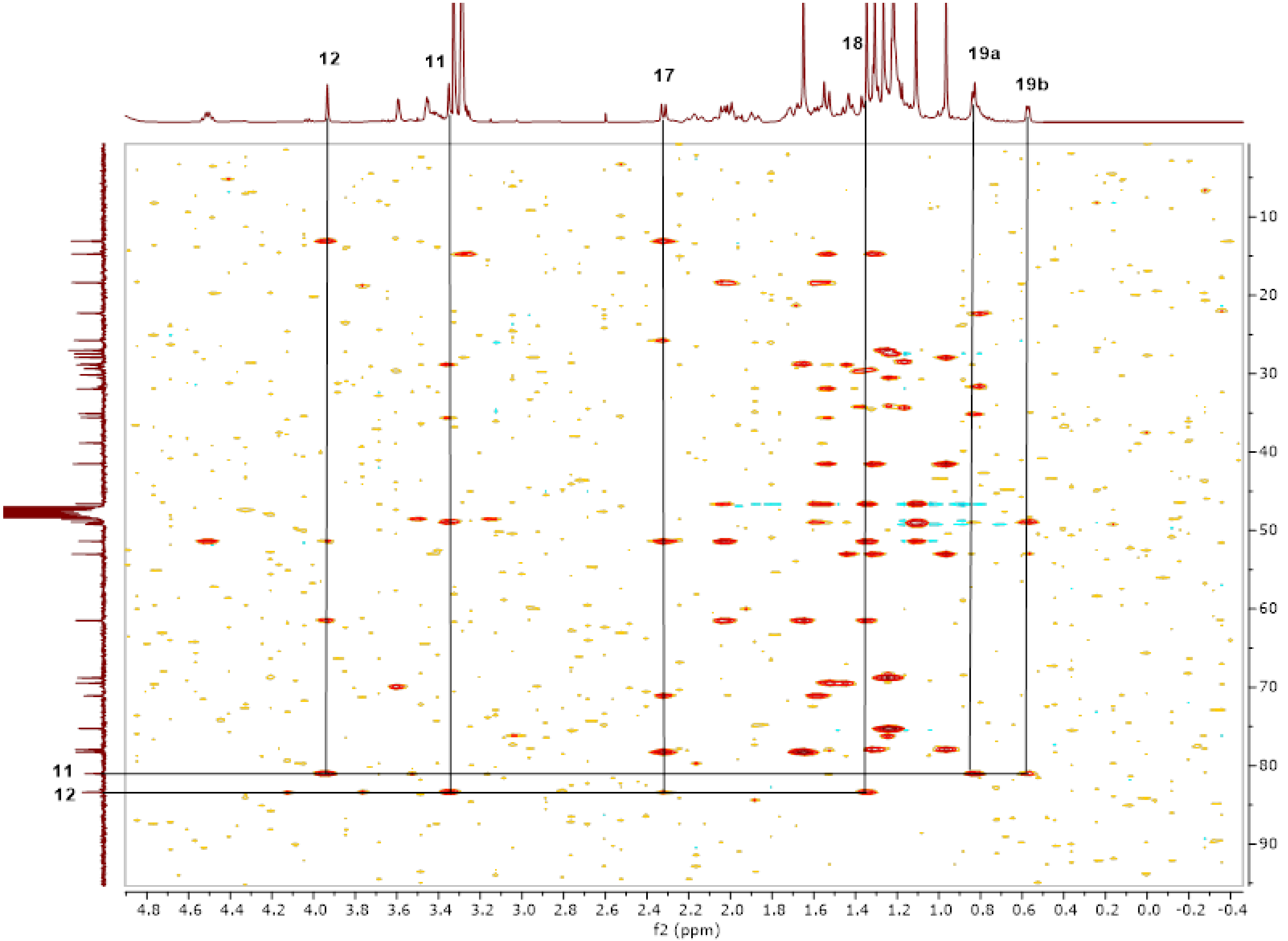

Figure S 145 HMBC spectrum of compound **16**

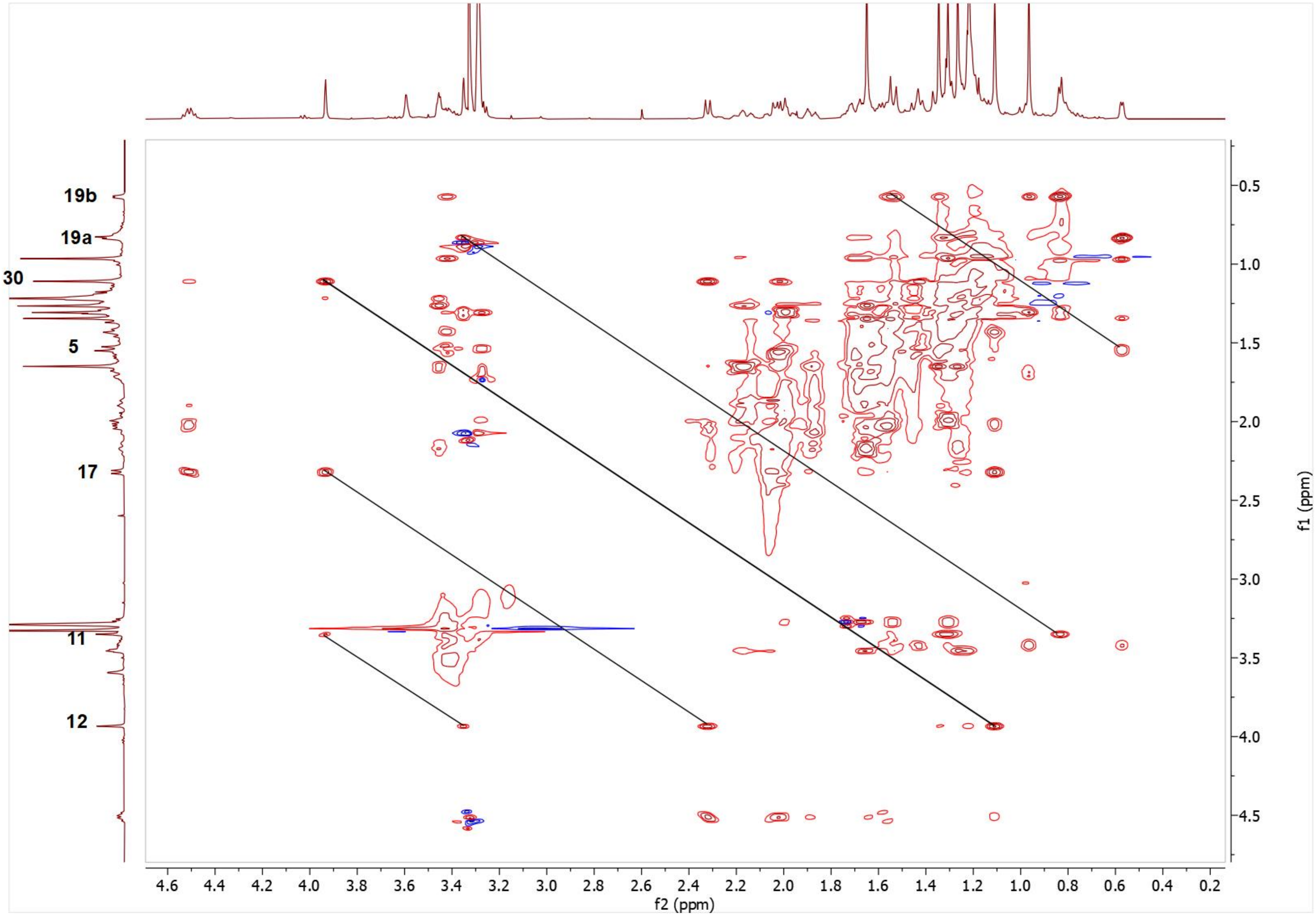

Figure S 146 NOESY spectrum of compound **16**

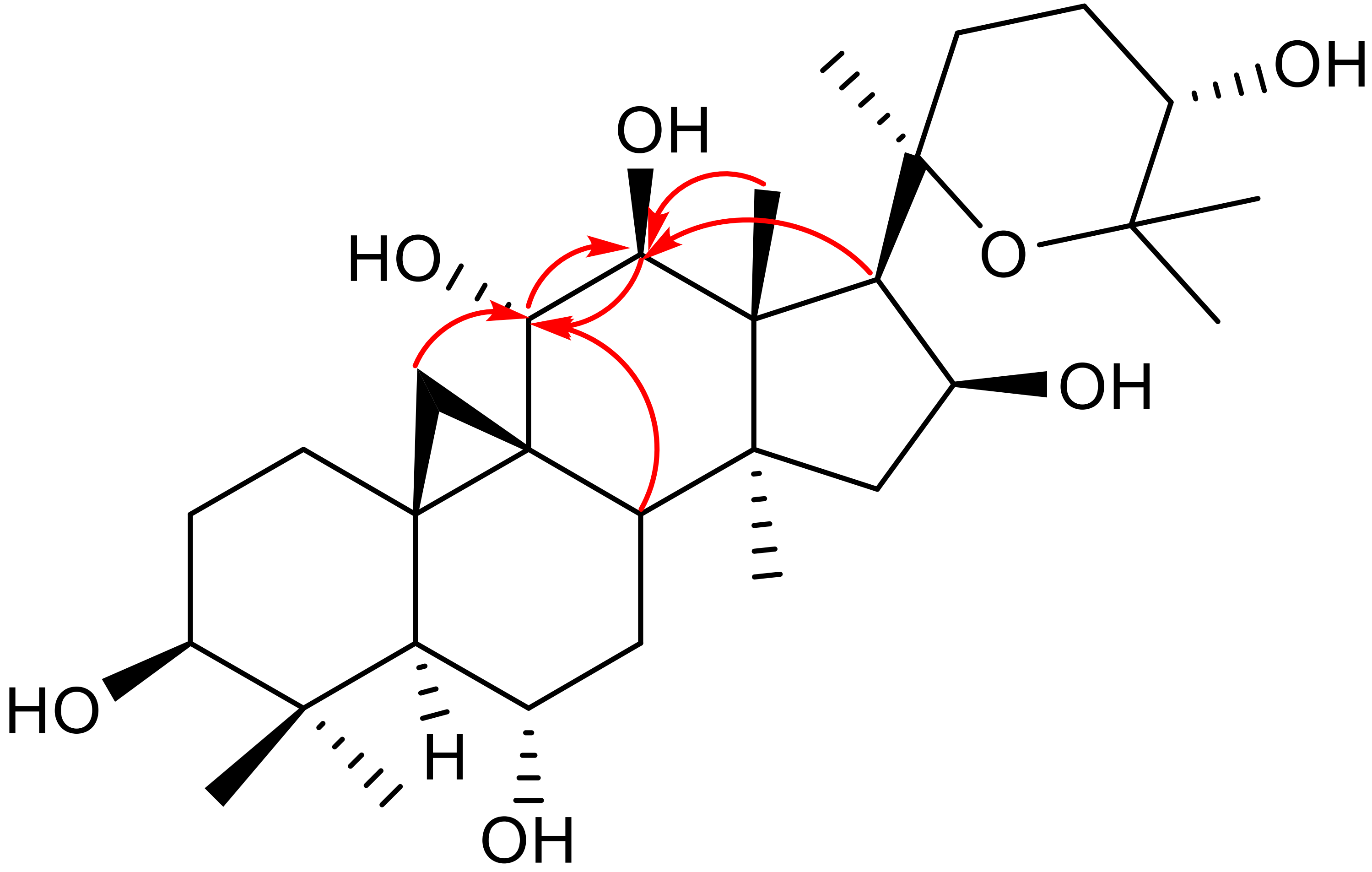

Figure S 147 Key HMBC correlations of compound **16** (arrows from H to C)

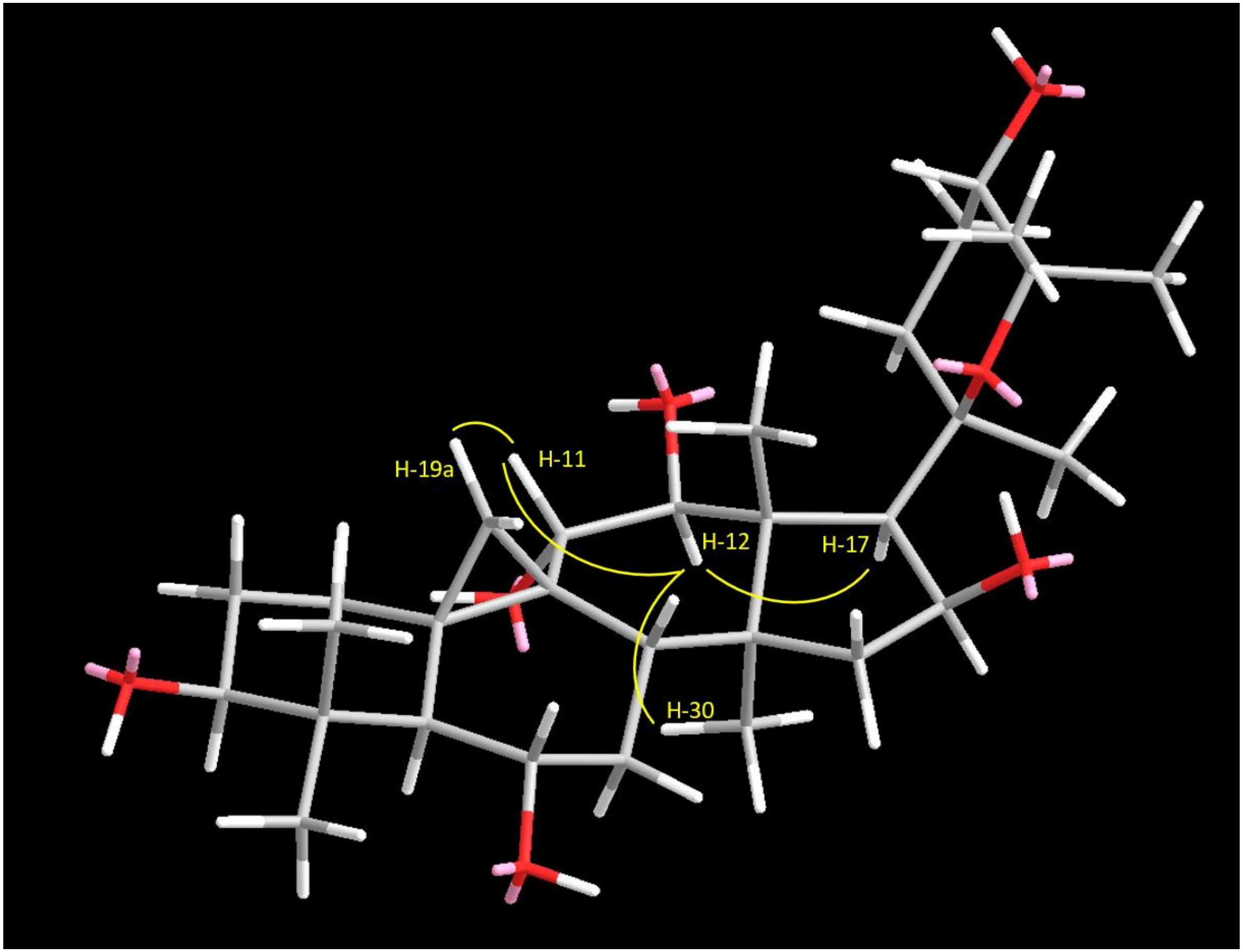

Figure S 148 Key NOE correlations of compound **16**

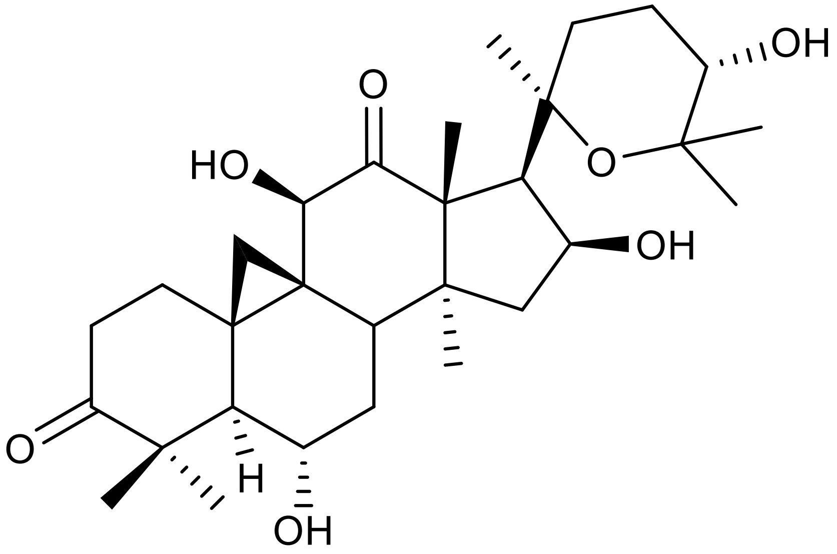

Figure S 149 Structure of compound **17**

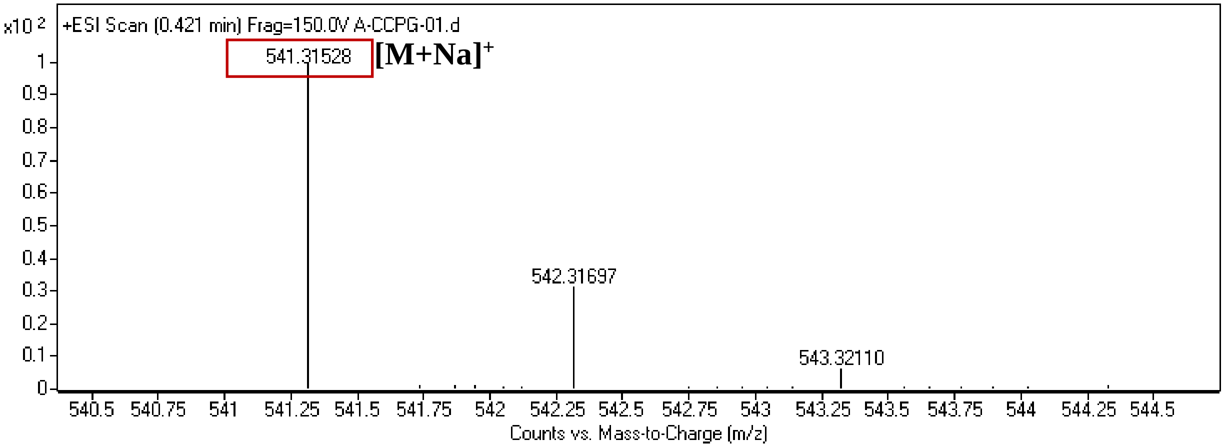

Figure S 150 HR-ESI-MS spectrum of compound **17**

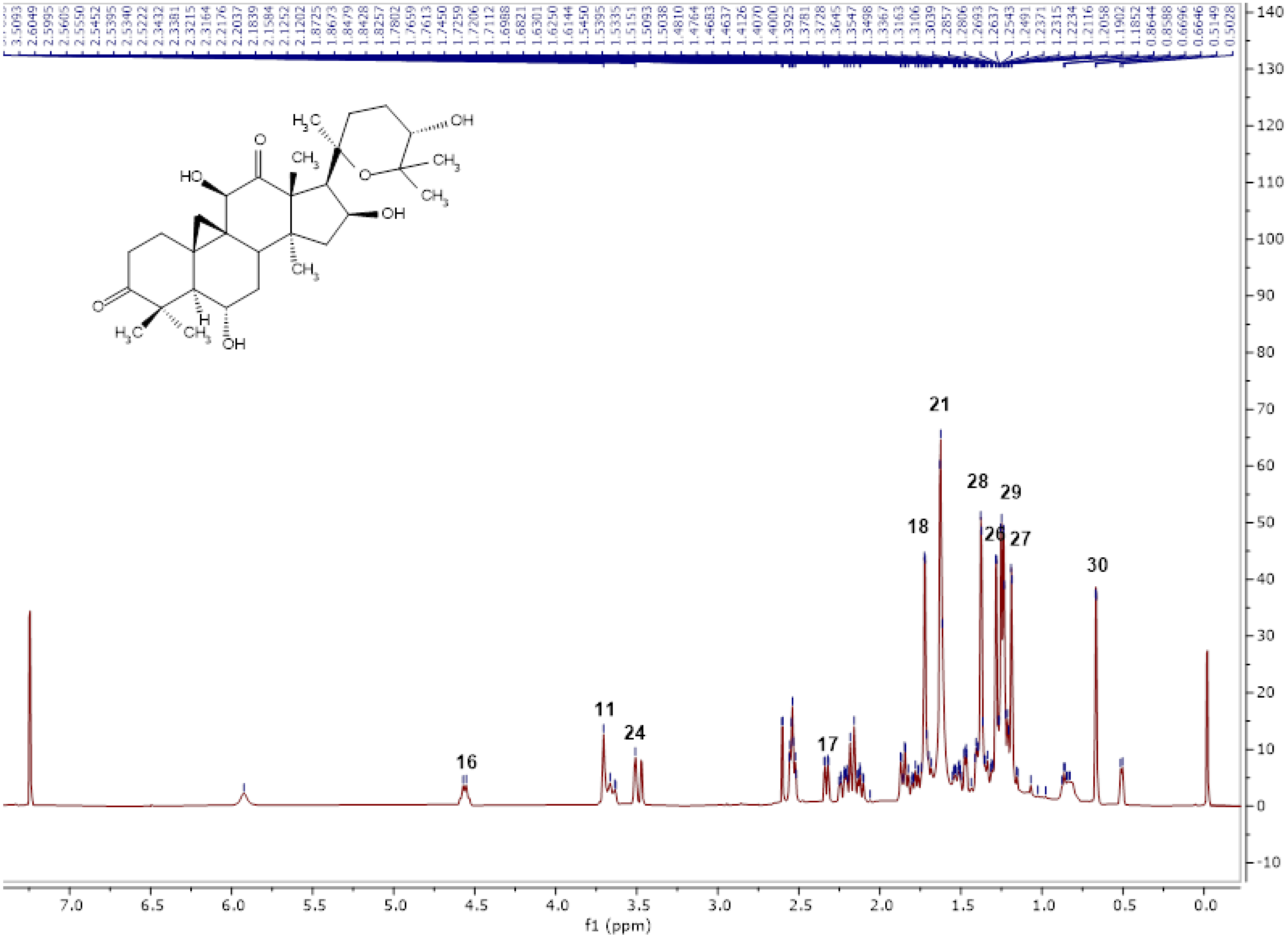

Figure S 151 ^{1}H-NMR spectrum of compound **17** (400 MHz, $CDCl_3$)

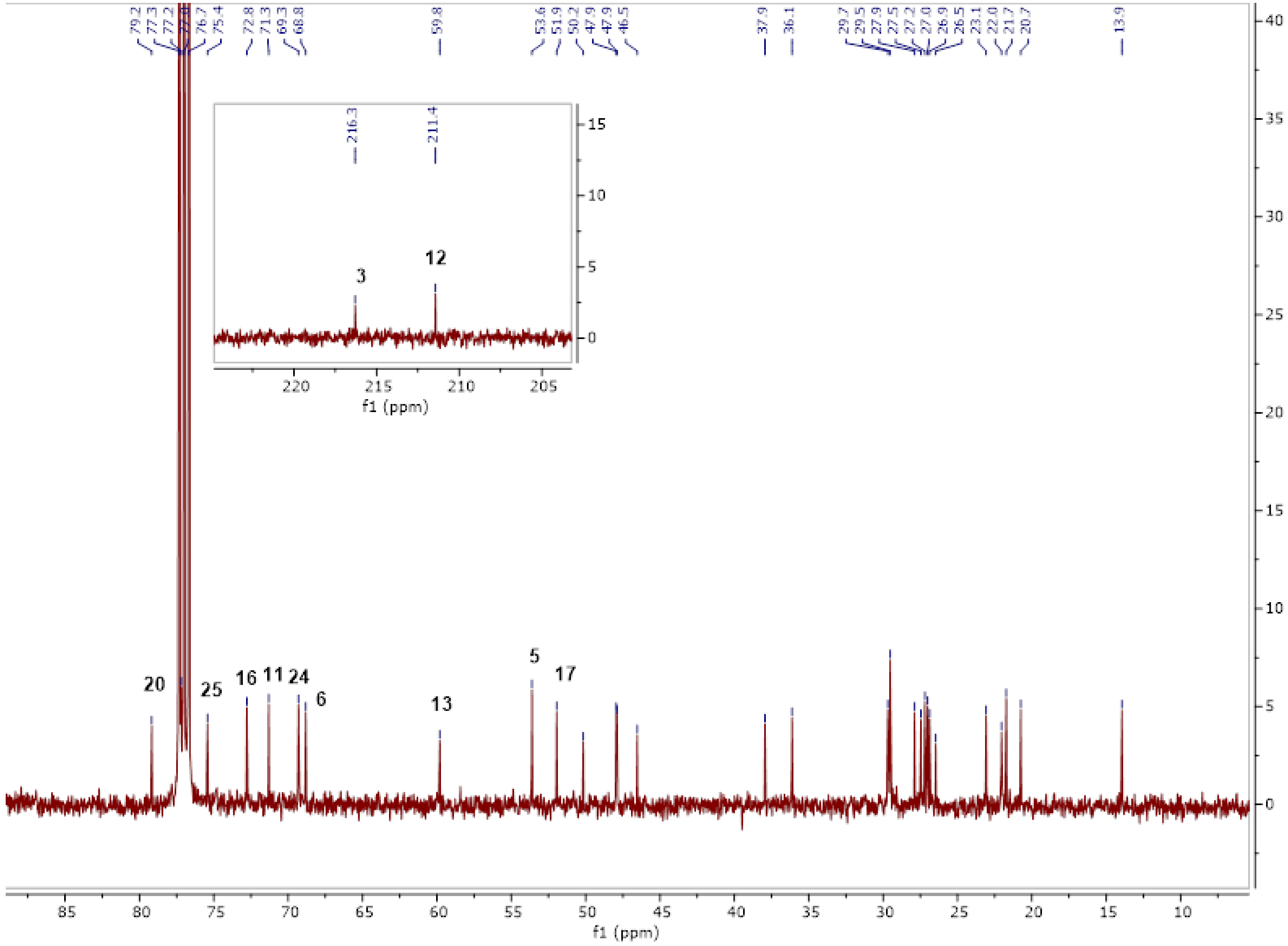

Figure S 152 ^{13}C-NMR spectrum of compound **17** (100 MHz, $CDCl_3$)

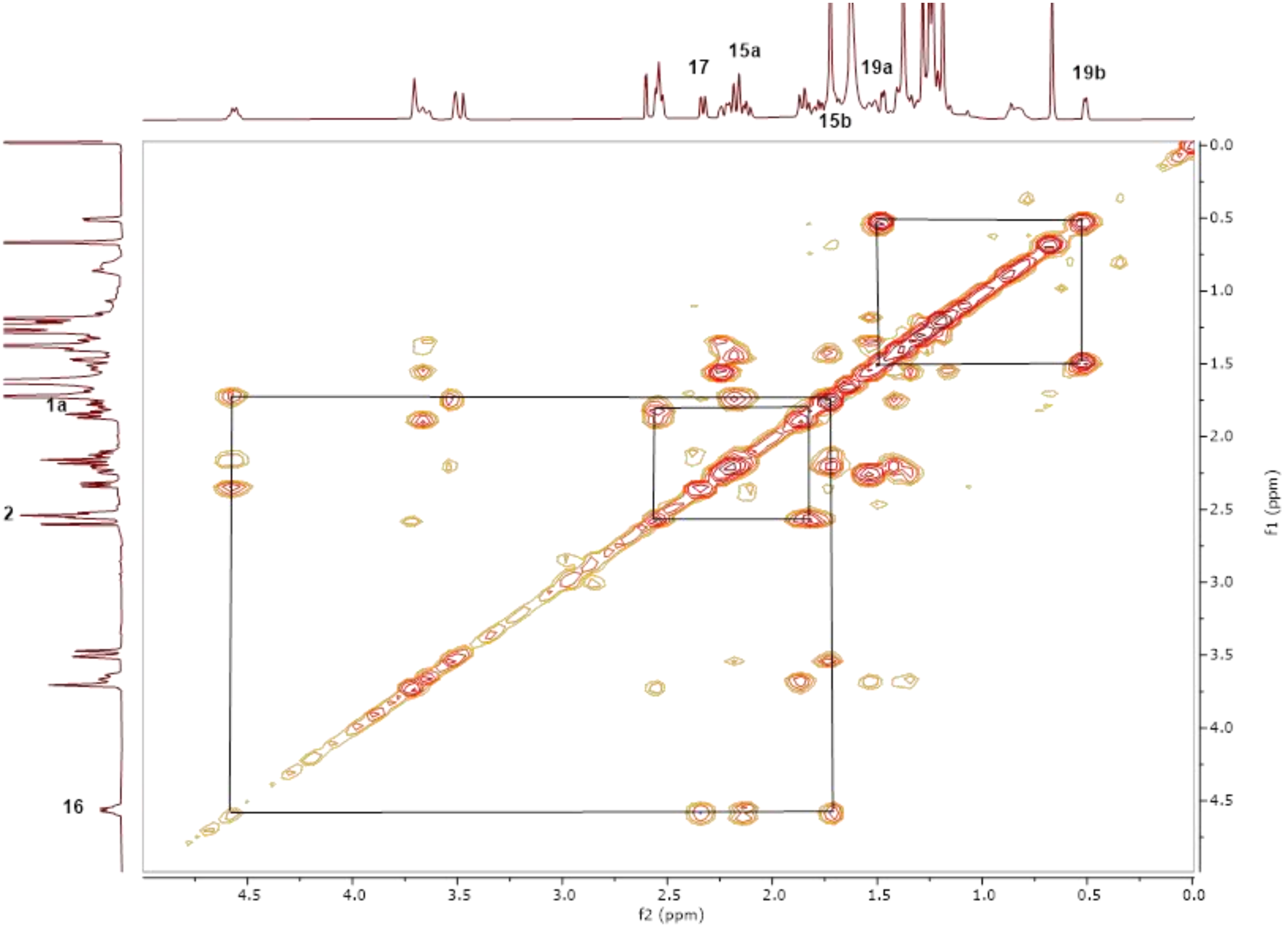

Figure S 153 COSY spectrum of compound **17**

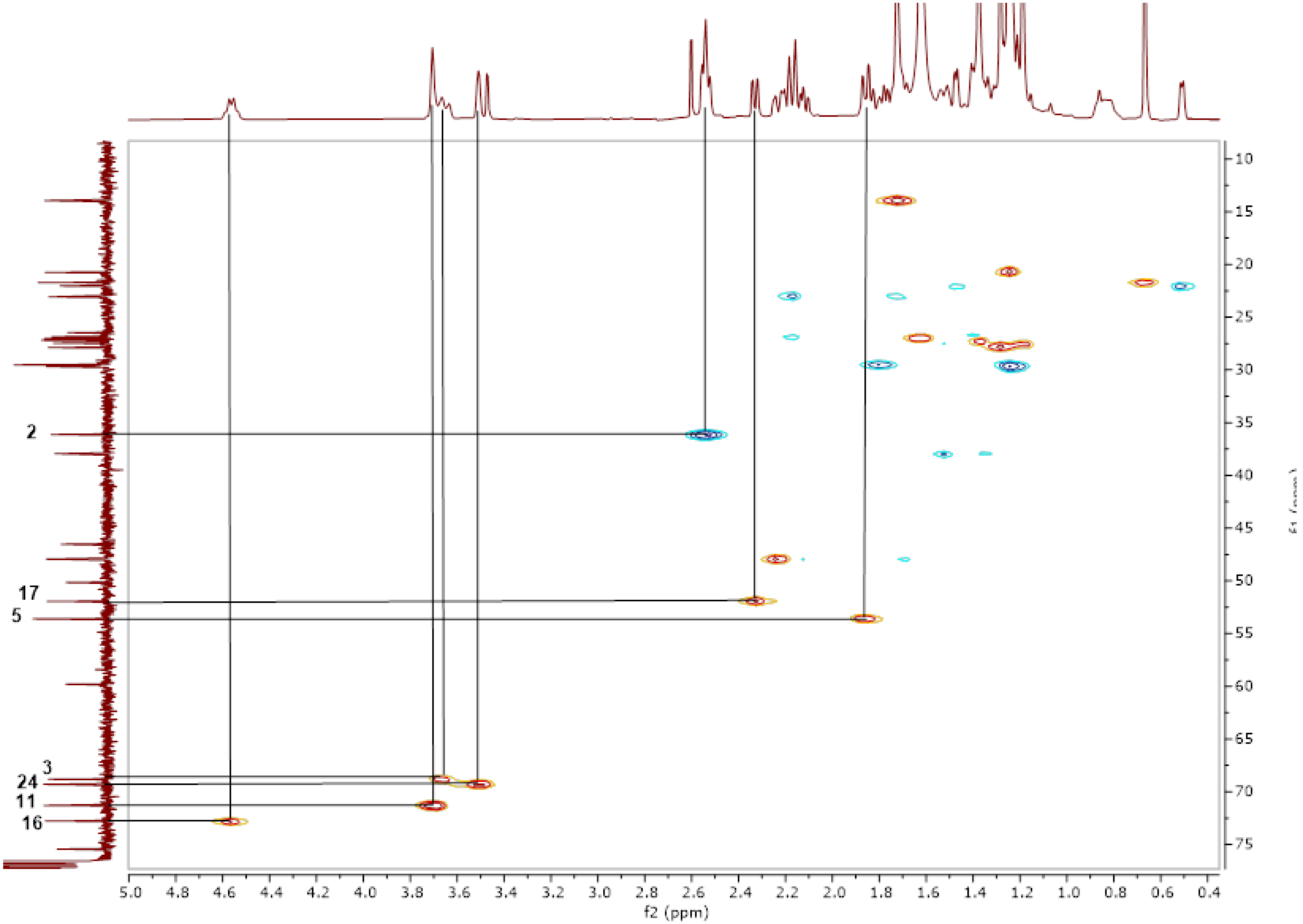

Figure S 154 HSQC spectrum of compound **17**

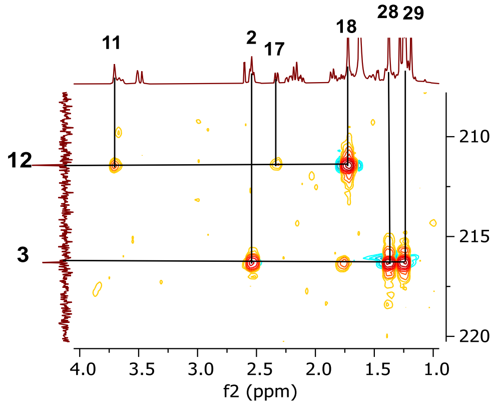

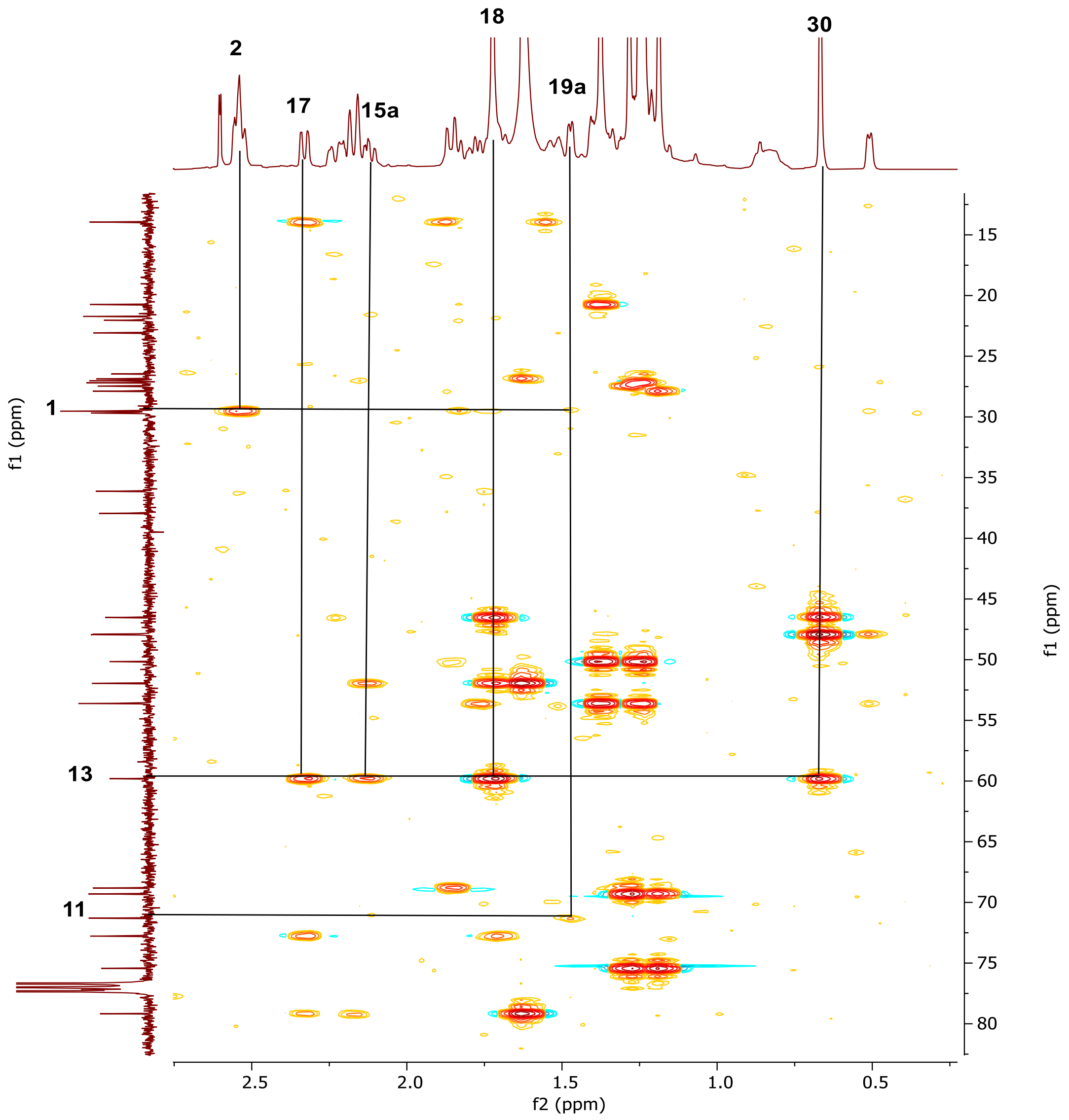

Figure S 155 HMBC spectrum of compound **17**

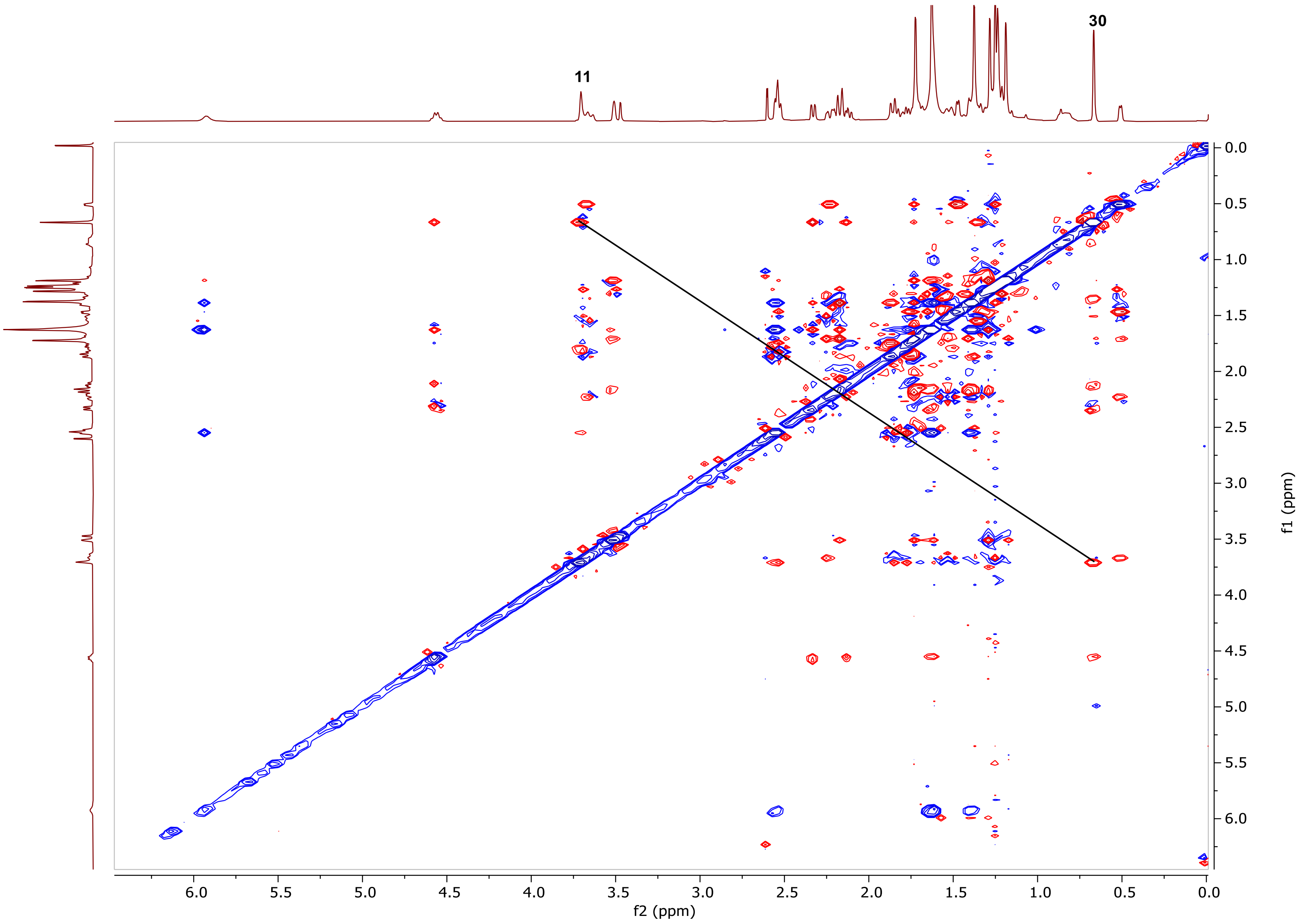

Figure S 156 NOESY spectrum of compound **17**

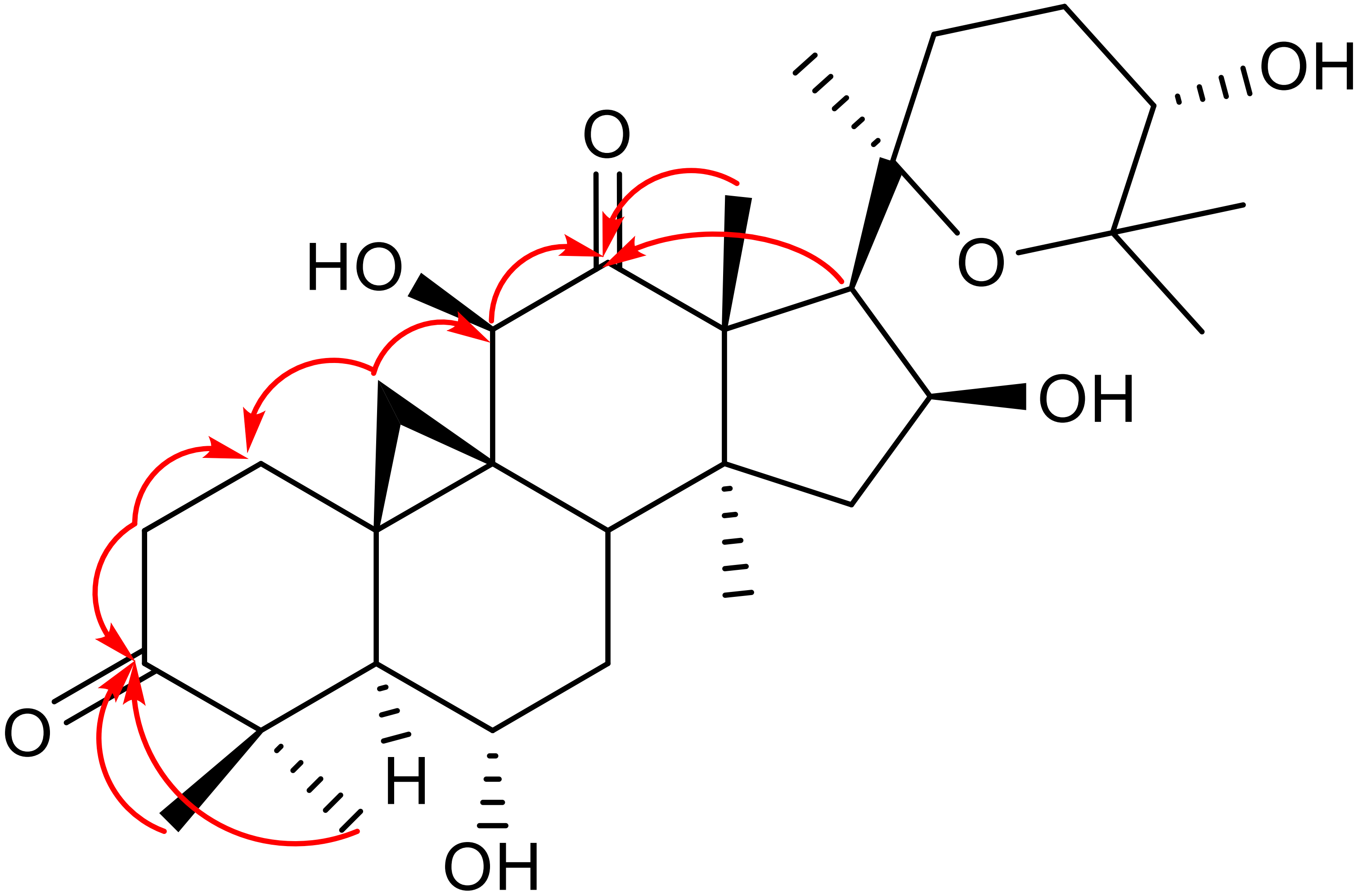

Figure S 157 Key HMBC correlations of compound **17** (arrows from H to C)

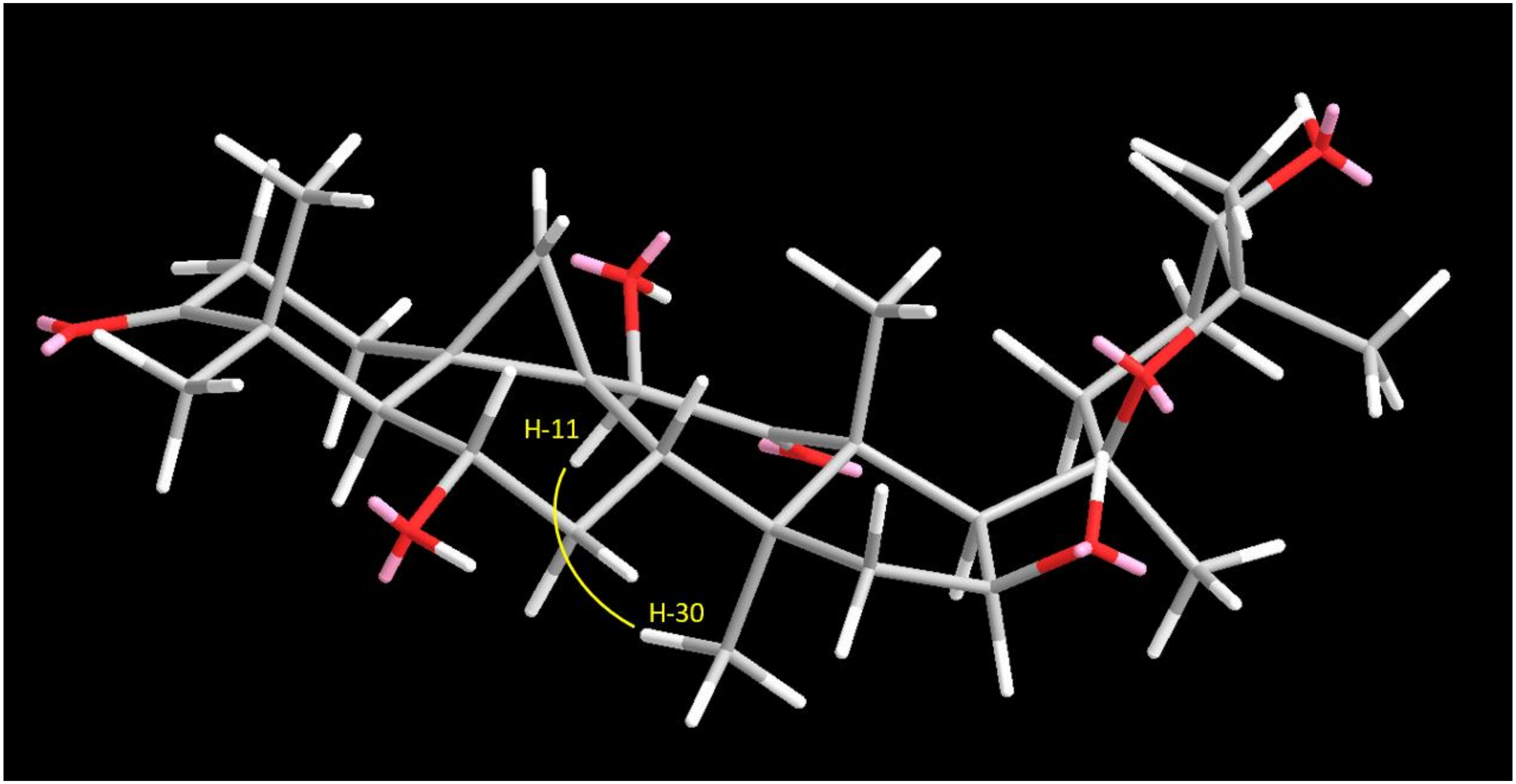

Figure S 158 Key NOE correlation of compound **17**

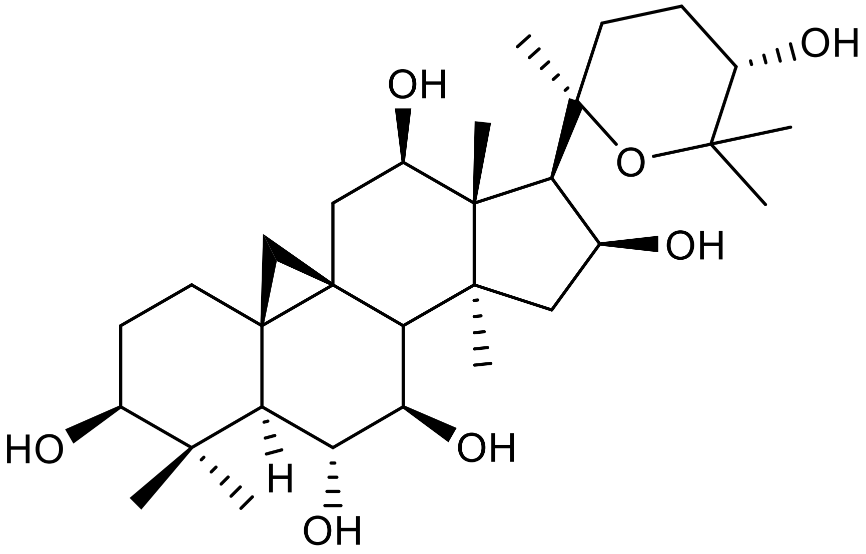

Figure S 159 Structure of compound **18**

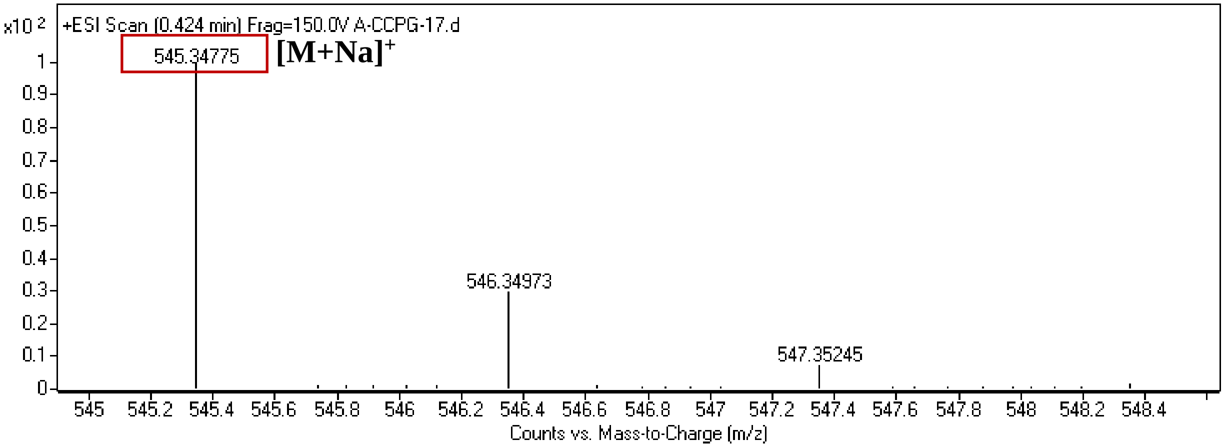

Figure S 160 HR-ESI-MS spectrum of compound **18**

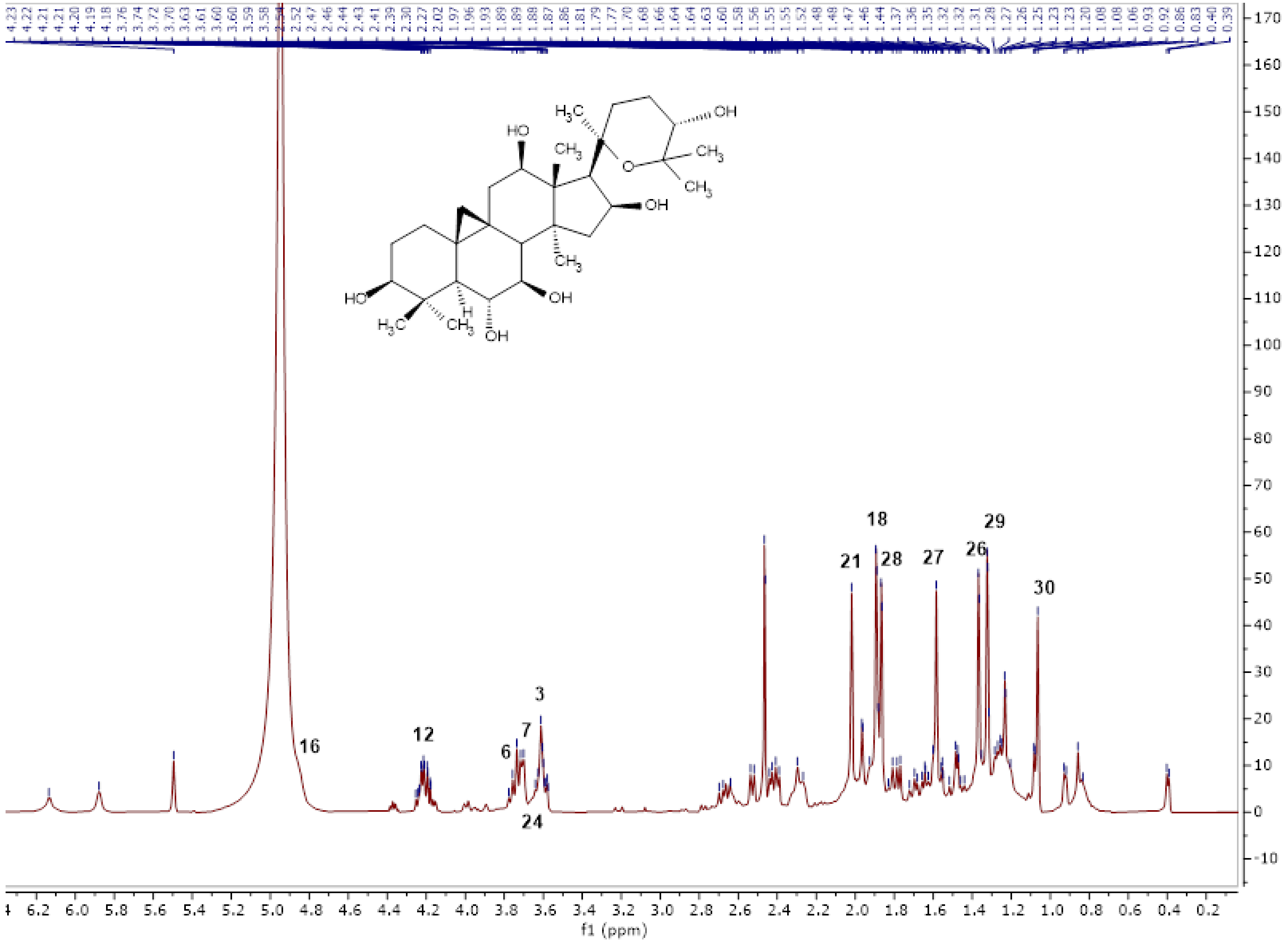

Figure S 161 ^{1}H-NMR spectrum of compound **18** (400 MHz, C_5D_5N)

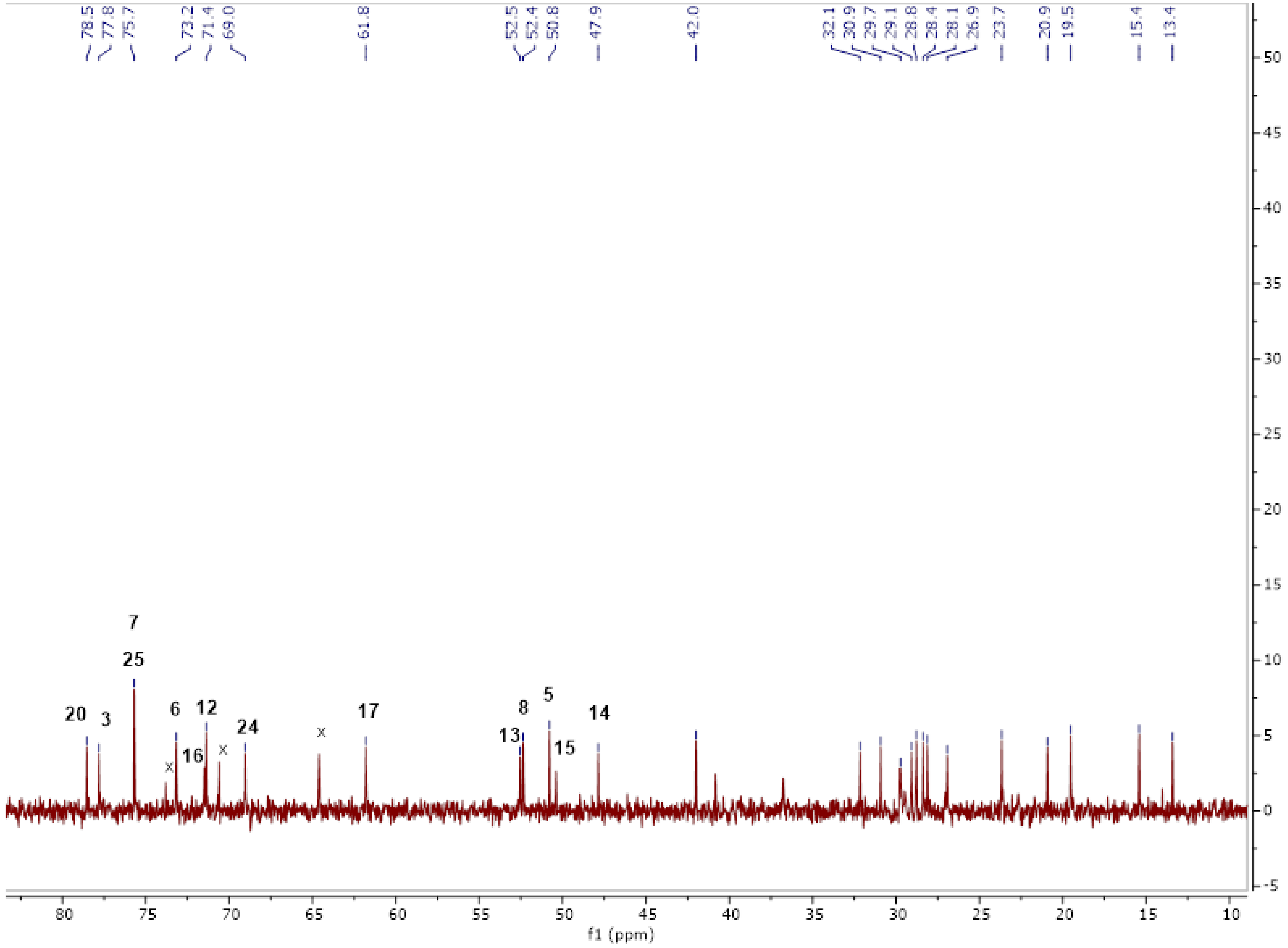

Figure S 162 ^{13}C-NMR spectrum of compound **18** (100 MHz, C_5D_5N)

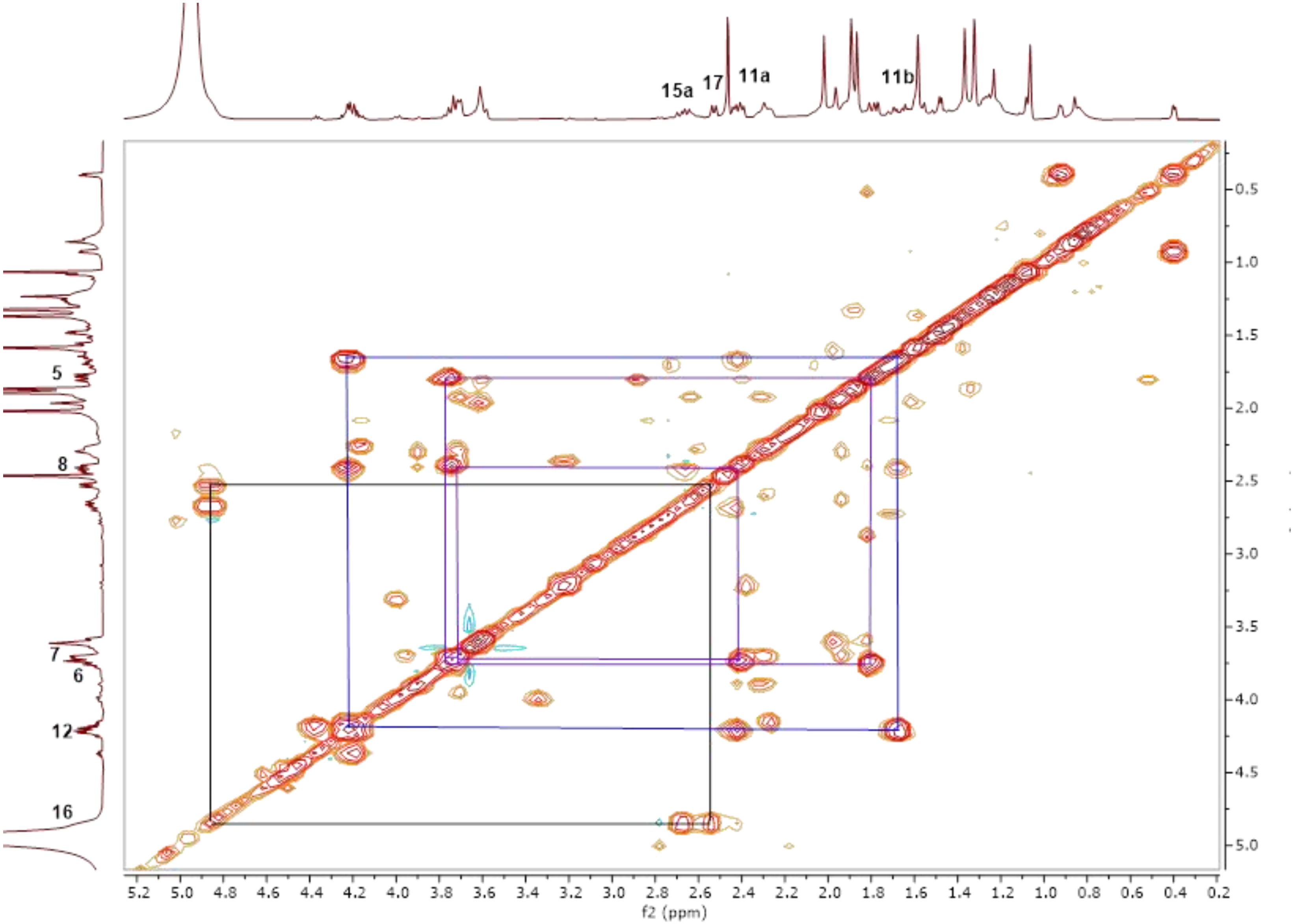

Figure S 163 COSY spectrum of compound **18**

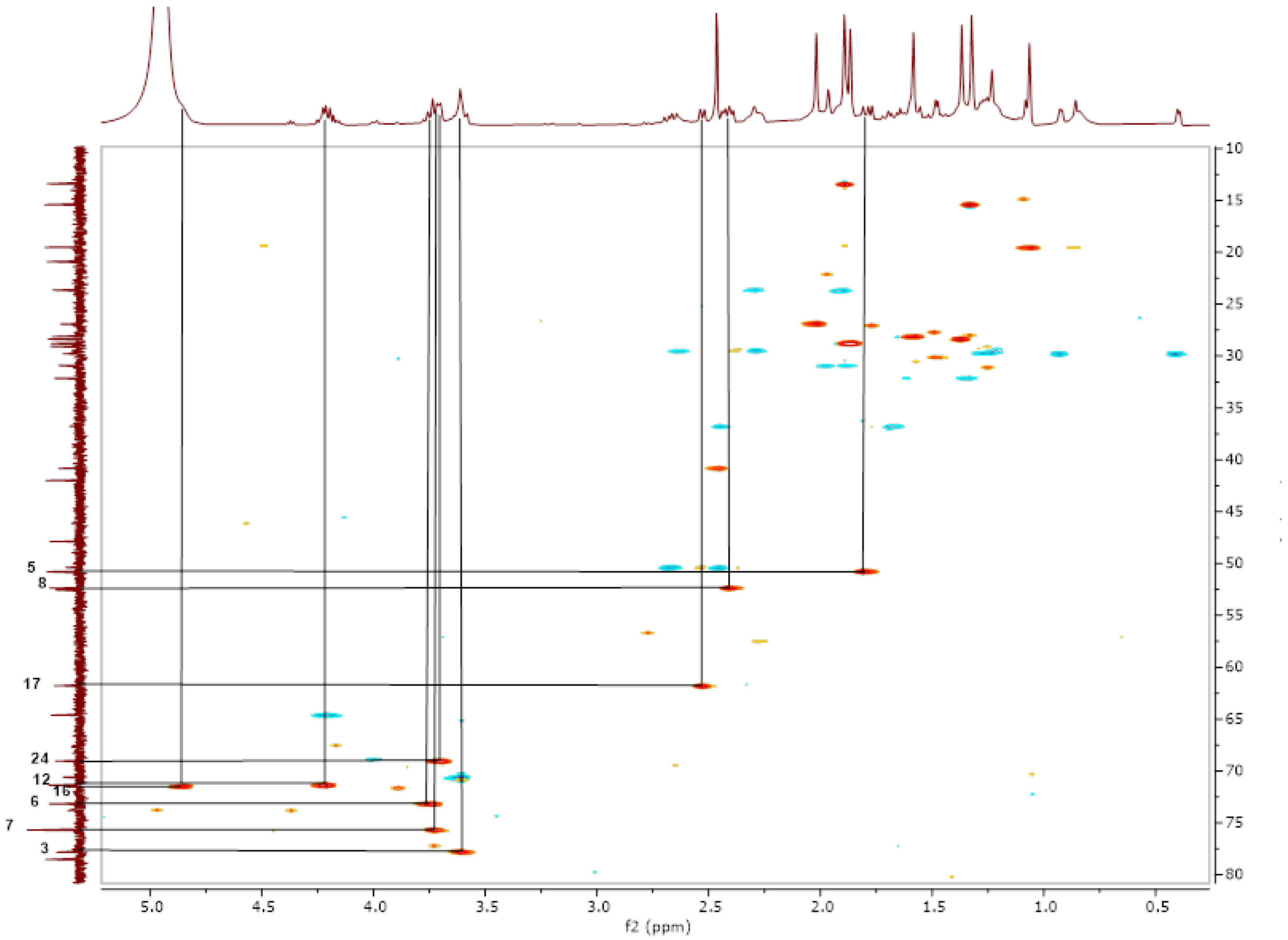

Figure S 164 HSQC spectrum of compound **18**

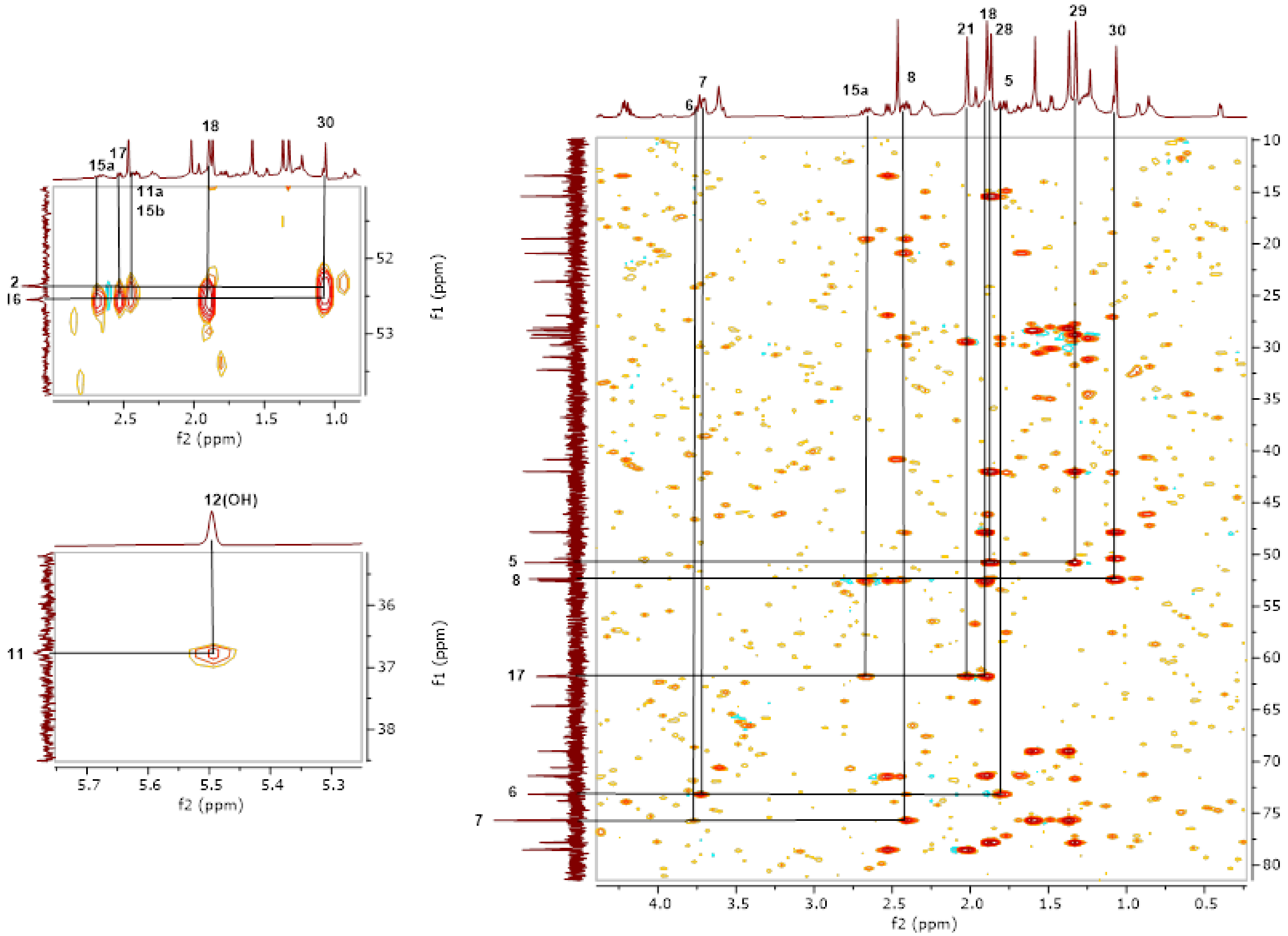

Figure S 165 HMBC spectrum of compound **18**

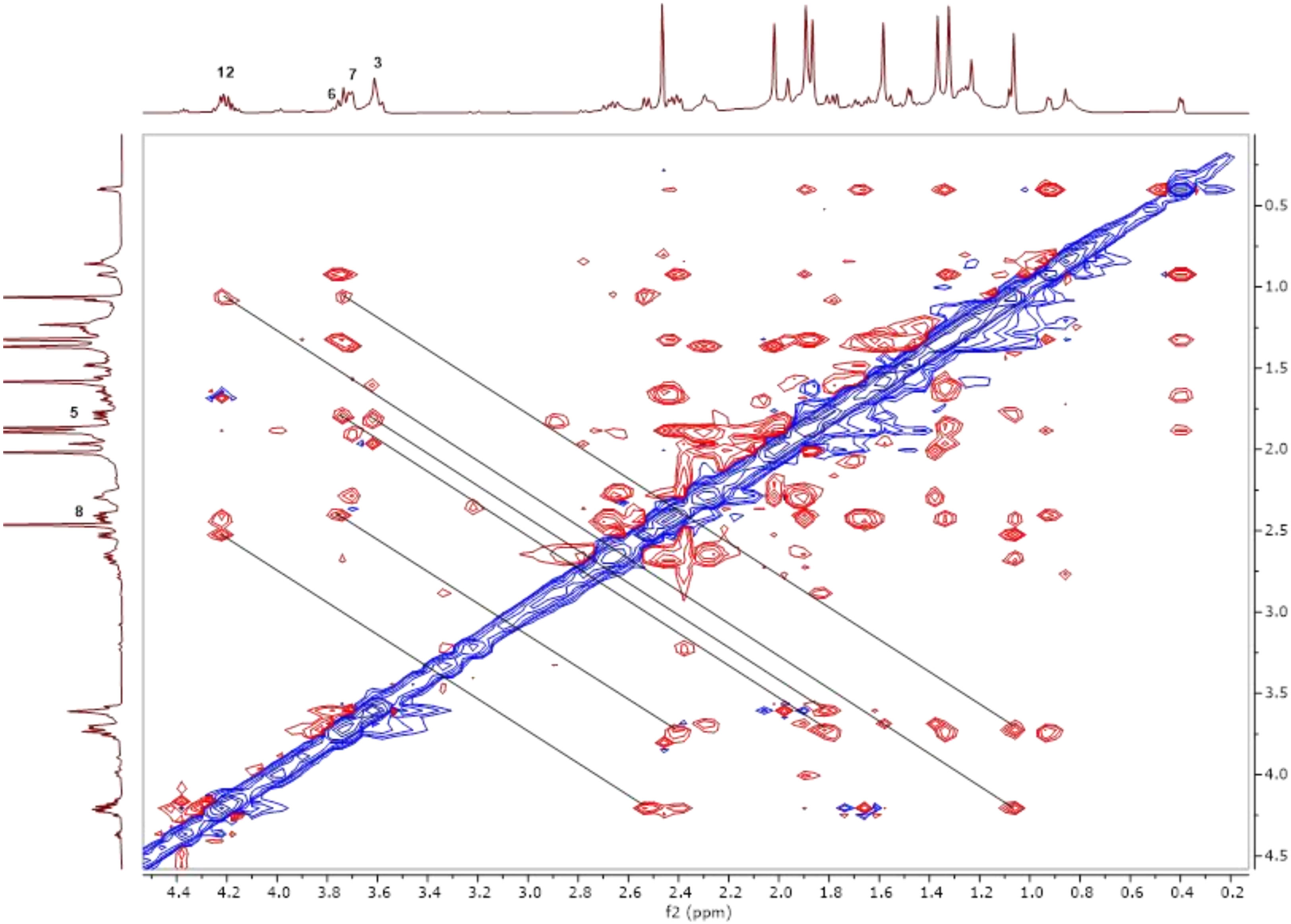

Figure S 166 NOESY spectrum of compound **18**

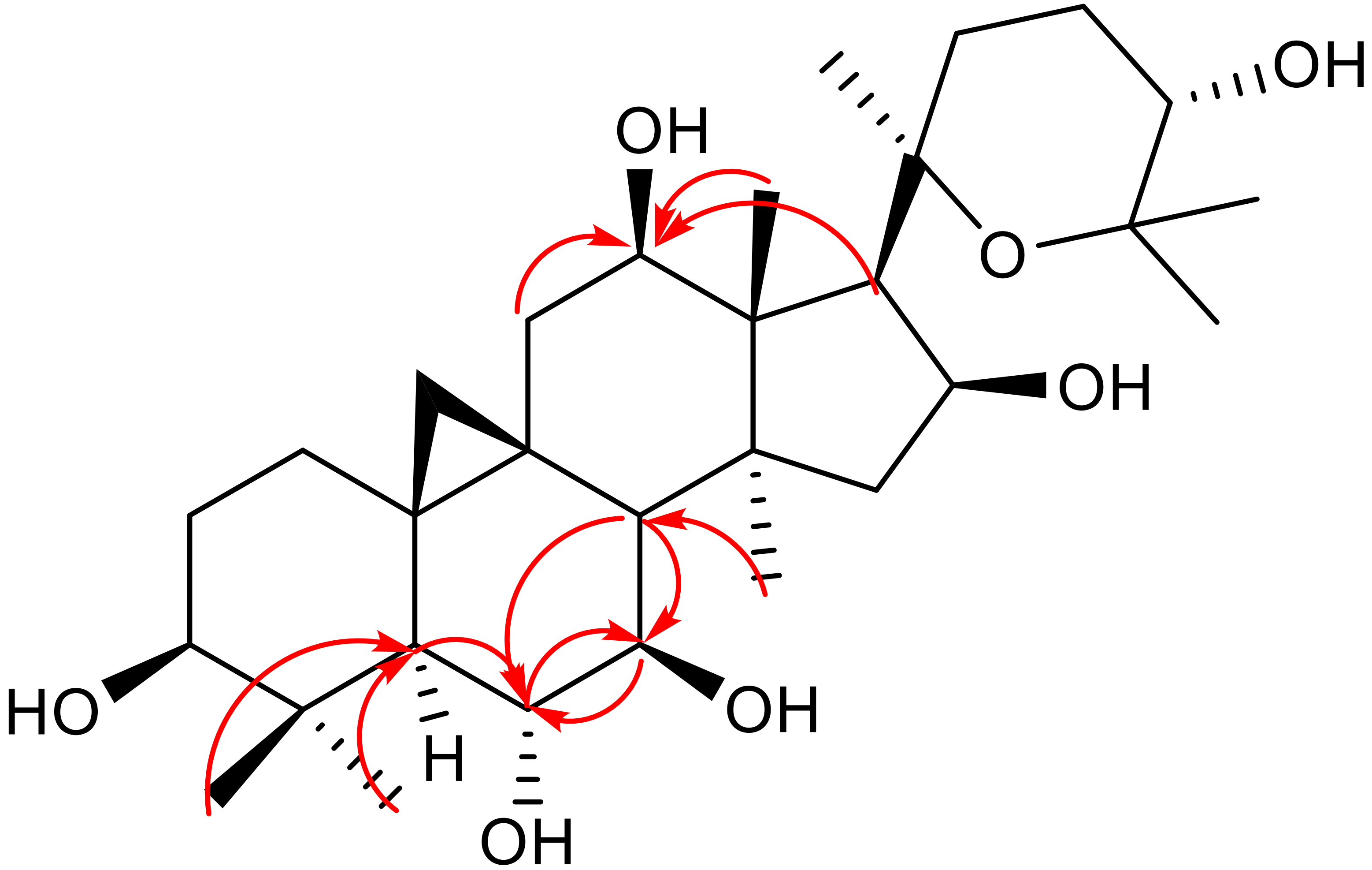

Figure S 167 Key HMBC correlations of compound **18** (arrows from H to C)

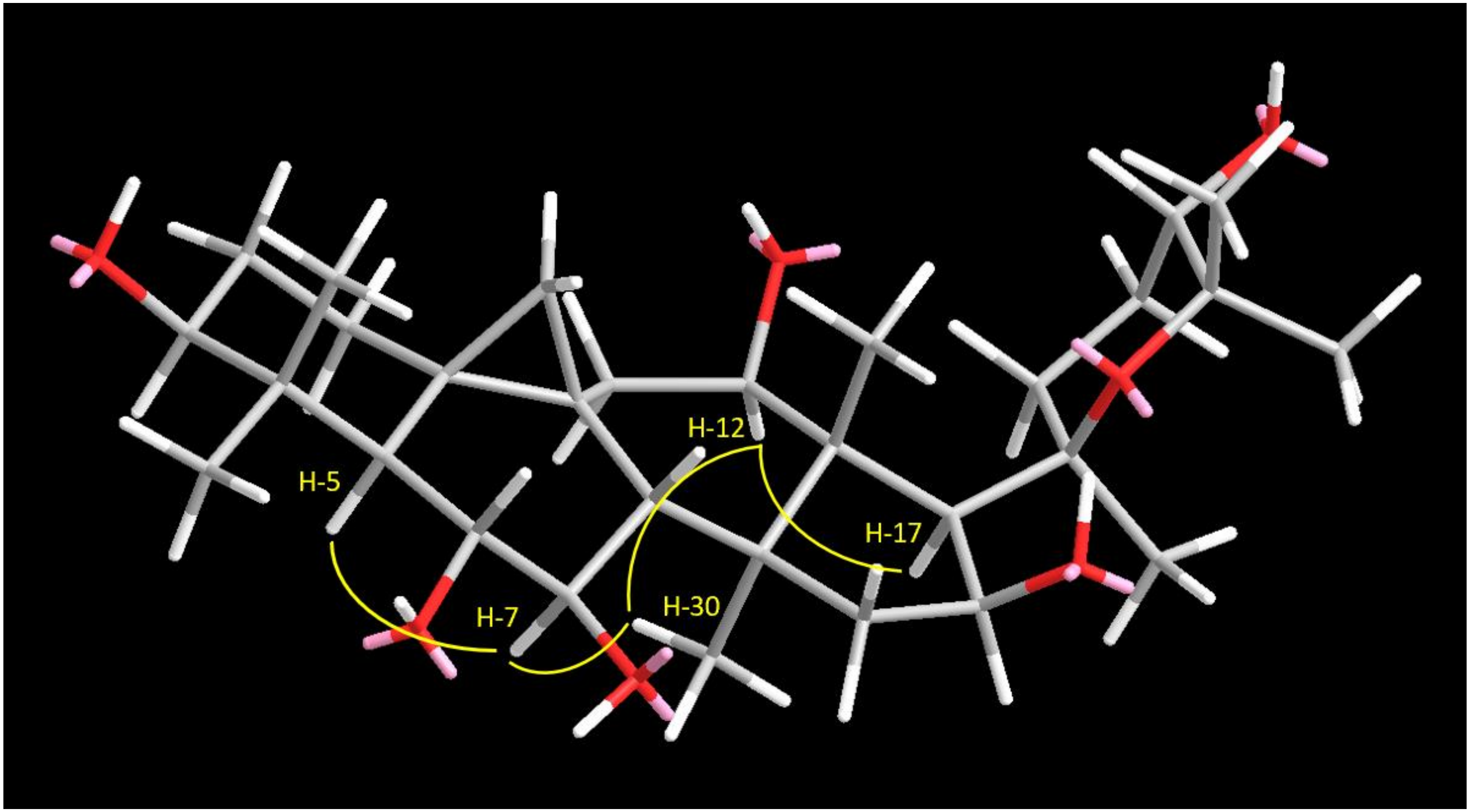

Figure S 168 Key NOE correlations of compound **18**

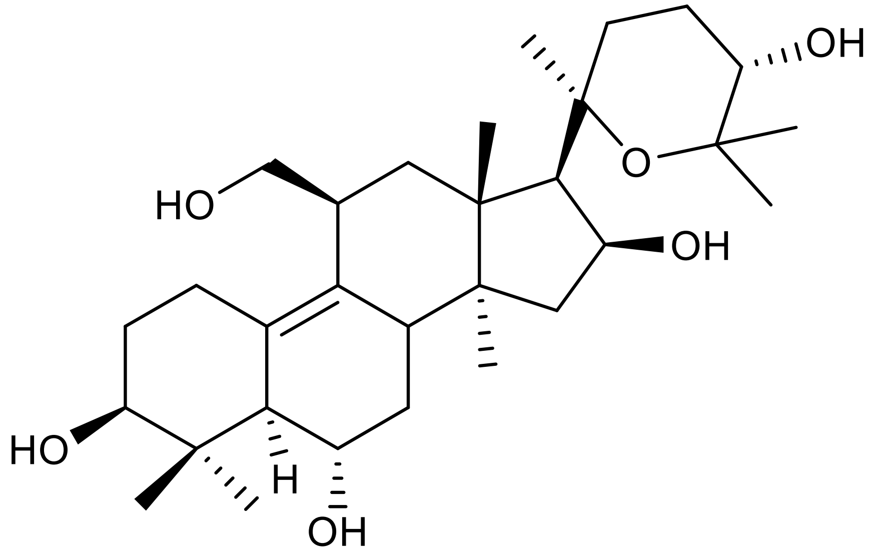

Figure S 169 Structure of compound **19**

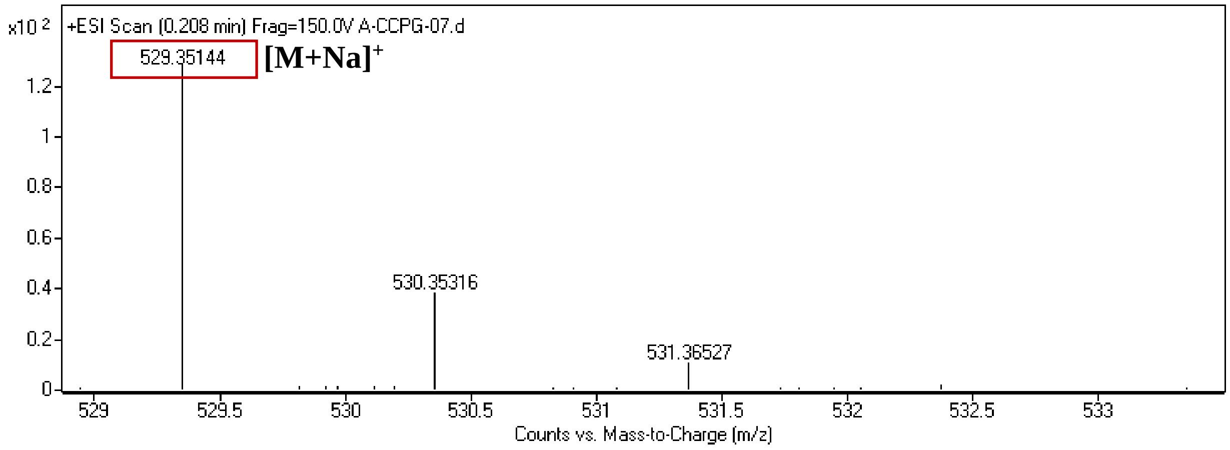

Figure S 170 HR-ESI-MS spectrum of compound **19**

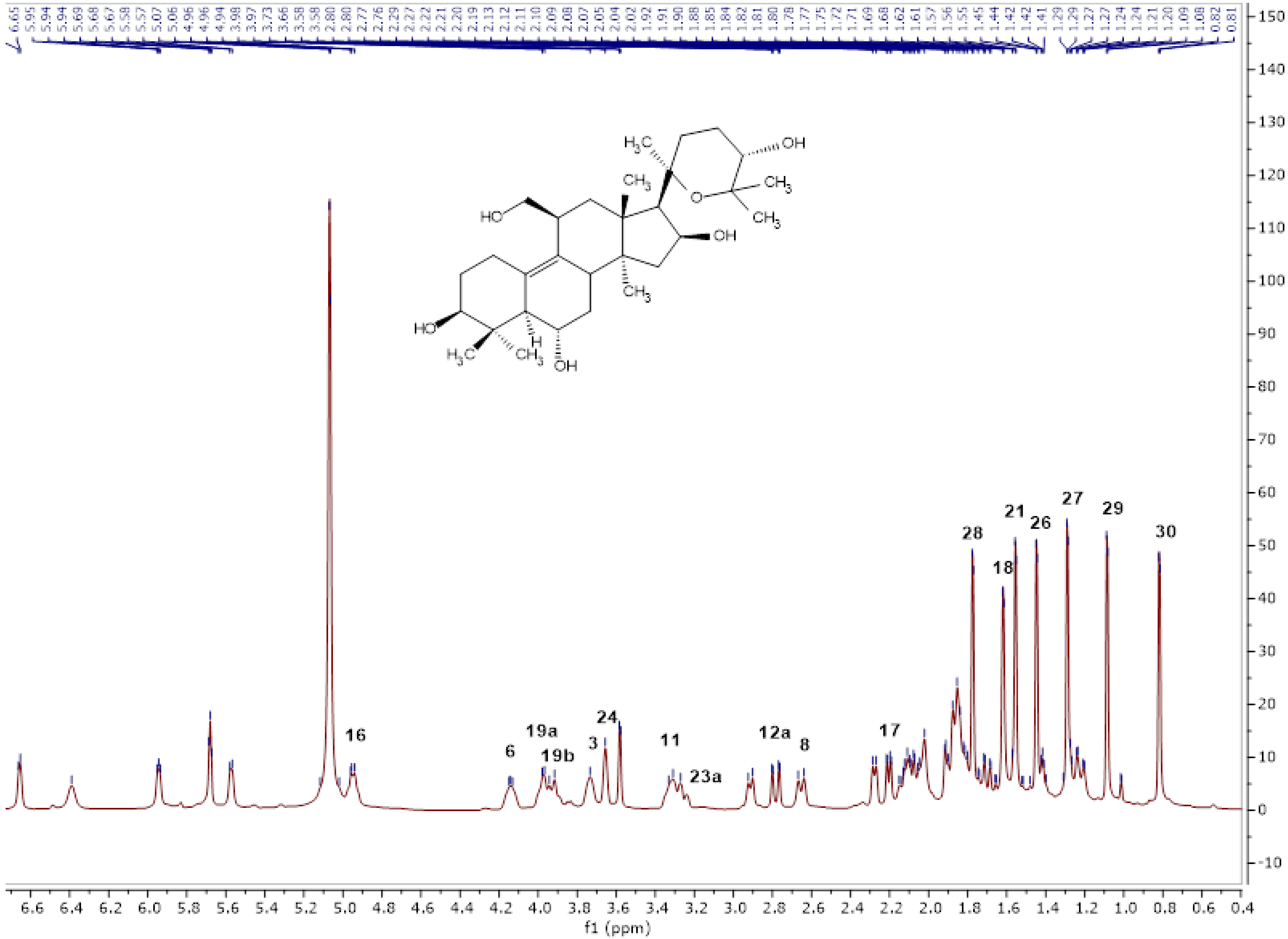

Figure S 171 ^{1}H-NMR spectrum of compound **19** (400 MHz, C_5D_5N)

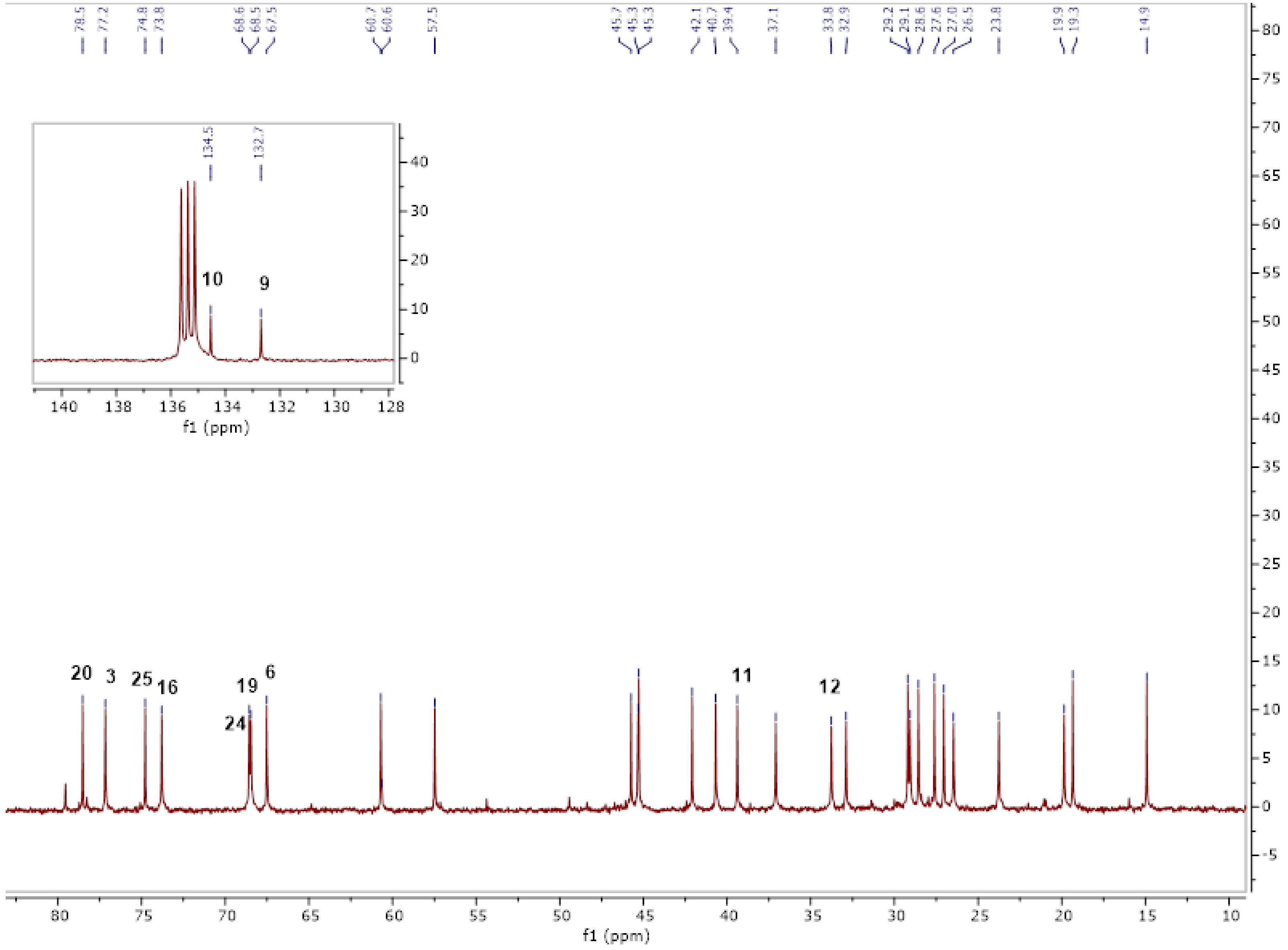

Figure S 172 ^{13}C-NMR spectrum of compound **19** (100 MHz, C_5D_5N)

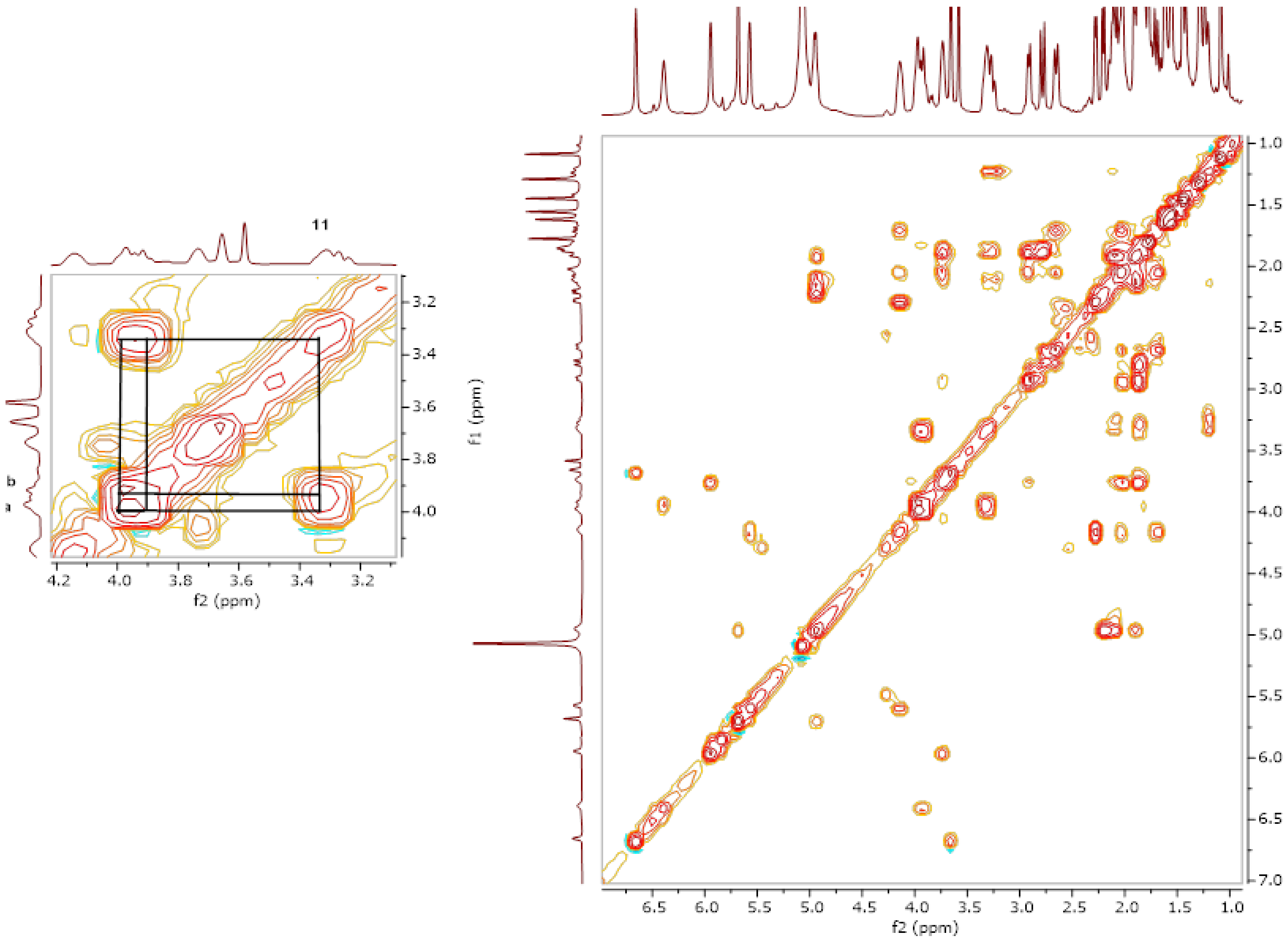

Figure S 173 COSY spectrum of compound **19**

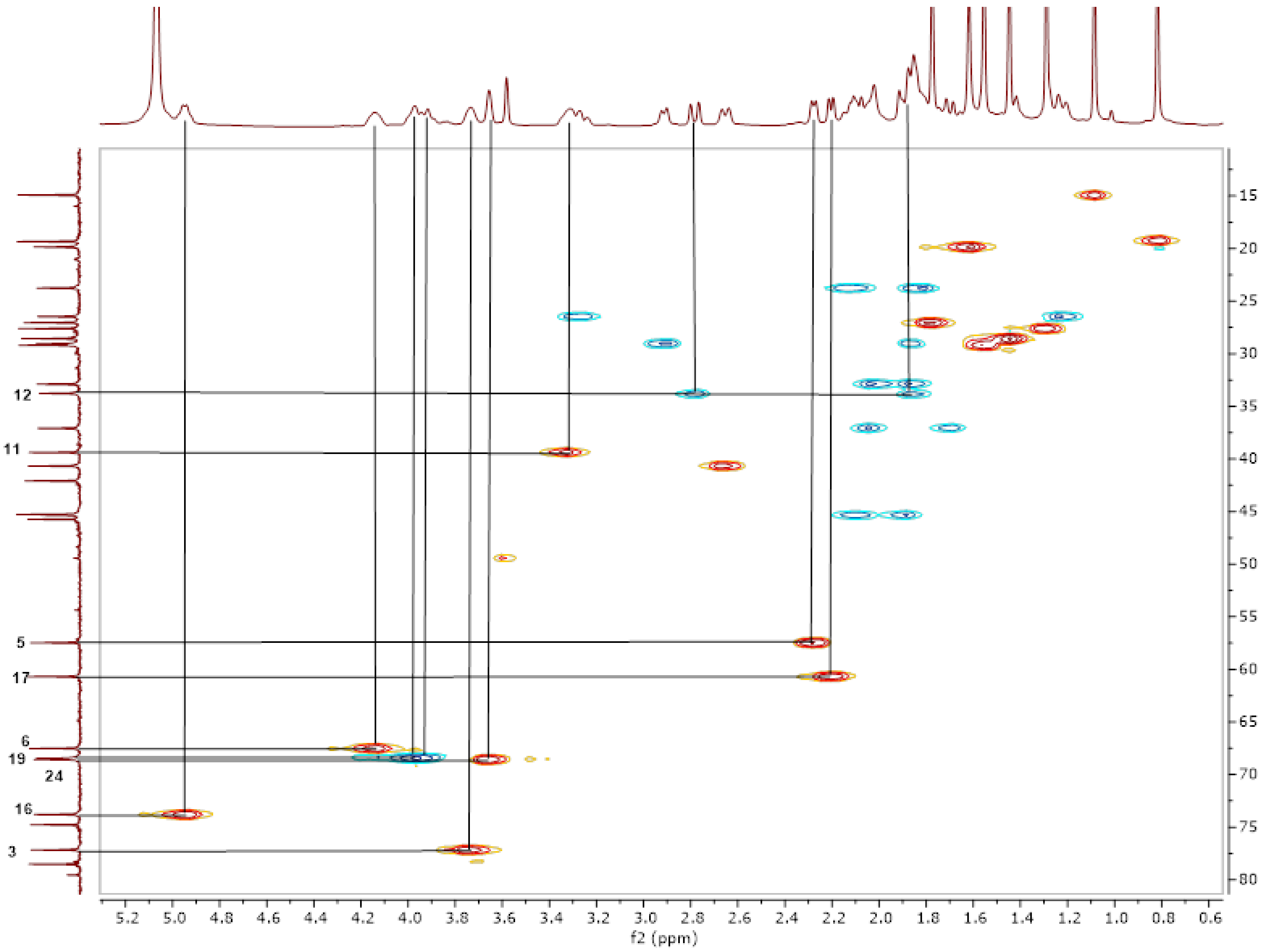

Figure S 174 HSQC spectrum of compound **19**

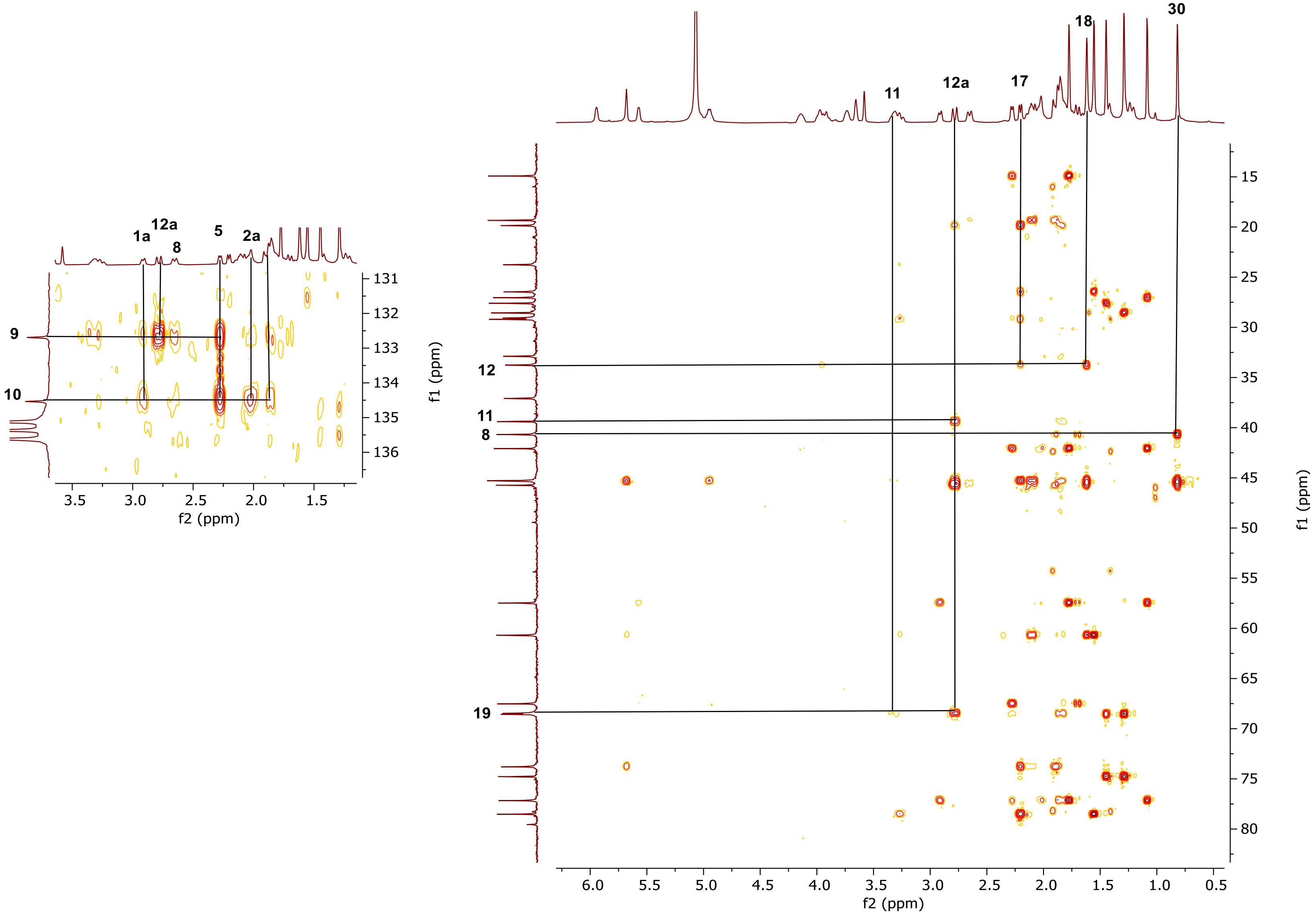

Figure S 175 HMBC spectrum of compound **19**

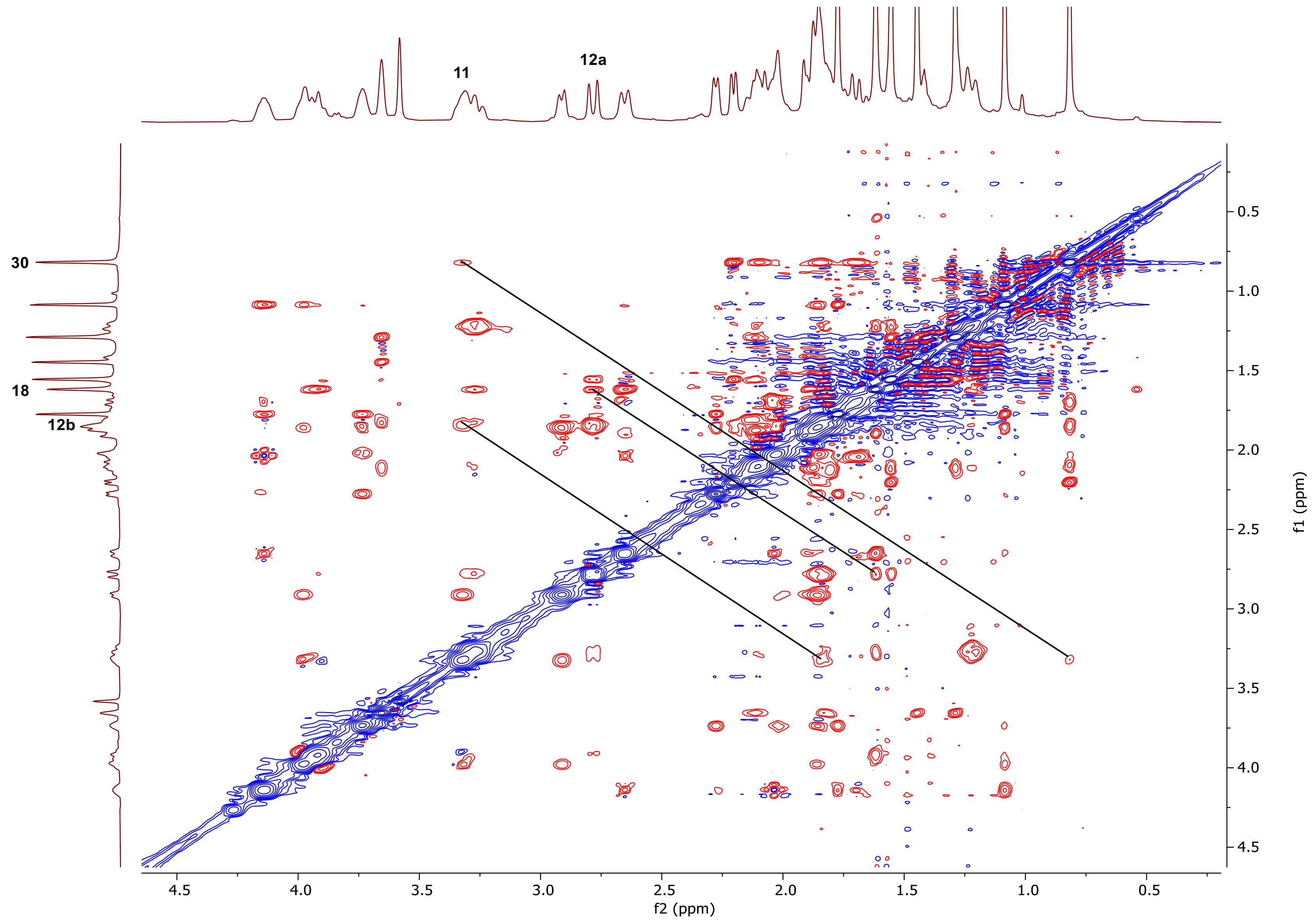

Figure S 176 NOESY spectrum of compound **19**

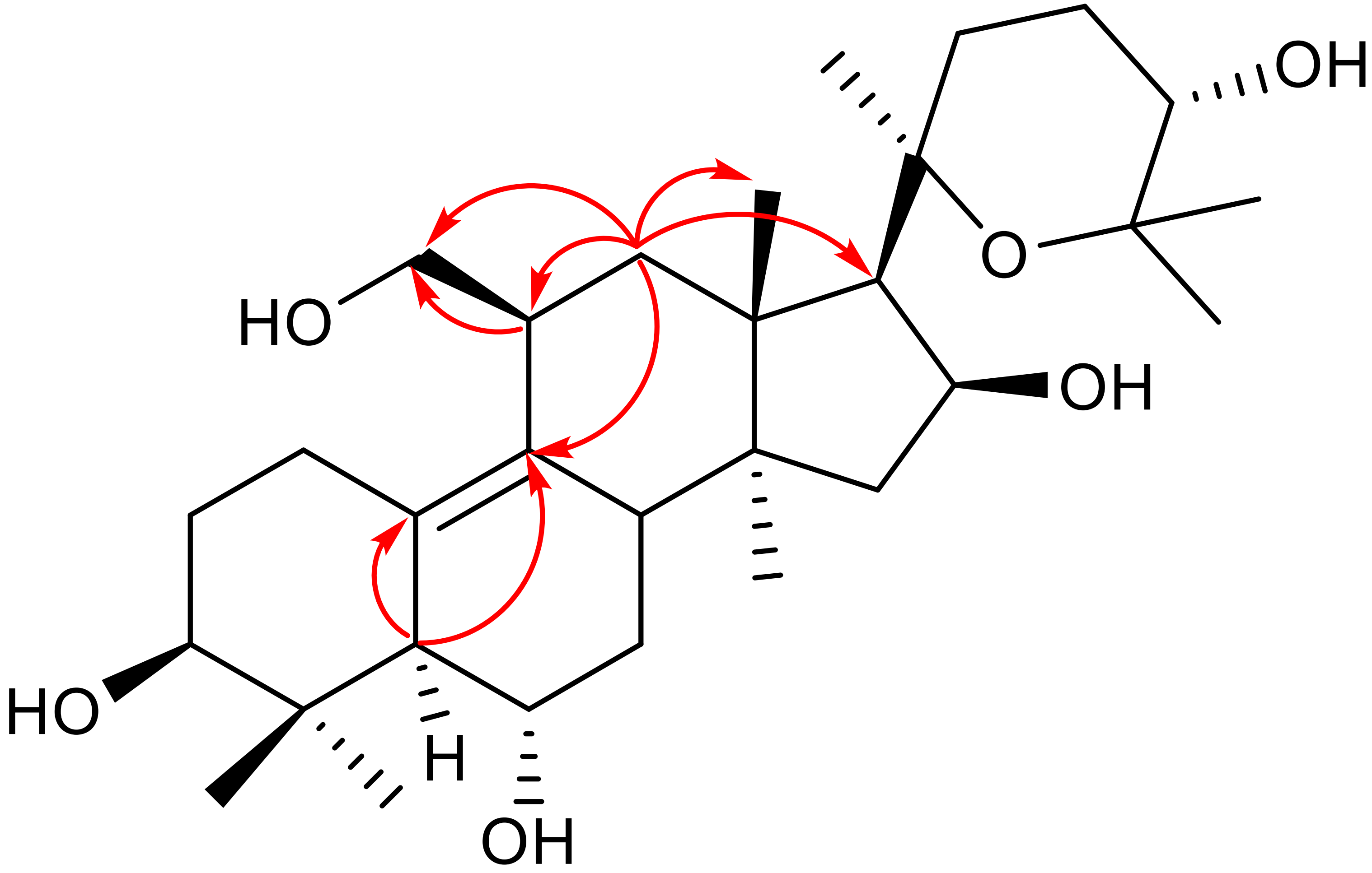

Figure S 177 Key HMBC correlations of compound **19** (arrows from H to C)

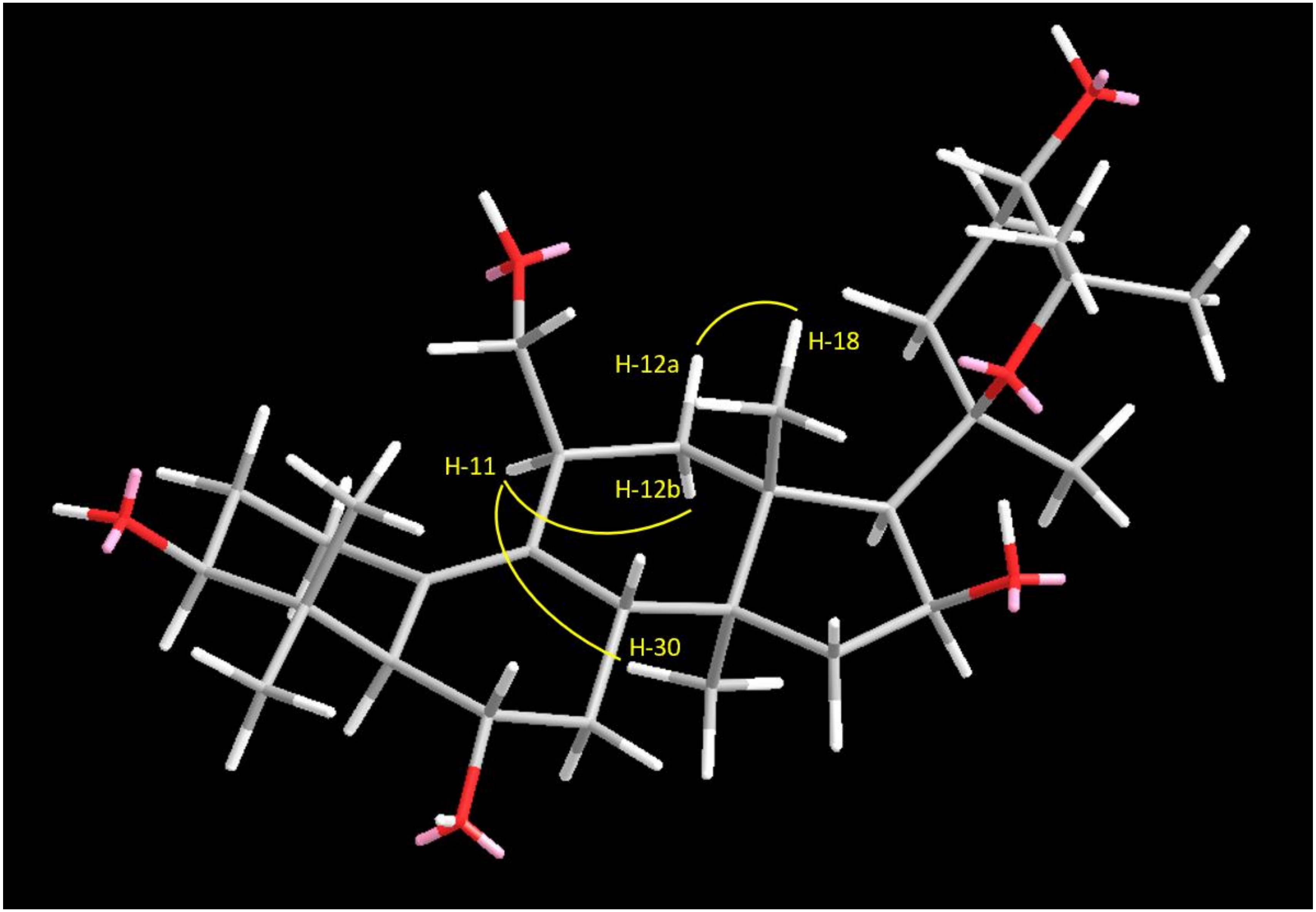

Figure S 178 Key NOE correlations of compound **19**

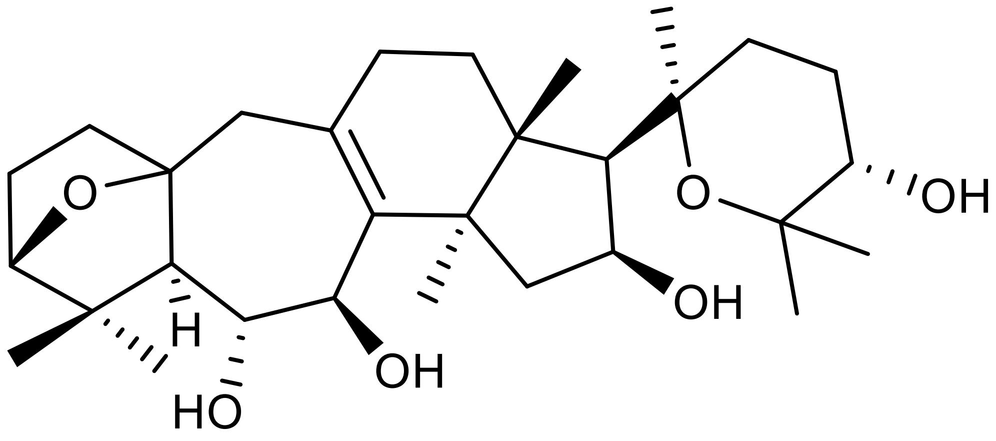

Figure S 179 Structure of compound **20**

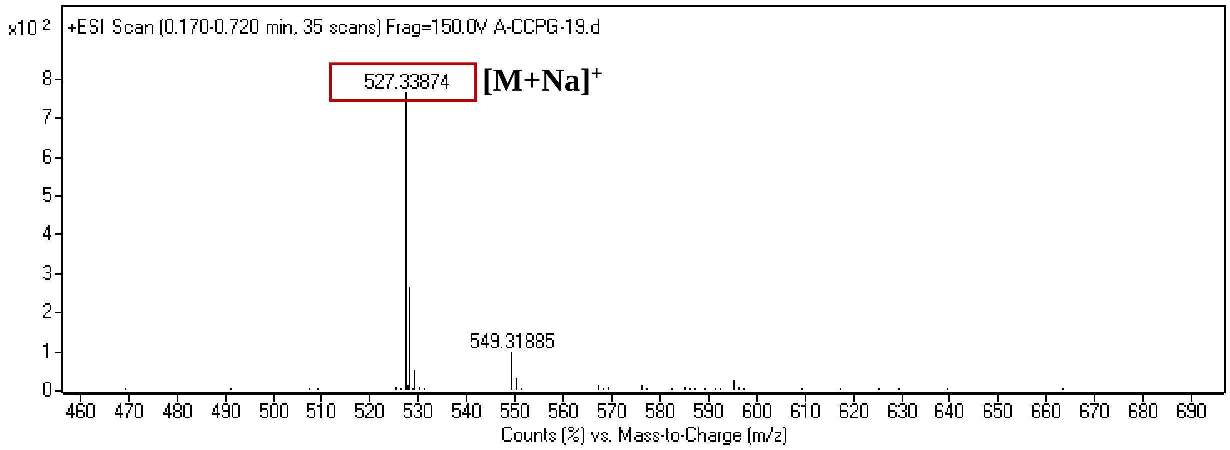

Figure S 180 HR-ESI-MS spectrum of compound **20**

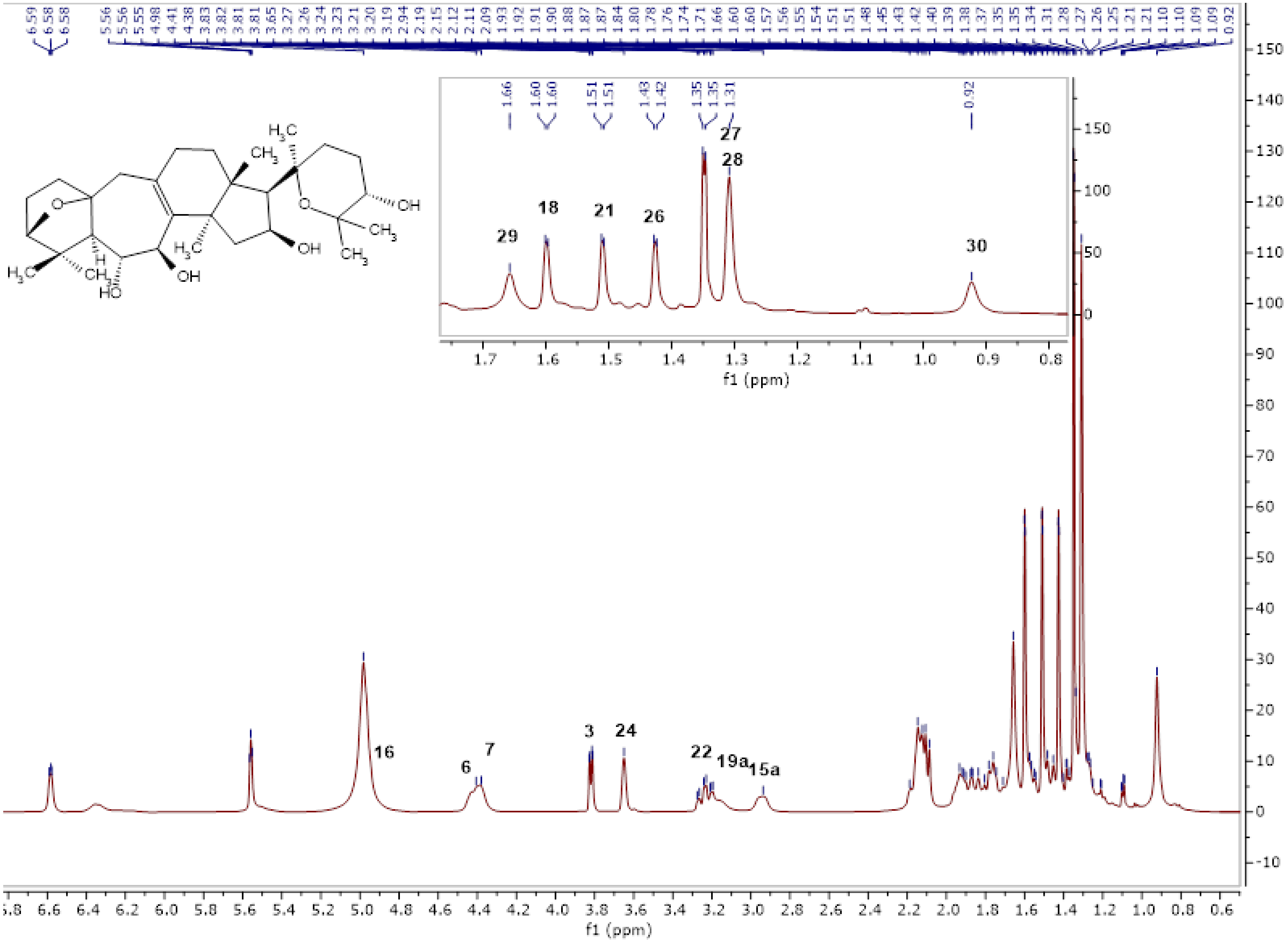

Figure S 181 ^{1}H-NMR spectrum of compound **20** (400 MHz, C_5D_5N)

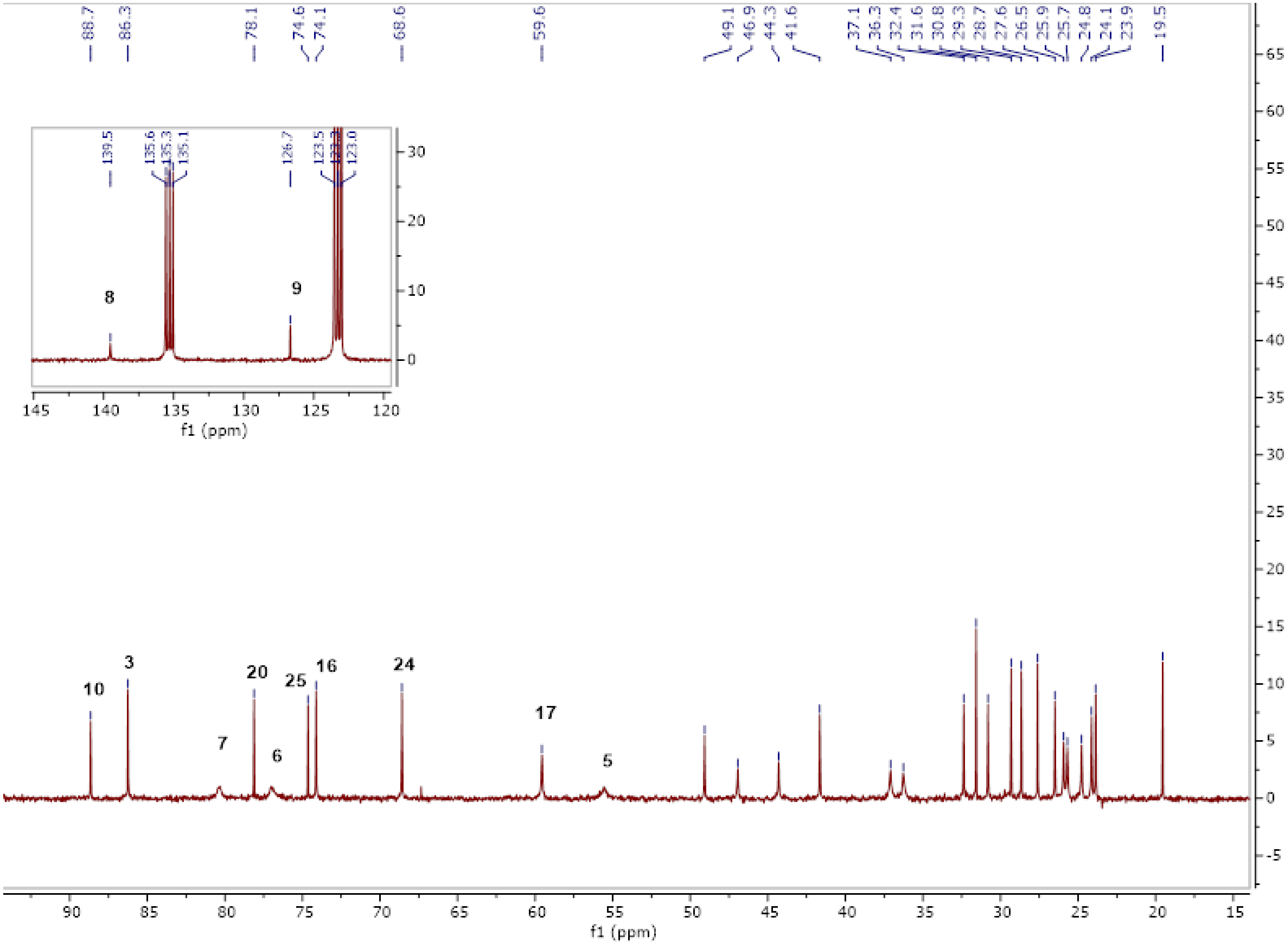

Figure S 182 ^{13}C-NMR spectrum of compound **20** (100 MHz, C_5D_5N)

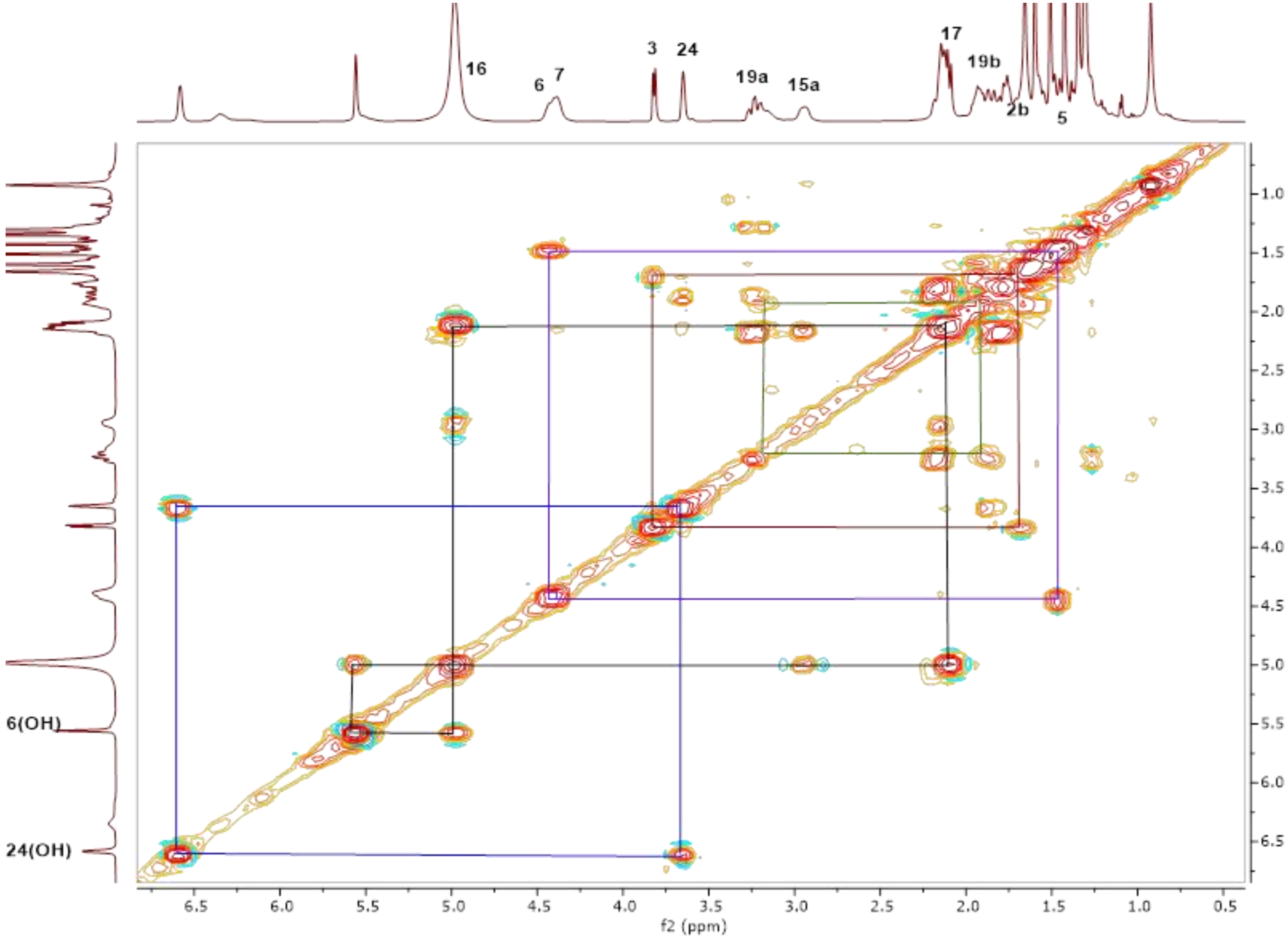

Figure S 183 COSY spectrum of compound **20**

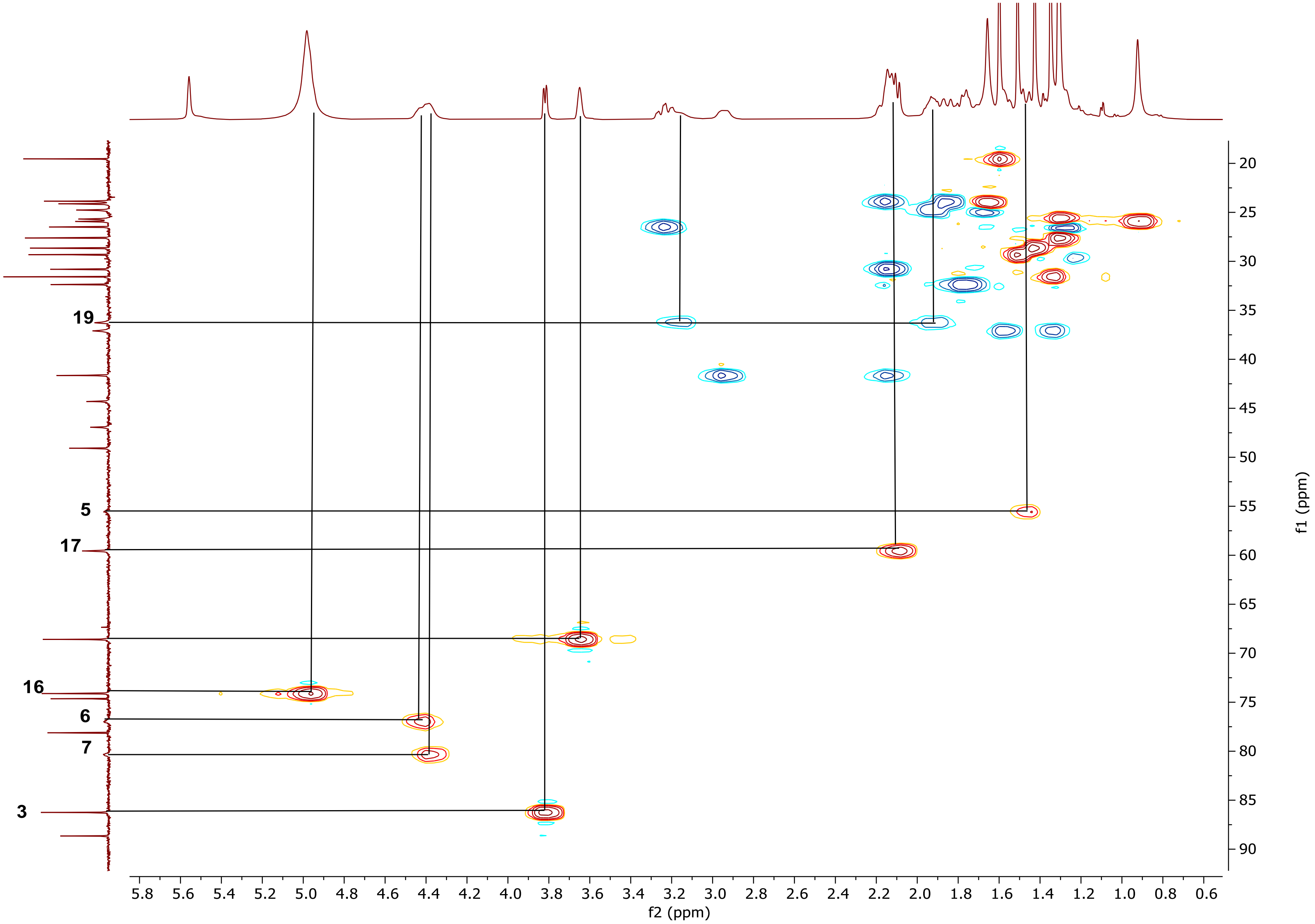

Figure S 184 HSQC spectrum of compound **20**

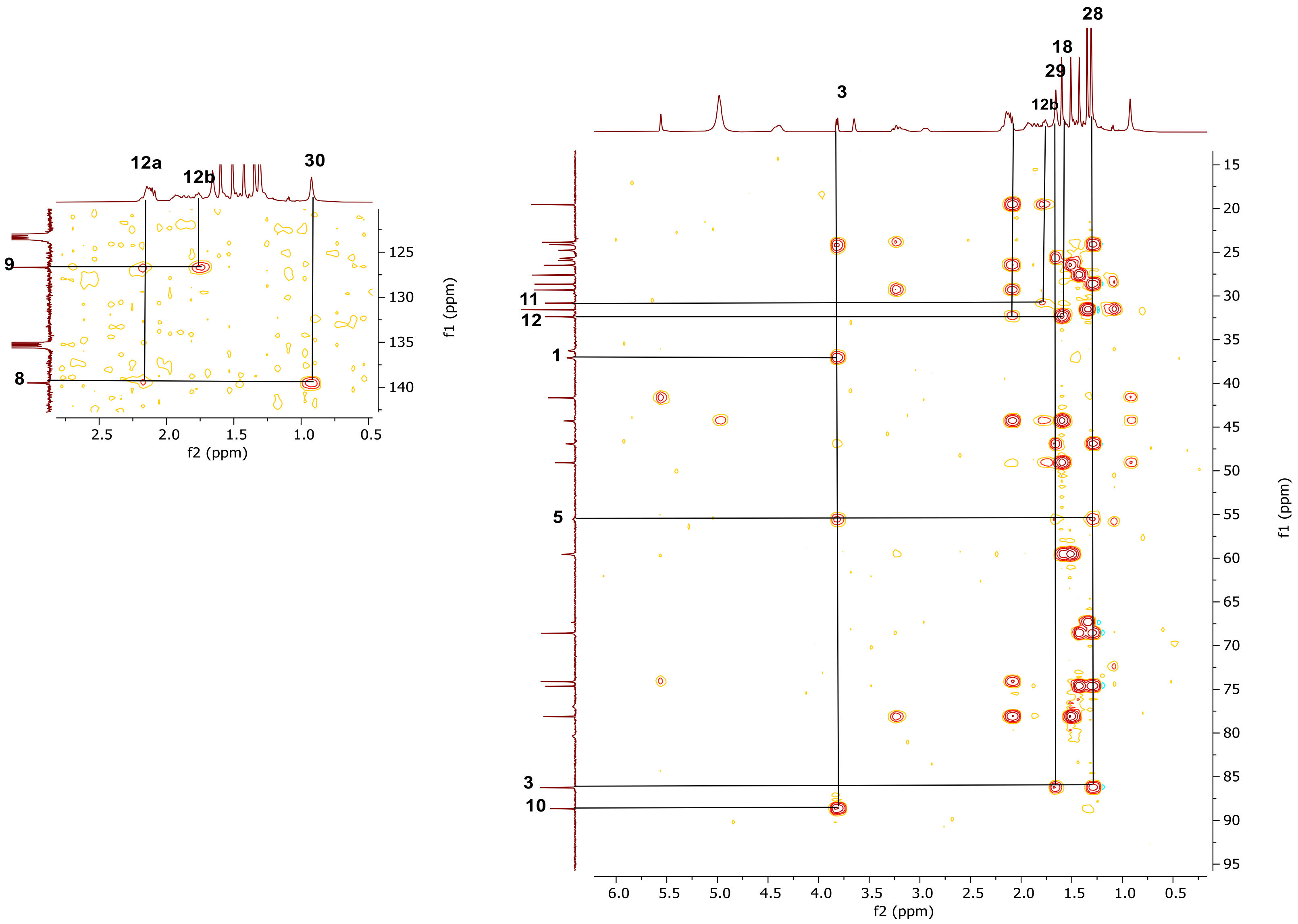

Figure S 185 HMBC spectrum of compound **20**

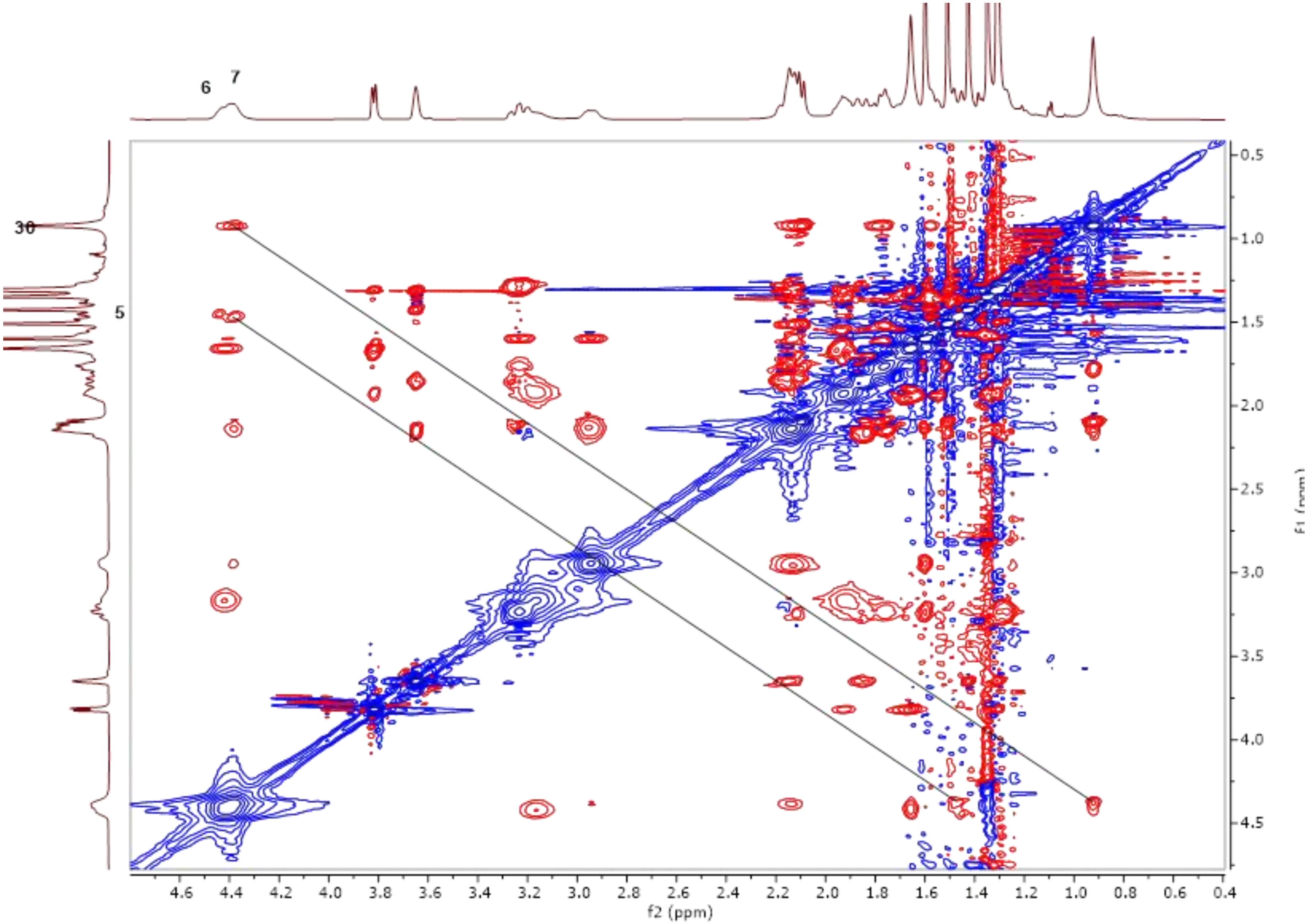

Figure S 186 NOESY spectrum of compound **20**

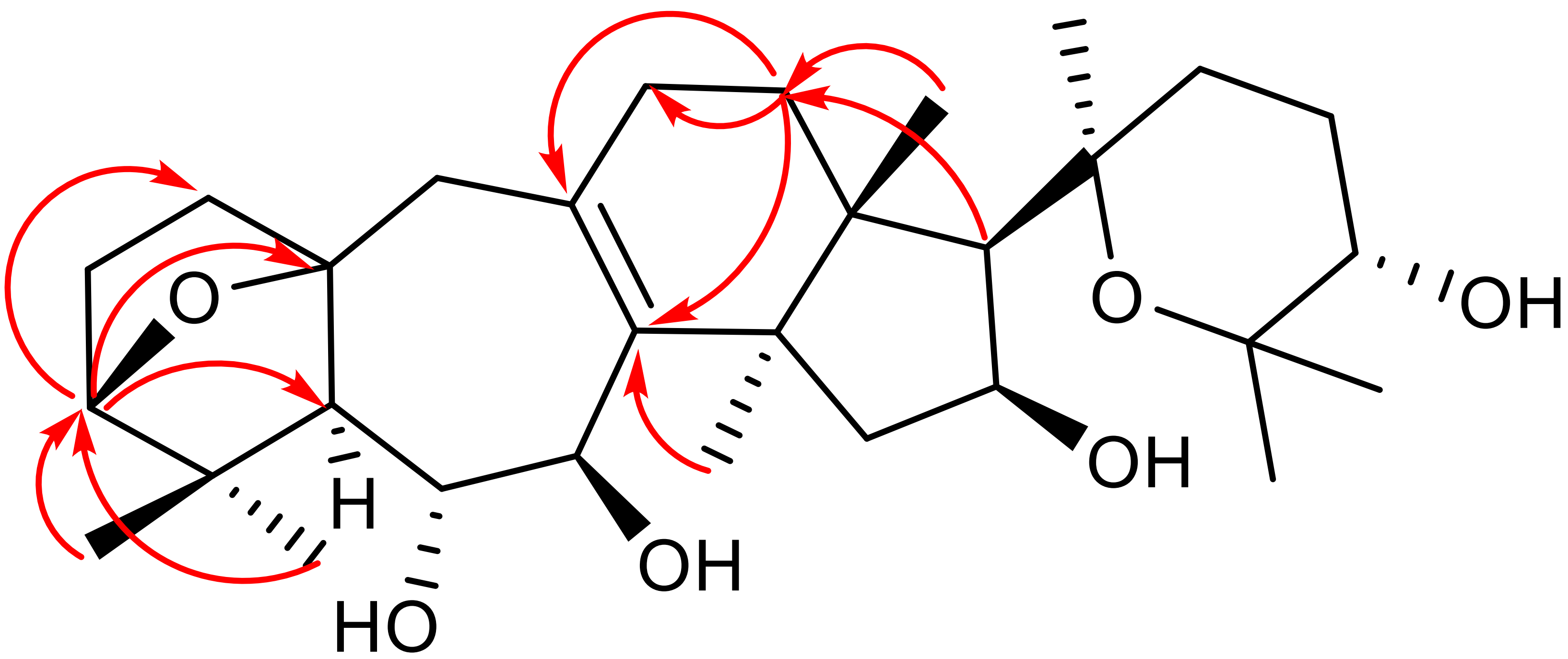

Figure S 187 Key HMBC correlations of compound **20** (arrows from H to C)

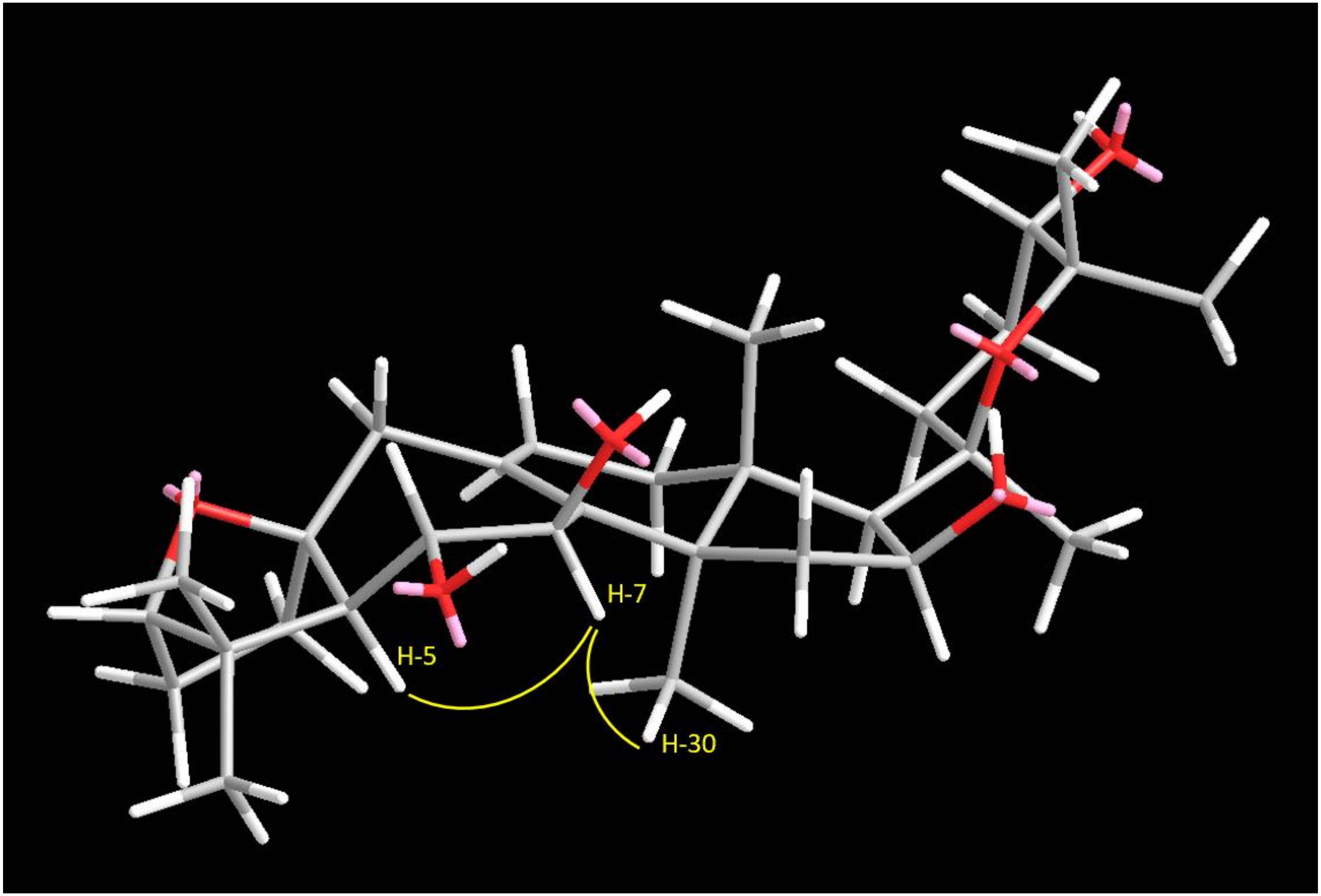

Figure S 188 Key NOE correlations of compound **20**

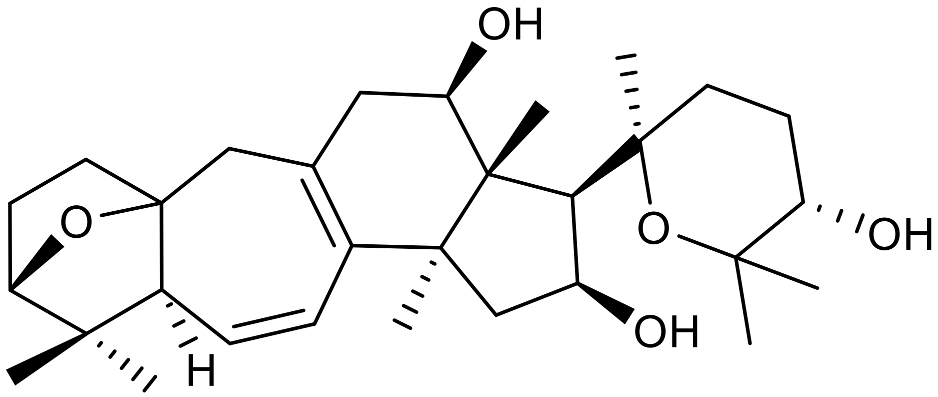

Figure S 189 Structure of compound **21**

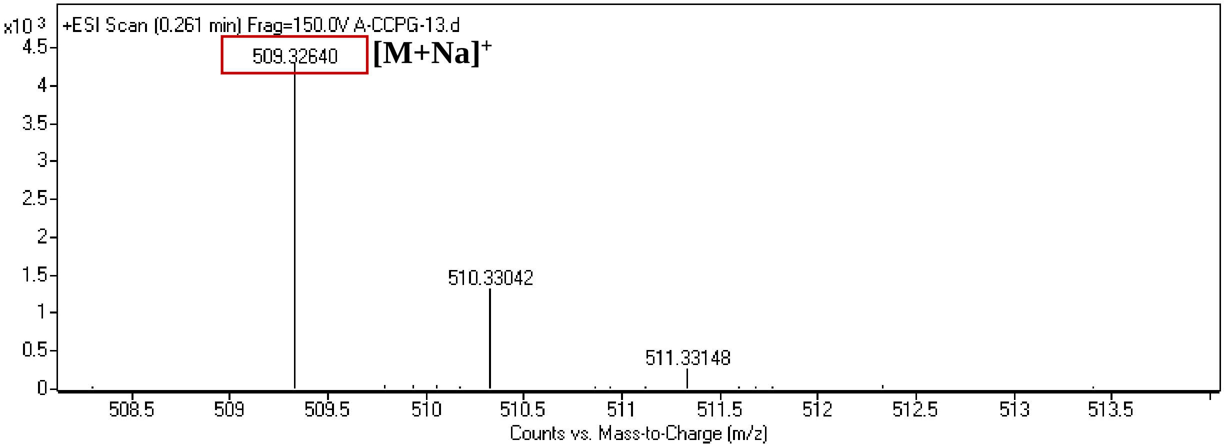

Figure S 190 HR-ESI-MS spectrum of compound **21**

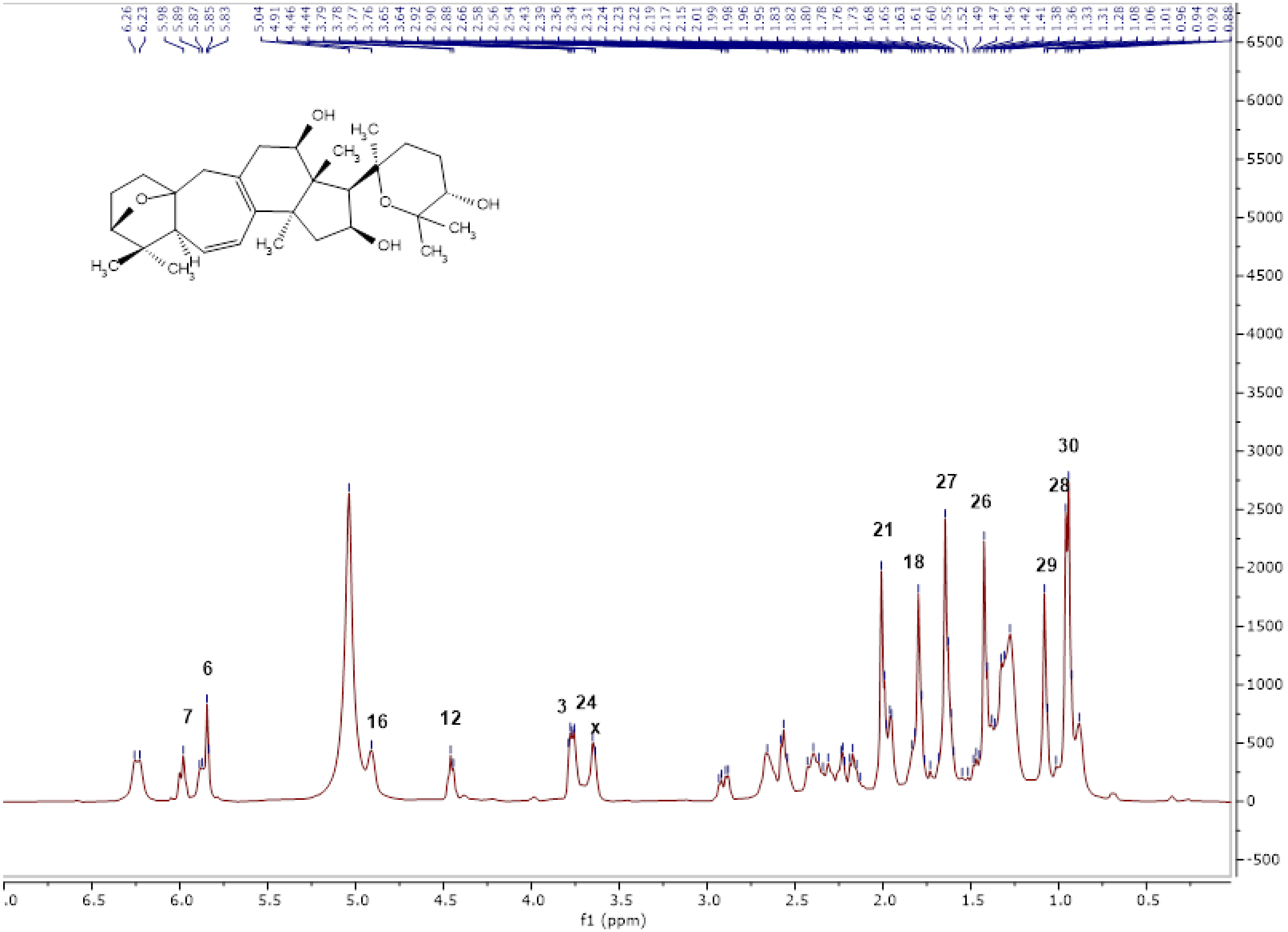

Figure S 191 ¹H-NMR spectrum of compound **21** (500 MHz, C_5D_5N)

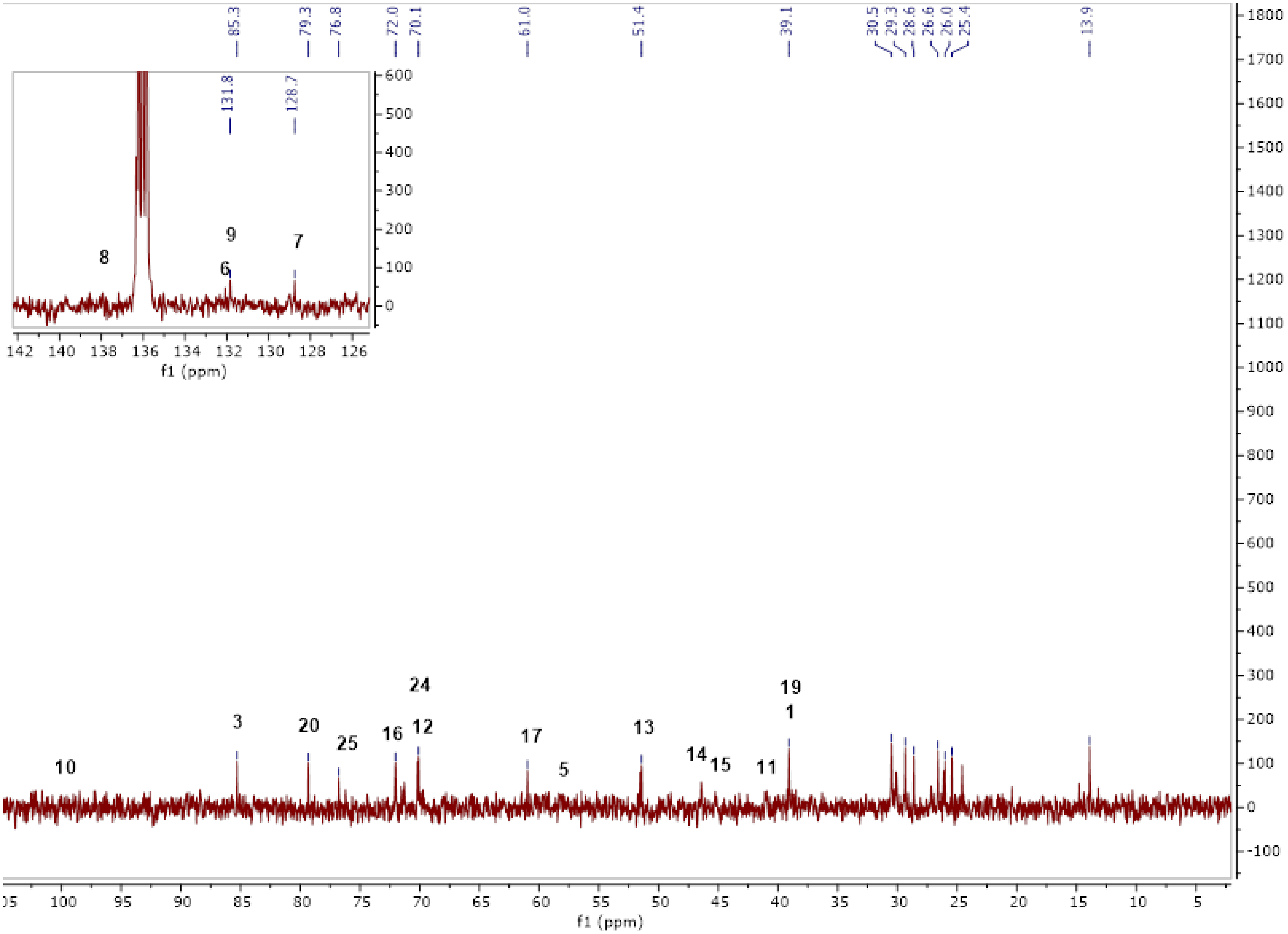

Figure S 192 ^{13}C-NMR spectrum of compound **21** (125 MHz, C_5D_5N)

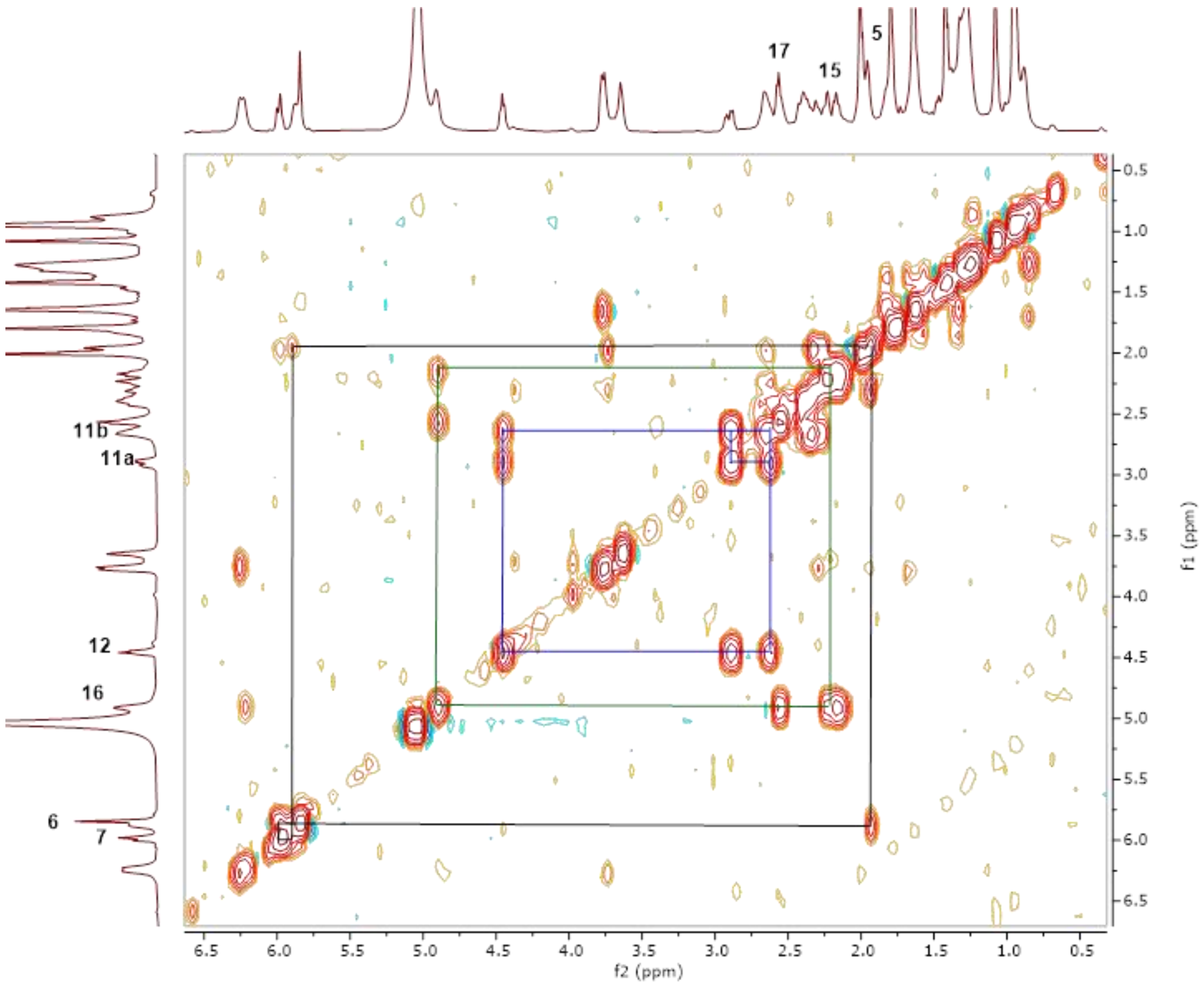

Figure S 193 COSY spectrum of compound **21**

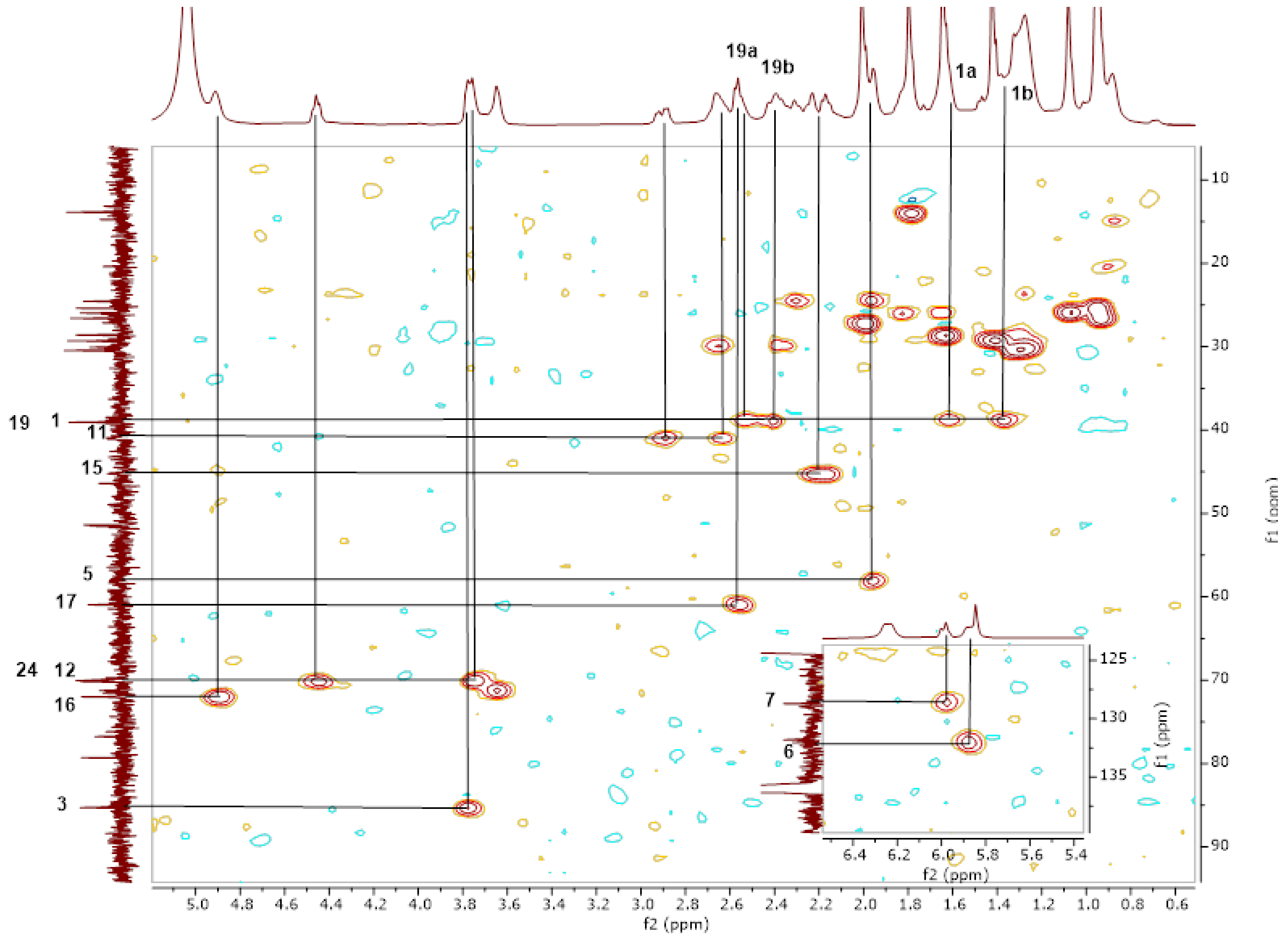

Figure S 194 HSQC spectrum of compound **21**

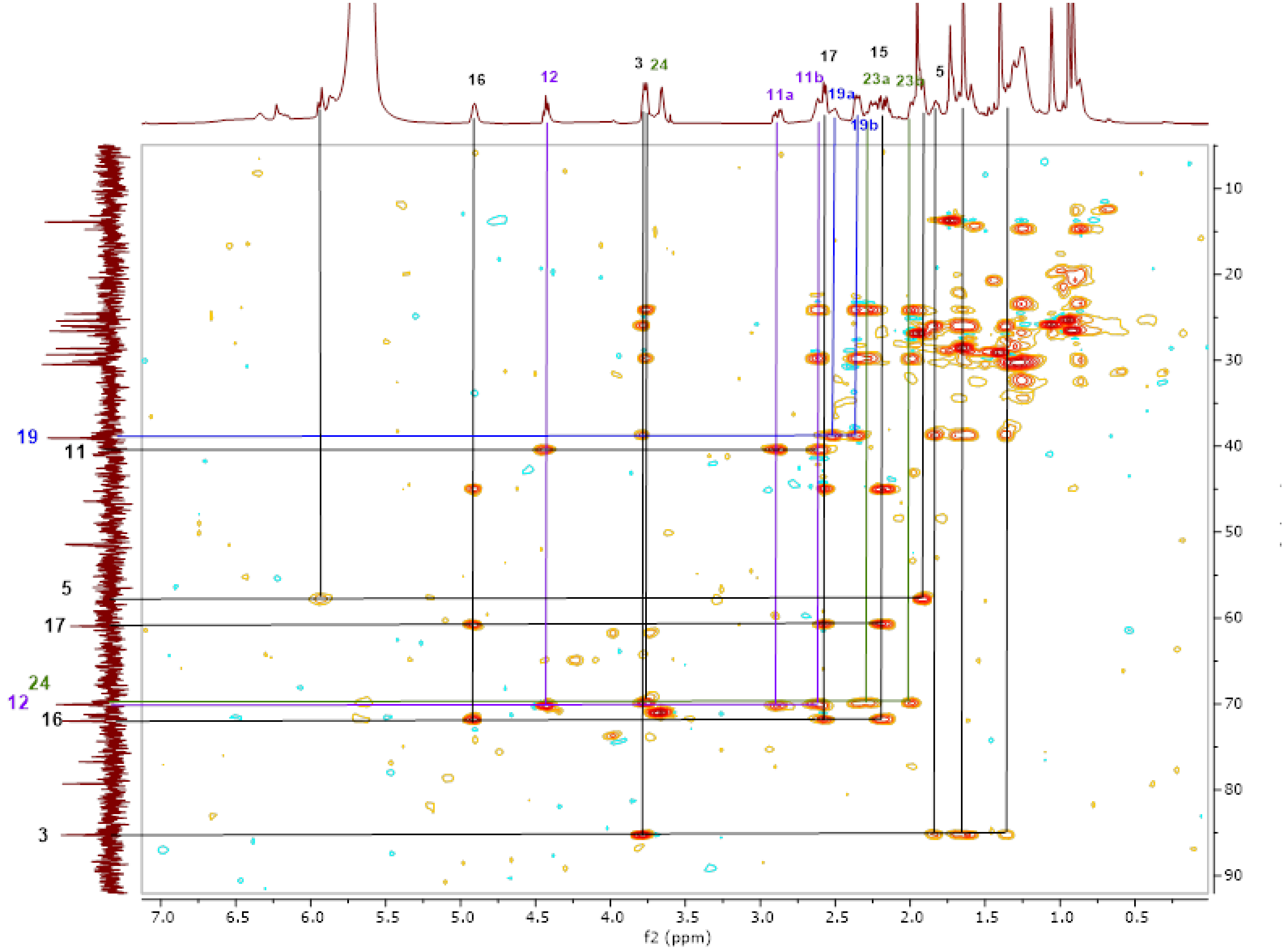

Figure S 195 HSQC-TOCSY spectrum of compound **21**

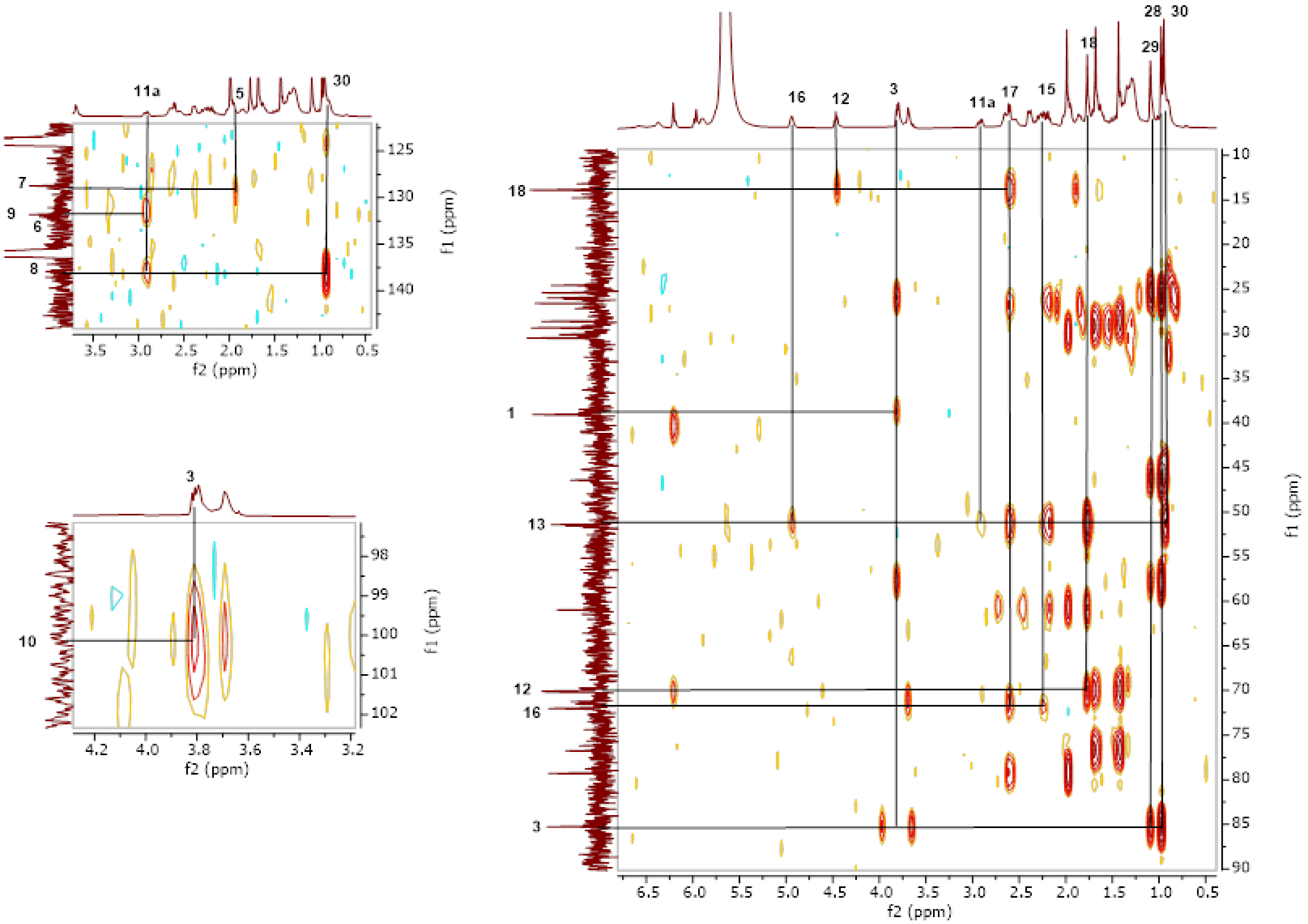

Figure S 196 HMBC spectrum of compound **21**

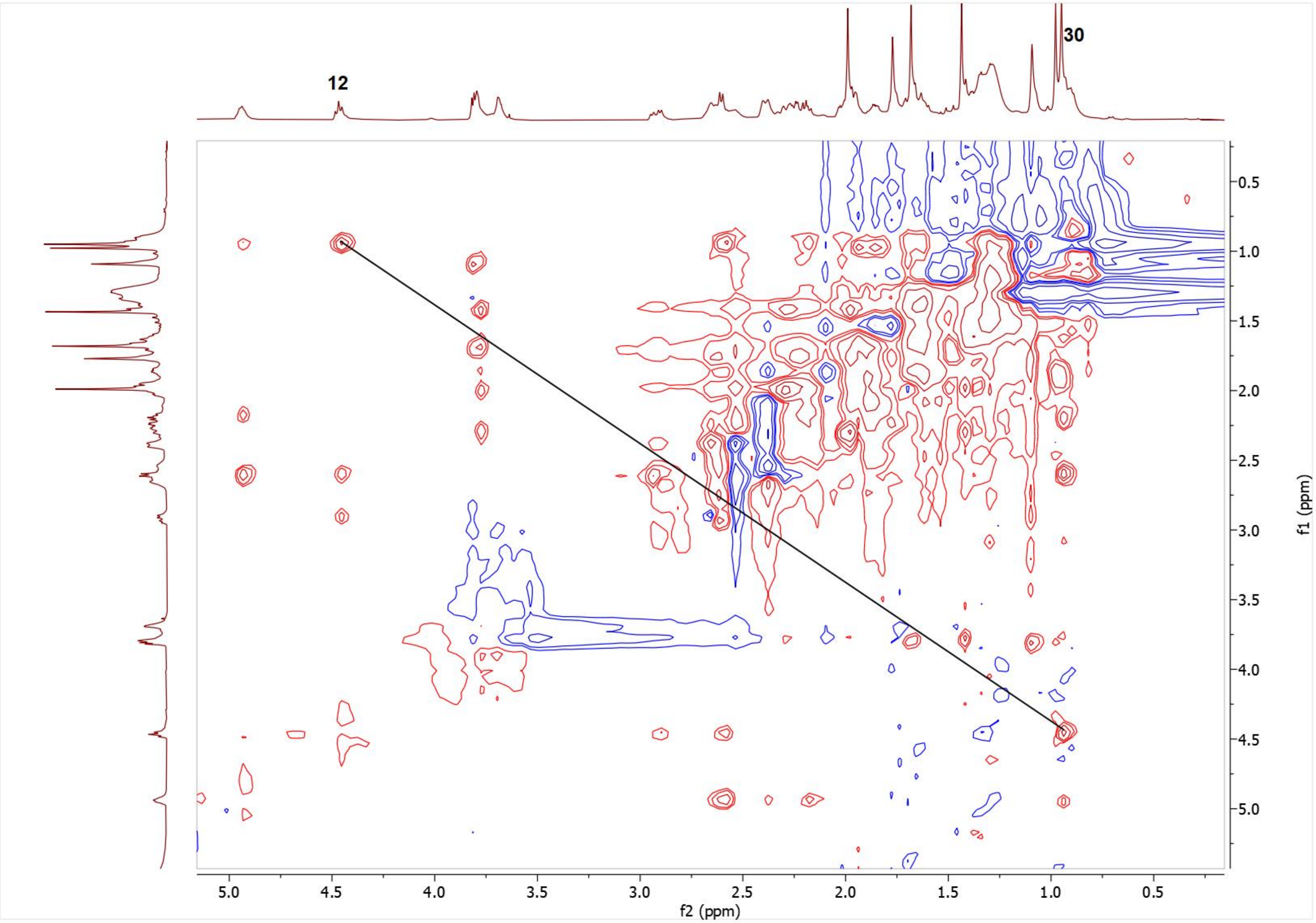

Figure S 197 NOESY spectrum of compound **21**

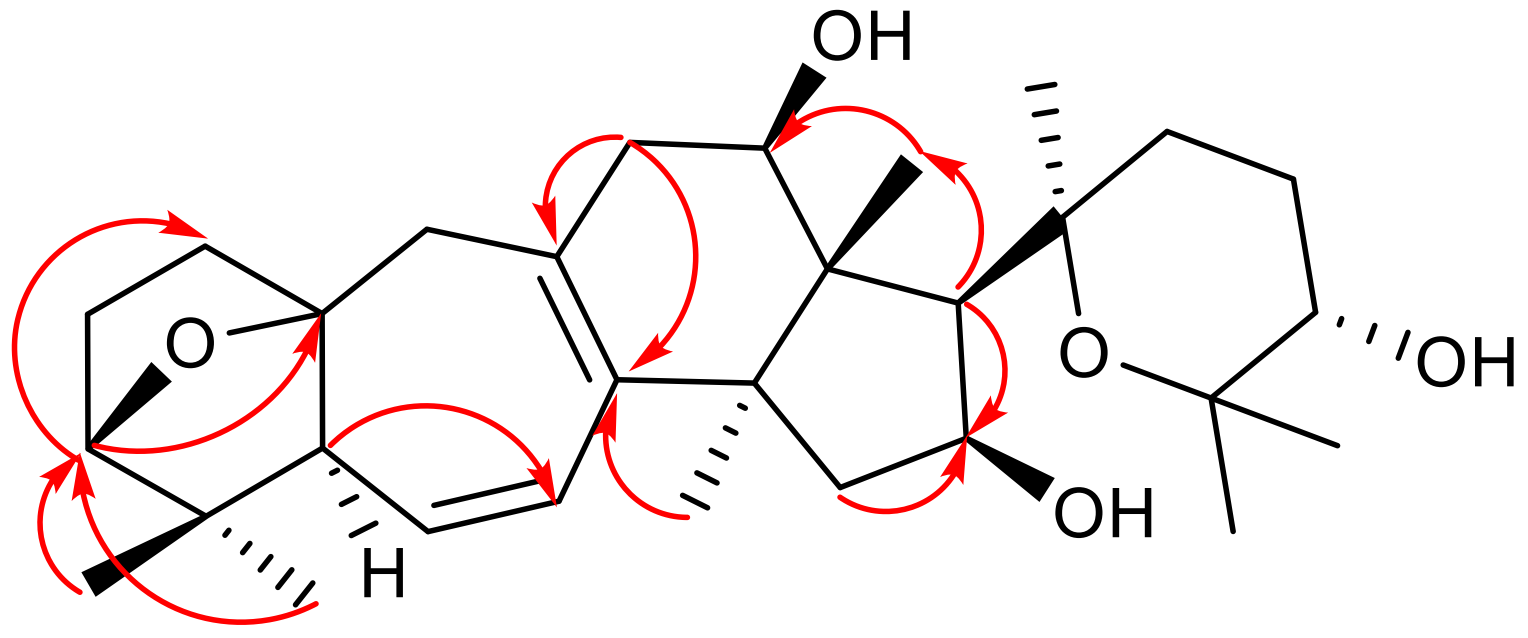

Figure S 198 Key HMBC correlations of compound **21** (arrows from H to C)

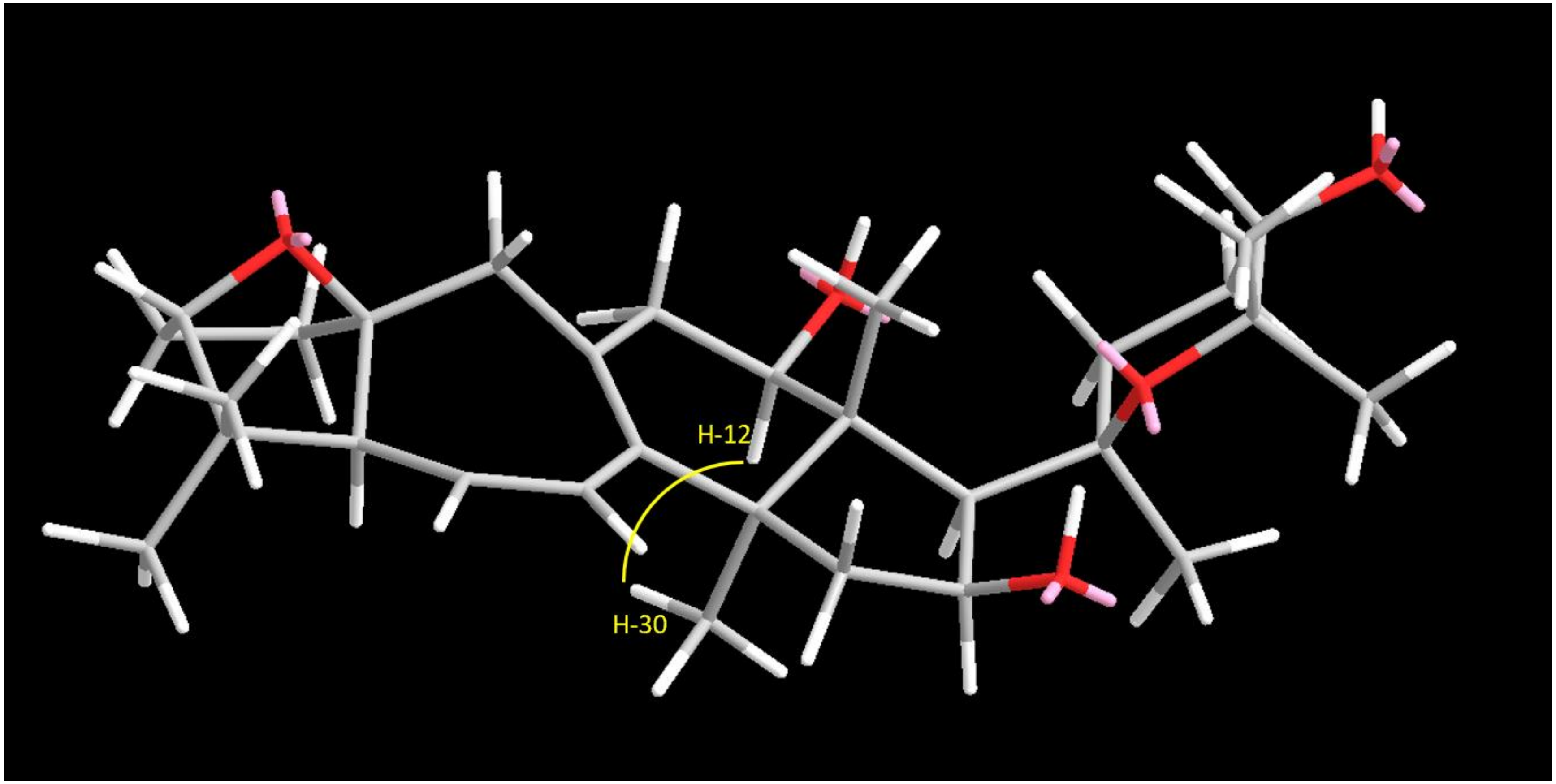

Figure S 199 Key NOE correlation of compound **21**

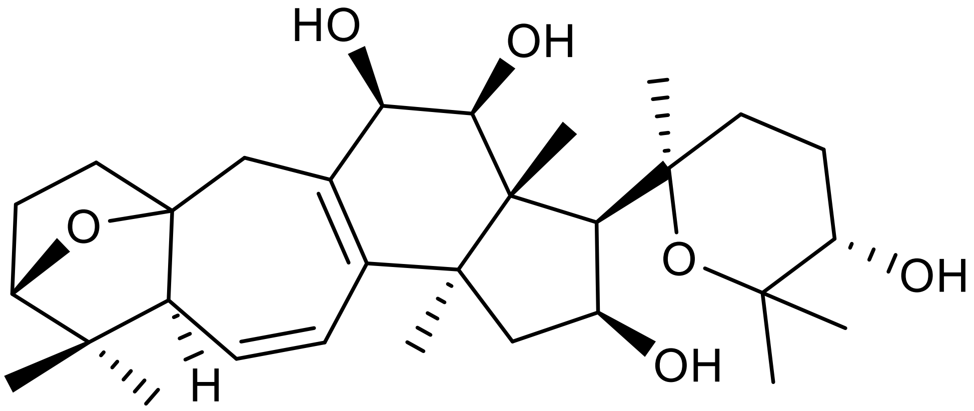

Figure S 200 Structure of compound **22**

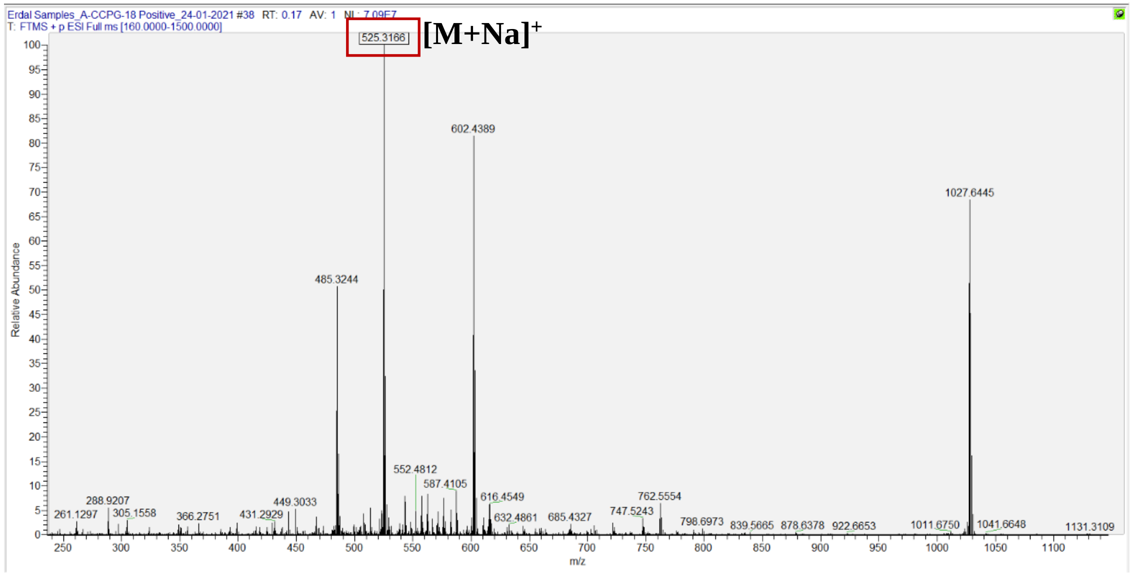

Figure S 201 HR-ESI-MS spectrum of compound **22**

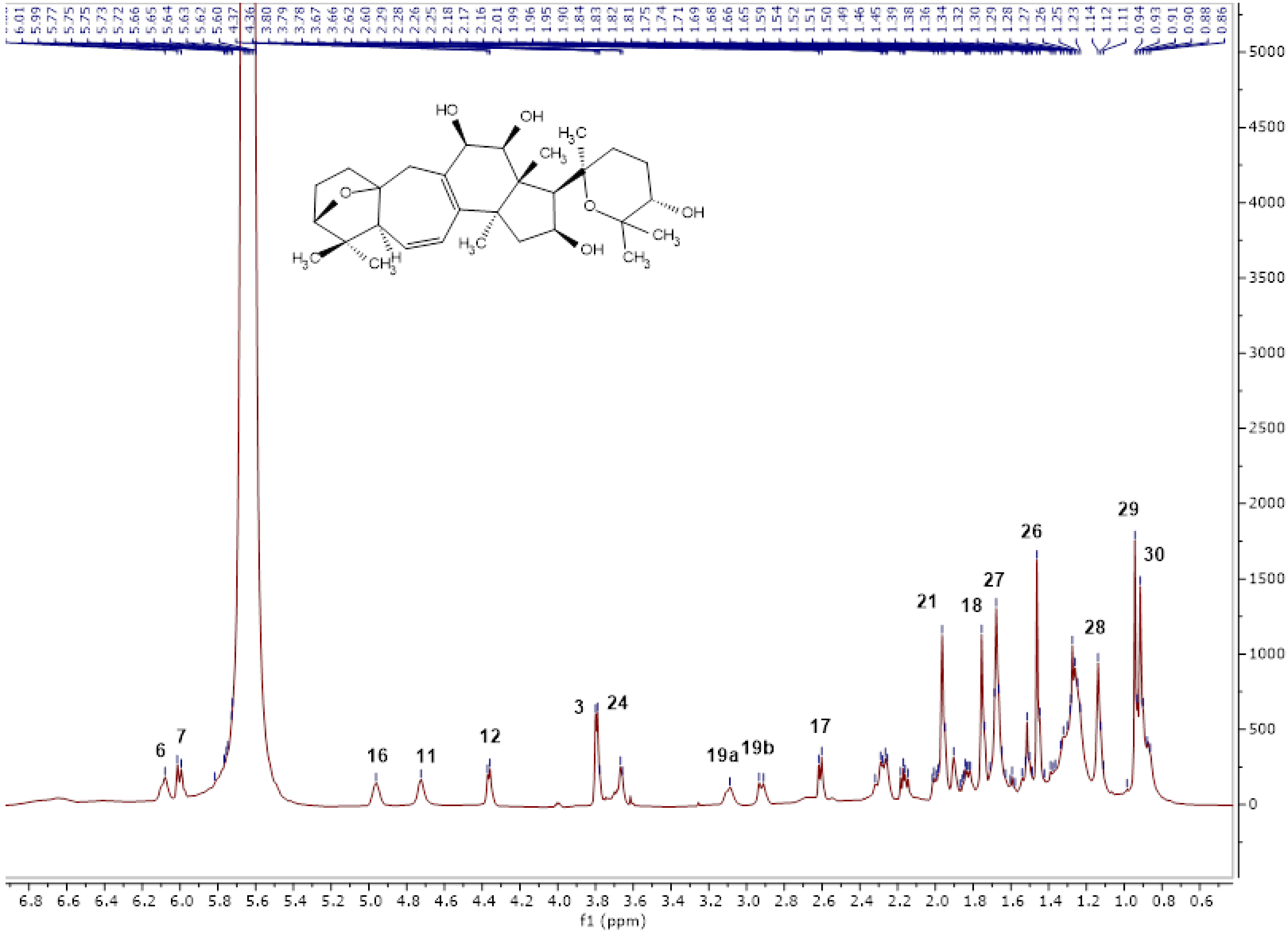

Figure S 202 ^{1}H-NMR spectrum of compound **22** (500 MHz, C_5D_5N)

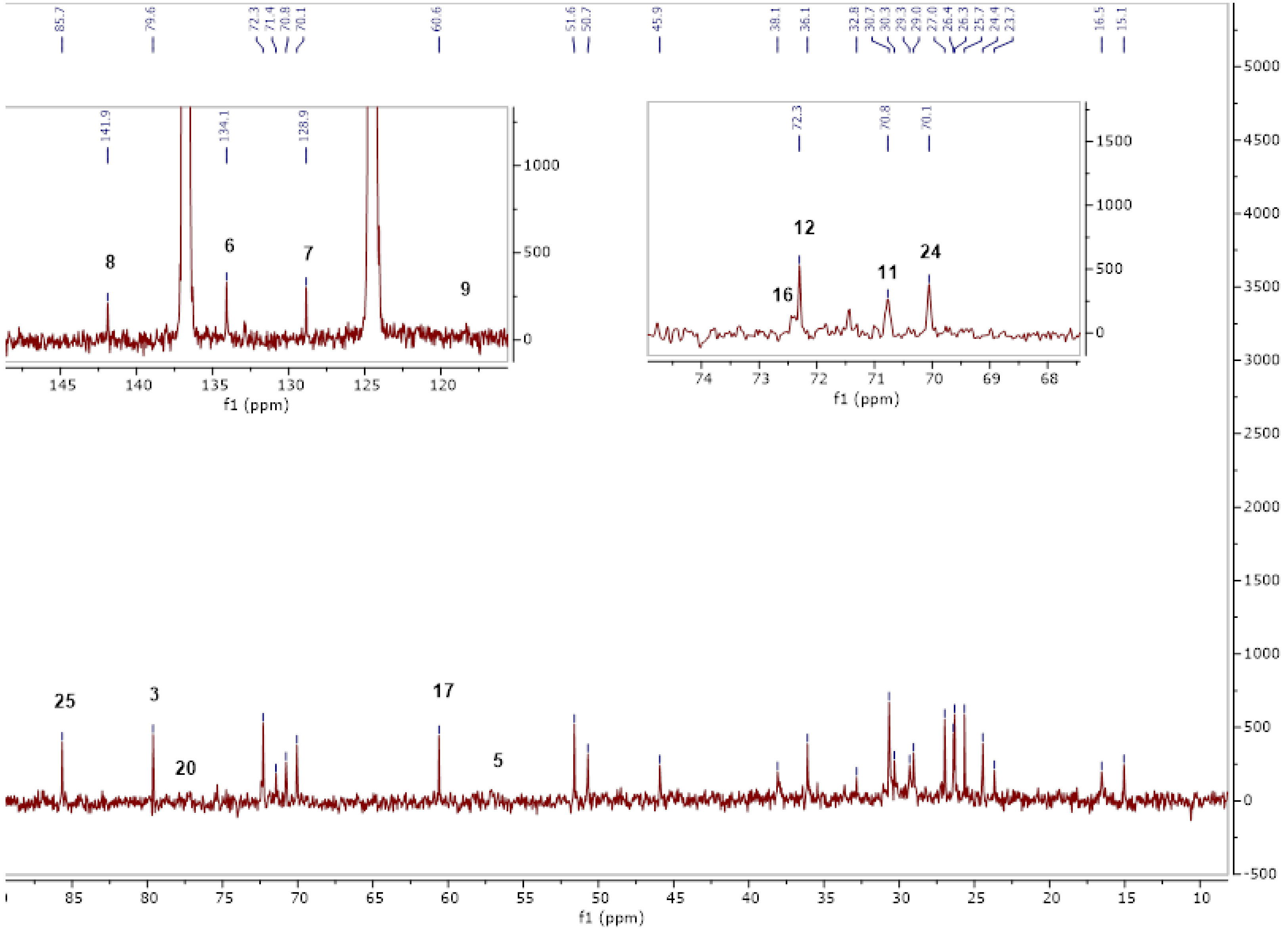

Figure S 203 ^{13}C-NMR spectrum of compound **22** (125 MHz, C_5D_5N)

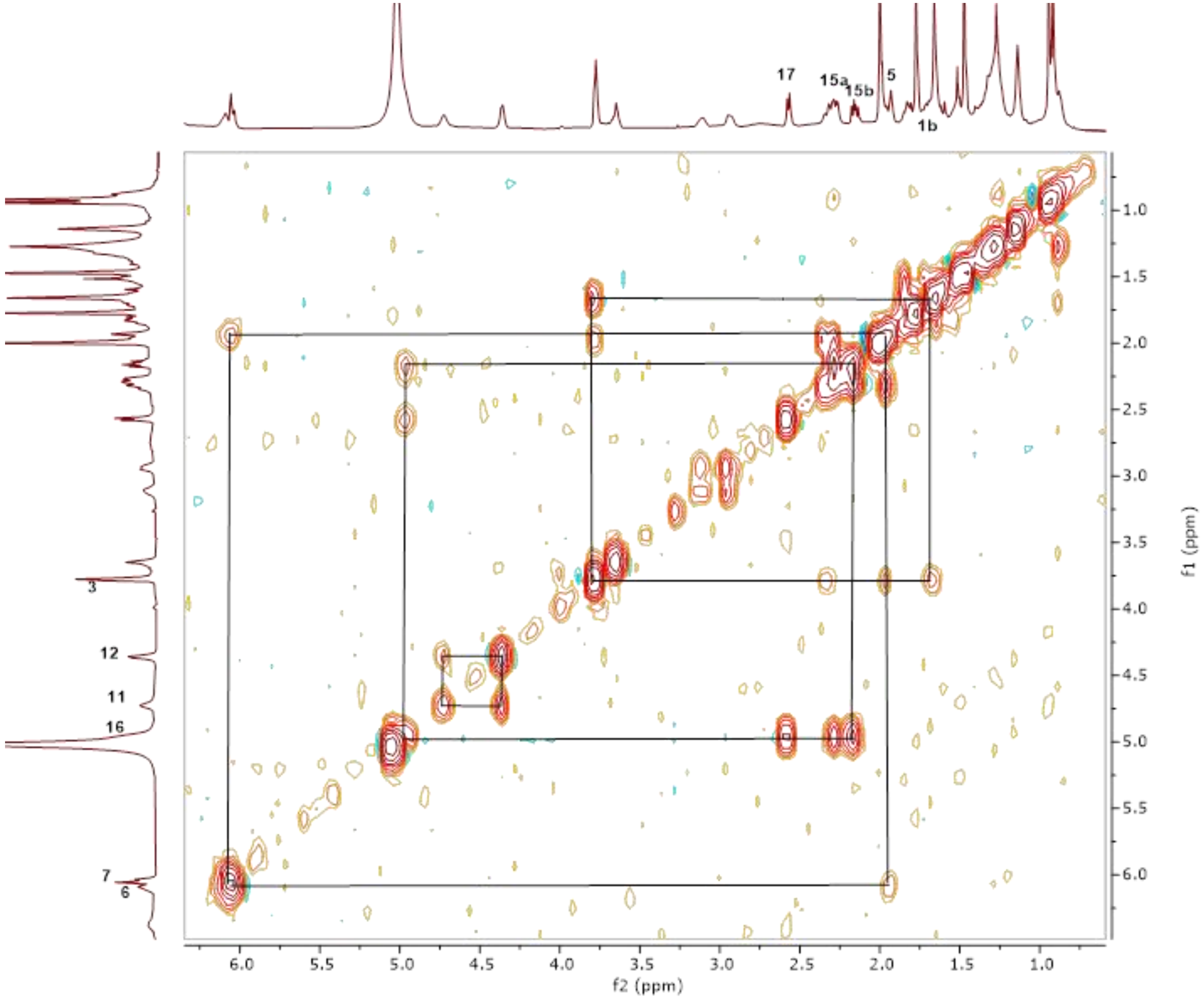

Figure S 204 COSY spectrum of compound **22**

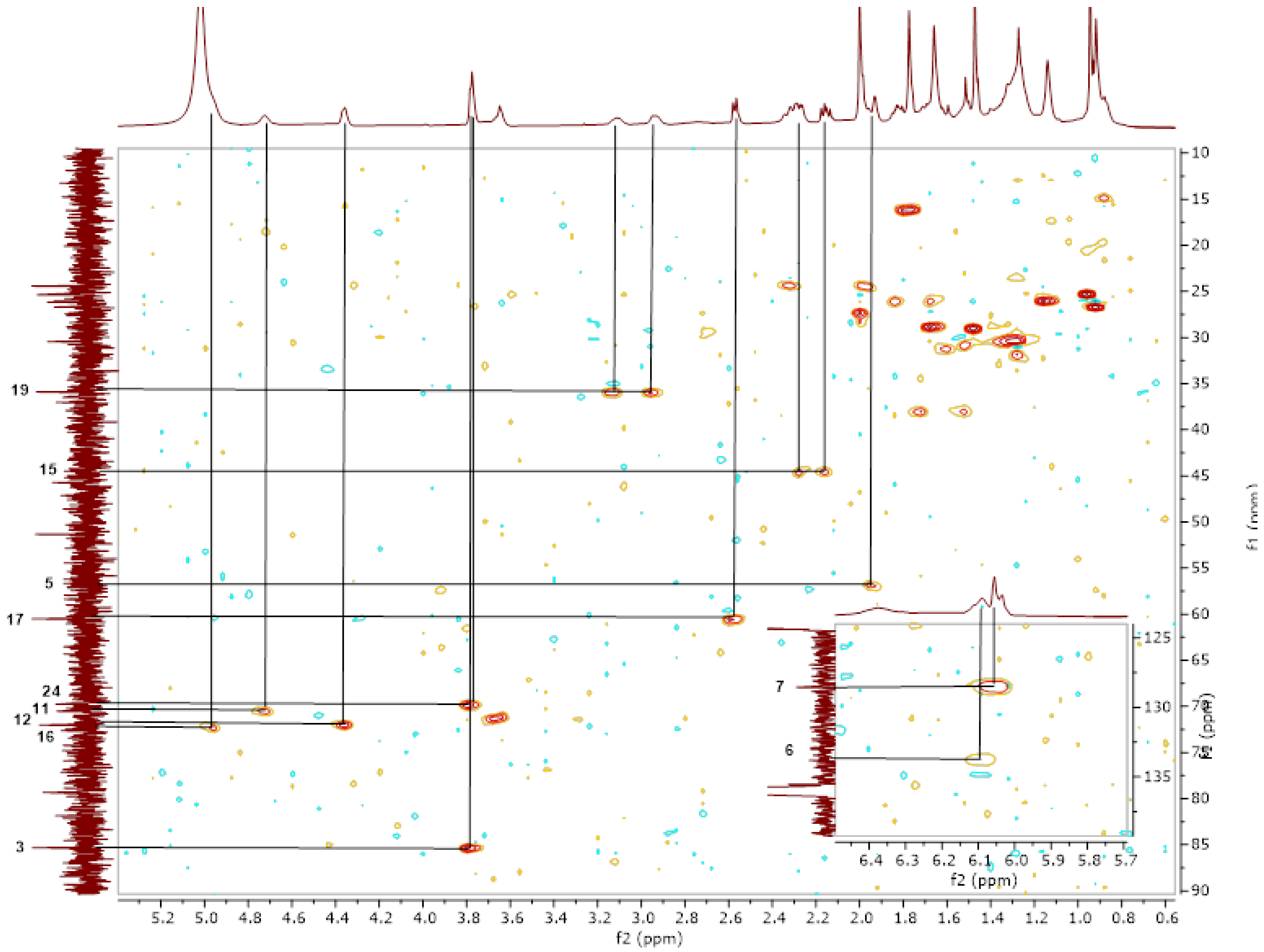

Figure S 205 HSQC spectrum of compound **22**

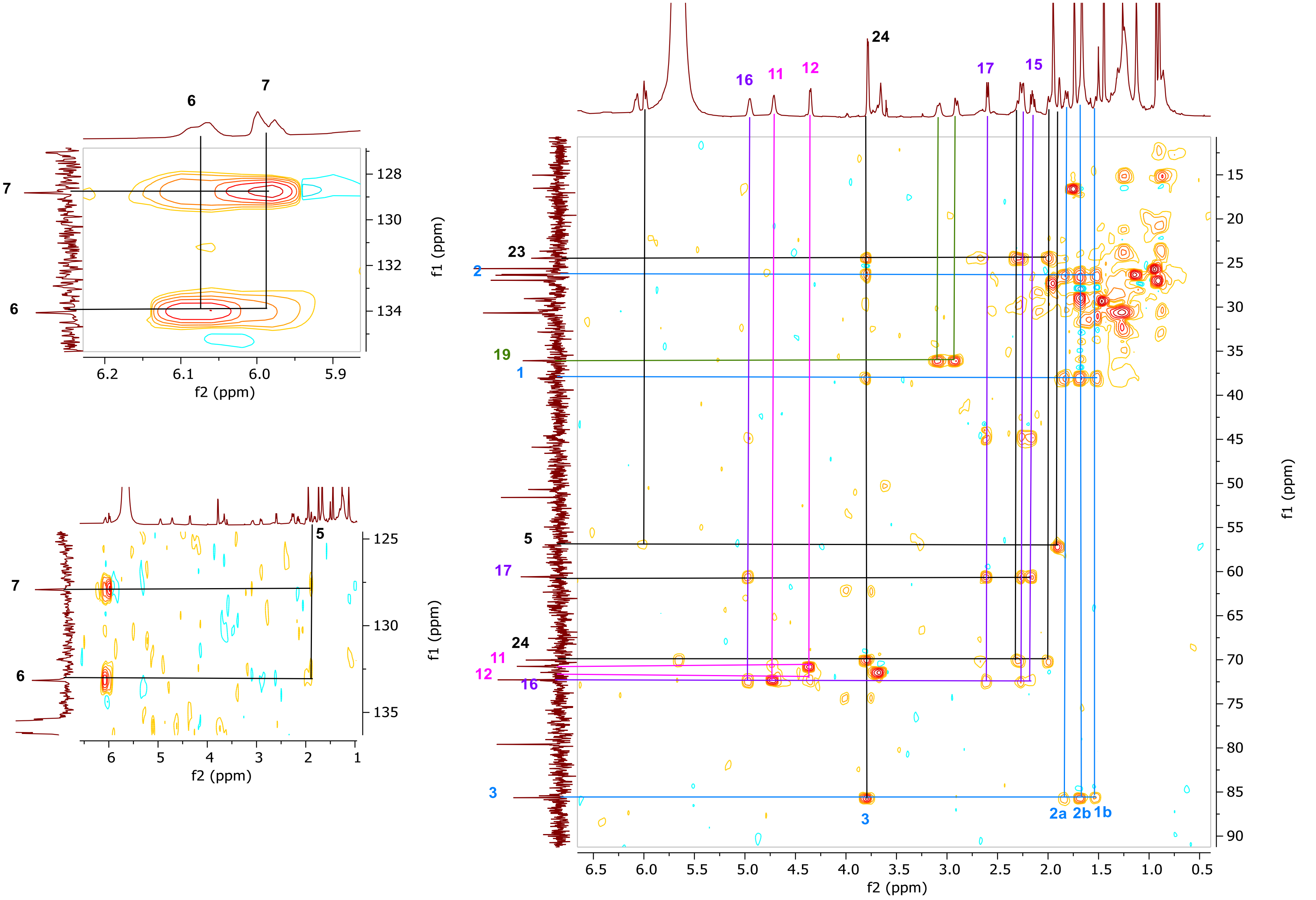

Figure S 206 HSQC-TOCSY spectrum of compound **22**

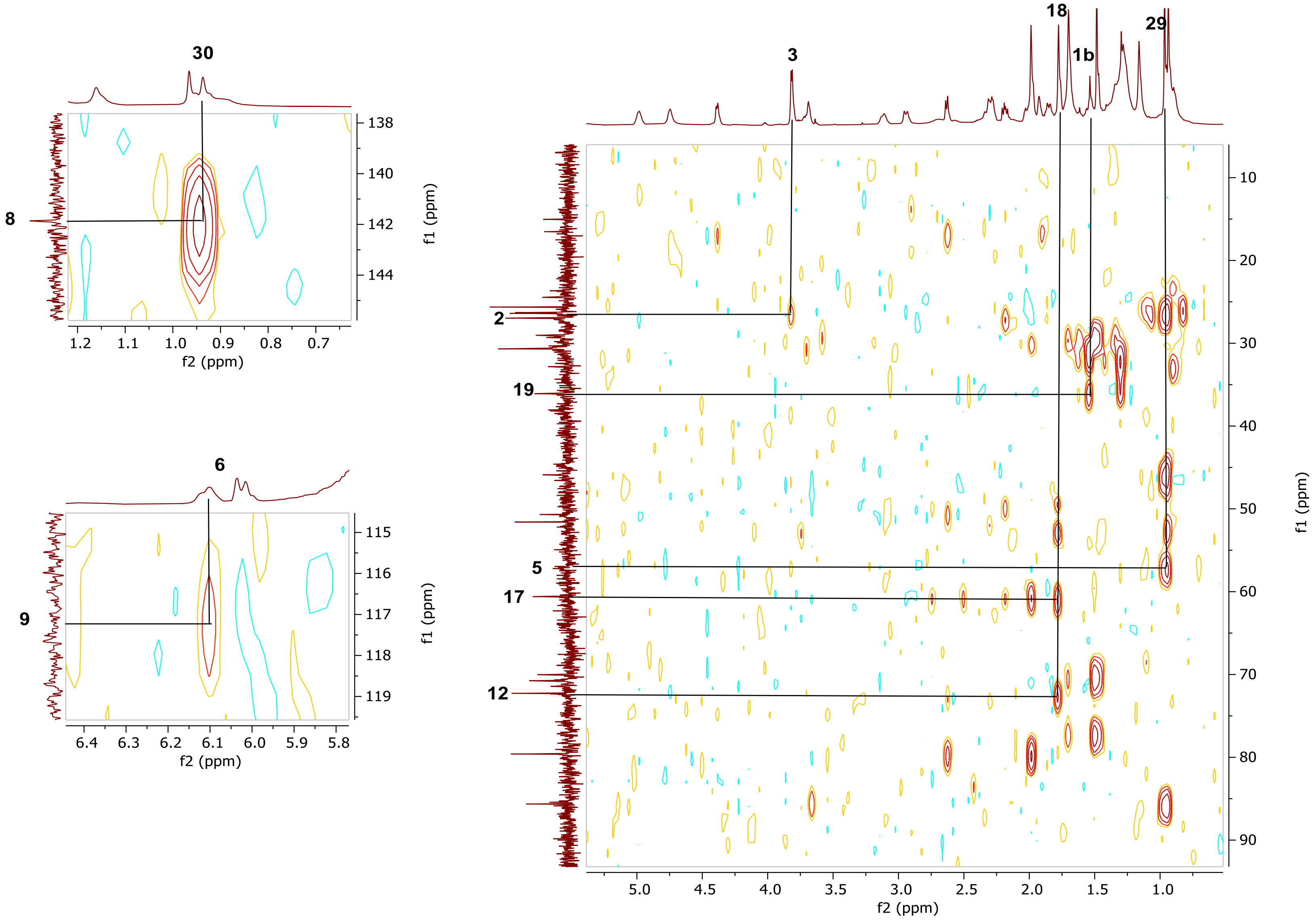

Figure S 207 HMBC spectrum of compound **22**

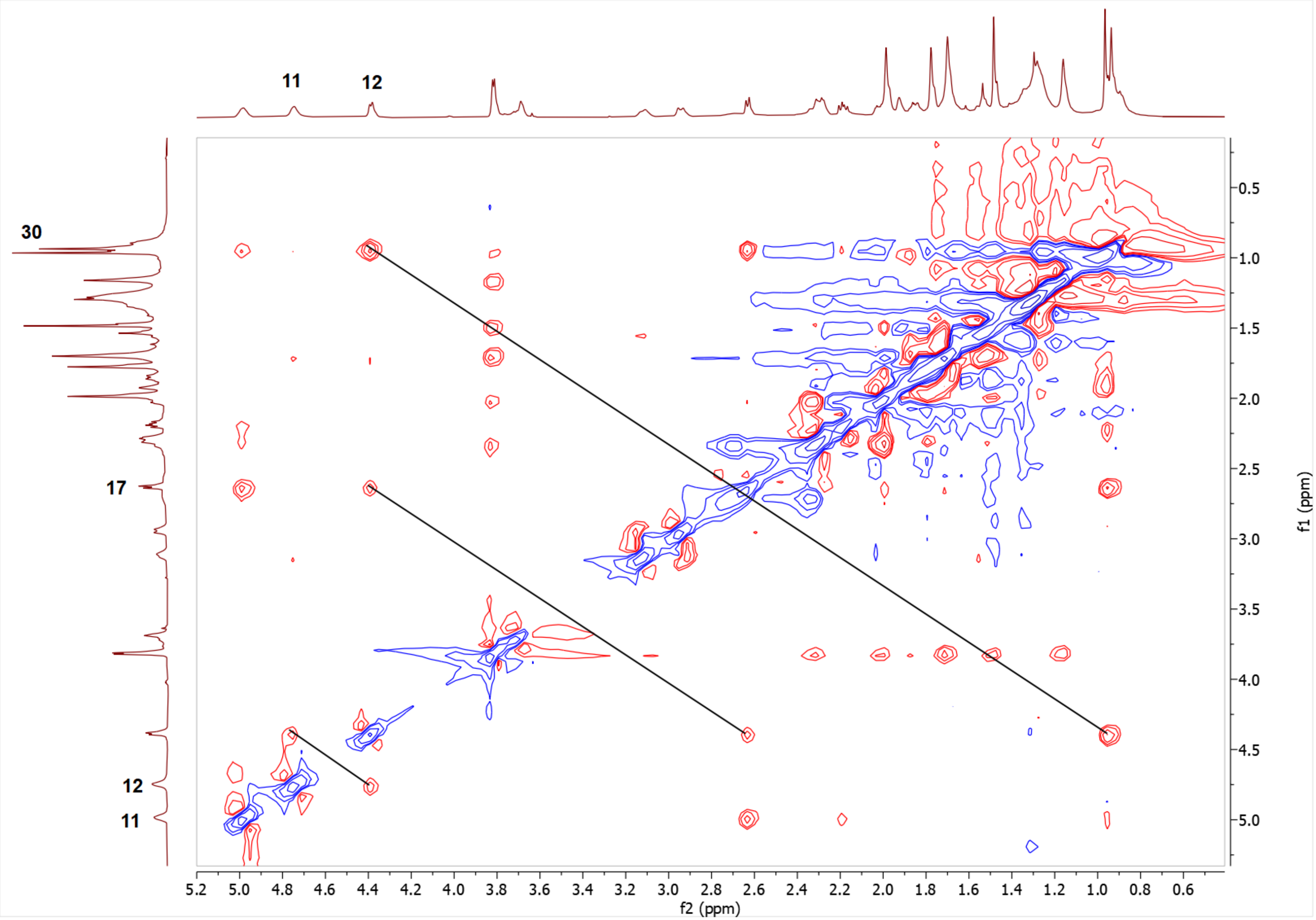

Figure S 208 NOESY spectrum of compound **22**

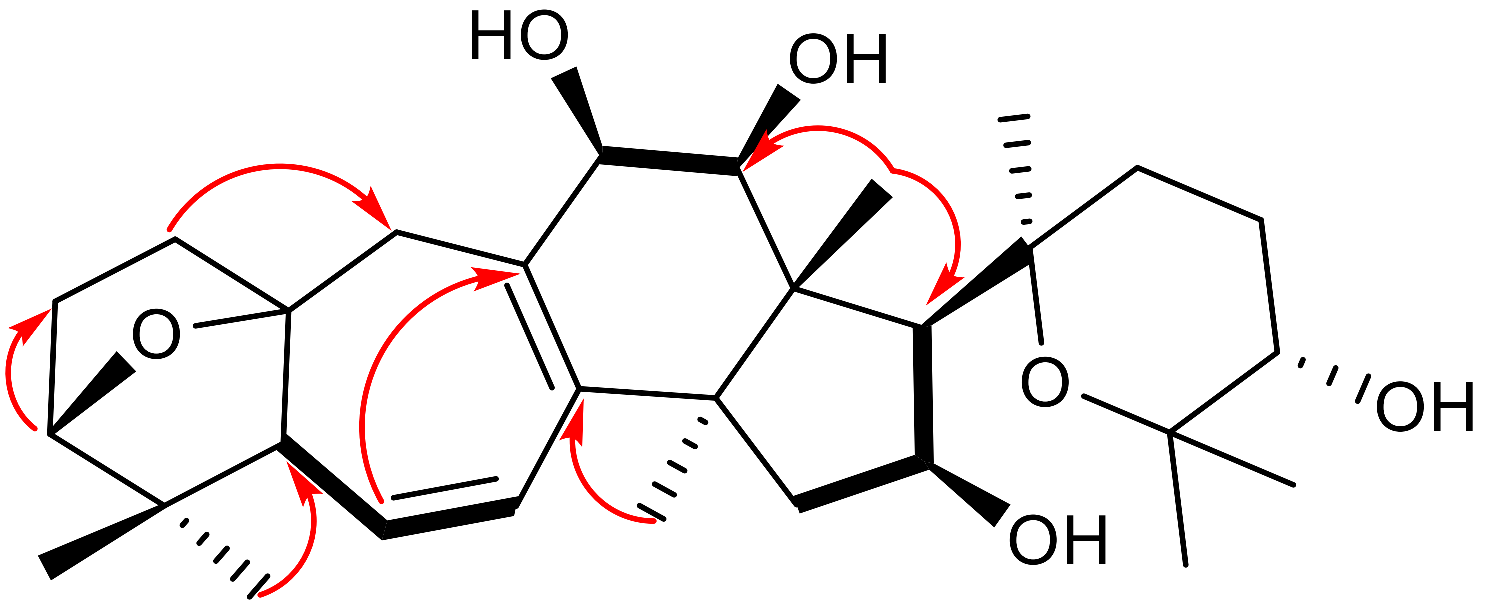

Figure S 209 Key HMBC correlations of compound **22** (arrows from H to C)

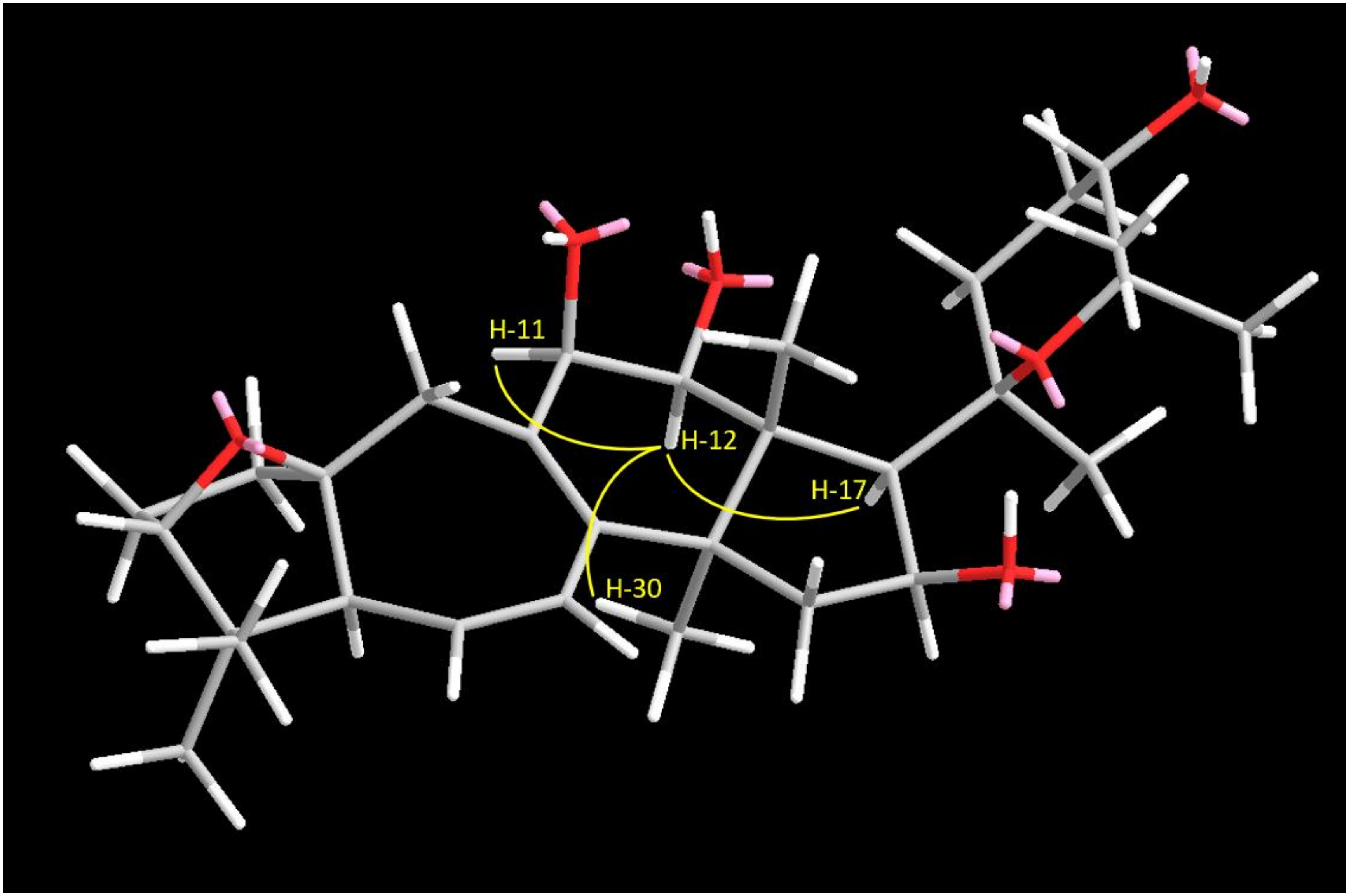

Figure S 210 Key NOE correlations of compound **22**

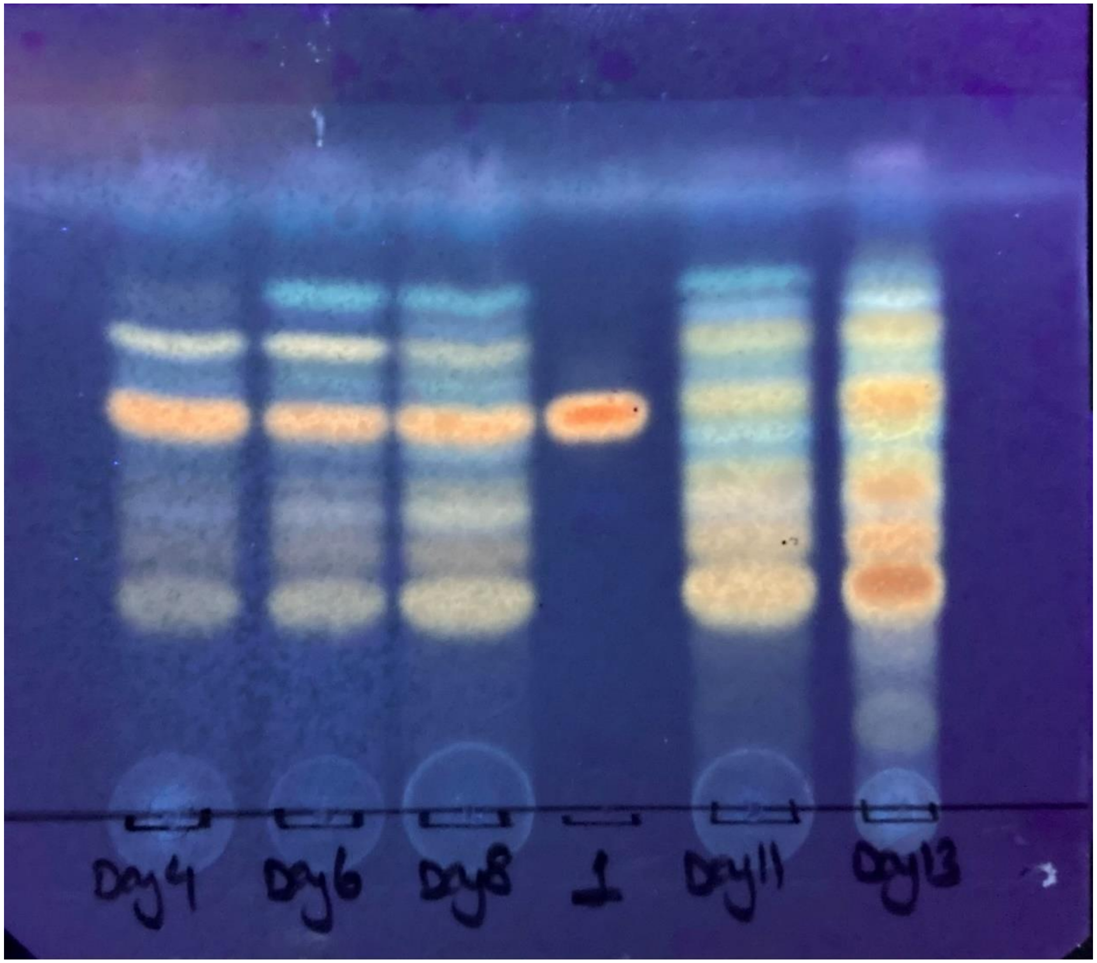

Figure S 211 Thin layer chromatogram of EtOAc extracts for different biotransformation time points (Day 4, 6, 8, 11 and 13) [(Silica gel, Mobile phase: 87.5:12.5 ($CHCl_3$:MeOH)]

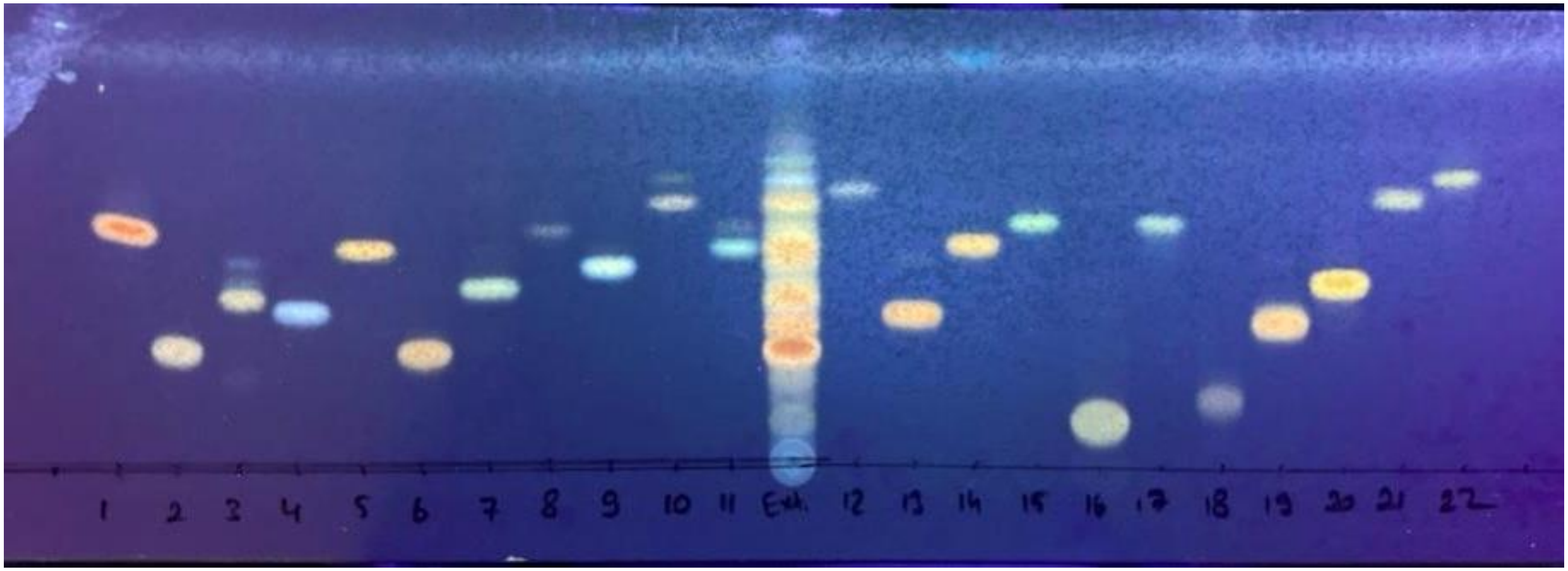

Figure S 212 Thin layer chromatogram of the isolated metabolites and the EtOAc extract [Silica gel, Mobile phase: 87.5:12.5 ($CHCl_3$:MeOH)]

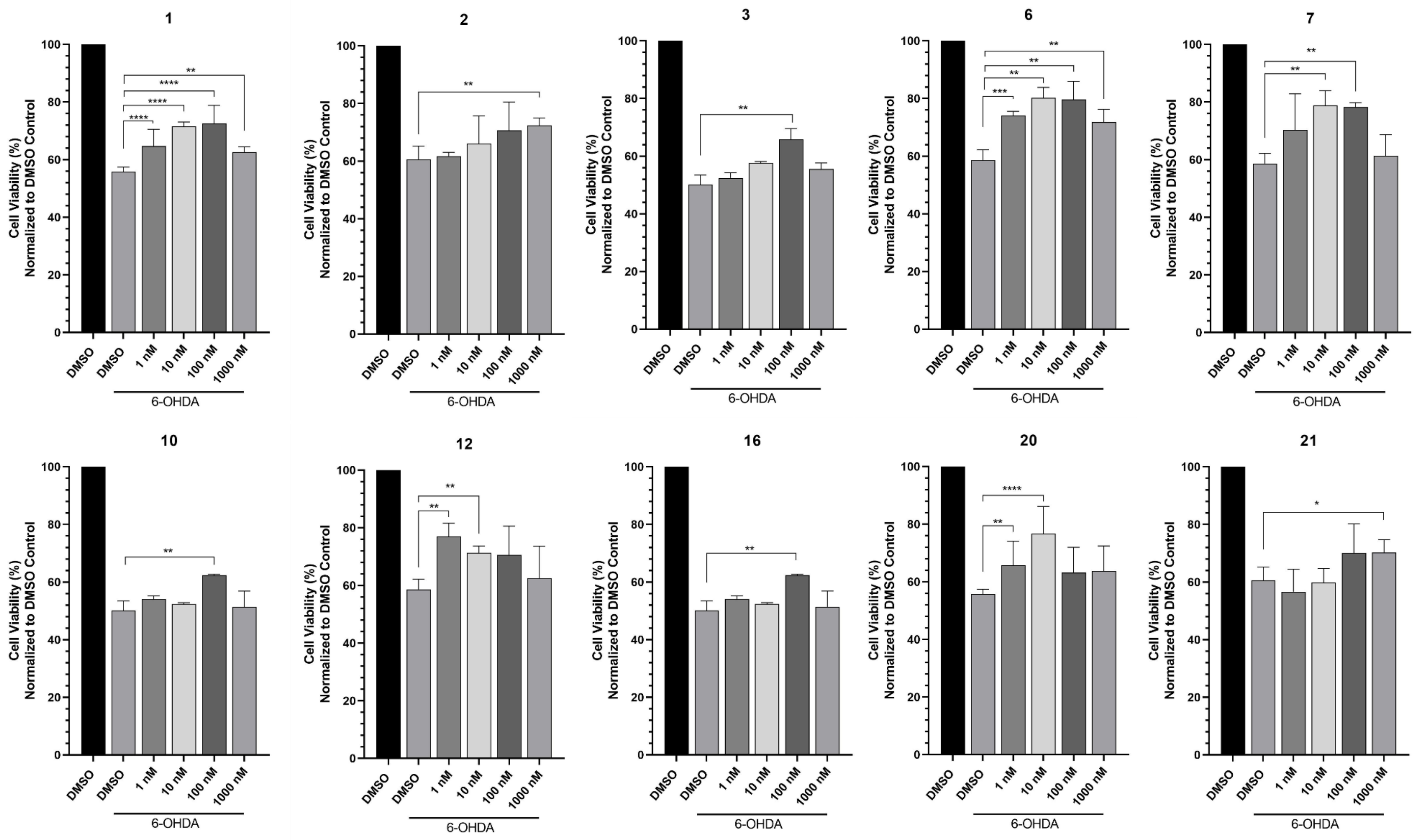

Figure S 213 Neuroprotective activity of selected compounds against 6-OHDA toxicity Error bars are the standard deviations (n = 3). p-Values were calculated with respect to 6-OHDA-treated cells (*p < 0.05, **p < 0.01, ***p < 0.001, ****p < 0.0001)
